# Suppressing acoustomigration and temperature rise for high-power robust acoustics

**DOI:** 10.1038/s41467-026-72102-7

**Published:** 2026-04-22

**Authors:** Fangsheng Qian, Shuhan Chen, Wei Wei, Jiashuai Xu, Kai Yang, Junyan Zheng, Zijun Ren, Xingyu Liu, Yansong Yang

**Affiliations:** Department of Electronic and Computer Engineering, The Hong Kong University of Science and Technology, Hong Kong, China

**Keywords:** Electrical and electronic engineering, Mechanical engineering

## Abstract

High-frequency acoustic wave transducers, favored for their compact size, are not only dominating mobile handsets but are also expanding into various interdisciplinary fields. However, as strong vibration can “shake off” substances and produce heat, a long-standing bottleneck has been the ability to harness acoustics under high-power loads, especially for interdigital-transducer-based surface acoustic wave devices. To suppress three fundamental mechanisms: self-heating, thermal instability, and acoustomigration, we propose a layered acoustic wave platform utilizing a quasi-infinite multifunctional top layer that redefines mechanical and thermal boundary conditions. The proposed transducer achieves a 70% reduction in temperature rise, a temperature coefficient of frequency of −13 ppm/°C, and an unprecedented threshold power density of 45.61 dBm/mm^2^ — over one order of magnitude higher than that of state-of-the-art thin-film surface acoustic wave counterparts. This architecture enables scalable deployment of high-power acoustic wave components in space-constrained hybrid platforms and opens the functional diversification of acoustic wave transducers.

## Abstract (web summary)

High-power acoustic wave transducers face three challenges: self-heating, thermal instability, and acoustomigration. This study presents a layered acoustic wave (LAW) architecture that boosts power density via thermal and mechanical boundary engineering.

## Introduction

**Fig. 1:**
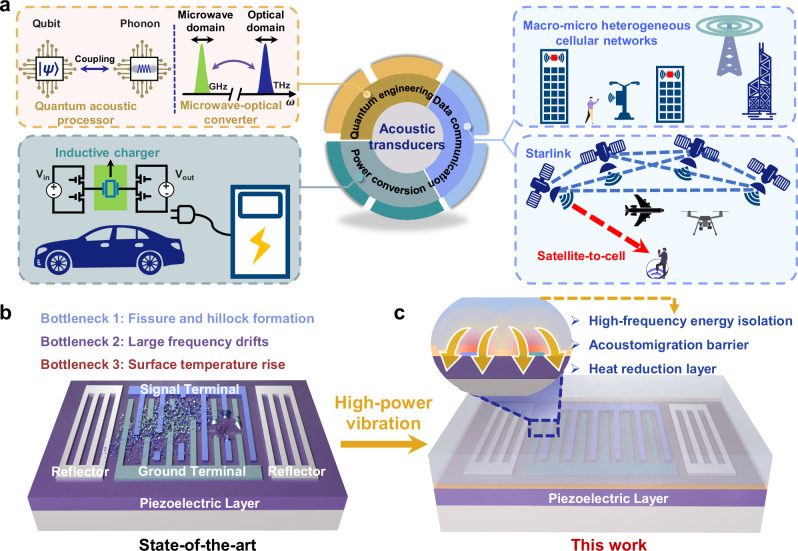
Conceptualization of LAW transducers for ultra-high power capacity. **a** Acoustic transducers are ubiquitous, leveraging their compact size and strong multi-domain coupling for applications in quantum engineering, power conversion, and next-generation wireless communication. However, this miniaturization is a double-edged sword: it intensifies power density and increases susceptibility to device instabilities arising from nonlinear effects and self-heating. **b** Schematic illustrating TF-SAW transducer failure mechanisms under high-power loads: surface strain within the piezoelectric cavity generates excessive mechanical stress in IDTs, exceeding material-dependent thresholds and inducing metallic cluster migration/diffusion. Thermal management in state-of-the-art piezoelectric-on-insulator (POI) architecture is constrained by the poor thermal conduction at the top boundaries (piezoelectric layer/air interface). Furthermore, elasticity and acoustic velocity dispersion caused by thermal expansion lead to significant frequency drifts. **c** Conceptual perspective-view schematic of a LAW transducer on a POI platform. Mitigating device instabilities in robust-critical scenarios, such as quantum acoustodynamics and infrastructure for electrified transport and wireless communication networks, requires a key innovation: rethinking top thermal-mechanical boundary conditions to channel heat away and increase the acoustomigration barrier in a concise solution.

Acoustics has emerged as a transformative platform across quantum engineering^1,2^, sustainable power transfer infrastructure^3–5^, next-generation wireless communication networks^6–8^, and other interdisciplinary fields^9,10^ (Fig. 1a). For acoustic transducers, its unique ability to confine GHz-frequency mechanical energy into chip-scale dimensions underpins critical advances: enabling coherent phonon-mediated qubit coupling for high-fidelity quantum information processing^11,12^; powering 5 G/6 G RF signal processing in billions of smartphones and micro base stations; and driving high-power satellite links—exemplified by SpaceX’s 2025 acquisition of Akoustis Technologies to develop high-performance acoustic filters for direct-to-cell networks, where power handling defines orbital link viability. Beyond communications, the inherently high operating frequency and power density of acoustic wave transducers also make them promising candidates for acoustic wave actuators and non-magnetic energy reservoirs in smart energy transfer systems, offering new pathways for more compact and sustainable power conversion infrastructure. In the emerging interdisciplinary fields, strong acoustoelectric interactions between mobile carriers and propagating phonons could produce non-reciprocal amplification and allow all-acoustic RF signal processing, including circulators and switches^9,13^. In addition, acoustic wave transducers feature orders-of-magnitude smaller footprints than circuit quantum electrodynamics devices at similar frequencies and can also support quantum control of mechanical motion in the strong-coupling regime for probing quantum foundations in complex systems^14^.

Despite enabling versatile acoustoelectric functionalities of broad relevance to the electronics community, these acoustic transducers confront a fundamental trade-off: while engineered as efficient energy reservoirs, their extreme energy density triggers nonlinear thermal instabilities, causing catastrophic thermal runaway and irreversible device failure. Among them, bulk acoustic wave (BAW) transducers, utilizing a thickness-extensional (TE) mode, can achieve higher power density owing to favorable stress profiles and lower electrode resistance. However, the very nature of this vertically confined, standing-wave mode limits its use in broader applications that rely on propagating acoustic waves for in-plane coupling, such as integrated sensing, microfluidics^15^, and hybrid acoustic-optics systems^16^. In contrast, surface acoustic wave (SAW) transducers offer distinct advantages in planar integrability, fabrication simplicity, and natural coupling with other physical fields. Therefore, enhancing the power density of the more versatile, interdigital-transducer (IDT)-based SAW platform is a critical step to unlock acoustic transducers’ full potential across these diverse domains. Fundamentally, four physical phenomena prevent power-scaling into large-signal regimes: (1) acoustomigration, driven by mechanical stress and self-heating effects, degrades electromechanical coupling by transporting metallic clusters; (2) thermoelastic instability, manifesting as frequency drift from temperature-dependent acoustic velocity and thermal expansion mismatch; (3) mechanical stress accumulation, inducing thin-film cracking or delamination; and (4) electromigration, where high current densities in the IDT electrodes induce atomic diffusion via electron momentum transfer, leading to void formation and eventual open-circuit failure. Additionally, arc discharging, another parasitic effect arising from pyroelectric charging, can be significantly mitigated by material engineering^17^. However, the first three phenomena have persisted as critical challenges for decades, constrained by the intrinsic material and architectural limitations of existing acoustic transducers. As illustrated in Fig. 1b, high-power vibration-induced mechanical stress on state-of-the-art thin-film surface acoustic wave (TF-SAW) transducers leads to visible structural failure, including severe cracking of the piezoelectric layer, delamination of metal electrodes, and widespread hillock formation in the IDT region. Early microscopy studies showed the hillock elevation of several micrometers under overloaded stress conditions, compared to a baseline root-mean-square roughness of only 2.5 nm in pristine devices^18^. Therefore, efficiently suppressing hillocks and voids formation ask for a novel design paradigm. Additionally, thermal management of current TF-SAW transducers relies on a high-thermal-conductivity substrate positioned beneath the active region. However, another key limitation of traditional configuration lies in the inefficient heat transfer from localized hot spots, primarily due to the piezoelectric layer’s inherently low thermal conductivity, hindering heat flow from the active area to the substrate. Therefore, the contribution of high-thermal-conductivity substrates to heat dissipation is minimal.

To march acoustic wave components toward high-power operations scenarios, overcoming the aforementioned limitations requires a concerted set of design strategies. These include: (a) Disrupting grain boundary continuity or introducing additive atoms to suppress fissure and hillock formation during the vibration; (b) Implementing multidirectional heat reduction routes (top, sides, and bottom) to dissipate heat from the active regions; (c) Incorporating additional temperature compensating layer to stabilize high-frequency vibration across ultra-wide temperature ranges.

In this article, we implement the new design strategies by introducing a layered acoustic wave (LAW) architecture, which comprises a piezoelectric cavity and an isotropic SiO_2_ isolation layer with thickness *h*, sandwiched between a high-velocity substrate and a quasi-infinite multifunctional overlayer (Fig. 1c). This architecture prioritizes acoustic energy confinement and mechanical stress redistribution through engineered acoustic boundaries, with constituent materials selected through a holistic electrical–mechanical–thermal co-design process. The key innovation is the decoupled yet synergistic function of each layer: the thin SiO_2_ layer provides electrical isolation and mitigates interfacial stress, while the quasi-infinite thick silicon (Si) film, far thicker than typical acoustic wavelengths, serves as a triple role. It acts as (1) a mechanical confiner that reshapes the von Mises stress distribution, reducing peak stress at the IDT/piezoelectrics interface; (2) an integrated heat-spreader through the top and side surfaces of the transducers with the thermal conductivity exceeding that of air by approximately two orders of magnitude^19^, creating vertical dissipation pathways that drastically lower operating temperatures under high power; and (3) a thermal expansion compensator that improves temperature stability without sacrificing effective electromechanical coupling (*k*_t_^2^) compared to traditional temperature-compensated SAW (TC-SAW) transducers. By co‑designing the electrical, thermal, and mechanical boundary conditions, we achieve simultaneous enhancement in power density, thermal management, and temperature stability—without introducing complex fabrication steps or the typical performance trade‑offs associated with multi‑layer stacks. This integrated approach enables a fundamental shift from merely mitigating power‑induced failure to actively engineering the transducer’s intrinsic power‑handling ceiling.

To directly evaluate the fundamental power-handling enhancement enabled by our architectural innovation, we conducted standardized high-power reliability experiments at the transducer level. This approach isolates the performance of the transducer design itself from the confounding variables inherent to system-level measurements, such as layout‑dependent power flow distribution, electromagnetic parasitic effects, and potential current/voltage division effects^20^, thereby providing a direct benchmark of the intrinsic advance in boundary condition engineering. At this component level, the LAW transducers exhibited a 70% reduction in steady-state temperature rise compared to their TF-SAW counterparts, highlighting significantly improved thermal management. Furthermore, the LAW architecture achieved a threshold injected power density of 45.61 dBm/mm^2^, representing a 12.73-fold enhancement over conventional TF-SAW transducers (34.56 dBm/mm^2^). This advancement extends to cryogenic environments, with the LAW platform reaching a threshold of 49.45 dBm/mm^2^ (88.11 W/mm^2^) at −85 °C, demonstrating exceptional high-power robustness in low-temperature conditions. Our work addresses these aforementioned challenges head-on, demonstrating an unprecedented, one order-of-magnitude improvement in power capacity and thus enabling the transition of acoustics from small-signal to large-signal regimes, for the first time. Critically, this architecture and design paradigm can be broadly extensible to other material systems, opening a broad pathway for performance‑driven diversification in next‑generation acoustic technologies.

## Results

### Boundary redefining

**Fig. 2:**
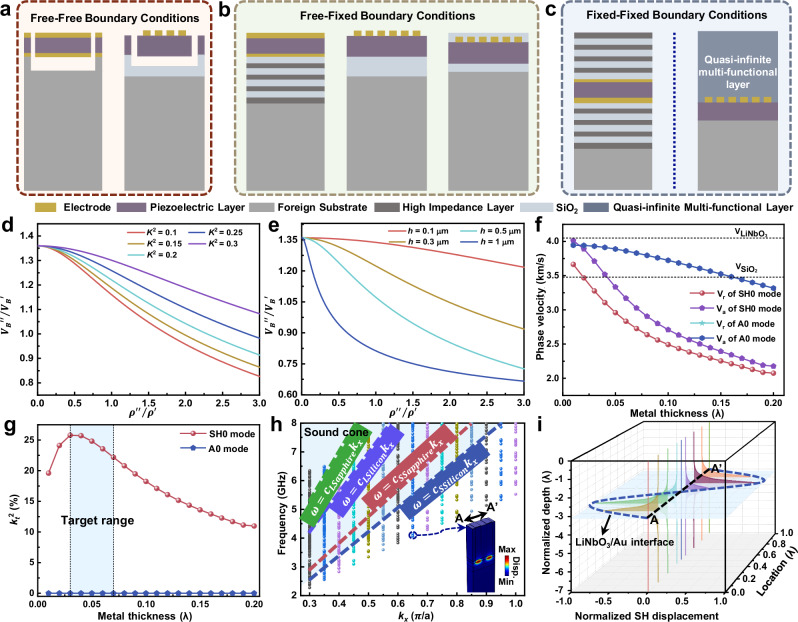
Theoretical analysis and calculation for LAW transducers design. **a** Representative “free-free” acoustic boundary platforms (e.g., thin-film bulk acoustic resonators [FBARs] and antisymmetric Lamb wave resonators), requiring a cavity release process to isolate the transducer from the substrate, forming an air-transducer-air configuration. **b** “Free-fixed” boundary platforms (e.g., solidly mounted FBAR [SMR], TF-SAW, and temperature-compensated SAW [TC-SAW]), where the transducers are anchored on substrates to create an air-transducer-substrate architecture. **c** Two realizations of “fixed-fixed” boundaries for acoustic confinement: Dual-Bragg reflectors, requiring precise thickness control of alternating layers; Our layered acoustic wave architecture, employing a simplified, quasi-infinite single overlayer. **d** Acoustic velocity ratio$\,{{V}_{B}}^{{\prime} {\prime} }/{{V}_{B}}^{{\prime} }$ versus mass density ratio ${\rho }^{{\prime} {\prime} }/{\rho }^{{\prime} }$ in the LAW transducer with varying *K*^2^ in LiNbO_3_. **e** Dependence of ${{V}_{B}}^{{\prime} {\prime} }/{{V}_{B}}^{{\prime} }$ on ${\rho }^{{\prime} {\prime} }/{\rho }^{{\prime} }$ in LAW transducers with modulated SiO_2_ interlayer thickness (100 nm–1 µm). **f** Eigenmode analysis of phase velocities for the SH0 and A0 modes as functions of normalized Au thickness. **g** Calculated *k*_*t*_^2^ dependence on normalized Au thickness. **h** Dispersion relations of LAW transducers showing sub-6 GHz frequency scalability. *c*_LSapphire_, longitudinal velocity of sapphire; *c*_LSilicon_, longitudinal velocity of silicon; *c*_SSapphire_, shear velocity of sapphire; *c*_SSilicon_, shear velocity of silicon; ${k}_{x}$, wavevector component along the propagation direction (*x*-axis). Inset: Targeted SH mode with stress concentration at the LiNbO_3_/Au interface; allowed acoustic waves in the sound cone form a continuum $\omega > c\cdot {k}_{x}$, which can freely radiate into the bulk. **i** Cross-sectional SH-mode displacement profiles at 8 equally spaced positions (*λ*/8 intervals) along A–A’, illustrating standing-wave formation in the cavity and > 90% energy attenuation within 1*λ* depth.

Following the long-standing consensus of the required top air boundary, state-of-the-art acoustic transducers can be classified into two types: (i) “Free-Free” boundary conditions (Fig. 2a) and (ii) “Free-Fixed” boundary conditions (Fig. 2b). As the most straightforward strategy, Type I devices, free-free platforms, such as thin-film bulk acoustic resonators (FBARs) and Lamb wave devices^21–28^, achieve strong energy confinement but suffer from mechanical fragility and electrical instability due to their suspended structure. Based on the dispersion relationships of waves, acoustics wave in each medium features a specific cut-off frequency below which waves propagate through the medium rather than reflect. Therefore, Type II free-fixed platforms, such as solidly mounted FBARs (SMRs) and TC-SAW devices^29–36^, mitigate some mechanical challenges by anchoring to high-velocity substrates or employing Bragg reflectors, yet they remain hampered by insufficient heat dissipation and persistent parasitic effects. Notably, TC-SAW architectures attempt to stabilize frequency response via a thin SiO_2_ overlay. This approach, however, introduces inherent trade-offs: the nanoscale thickness of the SiO_2_ layer results in poor thermal conductivity (~0.1 W m^−1^K^−1^), limiting effective heat removal from the active region^37^. Furthermore, the acoustic properties of SiO_2_ introduce an inherent trade-off as the layer thickens for compensation: its low acoustic phase velocity undermines energy confinement and sacrifices electromechanical coupling coefficient (*k*_*t*_^2^). Consequently, achieving temperature stability in this approach necessitates a fundamental compromise with the transducer’s electromechanical conversion efficiency. While high-thermal-conductivity substrates mitigate certain thermal challenges, persistent acoustomigration and significant thermal instability persist and do not favor Type II devices as a suitable candidate for achieving the ideal performance metrics required in Fig. 1a. As a result, these conventional approaches fall short of the ideal combination of high-power handling, thermal stability, and minimal acoustic loss required for next-generation applications. While the conceptual use of an upper cladding to create the “fixed-fixed” boundary condition has been explored^38–40^, primarily in BAW resonators, for purposes such as enhancing quality factor (*Q*) or *k*_*t*_^2^. These prior implementations often rely on complex multi-layer Bragg reflectors (Fig. 2c), which require precise thickness control and, critically, introduce multiple thermal interfaces that impede vertical heat flow, exacerbating thermal management challenges^41^. Unlike them, the proposed LAW architecture leverages a simplified, quasi-infinite medium that firstly engineers the von Mises stress distribution and creates an efficient thermal dissipation path to revolutionize power handling in IDT-based SAW platforms, directly addressing the root causes of power-induced failure without the thermal penalty of multi-interface stacks (Fig. 2c). This advancement offers inherent advantages in planar integrability and coupling to other physical fields (e.g., optics or fluids) compared to vertically confined BAW modes, making its power resilience a key enabler for broader applications.

Achieving high-quality electromechanical resonance under Fixed-Fixed boundary conditions requires new thinking about both the lower (substrate) and upper (cladding) boundaries. Key criteria for new boundary design include: (i) effective acoustic energy confinement within the piezoelectric layer; (ii) strong drift barriers to suppress acoustomigration; (iii) sufficient electrical insulation to minimize dielectric loss; (iv) multidirectional heat dissipation exceeding air convection; (v) negligible mechanical damping to preserve high quality factors; and (vi) field concentration using low-permittivity adjacent layers. While these guidelines are qualitative, they provide a systematic foundation for electrical–mechanical–thermal co-design. Compared to other common alternatives like lithium tantalate (LiTaO_3_) and aluminum nitride (AlN), lithium niobate (LiNbO_3_) exhibits a larger electromechanical coupling (*K*^2^) behavior. This property makes LiNbO_3_ an optimal choice for the piezoelectric layer in our LAW transducer implementation, enabling enhanced performance in wideband signal processing and high-frequency non-magnetic power conversion applications. For the foreign substrate, sapphire is selected for the bottom boundary due to its low cost and substantial acoustic velocity mismatch with the LiNbO_3_ layer for shear horizontal (SH) modes, enabling efficient energy confinement and simplifying the heterostructure to a three-layer configuration. A thin SiO_2_ layer is uniformly deposited atop LiNbO_3_ to provide electrical isolation and residual stress relief for the following top quasi-infinite layer.

To analyze and strengthen the SH wave vibration in the LAW architecture, governing equations for the three-layer stack are derived using recursive relations^42,43^, assuming ideal interfacial adhesion. The detailed derivation process is presented in Supplementary Section 1. Figure 2d illustrates the dependence of the slow shear acoustic velocity ratio ${{V}_{B}}^{{\prime} {\prime} }/{{V}_{B}}^{{\prime} }$ (SiO_2_ to top quasi-infinite layer) on the corresponding mass density ratio of ${\rho }^{{\prime} {\prime} }/{\rho }^{{\prime} }$, across varying *K*^2^ in LiNbO_3_. Non-leaky SH mode is confined below the plotted curve in Fig. 2d, translating that there is a lower limit of ${{V}_{B}}^{{\prime} }$ for effective top boundary design. And enhanced *K*^2^ broadens the SH mode’s existence range at fixed ${\rho }^{{\prime} {\prime} }/{\rho }^{{\prime} }$. Figure 2e demonstrates the influence of SiO_2_ thickness on the existing range of SH-LAW. The threshold for ${{V}_{B}}^{{\prime} {\prime} }/{{V}_{B}}^{{\prime} }$ decreases with increasing *h*, reflecting most acoustic fields concentrate near the LiNbO_3_ surface. It can be concluded that the lower limits of ${{V}_{B}}^{{\prime} {\prime} }/{{V}_{B}}^{{\prime} }$ obeys a negative relationship with *h*. For *h* = 1 µm, the calculated ${{V}_{B}}^{{\prime} {\prime} }/{{V}_{B}}^{{\prime} }$ curve decreases sharply as ${\rho }^{{\prime} {\prime} }/{\rho }^{{\prime} }$ increases, constraining viable top-layer materials. Material candidates meeting these criteria include *α*-Si, Si_*x*_N_*y*_, AlN, Al_2_O_3_ for their mechanical properties. And the metals can be Cu, Au, Pt, Ag for making the slow shear acoustic velocity (${V}_{B}$) smaller than 3500 m/s. Au is chosen for IDTs due to its chemical inertness during SiO_2_ deposition. Beyond stability, the choice of dense electrode material, such as Au, provides additional benefits for the transducer’s *k*_*t*_^2^ performance. The IDT structure inherently shifts the mechanical stress field from the piezoelectric layer towards the electrode layer. A high-density electrode like Au enhances mass loading and couples more effectively with this stress field and the applied electric field, leading to a higher achievable *k*_*t*_^2^. Additionally, *α*-Si serves as the top quasi-infinite layer for its fast shear velocity, positive temperature coefficient of elastic stiffness, and low dielectric constant^44^. Additionally, the combination of the sandwiched SiO_2_ and upper *α*-Si layer contributes to relatively large coupling thanks to the negative $\Delta \left(V\right)$. Simulated performance comparisons of LAW transducers with and without optimized boundaries are detailed in Supplementary Section 2.

To further refine the structural design of the LAW transducers, quasi-3D eigenmode simulations were performed in COMSOL Multiphysics by systematically varying the Au electrode thickness. This analysis evaluates its impact on phase velocity and *K*^2^ for the fundamental SH (SH0) and antisymmetric (A0) modes, as presented in Fig. 2f, g. The slow shear bulk wave velocities of LiNbO_3_ and amorphous SiO_2_ are indicated in Fig. 2f. To maximize the coupling and prevent bulk wave leakage, the phase velocity of SH mode is engineered to remain below the *V*_SiO2_ by slightly leveraging the mass loading effect of Au^45,46^. This translates to an increased acoustic impedance mismatch between LiNbO_3_ and SiO_2_ at optimal thickness, contributing to strong SH-wave reflection, which corresponds to a mode conversion from a leaky Love wave to a nonleaky SH wave^47^. However, thicker Au electrodes shift the SH stress field upward, indicating a moderate coupling, as confirmed by Fig. 2g. A targeted Au thickness range of 0.04–0.07*λ* is then identified.

Figure 2h presents the dispersion relation for the acoustic modes in the LAW configuration. Owing to the structure’s periodicity in the *x*-direction, only the *k*_*x*_ component of the wave vector ***k*** is conserved throughout the entire system. Each point in Fig. 2h represents an eigenmode of the LAW device. The asymmetric boundary conditions partition the dispersion diagram into five regions bounded by sound lines $\omega=c\cdot {k}_{x}$, where *c* denotes the longitudinal or shear acoustic velocity in either the sapphire substrate or quasi-infinite Si layer. Below the lowest shear sound line, elastic waves are prevented from radiating into the quasi-infinite *α*-Si layer or sapphire substrate. Such localized modes exhibit evanescent exponential decay along the depth direction. Above the highest sound line^48,49^, elastic waves propagate freely in the bulk substrate and quasi-infinite top layer, defining this region as the “sound cone”. Within the three intermediate regions, elastic waves of distinct modes exhibit unidirectional leakage into silicon.

Overall, the stacked multilayer acoustic waveguides enable dispersion-engineered SH-LAW transducers to operate in the sub-6 GHz frequency range. This complete vertical boundary architecture, optimized for actual material properties and adjacent layer thicknesses, maximizes acoustic energy confinement within the piezoelectric cavity and ensures rapid exponential decay of elastic energy away from the active region, as shown in Fig. 2i.

### Multifunctional implementation and characterization of LAW transducers

The performance of LAW transducers is governed by their 3D geometry and constituent material properties. In particular, the large *K*^2^ in LiNbO_3_-based acoustic transducers, while enabling wideband operation, also increases their sensitivity to temperature fluctuations. The temperature coefficient of frequency (TCF), a critical parameter for spectral stability in practical environments, can be expressed as: TCF = TCV−CTE, where TCV is the temperature coefficient of acoustic velocity and CTE represents the coefficient of thermal expansion along wave propagating directions. Minimizing TCF requires both suppression of material deformation and reduction of TCV across operational temperatures.

Although SiO_2_ is exploited in TC-SAW devices for temperature compensation, its efficacy depends critically on the Si–O–Si bond angle, which is highly sensitive to deposition conditions. This sensitivity complicates precise modeling of SiO_2_’s temperature-dependent elasticity, necessitating experimental isolation of material-specific TCF contributions in defining geometric specifics of LAW transducers (see Supplementary Section 3). Furthermore, increasing SiO_2_ thickness narrows the LAW existence range, as demonstrated by Fig. 2e. After comprehensive consideration, a 270 nm amorphous SiO_2_ layer was selected to balance frequency stability, energy confinement, and electrical isolation.

Thermal management also plays a crucial role in high-power operation. Considering the thermal boundary conditions, heat-spreading in amorphous materials exhibits thickness-dependent behavior: Below 100 nm, *α*-Si thermal conductivity is dominated by diffusion (non-propagating modes), transitioning to propagating-dominated transport above 100 nm^50^. Previous experimental investigations confirm that both in-plane thermal conductivity ${k}_{{\rm{||}}}$ and cross-plane thermal conductivity ${k}_{\perp }$ increase rapidly as film thickness approaches 2 µm^51^.

Besides, beyond a thickness of ~ 0.6 µm, the *α*-Si cladding layer acts as a semi-infinite medium from the perspective of the guided mode, making the frequency independent of further thickness increases (Supplementary Section 4). From the standpoint of thermal boundary, the quasi-infinite layer was therefore designed to exceed 2 µm in thickness to provide efficient thermal dissipation.

**Fig. 3:**
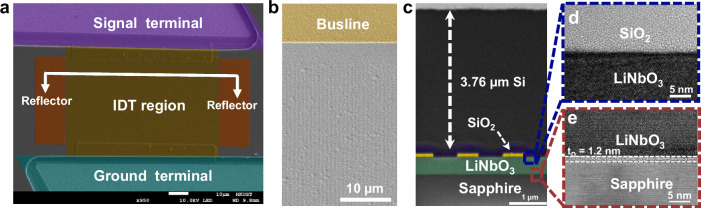
Boundary discussion and implementation for LAW transducers. **a** False-color top-view SEM image of a fabricated LAW transducer. **b** Enlarged SEM image of the active region, showing a void-free surface. **c** Cross-sectional SEM image of the IDT region, showing layers: 3.76 µm α-Si, 270 nm SiO_2_ (purple), 85 nm Au (yellow), 500 nm LiNbO_3_ (green), and sapphire substrate. Cross-sectional scanning transmission electron microscopy (STEM) images of the (**d**) SiO_2_/LiNbO_3_ interface in the blue rectangle in (**c** and **e**), LiNbO_3_/sapphire interface in the red rectangle in (**c**).

The structural integrity and interface quality of the fabricated LAW transducers were confirmed through a series of microscopy analyses. Figure 3a, b display top-view scanning electron microscope (SEM) images of the fabricated LAW transducers, with Fig. 3b detailing the IDT active region. Cross-sectional SEM analysis (Fig. 3c) confirms a void-free 3.76 µm-thick *α*-Si layer deposited via conformal plasma-enhanced chemical vapor deposition (PECVD). This process ensures uniform SiO_2_ insulation and robust interfacial adhesion to the LiNbO_3_ substrate, enhancing thermal dissipation through maximized interfacial contacts. Transmission electron microscopy (TEM) image in Fig. 3d reveals a sharp SiO_2_/LiNbO_3_ interface with a dense, defect-free metal migration barrier. Further, the LiNbO_3_/sapphire bonded interface (Fig. 3e) exhibits minimal interfacial damage (1.2 nm), with both the LiNbO_3_ thin film and sapphire substrate retaining high crystallinity post-fabrication. Energy-dispersive spectroscopy (EDS) elemental mapping of the scanning transmission electron microscopy (STEM) image confirms uniform distribution of Si, O, Au, Nb, and Al across the transducer structure, as detailed in Supplementary Section 5.

**Fig. 4:**
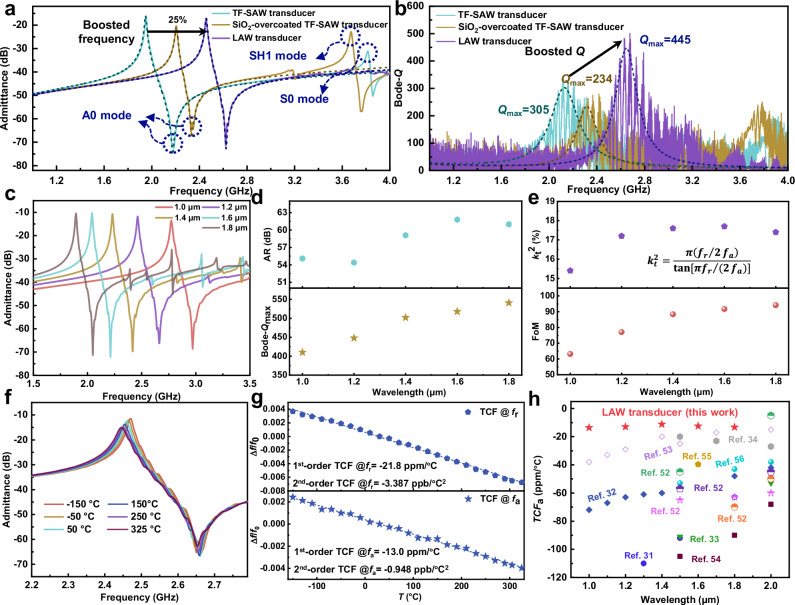
Measurement results from the LAW transducers. Measured (**a**) admittance and **b** corresponding Bode-*Q* curves of transducers in conventional TF-SAW, SiO_2_-overcoated TF-SAW, and LAW configurations. **c** Admittance curves of LAW transducers as a function of wavelength. Extracted metrics include (**d**) admittance ratio (AR) and Bode-*Q*_max_ in the passband, **e** *k*_*t*_^2^ and figure of merit (FoM) for LAW transducers across varying wavelengths. **f** Temperature-dependent admittance responses of a LAW transducer (*λ* = 1.2 µm) measured over an ultra-wide temperature range from −150 to 325 °C. **g** Extracted relative frequency drifts under temperature variation, with the first-order and second-order TCF at *f*_*r*_ and *f*_*a*_. **h** Scatter plot comparison of TCF_*a*_ for the LAW transducers in this work and for advanced TF‑SAW transducers reported in the literature. Data points are compiled from the following references: LiNbO_3_/SiO_2_/Quartz^31^; LiNbO_3_/SiC^32^; LiNbO_3_/SiO_2_/Si^33^; SiO_2_/LiNbO_3_^34^; LiNbO_3_/69°Y90°X-Quartz, LiNbO_3_/60°Y90°X-Quartz, LiNbO_3_/AT-Quartz and LiNbO_3_/YZ-Quartz^52^; LiNbO_3_/SiO_2_/SiC^53,54^; LiNbO_3_/SiC^55^; and LiNbO_3_/SiO_2_/poly-Si/Si^56^.

To isolate the mechanical damping effects of *α*-Si and SiO_2_, conventional and SiO_2_-overcoated TF-SAW transducers were measured after the fabrication of each transducer configuration. A standardized wafer cleaning protocol was performed prior to each subsequent fabrication step to remove potential contaminants introduced during measurements. Befitting from the design of acoustic boundary conditions, the strong plate modes (in-band A0 mode, S0, and SH1 mode) inherent to the baseline TF-SAW and SiO_2_-overcoated TF-SAW structures are suppressed simultaneously after adding the *α*-Si multi-functional layer. These results can be directly attributed to the tailored sound cone and stress field, providing more freedom in acoustic boundary designs on the LAW platform (Fig. 4a). The in-band spurious modes on three platforms are transversal modes arising from the waveguiding effect in the aperture direction. The suppression strategies are discussed in Supplementary Section 6. Furthermore, the identical admittance observed across all three transducer configurations at frequencies distant from the resonant frequency (*f*_*r*_), indicates unaffected static capacitance by the top boundary, confirming effective electric field confinement inside the piezoelectric cavity. Compared to the TF-SAW transducer, measured admittance curves of SiO_2_-overcoated TF-SAW transducers exhibit an upward frequency shift attributed to velocity compensation. However, the additional SiO_2_ overlayer presents degraded device performances, including the TCF, *k*_*t*_^2^ and *Q*. Subsequent deposition of the high-velocity Si cladding layer continuously increases *f*_*r*_ from 2.205 GHz (SiO_2_-overcoated configuration) to 2.458 GHz and boosts Bode-*Q*_max_ from 234 (SiO_2_-overcoated TF-SAW configuration) to 445, as illustrated in Fig. 4b. The *Q*-factor enhancement stems from stress field manipulation enabled by the high-velocity *α*-Si cladding layer. Detailed performance merits of these three types of transducers are summarized in Supplementary Sections 7 and 8. Following that, LAW transducers with a range of lateral wavelengths (*λ* ranging from 1.0 to 1.8 µm) were tested, as presented in Fig. 4c. The measured admittance ratio (AR), defined as the difference between admittance magnitudes at *f*_*r*_ and the anti-resonant frequency (*f*_*a*_) of each transducer, demonstrating that the LAW architecture achieves AR values comparable to or exceeding those of state-of-the-art TF-SAW devices with the same wavelength on LiNbO_3_/SiC platform^17^, as shown in Fig. 4d. It can be observed that other spurious modes occur beyond the targeted SH0 mode, especially for wavelengths larger than 1.2 µm. These are higher-order bulk waves scattered at vertical acoustic boundaries, which can be easily suppressed via further mechanical bandgap engineering, as detailed in Supplementary Section 6. Additional key performance metrics, including quality factor (Bode-*Q*_max_), *k*_t_^2^, and figure of merit (Bode-*Q*_max_ × *k*_t_^2^), are also summarized in Fig. 4d, e. Notably, the highest measured Bode-*Q*_max_ inside the passband is 559 at *λ* = 1.8 µm. While the moderate *k*_*t*_^2^ arises from dispersive stress field in the upper layer, the LiNbO_3_ layer itself maintains strong acoustic field confinement, with most LAW transducers exhibiting *k*_*t*_^2^ ˃ 17%, confirming sufficient electromechanical coupling for wideband applications. The optimized boundaries yield a FoM of 94 at *λ* = 1.8 µm, with only 7.9% degradation relative to TF-SAW counterparts. Device performance remains stable for *λ* ≥ 1.2 µm, and *Q*_3dB_ at *f*_*r*_ for all devices shows little variation, indicating minimal electrical loss as *λ* decreases from 1.8 µm to 1.0 µm. Below 1.2 µm, reduced coupling arises from lateral electric field dispersion, suggesting future optimization via LiNbO_3_ thickness adjustment.

Temperature stability was systematically characterized using a vacuum cryogenic probe station over an ultra-wide range (−150 to 350 °C). Figure 4f shows the admittance response of a LAW device (*λ* = 1.2 µm), presenting a 12 dB increase in AR as temperature decreases from 350 to −150 °C, which arises from reduced mechanical damping and ohmic loss. Note that *f*_*r*_ and *f*_*a*_ increase linearly with cooling, yielding fractional frequency variations proportional to temperature (Fig. 4g). Neglecting third and higher-order terms, the TCF can be determined based on the following formula: $({f}_{T}-{f}_{0})/{f}_{0}={A}_{1}\left(T-{T}_{0}\right)+\,{A}_{2}{\left(T-{T}_{0}\right)}^{2}$, where ${A}_{1}$ and ${A}_{2}$ refer to first- and second-order TCF, and room temperature ${T}_{0}$ is denoted as the reference temperature. Fitted TCF for *f*_*r*_ are ${A}_{1}=\,$−21.8 ppm/°C and ${A}_{2}=\,$−3.387 ppb/°C^2^, while those for TCF at *f*_*a*_ (TCF_*a*_) are ${A}_{1}=\,$−13 ppm/°C and ${A}_{2}$ = −0.948 ppb/°C^2^. Enhanced TCF in the LAW transducer arises from two mechanisms: suppression of thermal expansion provided by the extra upper interlayer force and modulation of TCV, which is highly dependent on the temperature coefficient of piezoelectric and elastic constants. More importantly, the phase velocity of SH mode exhibits dispersion with *λ*, that is, variation in *λ* caused by thermal expansion or contraction inherently alter the TCV term^52^. In this case, TCF can be captured by: ${\rm{TCF}}\equiv \frac{1}{f}\frac{{\rm{d}}f\left(T\right)}{{\rm{d}}T}=\frac{\lambda }{{v}_{p}}\frac{{\rm{d}}}{{\rm{d}}T}\frac{\lambda (T)}{{v}_{p}(T)}$, suggesting a coupling between TEC and TCV. As shown in Fig. 4h, LAW transducers achieve superior temperature stability and minimal dispersion compared to state-of-the-art SH-SAW devices^31–34,52–56^, marking the lowest reported TCF values for LiNbO_3_-based transducers. The architecture decouples TEC and TCV, mitigating dispersion-induced TCF instability. Comprehensive TCF at *f*_*r*_ (TCF_*r*_) comparison between this work and state-of-the-art works are presented in Supplementary Section 9. Compared to SiO_2_-overcoated TF-SAW devices in Supplementary Table S1, our LAW architecture improves the first- and second-order TCF_*r*_ performance from −117.51 to −21.8 ppm/°C, stemming from α-Si’s positive TCV and efficient interlayer stress compensation.

### Power handling capability evaluation

To rigorously assess power capacity across different acoustic architectures using the setup described in Supplementary Section 10, several aspects in the evaluation process should be taken into consideration to allow for accelerated testing and ensure a fair comparison. First, a fundamental prerequisite for a meaningful comparison is a benchmarking method that isolates the intrinsic impact from architectural innovation. Power handling measured at the filter level, however, reflects a system‑level property influenced by numerous design‑specific factors, such as cascading resonators, power‑routing layout, matching networks, and test conditions. These variables can obscure the intrinsic improvement attributable solely to the core transducer technology, as comprehensively discussed in Supplementary Section 11. We thus conducted high-power testing at the individual transducer level to eliminate confounding effects from multi-transducer interactions within filters and enable a direct comparison of the fundamental design. Second, a direct comparison of power-handling performance requires TF-SAW and LAW transducers fabricated from an identical layout. This control is essential because the static capacitance (*C*_0_) governs the frequency‑dependent von Mises stress profile, which in turn dictates the intrinsic, architecture‑specific weakest point for failure in each platform, as analyzed in Supplementary Section 12. See Supplementary Section 13, for the measured *S*-parameters responses of these two transducers, highlighting a minimal device-to-device variation and no degradation due to boundary-engineered energy confinement. Third, their impedance mismatch with 50 Ω systems demands precise driving frequency selection. See Supplementary Section 14 for detailed power dissipation distribution curves within a representative LAW transducer, showing their dependence on driving frequencies and measurement configurations. Most importantly, it must be considered that port impedance mismatch can be a significant concern when performing power tests at the individual transducer level, as it leads to reflected power that does not contribute to device stress. To ensure that the reported power metrics accurately represent the stress experienced by the device, all quoted power values, including the critical “injected power” in the following part, are rigorously defined as the delivered power, i.e., the incident power minus the reflected power. This definition explicitly excludes the reflected portion, thereby eliminating any bias introduced by impedance mismatch and ensuring a fair comparison of the intrinsic power-handling capability of each architecture. For the testing frequency selection, the preliminary criteria are based on the fact that the higher the dissipated power, the higher the temperature in the active region, and the shorter the time-to-failure. A detailed experimental validation to support this claim is presented in Supplementary Sections 12 and 15. Thus, driving frequencies favoring maximum power absorption for transducers should be obtained from the *S*-parameters measured under small-signal operational conditions. Last, the power absorption peak shifts downward by several megahertz under elevated power due to the non-zero TCF. To compensate, a predefined frequency window was used to mitigate thermal shifts in the power absorption peak, replacing single-frequency continuous-wave testing to maintain accuracy. Guided by these principles, standardized reliability measurements were performed to study acoustomigration dynamics and quantify the threshold power durability of each architecture.

**Fig. 5:**
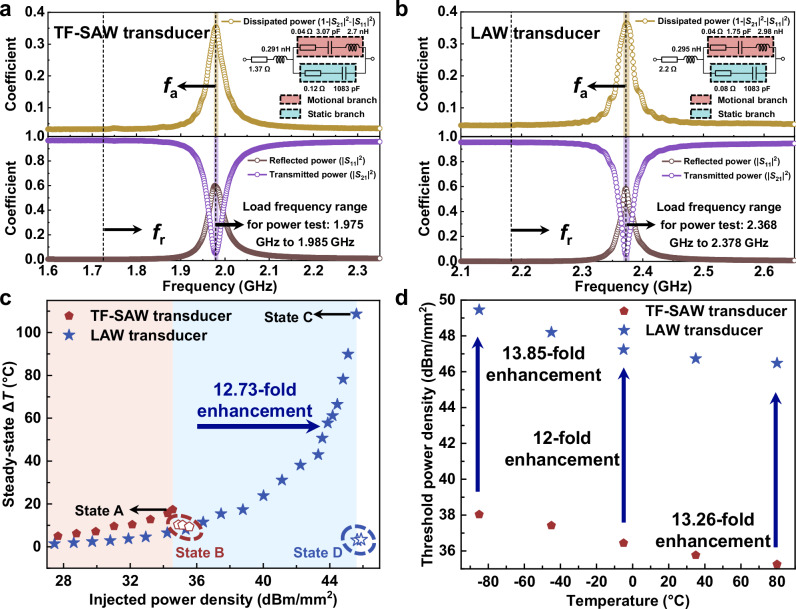
Power test design and measurement for TF-SAW and LAW transducers. **a** Measured dissipated, reflected, and transmitted power coefficients as a function of driving frequency for a 2-port TF-SAW transducer operating at a load power of *P* = −15 dBm. **b** Measured dissipated, reflected, and transmitted power coefficients as a function of driving frequencies for a 2-port LAW transducer operating at a load power of *P* = −15 dBm. Both transducers share an identical layout design to ensure equitable high-power comparison. *f*_*r*_ and *f*_*a*_ are marked by the dashed lines in each plot. The loading frequency ranges for the two transducers are also indicated. Fitted parameters from the modified Butterworth-Van Dyke (mBVD) model for each configuration are summarized in the respective insets. **c** Steady-state temperature rises extracted from thermal mapping data of both transducers are plotted against the injected power density within the specified load frequency range. Injected power density is defined as the actual power delivered to the DUT (incident power minus reflected power) divided by the whole rectangular transduction area, including the IDT area, reflectors, and bus lines. States A to D denote operating conditions of the transducers under progressively increasing power load levels. **d** Comparisons of injected power density threshold for LAW and TF-SAW transducers are presented across testing temperatures ranging from −85 to 80 °C, where the threshold is defined as the maximum power density supporting reliable operation for 5-min power loading under the specified conditions. Prior to reaching the power density thresholds, both DUTs withstood sequential tests at incrementally higher power levels.

Figure 5a, b present the frequency-dependent dissipation, reflection, and transmission coefficients of both TF-SAW and LAW transducers at −15 dBm load power. While nonlinear distortions emerge at elevated powers, their effect on overall absorption efficiency in the target frequency window remains marginal and is assumed equivalent between device types^57^. Outside the defined frequency window, most transmitted power passes through the two-port configuration with minimal energy loss. Since electromigration and acoustomigration are thermally facilitated, in situ monitoring of active region temperature is critical for understanding the failure modes. For conventional contact-method surface temperature measurements, a light-absorbing black paint, infrared (IR)-opaque and highly emissive, is typically coated on the device under test (DUT) surface to ensure uniform emissivity and consistent thermal boundary conditions. However, applying this IR-opaque coating would interfere with the operation of TF-SAW transducers. Therefore, a non-contact method, with a pre-calibrated IR camera and macro lens, was utilized for real-time temperature mapping of acoustic wave transducers without coating the IR opaque black paint. The emissivity correction protocol for quantitative extraction of temperature rise in both architectures is presented in the “Methods” section.

Due to the low reverse transmission in the power amplifier and isolator, direct measurement of admittance responses during high-power testing was not possible. However, operational conditions can be continuously tracked via in-situ IR temperature imaging and *S*_21_ scattering parameter responses. While the metal migration process in TF-SAW transducers exhibits temporal continuity, manifested by a gradual progression of voids and hillocks, both device types exhibit rapid drops in *S*_21_ and temperature upon reaching their threshold injected power. A detailed explanation of the correction method for the scattering parameters and reflection coefficients calculation is provided in the “Methods” section. Figure 5c summarizes the extracted temperature rises of TF-SAW and LAW transducers under varying injected power levels. The injected power threshold is defined as the maximum power level that DUTs can withstand for 10 min without an *S*_21_ drop. For a single TF-SAW transducer, the measured temperature experiences a nonlinear rise as the injected power increases from 15.75 dBm (37 mW) to 22.65 dBm (184 mW), driven by escalating electrical/mechanical losses and increased power dissipation coefficients. A sudden temperature drop occurs at an injected power of 22.99 dBm (199 mW). After this point, increasing the input power further results in a relatively constant temperature, indicating consistent dissipated power despite higher output power from the isolator. Given the relatively large area (0.0644 mm^2^) of the active area, this corresponds to a threshold power density of 34.56 dBm/mm^2^. For the LAW configuration, the measured surface temperature rises in the injected power range below 22.65 dBm are lower than those of the TF-SAW counterparts. The maximum temperature rise is only 5.2 °C, while the TF-SAW transducer exhibits a temperature rise of 17.4 °C, corresponding to a 70% reduction. This improvement is attributed to the extra heat dissipation routes provided by the top quasi-infinite layer. The single LAW transducer sustains stable operation up to an injected power of 33.70 dBm (2.34 W), corresponding to a high power density threshold of 45.61 dBm/mm^2^. As detailed in Supplementary Section 12, we performed comprehensive high-power characterizations on TF-SAW and LAW devices with varying *C*_0_ values at multiple frequency points across the resonator band. The results consistently demonstrate that the proposed LAW architecture achieves approximately an order of magnitude improvement in power handling capability across the entire operating band, not merely at a single frequency point. Supplementary Section 16 provides further examples of in-situ temperature measurement and analysis for LAW transducers driven by various frequencies with high power loads.

Temperature-dependent robustness was further investigated using a cryogenic probe station, systematically evaluating devices from −85 to 80 °C (Fig. 5d). To avoid the destructive impact from a single test, multiple devices of each type, matched for power dissipation profiles (selected using the criteria in Fig. 5a, b), were evaluated across the temperature range. A consistent increase in threshold injected power density is observed in the LAW transducers as ambient temperatures decrease, with a nonlinear enhancement becoming pronounced below −45 °C. The LAW transducer achieves an injected power density threshold of 49.45 dBm/mm^2^ (88.11 W/mm^2^) at −85 °C, demonstrating unprecedented high-power resilience in low-temperature environments—a 13.85-fold improvement over TF-SAW counterparts. This enhancement is attributed to accelerated thermal dissipation and reduced energy loss in LAW platforms under cryogenic conditions. Above −45 °C, LAW devices exhibit a gradual decline in threshold power density from 48.20 dBm/mm^2^ at −45 °C to 46.48 dBm/mm^2^ at 80 °C. In contrast, TF-SAW devices exhibit a sharper reduction in power capacity, dropping from 37.42 to 35.26 dBm/mm^2^ over the same temperature range. The narrower variation of power threshold in LAW transducers across the entire temperature range reflects that their power durability is less prone to degradation under thermal stress, demonstrating a superior robust, and temperature-stable performance compared to TF-SAW devices. See Supplementary Sections 17 and 18, for detailed performance comparison of the transducers tested before and after the high-power loads.

**Fig. 6:**
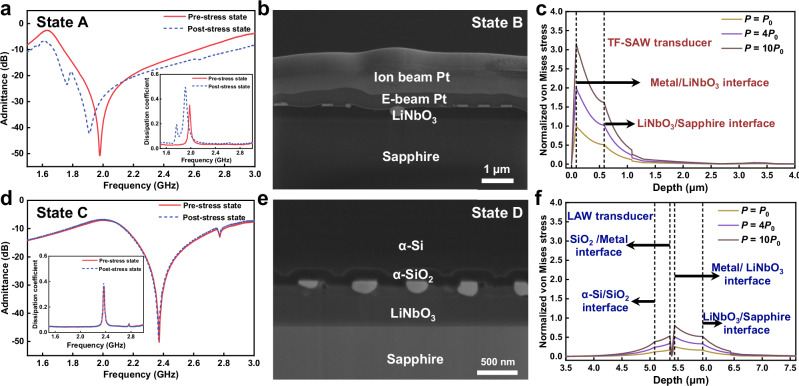
Electrical, structural, and mechanistic analysis of TF-SAW and LAW transducers. **a** Measured admittance curve and power dissipation characteristics of the TF-SAW transducer at state A, showing spurious mode, degraded *k*_*t*_^2^, and *Q*. **b** Cross-sectional view SEM image of the failed TF-SAW transducer at state B, revealing severe electrode migration (acoustomigration) and material deformation. **c** Depth-resolved von Mises stress profiles for the TF-SAW transducer under increasing injected power (*P*_0_, 4*P*_0_, 10*P*_0_), all normalized to the maximum stress in the TF-SAW device at *P*_0_. **d** Measured admittance curve and power dissipation profiles of the LAW transducer at state C, exhibiting preserved frequency response. **e** Cross-sectional view SEM image of the failed LAW transducer at state D, showing localized damage without catastrophic electrode migration. **f** Corresponding von Mises stress profiles for the LAW device under the same power levels (*P*_0_, 4*P*_0_, 10*P*_0_), normalized to the TF-SAW-at-*P*_0_ reference. The peak stress in the LAW transducer is only ~1/4 of the baseline TF-SAW’s value at *P*_0_ through stress redistribution via upper-boundary engineering—a mechanism not addressed by conventional substrate engineering.

To elucidate post-stress failure mechanical and electrical conditions, two states were defined for each transducer configuration: pre- and post-threshold power exposure (Fig. 5c, States A and C for TF-SAW and LAW devices, respectively), followed by further testing above threshold (States B and D). After 10 min at threshold power, TF-SAW devices showed reduced *k*_*t*_^2^, an in-band spurious mode, degraded *Q*, and smaller static capacitance (Fig. 6a). The inset of Fig. 6a reveals a downward shift in the dissipation coefficient, corroborating temperature measurements in Fig. 5c. Cross-sectional SEM and EDS mapping of failed TF-SAW transducers (State B, Fig. 6b) revealed severe deformation, gold migration, and hillock formation, confirming acoustomigration-induced failure. A bright line is visible along the LiNbO_3_ surface between failed electrodes (see Supplementary Section 19, for comprehensive acoustomigration investigation and EDS mapping). The EDS analysis shows the bright line is comprised of Pt and Au, indicating that acoustic-induced migration under high-power conditions has caused gold to accumulate in these regions. Besides, the EDS mapping results confirm the severely deformed LiNbO_3_ layer and IDTs under high-power loads.

In contrast, the LAW transducer in State C maintained electrical and mechanical properties nearly identical to their pre-stress state (Fig. 6d), even after withstanding over 12 times higher power density. Cross-sectional SEM of stressed LAW devices (State D, Fig. 6e) shows no evidence of electrode migration, due to the suppressed grain boundary continuity provided by the upper *α*-Si cladding. However, the deformed electrodes are diffused into the LiNbO_3_ layer, and an electrically short connection between two metal strips is formed in the left region highlighted by a rectangular frame (see Supplementary Section 20, for exhaustive EDS mapping and discussion on the cross-section). Some voids between electrodes and the SiO_2_ isolation layer were observed, attributed to mechanical stress under extreme loading, but without catastrophic device failure.

The fundamental mechanism behind this dramatic improvement in power durability is revealed by the von Mises stress distribution at the critical interface. As shown in the depth-resolved profiles (Fig. 6c, f), normalized to the maximum stress in the baseline TF-SAW device at a reference power *P*_0_, the maximum stress in the LAW platform is only about 1/4 of that in the SAW device under the same injected power (*P*_0_). Even as the power increases to 10*P*_0_, the relative stress level in the LAW device rises only marginally to approximately 4/5 of the baseline TF-SAW’s stress at *P*_0_. This substantial and persistent stress suppression demonstrates that the LAW architecture enhances power capacity primarily through the redistribution and mitigation of mechanical stress at the fundamental level, thereby directly inhibiting the driving force for acoustomigration. This result underscores a key advantage of upper-boundary engineering over conventional substrate engineering. As reported by previous work, merely improving substrate thermal conductivity does not effectively reshape this interfacial stress distribution; the failure bottleneck remains^58^. In contrast, the co-designed top cladding in the LAW platform simultaneously manages heat and actively tailors the acoustic boundary condition, achieving a fundamental improvement in power handling that overcomes the inherent bottlenecks of traditional approaches.

## Discussion

We have presented new findings in acoustics and proposed a new layered acoustic wave architecture that fundamentally redefines the electrical, mechanical, and thermal boundary conditions of high-frequency acoustic transducers for vibrating at large power density. Through the synergistic integration of a piezoelectric cavity and a quasi-infinite multifunctional cladding, the LAW platform addresses three persistent challenges in high-power acoustic wave vibration with reduced loss: acoustomigration suppression, efficient heat dissipation, and enhanced thermal stability. Our experimental results demonstrate that LAW transducers achieve a 70% reduction in temperature rise and a 12.7-fold increase in threshold power density compared to state-of-the-art TF-SAW devices, while maintaining robust frequency stability with a first-order TCF of −13 ppm/°C across a wide operational temperature range of 475 °C. Notably, the architecture enables reliable GHz acoustics at ultra-high power densities. By decoupling the constraints imposed by conventional acoustic transducer architectures, this work establishes a generalizable design paradigm for next-generation acoustic technologies. The principles demonstrated here are broadly applicable to a wide range of IDT-based acoustics systems and open pathways for the scalable deployment of ultracompact, high-power acoustic transducers in emerging applications—from 5 G/6 G communications to miniaturized, non-magnetic energy reservoirs.

## Methods

### Device fabrication

The devices were fabricated on a 500 nm-thick X-cut single-crystalline LiNbO_3_ thin film bonded to a *c*-axis sapphire substrate using surface-activated bonding and grinding technology. IDTs were first patterned by electron-beam lithography, followed by deposition of a 5 nm Cr adhesion layer and 80 nm Au via electron-beam evaporation and lift-off (metal ratio = 0.5). After that, photolithography and an additional 100 nm Au layer enhanced electrical contact and transversal energy confinement. IDTs exhibited uniform sidewall profiles. To avoid accumulated thermal stress and prevent surface damage, a 270 nm SiO_2_ layer was deposited by PECVD at a low deposition rate, leveraging its low thermal budget. After that, a 3.76 µm-thick *α*-Si layer was deposited by PECVD. Residual stress introduced during the *α*-Si deposition process leads to buckling, delamination, fatigue, and other failure modes in the thick film. Large residual stress also facilitates device failure and thin-film cracking under high-power operation, degrading device performance by altering the mechanical properties of the LAW transducer^59^. Intrinsic stress in *α*-Si arises from systematic changes in the position of Si atoms that happen after a slip-free adhesion layer on the underlying substrate. These atomic arrangements can be controlled by adjusting the deposition conditions and processing time. To mitigate accumulated stress, the deposition process was divided into 18 cycles, each lasting 5 min. This strategy prevents the combination of both intrinsic stress and thermal stress, which could otherwise lead to the formation of cracks and hillocks in thick films. The residual stress in the *α*-Si thick film can be extracted via curvature measurement on a 4-inch standardized wafer. Detailed extraction method and influence of residual stress in the *α*-Si thick film on LAW device performance are discussed in Supplementary Section 21. Note that the optimized residual stress in *α*-Si thick film is 4.38 MPa. In the following steps, photoresist (HPR504) was employed as an etching mask, followed by *α*-Si etching using SF_6_/Ar hybrid gas in an inductively coupled plasma reactive ion etching (ICP-RIE) system. Subsequently, SiO_2_ was etched using CHF_3_/O_2_ hybrid gas in a reactive ion etching (RIE) system. The etching procedures were controlled by pre-calibrated etching time and depth, and the etching depth was examined via profilometry. Then, the etching mask was stripped in acetone, followed by a standard cleaning process.

### Electrical characterization

Following off-wafer short-load-open calibration, one-port measurement configuration is utilized to measure the scattering parameters of transducers under room temperature and atmospheric pressure, using a Keysight P5028A Vector Network Analyzer with a load power of −15 dBm. The admittance responses (*Y*-parameters) were subsequently calculated from the measured *S*-parameters without applying any extra de-embedding step. The performance merits of each device are evaluated by extracting the Bode-*Q* using established methods^60^. While *k*_*t*_^2^ exhibits significant variation depending on the calculation formula, making it less meaningful when compared with prior works. Here, *k*_*t*_^2^ in this work is calculated using the standard IEEE definition^61^: ${k}_{t}^{2}=\frac{{\rm{\pi }}\left({f}_{r}/2{f}_{a}\right)}{\tan \left[\pi {f}_{r}/\left(2{f}_{a}\right)\right]}$. Temperature-dependent frequency drift was characterized in a vacuum cryogenic probe station across a range of −150 to 325 °C, with 20 °C increments. To mitigate thermal instability from the measurement setup, the wafer was stabilized for 20 min at each target temperature prior to probe contact, followed by an additional 5-min settling period post-landing to ensure thermal equilibrium at the probe tips.

### Power durability measurement

The power handling capability of acoustic devices is conventionally evaluated on the basis of the time to failure (TTF) metric, which correlates closely with not only the input power (${P}_{{\rm{in}}}$), the device temperature (*T*), but also the driving frequency. TTF follows Eyring’s model: $\tau=\alpha \cdot \exp \left(E/{kT}\right)\cdot {P}_{{\rm{in}}}^{m}$, where *k* and *E* represent the Boltzmann constant and activation energy, and *α*, *m* are device-specific constants. In this model, excessive power and high temperature are typically applied to accelerate device failure to a realistic value due to the Arrhenius law. While accelerated failure tests for acoustic transducers under elevated power and temperature (enabled by the Arrhenius law) allow TTF prediction via numerical models built on massive experimental data, no standardized protocol exists for acoustic transducers.

To address this gap, we established a benchmarking method for power capacity by directly monitoring temperature and *S*_21_ variations in TF-SAW and LAW transducers. Device failure is identified by an abrupt temperature drop and *S*_21_ degradation, which indicates impedance mismatch preventing power delivery to the DUTs. We incrementally increased the isolator’s output power from 18 dBm in 1 dB steps until reaching 30 dBm, followed by 0.5 dB steps up to 33 dBm, and finally 0.25 dB steps until reaching the injected power threshold, at which point device failure occurred within a 5-min loading time. For each frequency sweep, the time of absorption is 10 s, sufficient for the device to reach thermal equilibrium at each power level^62^. Besides, a port-extension calibration was performed at each power level using a CS-5 substrate’s “Thru” standard. The *S*_21_ response measured directly at 10 dBm input power after necessary off-wafer calibration is plotted in Supplementary Section 22, and aligns with the de-embedded *S*_21_ response of the DUT by using the Thru standard as a reference. Reflection coefficients of transducers under varying power loads were then extracted via the de-embedded *S*_21_ transmission response modelling.

Prior to in-situ temperature distribution measurements of the DUT during power load experiments, an emissivity correction is required for quantitative thermal mapping. While emissivity calibration is typically performed per constituent layer, the heterogeneous geometry with nonuniformity within the active region complicates this process. To address this, TF-SAW and LAW transducers were uniformly heated to predefined temperatures (50–130 °C, 20 °C increments) on a controlled hotplate. The surface emissivity was then calibrated by correlating IR camera radiation data with known temperatures. At each increment, samples were stabilized for 30 min and measurements were averaged over five devices to minimize uncertainty.

### Nano/microstructure analysis

Cross-section samples of the broken TF-SAW transducer, as well as pristine and damaged LAW transducers, were prepared for TEM using an FEI Helios G4 UX Dual-Beam focused-ion-beam/SEM system. Surface and cross-sectional morphologies were examined with a JEOL-7800F SEM. High-resolution TEM (HR-TEM) and high-angle annular dark-field scanning TEM (HAADF-STEM) images were obtained using a JEOL JEM-ARM200F STEM equipped with a Cs probe corrector. This instrument, featuring dual wide-area (100 mm^2^) silicon drift detectors, was also used for energy-dispersive X-ray spectroscopy (EDS) elemental mapping to assess compositional uniformity and interfacial quality across the device stack.

## Supplementary information

Supplementary information

Transparent Peer Review file

## Source data

Source data

## Data Availability

All data supporting the findings of this study are available within the article and its supplementary files. Any additional requests for information can be directed to and will be fulfilled by the corresponding authors. Source data are provided in this paper. Source data are provided with this paper.
