# Supplementary material for: Suppressing acoustomigration and temperature rise for high-power robust acoustics
Source: Nat Commun. 2026 Apr 22;17:5543. doi: 10.1038/s41467-026-72102-7 (PMC13287746; doi:10.1038/s41467-026-72102-7)
Supplement: Supplementary file 1 — Supplementary information [file 41467_2026_72102_MOESM1_ESM.pdf]

Supplementary Information for “Suppressing Acoustomigration and Temperature Rise for High-Power Robust Acoustics”

Fangsheng Qian[1], Shuhan Chen[1], Wei Wei[1], Jiashuai Xu[1], Kai Yang[1], Junyan Zheng[1], Zijun Ren[1], Xingyu Liu[1], and Yansong Yang[1*]

[1]Department of Electronic and Computer Engineering, The Hong Kong University of Science and Technology, Hong Kong, China.

*Correspondence and requests for materials should be addressed to: * Yansong Yang: eeyyang@ust.hk

Table of contents

1 Boundary analysis for maximizing acoustic wave confinement..3

2 Energy confinement comparison of LAW transducers with different cladding layers......6

3 Measured TCF results of TF-SAW and SiO_2-overcoated TF-SAW devices......................8

4 Velocity saturation effect on the LAW platform...10

5 EDS mapping of the as-fabricated LAW transducer...11

6 Spurious modes suppression strategies for LAW transducers 12

7 Measurement results obtained from the baseline TF-SAW transducers 16

8 Performance comparison between TF-SAW, SiO_2-overcoated TF-SAW, and LAW transducers 17

9 Advantages of LAW transducer in terms of temperature compensation effect 18

10 Power durability measurement setup 19

11 Power handling capability evaluation at filter-level 20

12 Frequency- and C_0-dependent von Mises stress profiles 24

13 Measured S-parameter responses for TF-SAW and LAW transducers 29

14 Dissipated power distribution of a typical LAW transducer vs. frequency 30

15 High-power test for SAW transducer at f_r...32

16 Thermal analysis of a LAW transducer under high RF loads......................................35

17 Performance comparisons of TF-SAW transducers before and after power tests 37

18 Performance comparisons of LAW transducers before and after power tests 38

19 Acoustomigration investigation on broken TF-SAW transducers (State B)................39

20 EDS mapping of a failed LAW transducer (State D)...41

21 Influence of residual stress of the silicon cladding layer on device performance 42

22 Calculation method of reflection coefficients for high-power tests.............................45

References...47

1 Boundary analysis for maximizing acoustic wave confinement

We assume a three-layer heterostructure in which an isotropic and homogeneous electrical insulating SiO_2 layer with thickness h is inserted in between the $LiNbO_3$ and the top cladding layers. The initial boundary engineering of the proposed LAW configuration aims at maximizing the acoustic wave confinement. The LAW velocity in the proposed configuration can be calculated starting from Equations (S1) and (S2)[1]:

$$\Omega(V_{\mathrm{LAW}}) + \Delta(V_{\mathrm{LAW}}) = K^2, \tag{S1}$$

$$\Delta(V) = \frac{\rho'}{\rho}\left(\frac{V_B'}{V_B}\right)^2 \Omega'(V), \tag{S2}$$

where $\Omega(V) = \sqrt{1-(V/V_B)^2}$ and $\Omega'(V) = \sqrt{1-(V/V_B')^2}$. K^2 refers to the electromechanical coupling coefficient for thickness-shear vibration and V_B represents the SH-mode velocity of the $LiNbO_3$ layer. For the specific three-layer structure, $\Delta(V)$ can be derived from the recursive relation of acoustic impedance matrixes[2], this yields:

$$\Delta(V) = \frac{\rho''}{\rho}\left(\frac{V_B''}{V_B}\right)^2 \Omega''(V) \frac{1+\frac{\rho''\Omega''(V)}{\rho'\Omega'(V)}\left(\frac{V_B''}{V_B'}\right)^2 \tanh\big(\beta h \Omega''(V)\big)}{\frac{\rho''\Omega''(V)}{\rho'\Omega'(V)}\left(\frac{V_B''}{V_B'}\right)^2 + \tanh\big(\beta h \Omega''(V)\big)}, \tag{S3}$$

where $\Omega''(V) = \sqrt{1-(V/V_B'')^2}$, and β is denoted as the wavenumber of LAW along the boundary between the sandwiched SiO_2 layer and the $LiNbO_3$ layer. Generally, the LAW velocity V_{LAW} should be slower than that of slow shear bulk waves in three layers. Otherwise, the LAW exhibits a leaky nature with degraded performance. When $V_B'' < V < V_B'$, it should be noted that $\Omega'(V)$ and $\Omega''(V)$ are purely real and purely imaginary, respectively. Under this circumstance, $\Omega''(V)$ can be rewritten as: $\Omega''(V) = \mathrm{i}\zeta$, $\zeta = \mathrm{Im}[\Omega''(V)]$, $\zeta = (V/V_B'')^2 - 1$. Rearrange Equation (S3) by using the relationship of $\tanh(\mathrm{i}x) = \mathrm{i}\tan(x)$, we get

$$\Delta(V) = \frac{\rho''}{\rho}\left(\frac{V_B''}{V_B}\right)^2 \zeta \frac{1-\frac{\rho''\zeta}{\rho'\Omega'(V)}\left(\frac{V_B''}{V_B'}\right)^2 \tan(\beta h \zeta)}{\frac{\rho''\zeta}{\rho'\Omega'(V)}\left(\frac{V_B''}{V_B'}\right)^2 + \tan(\beta h \zeta)}. \tag{S4}$$

From the perspective of material designs, it is recommended that materials for the upper and sandwiched layers should feature large $\rho'' V_B''/\rho' V_B'$ to achieve a large $-\Delta(V)$, giving rise to an increased coupling coefficient and extended the existence range of LAW. Equation (S4) implies that $\Delta(V)$ should be negative provided that:

$$\frac{\rho''\varsigma}{\rho'\Omega'(V)}\left(\frac{V_B''}{V_B'}\right)^2\tan(\beta h\zeta) > 1. \tag{S5}$$

Given the sandwiched materials SiO_2 thanks to its good insulation properties, we can attempt to extend the range to $V_B'' < V_{\mathrm{LAW}} < V_B'$ when satisfying the condition of equation (S5). For simplicity, we define the following relationships:

$$\begin{cases} \dfrac{\rho''}{\rho} = x, \dfrac{V_B''}{V_B} = y \\ \dfrac{\rho''}{\rho'} = a, \dfrac{V_B''}{V_B'} = b \end{cases}.$$

Considering the electrode thickness 0.06λ, we have $V_B < V_{\mathrm{LAW}} < V_B'' < V_B'$, i.e., $b < 1 < y$. $\Omega(V) + \Delta(V)$ is numerically solved to show monotonical decrease with V.

For $V_{\mathrm{LAW}} < V_B''$, it exists $\Omega(V_B'') + \Delta(V_B'') = \sqrt{1 - (V_B''/V_B)^2} + 0 \le K^2$, which can be derived as

$$y \ge \sqrt{1 - K^2}. \tag{S6}$$

The relation show in Equation (S7) is naturally satisfied for $y > 1$ known from existing velocity relations.

For $V_B < V_{\mathrm{LAW}}$, it exists

$$\Omega(V_B) + \Delta(V_B) = xy^2\sqrt{1-\left(\frac{1}{y}\right)^2}\,\frac{1 + ab^2\dfrac{\sqrt{1-\left(\frac{1}{y}\right)^2}}{\sqrt{1-\left(\frac{b}{y}\right)^2}}\tanh\left(\beta h\sqrt{1-\left(\frac{1}{y}\right)^2}\right)}{ab^2\dfrac{\sqrt{1-\left(\frac{1}{y}\right)^2}}{\sqrt{1-\left(\frac{b}{y}\right)^2}}+\tanh\left(\beta h\sqrt{1-\left(\frac{1}{y}\right)^2}\right)} \ge K^2. \tag{S10}$$

From Equation (S10), the LAW can be confined within the range:

$$b \le \left(\sqrt{\frac{y^2C}{a^2} + \frac{C^2}{4a^4}} - \frac{C}{2a^2}\right)^{1/2}, \tag{S11}$$

where C is denoted as:

$$C = \frac{1}{y^2 - 1}\left((\frac{xy\sqrt{y^2 - 1} - K^2 \tanh\left(\beta h\sqrt{1 - \left(\frac{1}{y}\right)^2}\right)}{K^2 - xy\sqrt{y^2 - 1}\tanh\left(\beta h\sqrt{1 - \left(\frac{1}{y}\right)^2}\right)}) \right)^2 . \qquad \text{(S12)}$$

2 Energy confinement comparison of LAW transducers with different cladding layers

Early investigations reveal that the power dissipation peak arises not only from Joule heating but also from SAW propagation losses, including bulk wave radiation. This necessitates energy confinement as the primary criterion for evaluating LAW configuration feasibility. As discussed in Fig. 2 of the main text, the very structure of a LAW transducer inherently requires an additional elastic medium atop a conventional TF-SAW design. While shear horizontal (SH) acoustic wave confinement has been validated for the $LiNbO_3$ on sapphire platform[3], energy confinement at the $LiNbO_3$/cladding interface remains critical to evaluate. To verify it, quasi-3D unit-cell frequency-domain simulations using COMSOL Multiphysics v6.0 are performed to compare α-Si and α-SiO_2 cladding layers. **Supplementary Fig. S1a** displays the simulated admittance curves for LAW transducers with 3 μm α-Si and α-SiO_2. For α-Si cladding, as shown in **Supplementary Fig. S1a**, the targeted SH resonance mode achieves a high electromechanical coupling coefficient of 17.8% with minimal bulk wave radiation, confirming the validity of boundary engineering. Mode A, corresponding to the first mechanical resonance, exhibits strong acoustic energy confinement at the $LiNbO_3$/α-Si interface, characterized by rapid energy decay in the thickness direction. Mode B, representing the second mechanical resonance, displays quasi-Sezawa behavior by coupling the fundamental symmetrical (S0) and asymmetrical (A0) Lamb wave modes. In contrast, the α-SiO_2 cladding shown in **Supplementary Fig. S1a** introduces significant spurious responses beyond the first resonance. The main mode presents a small k_t^2 of 8.1% and a much smaller impedance ratio (40 dB) compared to that of α-Si. Mode C, associated with the first mechanical resonance, shows slow SH component decay across the amorphous silicon dioxide layer, while Modes D and E correspond to bulk shear horizontal wave radiation, indicating poor energy confinement of the LAW transducer with α-SiO_2 cladding layer. These findings agree well with our theoretical calculation for LAW transducers design, validating the methodology of boundary engineering for acoustic energy confinement.

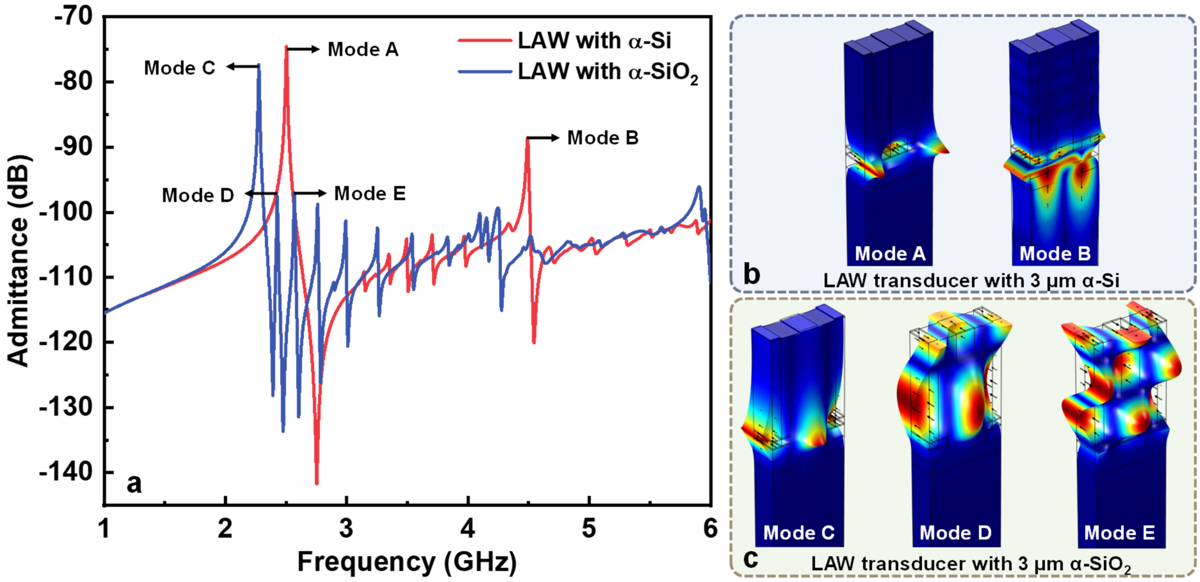

Supplementary Fig. S1 | Energy confinement comparison of LAW transducers with different thick cladding layers. a, Simulated admittance curves of LAW transducers with thick α-Si and α-SiO_2 cladding layers. **b,** Corresponding displacement mode shapes at f_r of Mode A and Mode B on the LAW transducer with 3 μm α-Si cladding layer. **c,** Corresponding displacement mode shapes at f_r of Mode C-E on the LAW transducer with 3 μm α-SiO_2 cladding layer.

3 Measured TCF results of TF-SAW and SiO_2-overcoated TF-SAW devices

As shown in **Supplementary Fig. S2a**, the measured admittance curve of an Au/$LiNbO_3$/Sapphire TF-SAW transducer (λ = 1.2 μm) exhibits a downward shift with increasing temperature, confirming a negative temperature coefficient of frequency (TCF). As the temperature decreases from 25 °C to 5 °C, the 3-dB quality factor (Q_a) at anti-resonant frequency (f_a) increases sharply from 98 to 314, marking a turnover point in dominant loss mechanisms. Additionally, spurious response near f_a also deteriorates Q_a at 25 °C. Continuously decreasing the temperature to −35 °C results in negligible Q_a variation, indicating stabilized energy loss behavior. A quadratic polynomial is utilized to fit the extracted frequency shifts at different temperatures, which shows a first-order TCF of −64.30 ppm/°C and a second-order TCF of −9.672 ppb/°C^2, as shown in **Supplementary Fig. S2b.** Compared to the uncoated TF-SAW transducer, f_r of 500 nm SiO_2-overcoated TF-SAW transducer shifts from 1.941 GHz to 2.318 GHz but exhibits degraded performance, as illustrated in **Supplementary Figs. S2c and S2d**. The admittance ratio decreases from 58 dB to 41 dB, and the k_t^2 also decreases from 28% to 11.40%. Notably, the SiO_2 coating exacerbates the TCF, increasing the first-order and second-order TCF to −161.96 ppm/°C and −622.18 ppb/°C^2, respectively.

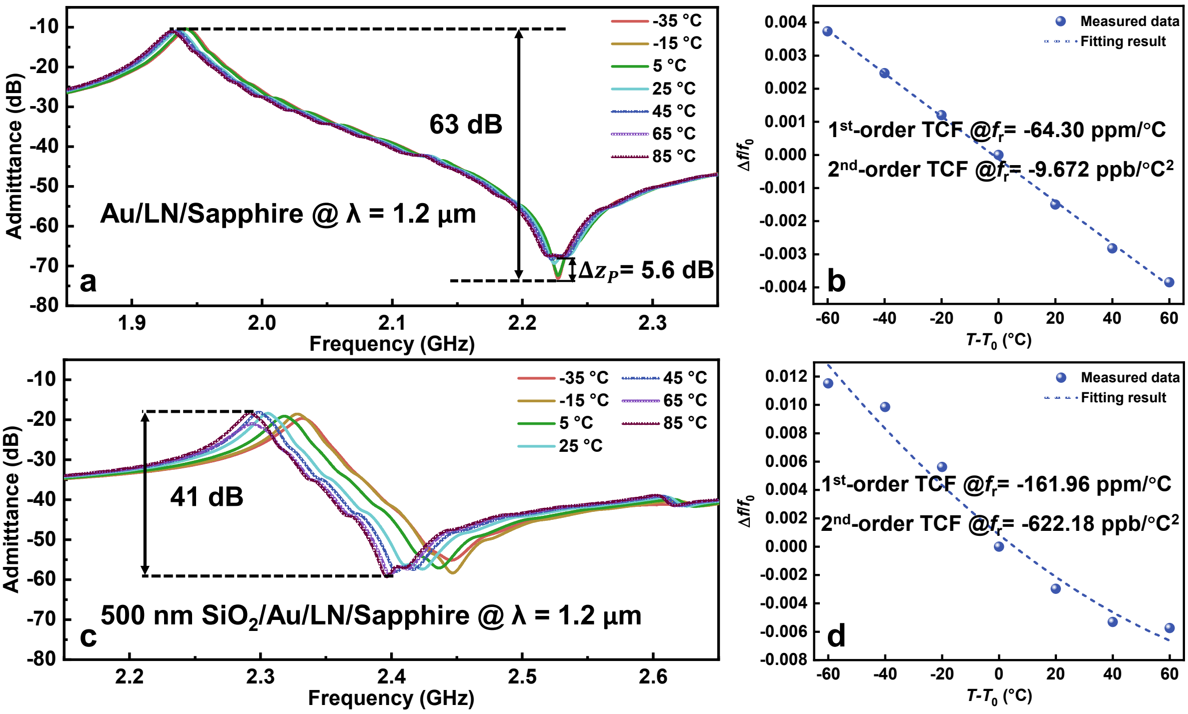

Supplementary Fig. S2 | Temperature-dependent admittance curves of TF-SAW and SiO_2-overcoated TF-SAW transducers. a, Measured admittance curves of a TF-SAW transducer (λ = 1.2 μm) across a temperature of −35 °C to 85 °C. **b,** Relative f_r shift of the TF-SAW transducer (λ = 1.2 μm) under testing temperatures ranging from −35 °C to 85 °C, with 1st and 2nd-order TCF fitting results. **c,** Measured admittance curves of a 500 nm SiO_2-overcoated TF-SAW transducer (λ = 1.2 μm) across a temperature of −35 °C to 85 °C. **d,** Relative f_r shift of a 500 nm SiO_2-overcoated TF-SAW transducer (λ = 1.2 μm) under testing temperatures ranging from −35 °C to 85 °C, with 1st and 2nd-order TCF fitting results.

4 Velocity saturation effect on the LAW platform

As shown in **Supplementary Fig. S3**, the resonant frequency of the fundamental layered SH0 mode increases with the thickness of the *α*-Si stress-manipulation layer, which modifies the effective Young's modulus and confines acoustic energy within the resonator cavity. Crucially, this frequency shift saturates at an *α*-Si thickness of approximately 0.6 µm, beyond which further increases have a negligible effect. This saturation behavior is elucidated by the simulated mode profile (inset of the figure for a 4-µm-thick *α*-Si layer), showing that the acoustic strain field is effectively confined at the IDT/$LiNbO_3$ interface and decays rapidly within the over-layer. As such, beyond the critical thickness, the stress-manipulation layer acts as a semi-infinite medium from the perspective of the guided mode, making the frequency independent of further thickness increases.

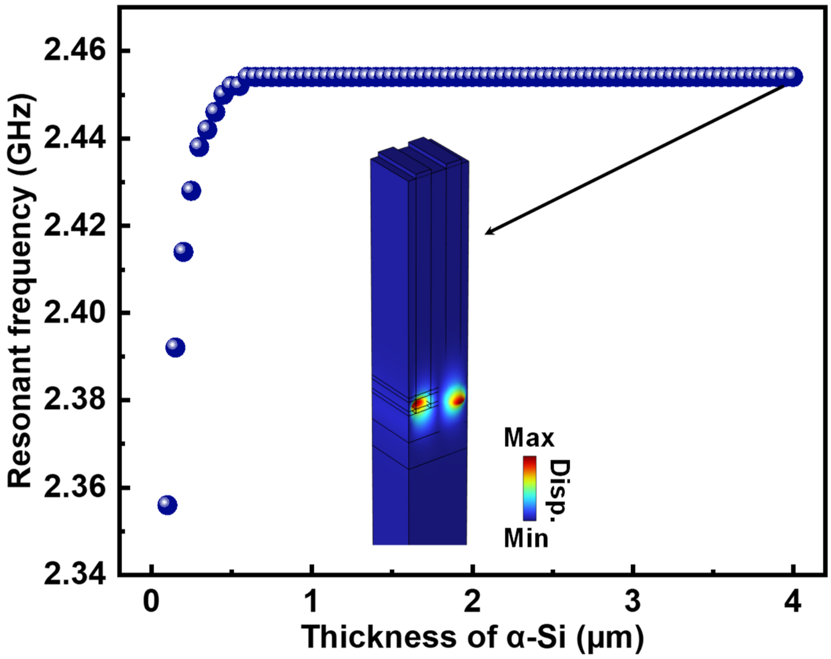

Supplementary Fig. S3 | Resonant frequency dependence on the *α*-Si top-cladding thickness and corresponding mode profile at a thickness of 4 µm.

5 EDS mapping of the as-fabricated LAW transducer

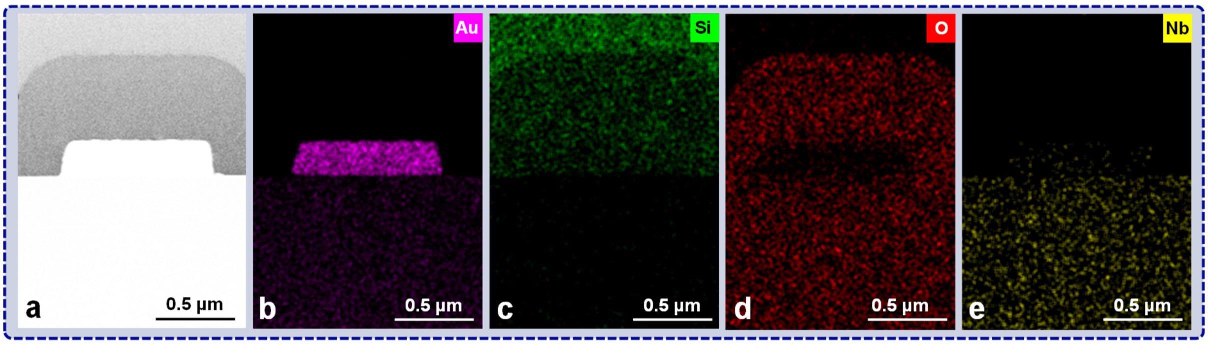

Supplementary Fig. S4 | Energy dispersive spectroscopy (EDS) mapping of the as-fabricated LAW transducer. a, High-angle annular dark-field (HAADF) imaging STEM image illustrates the zoom-up view of LAW configuration having vertical stacks of α-Si/α-SiO_2/Au/$LiNbO_3$. Corresponding elemental mapping results for **b,** Au, **c,** Si, **d,** O and **e,** Nb, respectively. The EDS characterization well demonstrates the successful fabrication of the 3D-stacked LAW architecture.

6 Spurious modes suppression strategies for LAW transducers

The spurious modes observed in Figs. 4a and 4c are identified as two distinct types: transversal modes, arising from waveguiding in the aperture direction, and higher-order bulk waves, generated by scattering at vertical acoustic boundaries. The suppression of transversal modes has been extensively investigated in piezoelectric-on-insulator (POI) platforms. Established techniques, including piston-mode designs[4], tilted electrodes[5,6], and slowness curve modulation[7], as demonstrated in prior work, can effectively flatten the lateral velocity profile and mitigate these modes.

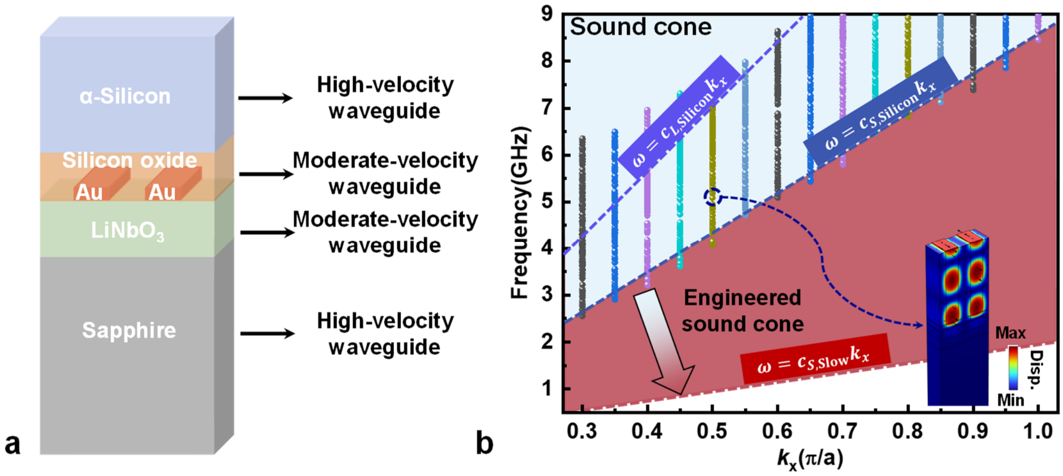

Supplementary Fig. S5 | Acoustic waveguide design for spurious-mode suppression. a, Schematic of the initial vertically stacked LAW transducer and its associated acoustic waveguides. **b**, Dispersion relation of an optimized LAW transducer design for suppressing bulk vibrational modes. The light-blue region denotes the initial sound cone for free-propagating bulk modes. The red-extended sound cone represents an engineered waveguide region with sufficiently low acoustic velocity, which redirects bulk modes away from the sandwiched resonator cavity.

To address the higher-order bulk waves, we propose and demonstrate a mechanical bandgap engineering strategy intrinsic to the layered architecture. The LAW structure can be conceptualized as a vertical stack of acoustic waveguides, as shown in **Supplementary Fig. S5a.** Given dispersion relationship for each mode, the target SH0 mode is confined within the $LiNbO_3$ layer, while parasitic bulk waves can propagate into the overlying SiO_2 and α-Si layers. To suppress those unwanted spurious waves, we engineer the sound cone in **k**-space to guide them into an additional, uppermost slow-wave layer (**Supplementary Fig. S5b**). In this

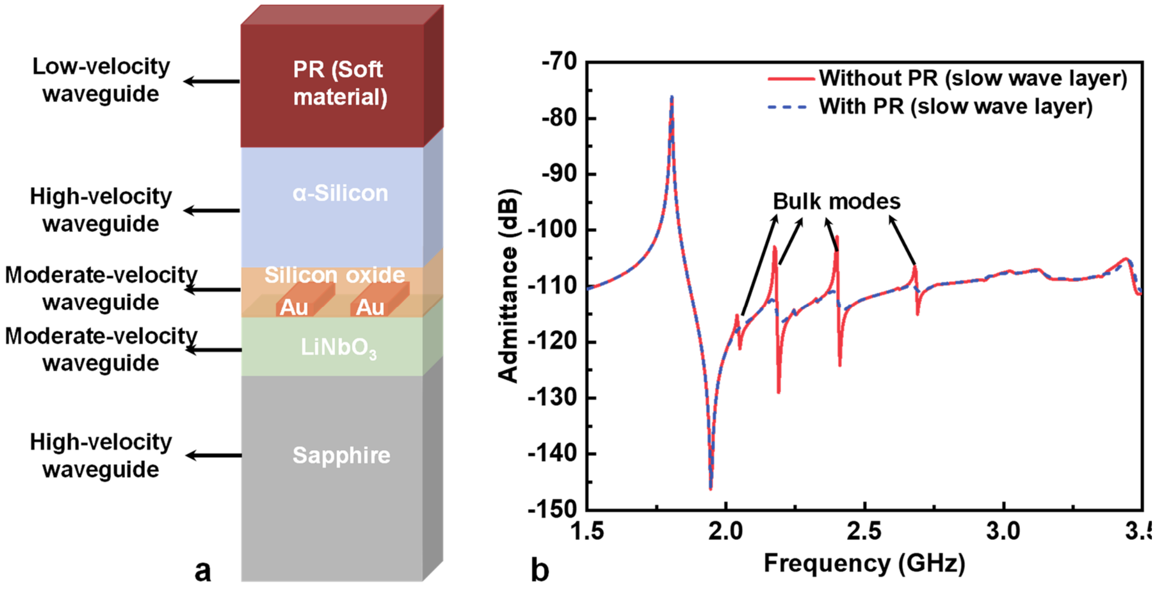

Supplementary Fig. S6 | Simulated verification of bulk-mode suppression via a slow-wave photoresist (PR) waveguide. a, Schematic of the engineered LAW transducer conceptualized as a vertical stack of distinct acoustic waveguides. **b**, Simulated admittance curves for the LAW transducer design with and without a slow-wave PR medium.

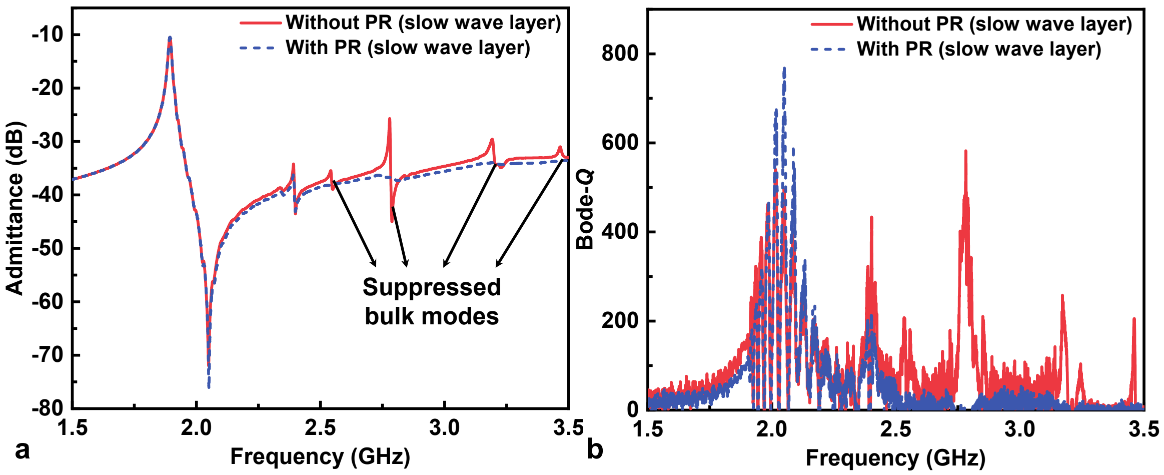

Supplementary Fig. S7 | Experimental verification of spurious bulk-mode suppression. a, Measured admittance and **b** extracted Bode-Q characteristics from one-port measurements, comparing the LAW transducer with (red) and without (blue) the slow-wave PR waveguide. The engineered mechanical boundary suppresses bulk modes, increasing the maximum Bode-Q of the targeted mode from 541 to 776.

engineered sound cone, initial bulk modes vibrating in the upper SiO_2 and α-Si waveguides propagate into a slower wave layer, which sits on the top of the LAW structure (**Supplementary Fig. S6a**).

Here, a photoresist (PR) layer serves as this slow-wave medium. Finite-element simulations confirm this principle: adding a PR waveguide significantly suppresses bulk-wave

spurs without degrading the primary SH0 mode's electromechanical coupling (k_t^2) or quality factor (**Supplementary Fig. S6b**). We experimentally validated this by coating a 5.3-μm-thick PR layer on a LAW transducer (with probing pads exposed for electrical contact). The measured admittance (**Supplementary Fig. S7a**) shows effective bulk-mode suppression. the maximum Bode-Q of the target mode increased from 541 to 776, while spurious modes were reduced to negligible levels (**Supplementary Fig. S7b**).

Wavelength-dependent measurements (**Supplementary Fig. S8**) further confirm the robustness of this mechanical boundary condition design. Key metrics, including admittance ratio (AR), Bode-Q_{max}, k_t^2, and the figure of merit (FoM = Bode-$Q_{\mathrm{max}} \times k_t^2$), are summarized in **Supplementary Fig. S9.** The engineered LAW transducer with a slow-wave overlay shows significant performance enhancement over both traditional SAW and baseline LAW devices. Additionally, this suppression strategy does not compromise power-handling capability: the maximum von Mises stress at the critical metal/$LiNbO_3$ interface remains unchanged, confirming that the thermal-mechanical integrity of the core resonator is preserved.

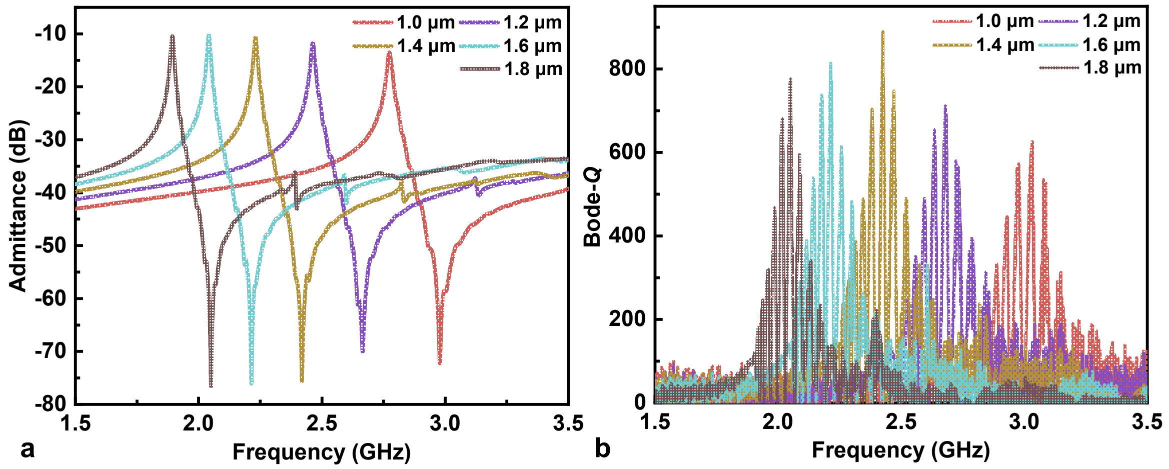

Supplementary Fig. S8 | Wavelength-dependent performance of engineered LAW transducers with the slow-wave PR waveguide. a, Measured admittance curves and **b,** extracted Bode-Q characteristics across different wavelengths, demonstrating the consistent performance enhancement benefiting from the spurious-mode suppression strategy.

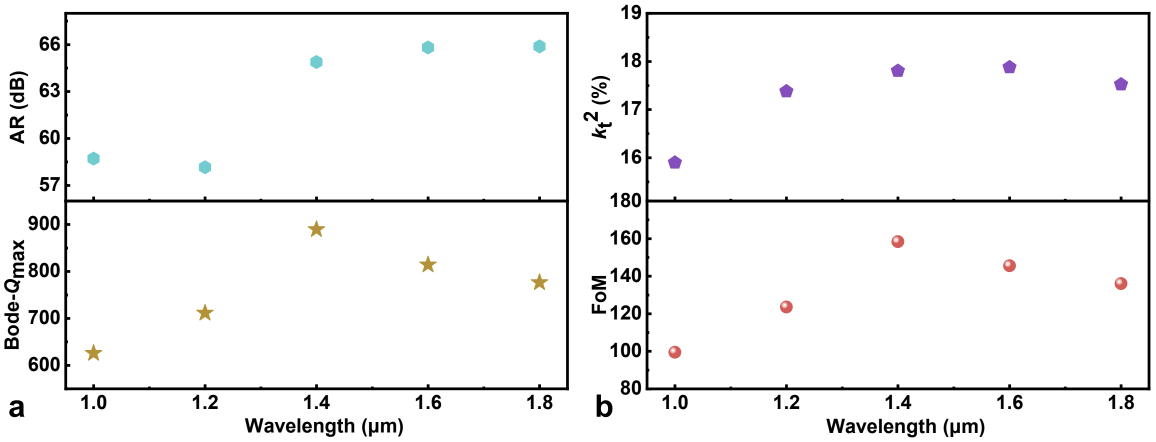

Supplementary Fig. S9 | Wavelength-dependent performance metrics of the engineered LAW transducer. Key metrics extracted for the device with the slow-wave PR layer across wavelengths, including **a,** admittance ratio (AR) and maximum Bode-Q (Bode-Q_{max}), and **b,** efficient electromechanical coupling coefficient (k_t^2) and figure of merit (FoM, defined as Bode-Q_{max} × k_t^2).

7 Measurement results obtained from the baseline TF-SAW transducers

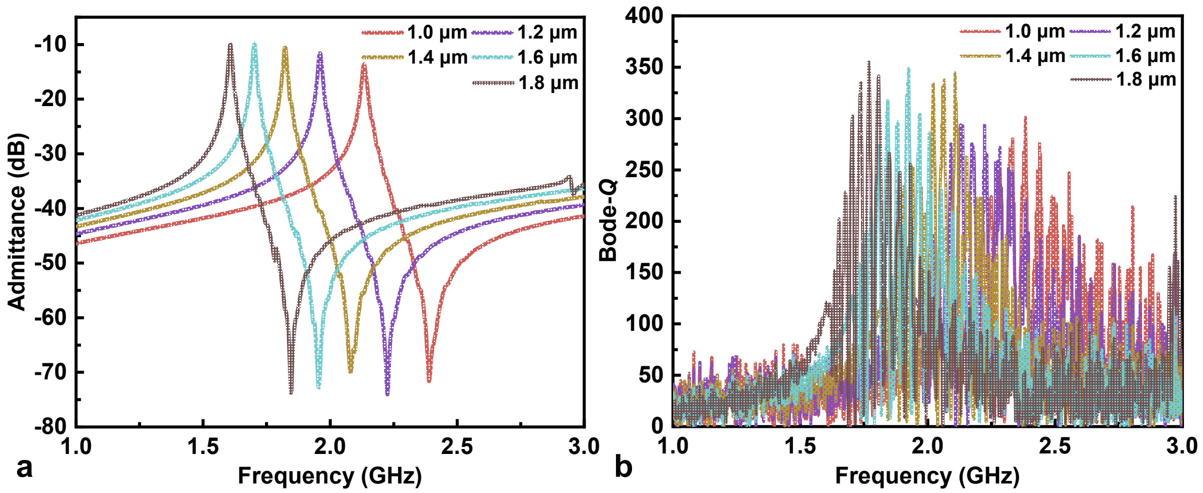

Supplementary Fig. S10 | Baseline performance of the SAW transducer. Wavelength dependence of **a**, measured admittance curves and **b**, extracted Bode-*Q* characteristics for the TF-SAW transducer prior to the deposition of any upper cladding layers.

8 Performance comparison between TF-SAW, SiO_2-overcoated TF-SAW, and LAW transducers

Supplementary Table S1. Summarized key metrics of transducers in three configurations

	**TF-SAW transducer**	**SiO_2-overcoated TF-SAW transducer**	**LAW transducer**
f_r	1.951 GHz	2.205 GHz	2.458 GHz
**AR**	56.18 dB	45.4 dB	54.1 dB
Q_r	139.4	129.7	175.4
Q_a	218.4	211.9	427.64
k_t^2	23.73%	12.47%	14.67%
**Bode Q_{max}**	305	234	445
**FoM**	79.26	34.79	73.50
1st-order **TCF_r**	−64.3 ppm/°C	−117.51 ppm/°C	−21.8 ppm/°C
1st-order **TCF_a**	−40.36 ppm/°C	−98.92 ppm/°C	−13 ppm/°C

9 Advantages of LAW transducer in terms of temperature compensation effect

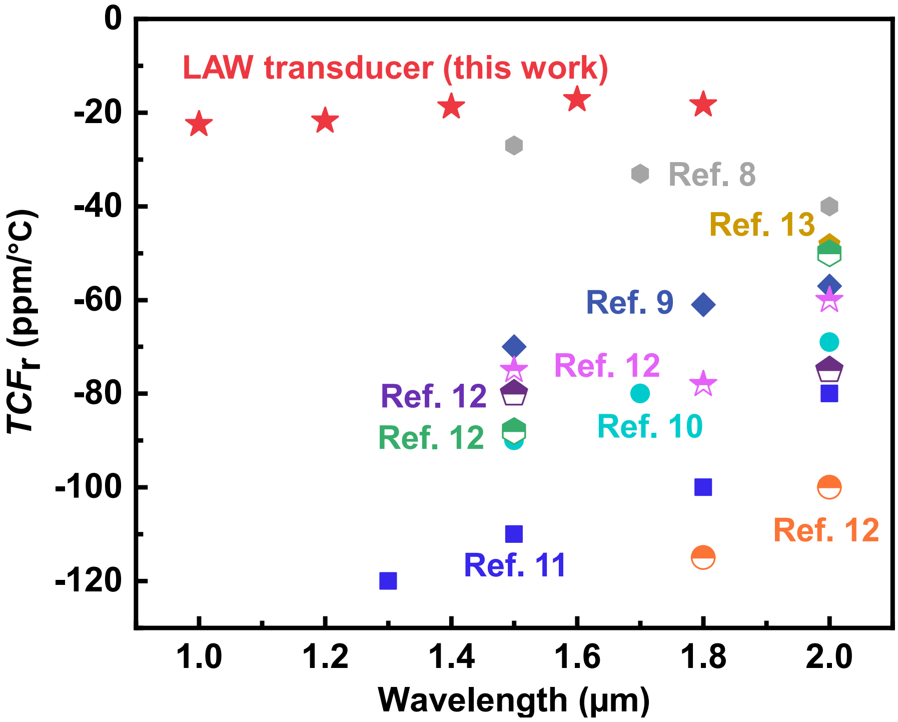

Supplementary Fig. S11 | Advantages of LAW transducers in terms of temperature compensation effect. The TCF at f_r (TCF_r) of the SH-mode LAW transducers is compared with the advanced acoustic transducers across two device configurations: (1) SiO_2-overcoated bulk $LiNbO_3$ substrates[8] and (2) $LiNbO_3$-on-insulator (LNOI) TF-SAW platforms fabricated on silicon (Si)[9,10], quartz[11,12], and silicon carbide (SiC)[13] substrates. All devices share identical geometric parameters to ensure a fair comparison.

10 Power durability measurement setup

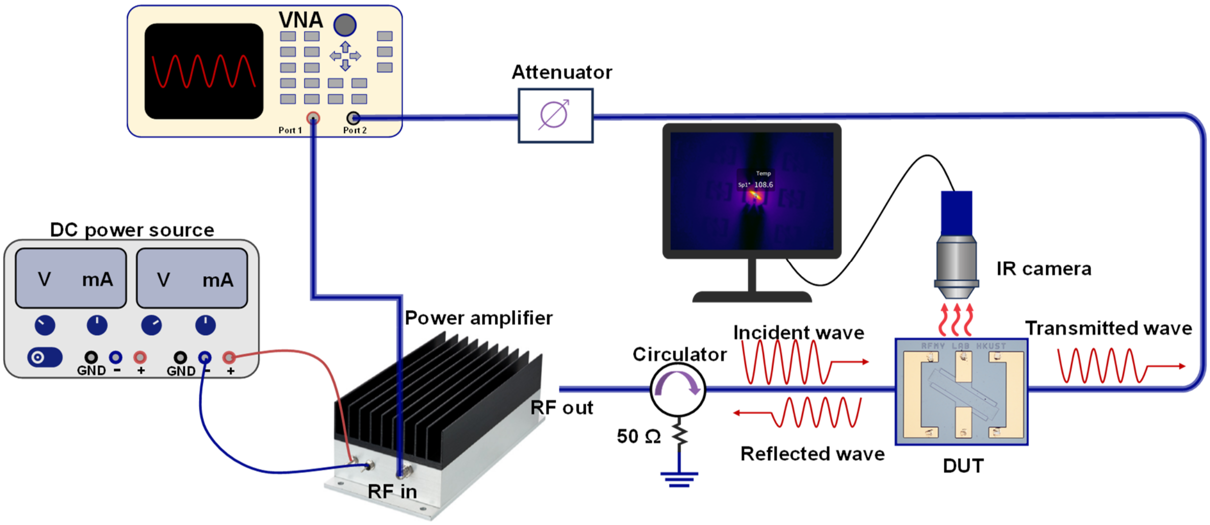

Supplementary Fig. S12 | Power durability measurement setup. Diagram of the experimental setup for the temperature mapping and the vectorial S_{21} parameters measurements of the testing transducers under high-power loads. The inset figure in DUT refers to the microscopy image of a LAW transducer with a two-port configuration. A thermal profile of the two-port LAW transducer under an injected power density load of 45.61 dBm/mm^2 is also presented, indicating a temperature rise of 108.6 °C.

11 Power handling capability evaluation at filter-level

To substantiate our claim that power handling is a system-level property confounded by non-architectural factors, we performed a controlled, high-power study at the IHP-SAW filter level using identical constituent resonators and, critically, an identical filter layout. The three tested configurations, unpackaged (forward connection), unpackaged (reverse connection), and packaged (forward connection), differ only in their electrical port connection sequence and the presence/absence of encapsulation, while sharing the exact same physical layout design. We define "forward connection" as the configuration where incident power arrives first at the filter's input terminal, and "reverse connection" as the case where it arrives first at the output terminal. **Supplementary Fig. S13** presents the S_{21} responses under increasing injected power. While all configurations remained stable up to 22.97 dBm, their failure points diverged sharply: 23.97 dBm (reverse), 26.97 dBm (forward), and 32.93 dBm (packaged). **Supplementary Fig. S14**, plots the corresponding minimum insertion loss (IL) versus injected power, revealing a maximum difference of 9.42 dB in failure threshold — a variation arising solely from connection and packaging, not from the acoustic resonators itself.

To investigate this further, we conducted continuous-wave tests on the forward-connected unpackaged filter at different frequencies within its passband (**Supplementary Fig. S15**). Thermal images captured by the infrared (IR) camera show that power dissipation is higher at the band edges (2.3942 GHz, 2.4842 GHz) than at the center (2.4515 GHz), with the spatial temperature profile, and thus the internal power flow distribution, shifting markedly with frequency. This directly visualizes the frequency-dependent, design-specific power routing within the composite acoustic-electromagnetic system, which also confirms that the physical locations and modes of resonator damage varied distinctly for each test frequency.

This combination of thermal, electrical, and mechanical evidence solidifies a key conclusion: the point of failure in a filter is not predetermined by the resonator alone but is a dynamic outcome of system-level variables such as power routing and packaging. Therefore,

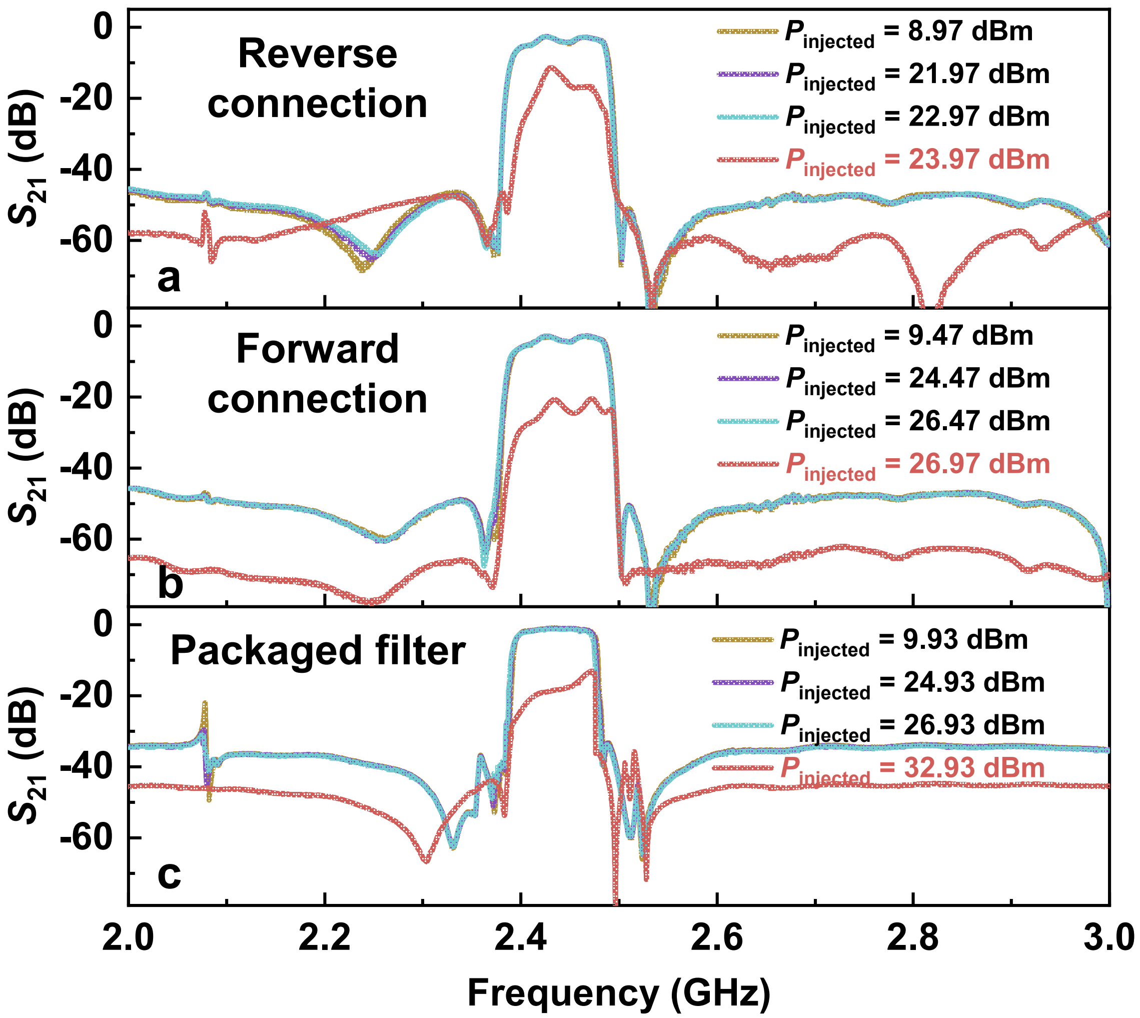

Supplementary Fig. S13 | Power-handling comparison under varied connection and packaging configurations. High-power S_{21} responses for IHP-SAW filters with identical layout but different interfaces: **a,** unpackaged with reverse connection, **b,** unpackaged with forward connection, and **c,** packaged filter with forward connection. The difference in failure points underlines that system-level power handling is also governed by electrical and packaging interfaces, not solely by the inherent resonator architecture itself.

power handling measured at the filter level yields a metric that is intrinsically confounded by design-specific routing, connection, and encapsulation effects. Such a system-level result cannot serve as a direct or fair benchmark for comparing the inherent power-handling capability of different resonator technologies at the device-architecture level. Thus, to isolate and evaluate architectural innovation, we employ transducer-level benchmarking using an active-area-normalized injected power density, providing a more direct and accurate assessment of architectural innovation.

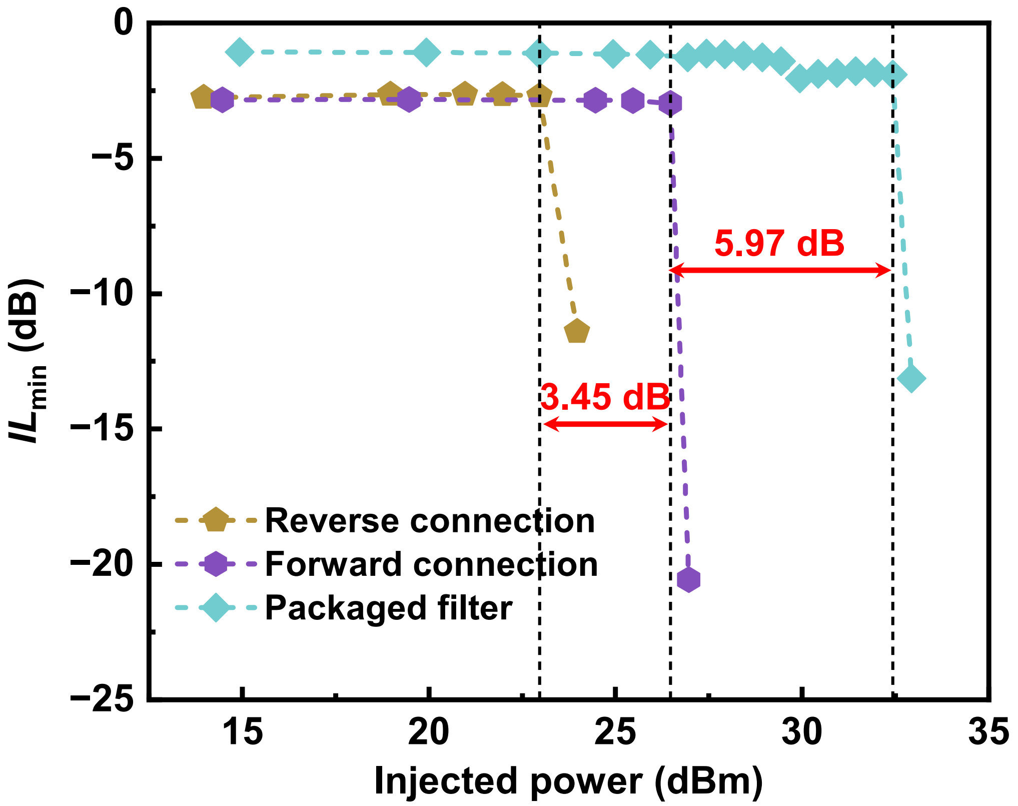

Supplementary Fig. S14 | Failure threshold comparison across interface configurations. Measured minimum insertion loss (*IL*) under high-power testing for the unpackaged (reverse/forward connection) and packaged IHP-SAW filters that share an identical layout. under high-power testing for the unpackaged (reverse/forward connection) and packaged IHP-SAW filters that share an identical layout.

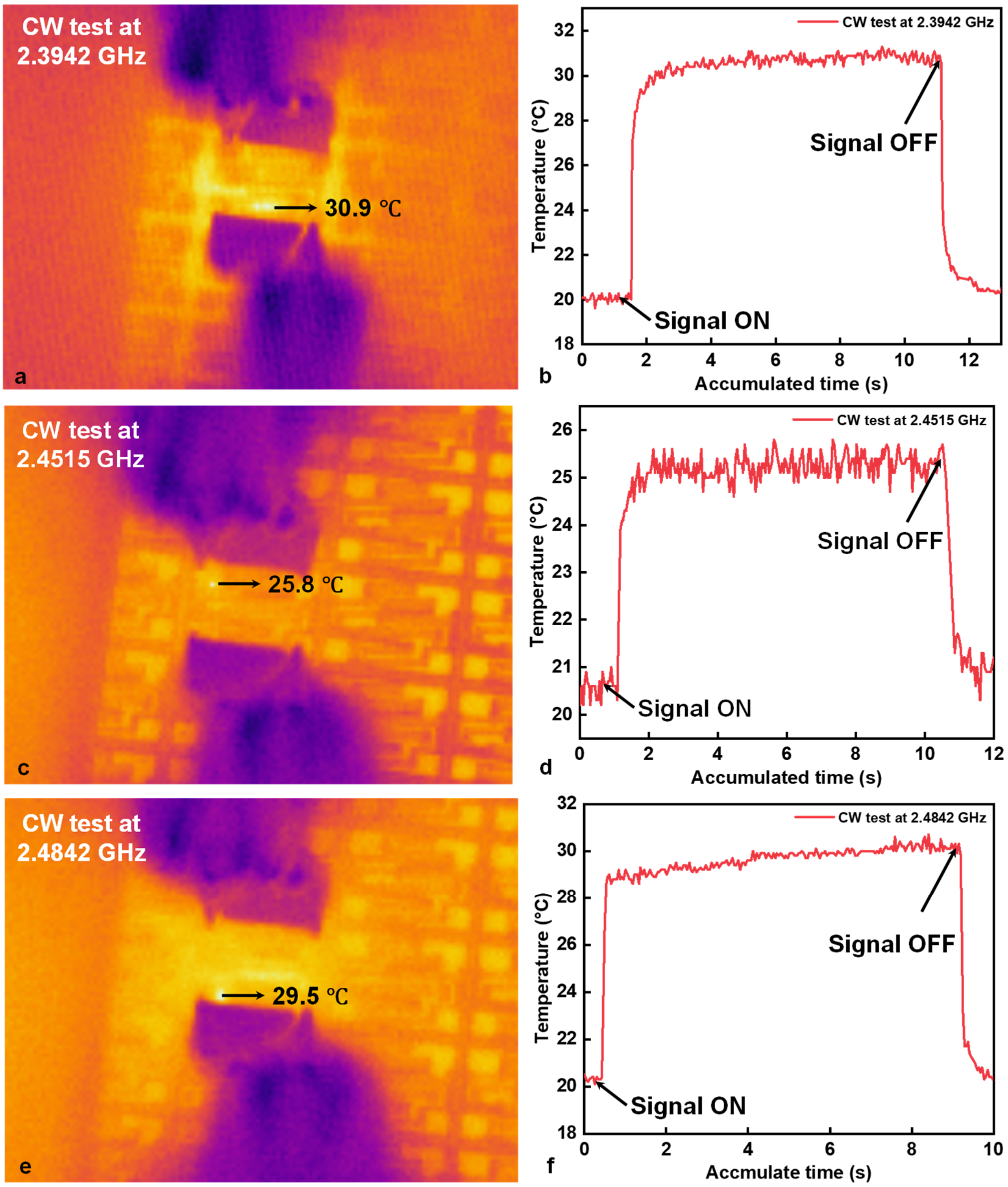

Supplementary Fig. S15 | Frequency-dependent thermal analysis of an unpackaged IHP-SAW filter. Thermal characterization under high-power continuous-wave (CW) excitation at distinct loading frequencies within the passband: **a, b,** 2.3942 GHz (left band edge), **c, d,** 2.4515 GHz (center), and **e, f,** 2.4842 GHz (right band edge). Each frequency pair presents the infrared thermal image (left) and the corresponding transient temperature profile over time (right), revealing how power dissipation and hot-spot location shift with frequency due to system-level power-routing effects.

12 Frequency- and C_0-dependent von Mises stress profiles

In principle, electromigration, which predominantly occurs at f_r, is another critical failure mode for acoustic wave devices. Therefore, to investigate the competing failure mechanisms in acoustic wave devices—specifically, electromigration (driven by current density) versus acoustomigration (driven by mechanical stress)—we performed finite element analysis (FEA) on a SAW transducer unit cell. The goal was to extract the frequency-dependent von Mises stress profile and identify the weakest failure point across the frequency domain. The extraction method is presented in **Supplementary Fig. S16**. A perfectly matched layer (PML) was applied at the substrate bottom to absorb leaked acoustic energy and eliminate spurious reflections. The simulated stress profile was obtained by sweeping the frequency and recording the maximum von Mises stress along the depth (from the IDT top surface into the substrate), which consistently peaked at the critical Au/$LiNbO_3$ interface. The results for devices with different static capacitances were normalized to their respective global maxima for direct comparison.

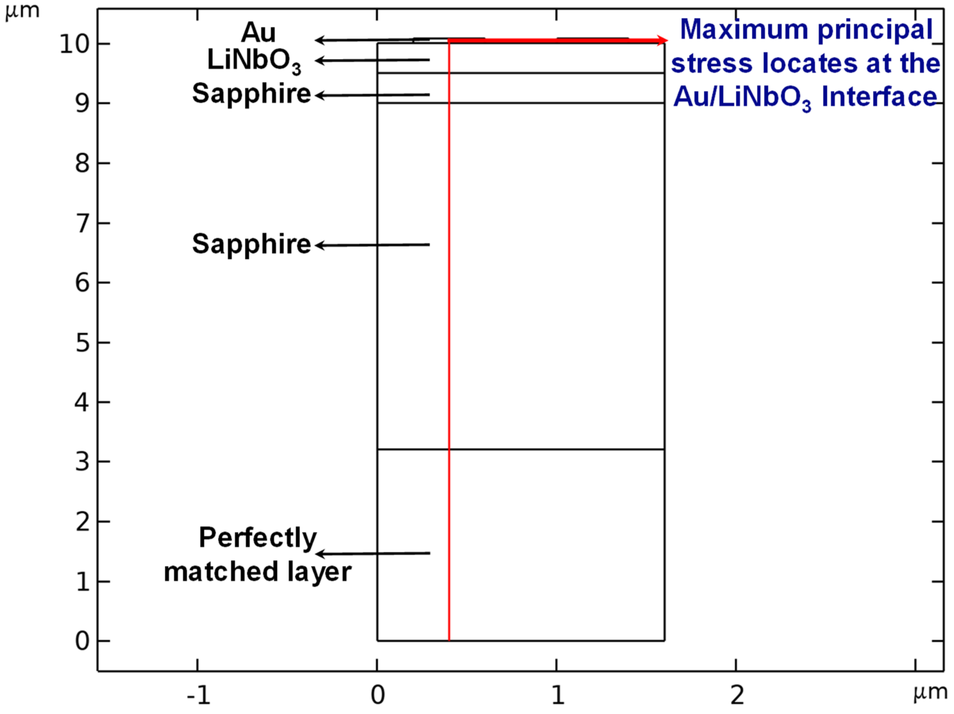

Supplementary Fig. S16 | Method for extracting depth-dependent von Mises stress. Schematic illustrating the finite-element-analysis (FEA) based procedure for evaluating the von Mises stress profile along the vertical axis, from the top surface of the interdigital transducer (IDT) layer down to the sapphire substrate.

The key finding is illustrated in **Supplementary Fig. S17**, where f_r and f_a are marked by dashed lines. At f_r, the transducer behaves as a near-short circuit. This condition maximizes current density, thereby promoting electromigration, but results in a minimal potential difference between IDT fingers, leading to negligible mechanical strain and thus minimal von Mises stress (thus minimizing acoustomigration). Conversely, as the load frequency increases towards f_a, the high impedance leads to a large potential difference, maximizing mechanical stress and acoustomigration risk. Therefore, the dominant failure mechanism and its corresponding "weakest failing point" in the frequency domain are not fixed; they shift between f_r and f_a, depending on the device's electrical design (i.e., its static capacitance), as showcased in **Supplementary Fig. S17**.

To experimentally validate these simulation results, we performed comprehensive high-power characterizations on TF-SAW and LAW devices with different C_0 values across the resonator band. **Supplementary Fig. S18a** presents a comparison of the injected power density thresholds for TF-SAW and LAW DUTs with $C_0 \approx 520$ fF, tested at three selected frequencies: f_r, the frequency of maximum dissipation ($f_{\max}$), and f_a. The exact driving frequencies and their corresponding dissipation coefficients are indicated in the figure.

For the TF-SAW devices, the threshold injected power density at $f_{\max}$ is 28.53 dBm/mm^2, representing the most failure-prone point. At f_r, the threshold is slightly higher at 29.15 dBm/mm^2, while at f_a it increases to 31 dBm/mm^2 due to lower dissipation (**Supplementary Fig. S18a**). These results for the small-C_0 device align with the expectation that failure susceptibility is highest near frequencies where acoustic energy is most strongly coupled. For the LAW device, the thresholds at f_r, $f_{\max}$, and f_a are 38.76, 38.46, and 41.63 dBm/mm^2, respectively. Although the improvement varies slightly across frequencies, the overall enhancement factor of approximately 11-fold for the LAW compared to the TF-SAW remains consistent with our earlier conclusions.

To further illustrate the difference in power handling robustness between the two

architectures, **Supplementary Figs. S18b** and **S18c** show the admittance responses of the TF-SAW and LAW devices before and after high-power stress at their respective maximum-dissipation frequencies. After exposure to 28.53 dBm/mm^2, the TF-SAW device exhibits irreversible damage: spurious modes appear, the Q-factor drops significantly, the k_t^2 decreases, and the static capacitance C_0 is also reduced (**Supplementary Fig. S18b**). In contrast, the LAW device stressed at 37.4 dBm/mm^2 shows only a minor frequency shift, while both k_t^2 and Q remain nearly unchanged (**Supplementary Fig. S18c**). Only when the power density is increased to 38.46 dBm/mm^2 does the LAW device begin to show degradation similar to that of the TF-SAW, yet its performance (e.g., k_t^2) is still superior to the damaged TF-SAW DUT.

We extended the same measurement for devices with $C_0 \approx 5.5$ pF. The frequency offset between f_r and $f_{\max}$ increases with C_0: for $C_0 \approx 520$ fF, the offsets are 44 MHz for TF-SAW and 49 MHz for LAW; for $C_0 \approx 5.5$ pF, they become 121 MHz for TF-SAW and 108 MHz for LAW (**Supplementary Fig. S18d**). At the maximum dissipation frequency, the LAW again demonstrates a marked improvement: the critical power density for the TF-SAW is 28.19 dBm/mm^2, while the LAW withstands 38.75 dBm/mm^2 before showing any notable change, an enhancement factor of 11.38-fold. Post stress admittance comparisons confirm that at 28.19 dBm/mm^2 the TF-SAW is irreversibly damaged, with its admittance ratio drops to 23.8 dB and spurious modes appearing, whereas the LAW stressed at 38.75 dBm/mm^2 exhibits only a slight frequency shift with k_t^2 and Q essentially preserved. Only after exposure to 40.35 dBm/mm^2 does the LAW show similar degradation, with an admittance ratio of 25.4 dB and emerging spurious responses.

In a standard filter design, the static capacitance is typically tuned for 50-Ω impedance matching, which positions the peak power dissipation (and thus the most likely failure point) somewhere between f_r and f_a. For the transducers used in our power-handling tests, the static capacitance was made to complement the spatial resolution of our infrared camera, enabling

clear thermal imaging of the failure epicenter. This design choice thus shifts the point of peak stress (and thus the observed failure) to f_a. This controlled alignment further validates that the observed failures are primarily driven by acoustomigration at the designed stress maximum, rather than by electromigration.

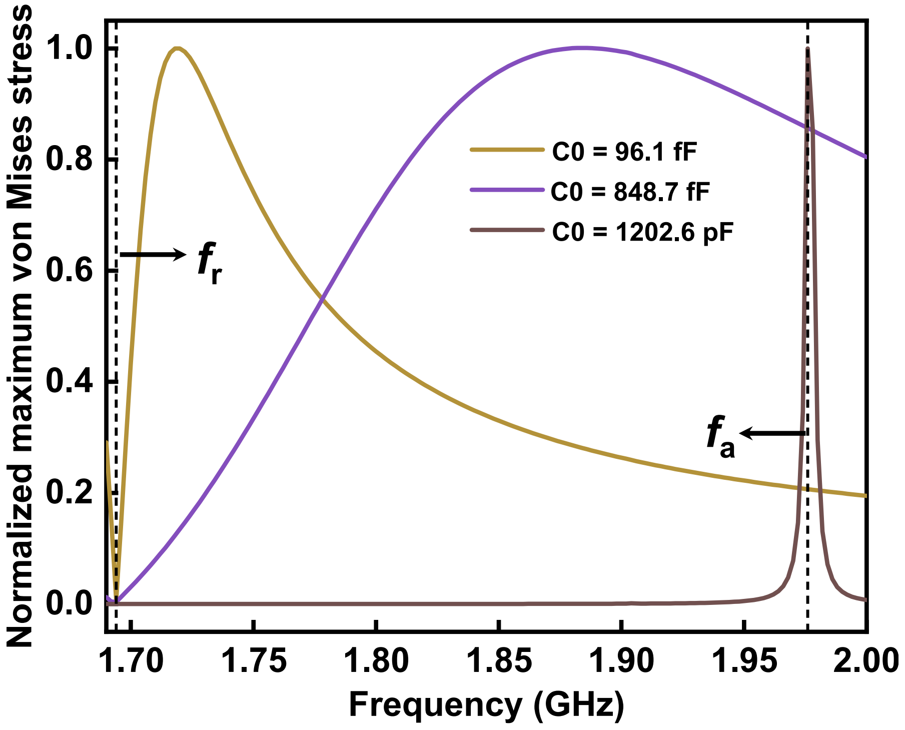

Supplementary Fig. S17 | Simulated frequency- and static capacitance-dependent von Mises stress profiles. The maximum stress is consistently located at the metal/$LiNbO_3$ interface. For each device, all profiles are normalized to the maximum von Mises stress in the depth direction extracted from the frequency sweep simulation.

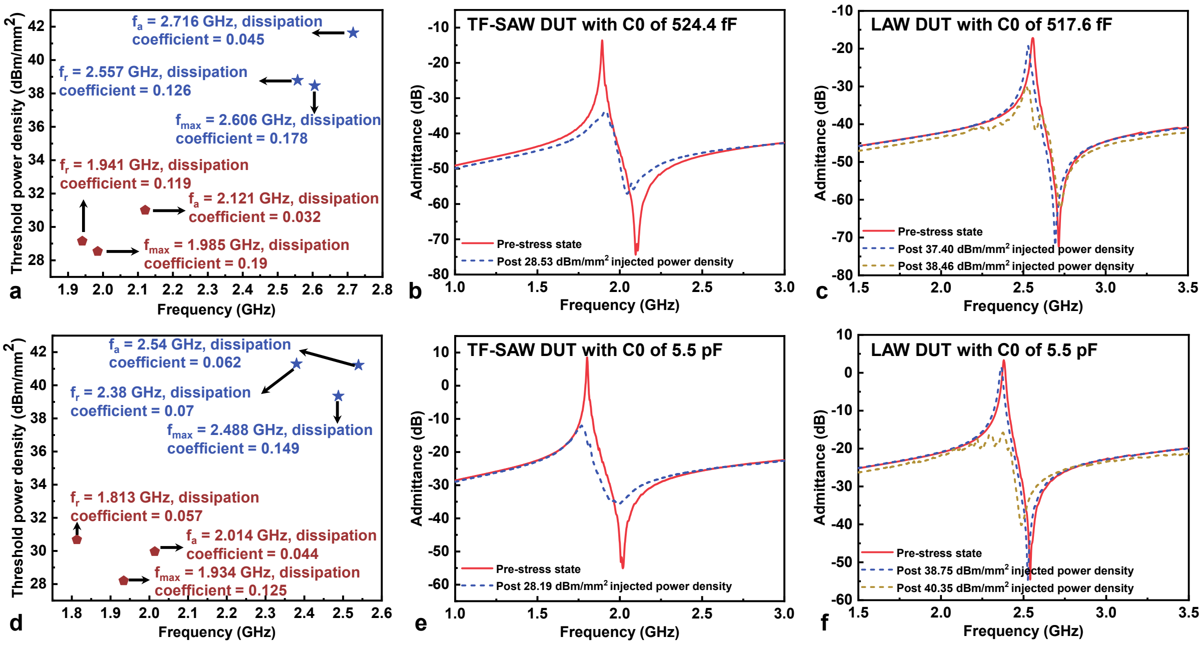

Supplementary Fig. S18 | Power handling characterization of TF-SAW and LAW transducers with varying static capacitance. a, Comparison of injected power density thresholds for TF-SAW and LAW DUTs with $C_0 \approx 520$ fF, measured at three selected frequencies across the resonator band. The driving frequencies and corresponding dissipation coefficients are indicated. **b-c**, Admittance responses before (pre-stress) and after (post-stress) high-power exposure for **b,** a TF-SAW DUT with $C_0 = 524.4$ fF, and **c,** a LAW DUT with $C_0 = 517.6$ fF. **d**, Same as a but for DUTs with $C_0 \approx 5.5$ pF. **e-f**, Corresponding pre- and post-stress admittance comparisons for **e,** a TF-SAW with $C_0 = 5.5$ pF and **f,** a LAW DUT with $C_0 = 5.5$ pF. The post-stress responses were obtained by small-signal frequency sweeps after the devices were subjected to continuous-wave high-power stress at the frequency corresponding to maximum dissipative absorption.

13 Measured *S*-parameter responses for TF-SAW and LAW transducers

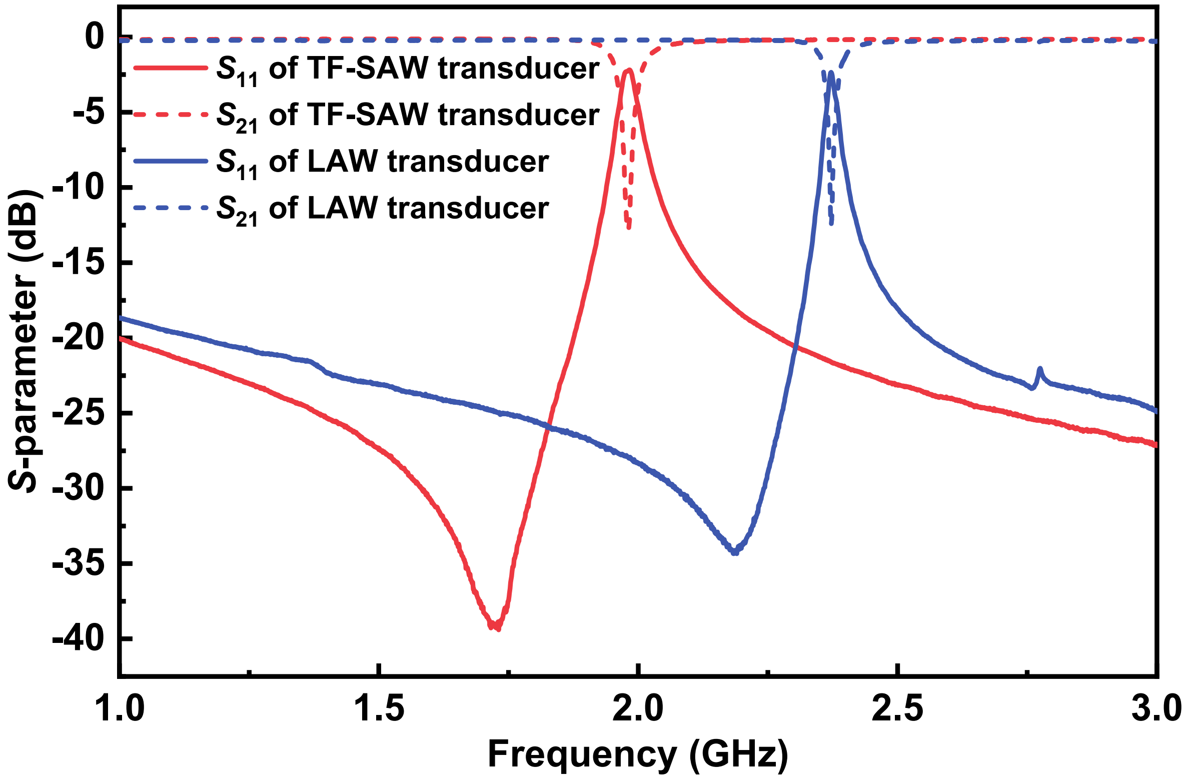

Supplementary Fig. S19 | *S*-parameter responses for TF-SAW and LAW transducers measured under small-signal conditions.

14 Dissipated power distribution of a typical LAW transducer vs. frequency

This section combines modified Butterworth-Van Dyke (mBVD) fitting and power flow simulation results for a typical LAW transducer to investigate the influence of different energy loss mechanisms and measurement port configurations on power dissipation profiles, with the aim of providing analysis for optimal driving frequency selection in high-power testing. **Supplementary Fig. S20** presents the admittance response of a typical LAW transducer and its mBVD fitting results. The parameters of each component in the mBVD model are summarized in the inset. Simulation results for the dissipated power within the LAW transducer in one-port and two-port configurations are illustrated in **Supplementary Fig. S20b** and **Fig. S20c**, respectively. The f_r and f_a are labelled by dashed lines. Note that different loss mechanisms dominate under different driving frequencies. The frequency-favoring dissipated power peak is located between f_r and f_a. For the one-port configuration, peak power dissipation occurs near f_r, where the reflection coefficient is minimized. Otherwise, input power is strongly rejected with port impedance mismatch. At f_r, nearly 23.5% of input power is dissipated, dominated by ohmic loss in the electrodes due to peak current density. The peak of viscous losses (modeled by R_m) is also the strongest nearby f_r. While a similar frequency-dependent power dissipation trend is observed in the two-port configuration, the dissipation peak shifts toward f_a, with only 7.8% of input power absorbed by the LAW transducer. Notably, viscous losses constitute the largest proportion of total dissipation in the two-port case. At f_a, minimal current flow renders ohmic losses negligible for both configurations. Additionally, power dissipation in R_0 (dielectric loss) exhibits an inverse trend relative to ohmic losses. Far from the passband, dielectric and ohmic losses dominate, while viscous losses diminish to negligible levels. Under these conditions, the LAW transducer can be treated as a capacitor. These simulation results clarify that the contribution of different energy loss mechanisms to total energy dissipation varies from frequency to frequency[14–16], highlighting the importance of frequency selection for accessing the power handling capability of each testing transducer.

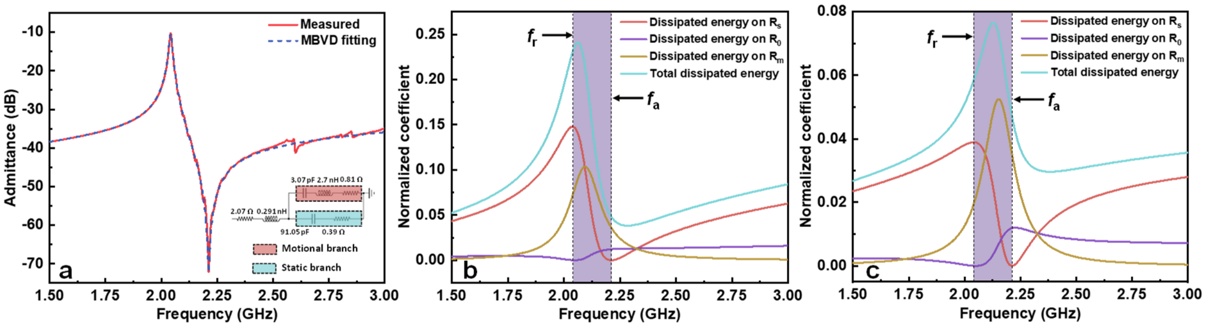

Supplementary Fig. S20 | mBVD fitting and power dissipation simulation results. a, Measured and fitted admittance curves of a typical LAW transducer. The inset plot summarizes the parameters of each component in the mBVD model. Power dissipation distribution in the LAW transducer with **b,** one-port configuration and **c,** two-port configuration. f_r and f_a denote the resonant and anti-resonant frequency of the LAW transducer.

15 High-power test for SAW transducer at f_r

To investigate the dominant failure mechanism in acoustic transducers, we characterized the RF power budget and conducted high-power stress tests at the resonant frequency (f_r). **Supplementary Fig. S21** presents the measured power reflection, transmission, and dissipation for a SAW transducer across a 10-MHz band centered at f_r. Within this band, the power reflection coefficient is negligible (0.00017), and the transmission coefficient is high (0.9834), indicating minimal power rejection. The associated dissipation at f_r, while slightly elevated relative to adjacent bands, remains orders of magnitude lower than that near the anti-resonant frequency (f_a).

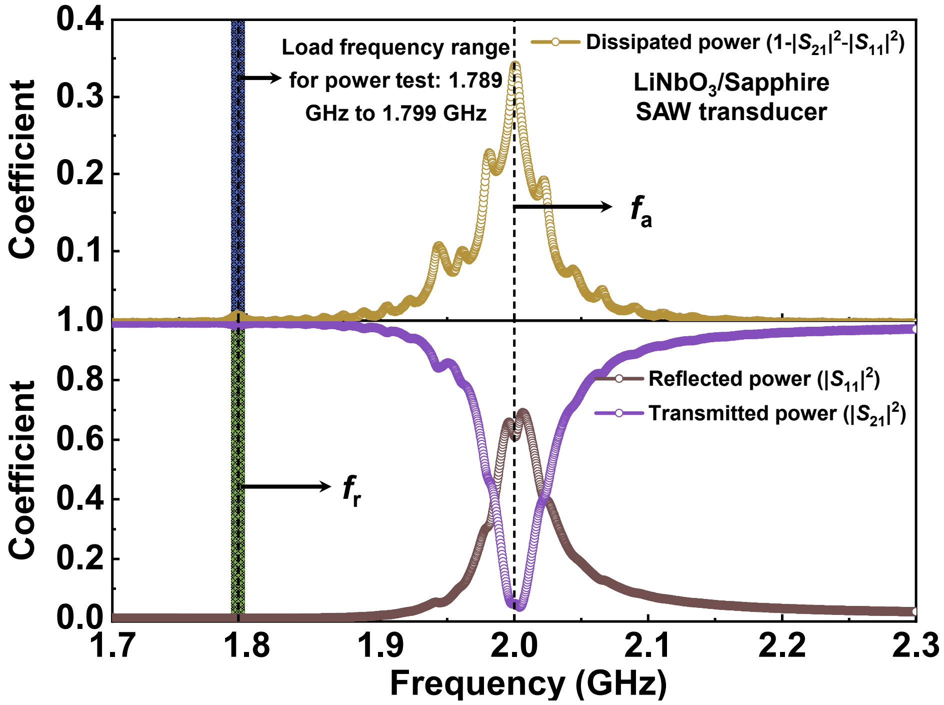

Supplementary Fig. S21 | Power budget analysis of a SAW transducer. a, Measured dissipated, reflected, and transmitted power coefficients for the $LiNbO_3$/Sapphire TF-SAW transducer under a -15 dBm incident load. Frequency range for power test: 1.789–1.799 GHz.

Following the test protocol established in the main text, the device was then subjected to a high-power stress test with injected power density swept from 25.93 dBm/mm^2 to 45.93 dBm/mm^2 — exceeding the failure point of the LAW transducer reported in **Fig. 5c**. Notably, no appreciable steady-state temperature rise was detected via infrared thermography, even at these high-power levels. Post-stress low-power (–15 dBm) S-parameter measurements,

performed after re-calibration, show no degradation in device performance (**Supplementary Fig. S22**), as confirmed by the almost unchanged frequency response.

The apparent robustness near f_r for our large-C_0 DUT (C_0 = 1083 pF) is not due to poor matching, but rather to the current path distribution dictated by the mBVD model. At f_r, the static branch impedance is only ~0.14 Ω, while the motional branch impedance is ~0.6 Ω. Consequently, most of the RF current flows through the static branch, effectively bypassing the motional branch where acoustomigration would occur. In contrast, for a small-C_0 DUT, the static branch impedance is much larger than that of the motional branch, forcing current through the motional branch and enabling acoustomigration-induced failure. This observation reinforces our central claim: the device becomes vulnerable when significant acoustic energy is present in the motional branch. The combination of the measurement results in **Supplementary Section 12 and Section 15**, including minimal electrical dissipation at f_r, the absence of significant temperature-rise, and the preservation of device performance post-stress, provides strong, multi-faceted evidence that the predominant failure mechanism in these acoustic transducers is acoustomigration (driven by high mechanical stress) rather than electromigration (driven by current density and Joule heating).

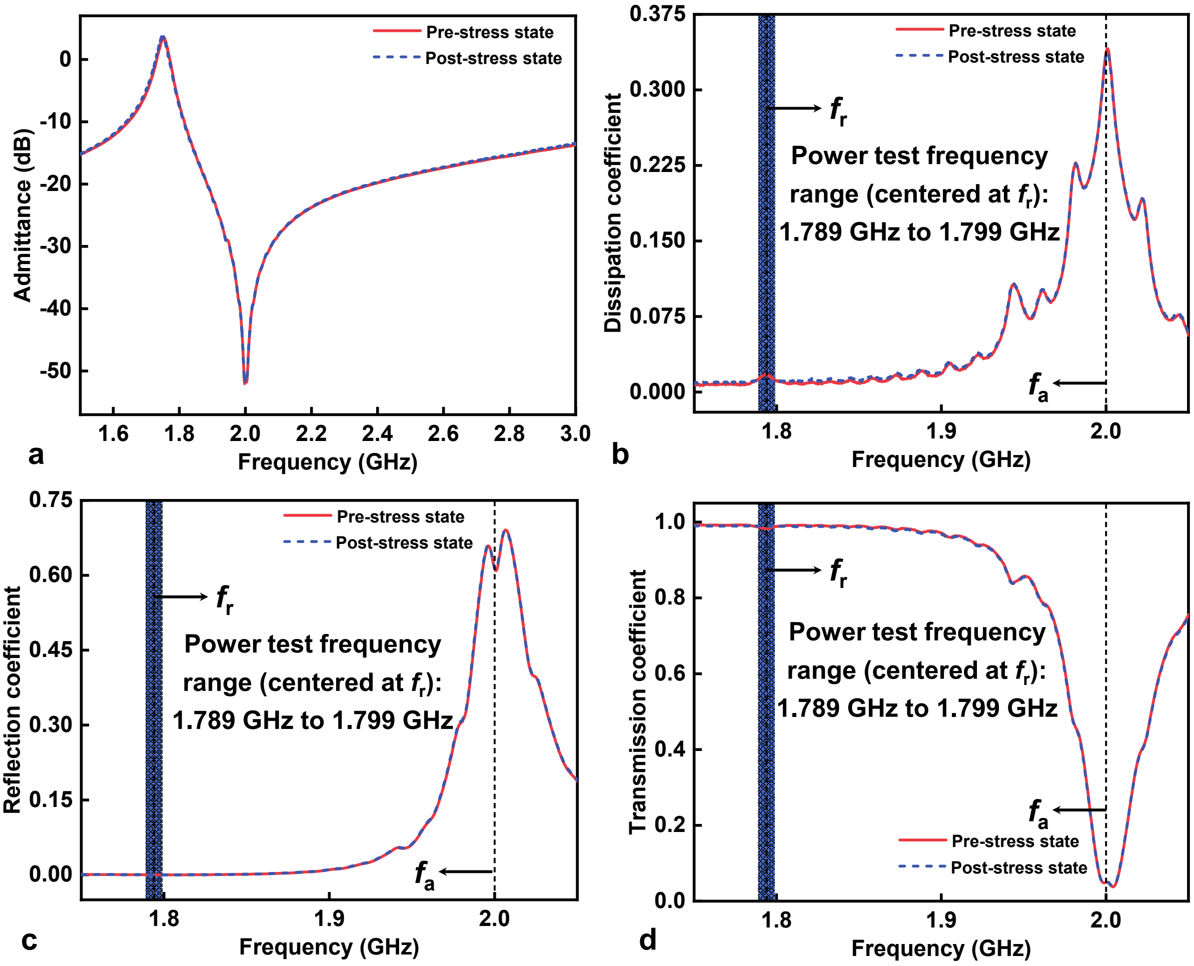

Supplementary Fig. S22 | Pre- and post-stress characteristics of the SAW transducer. Key parameters for the $LiNbO_3$/Sapphire TF-SAW DUT measured under a −15 dBm incident load: **a** Admittance curves, **b**, dissipation, **c**, reflection, and **d**, transmission coefficients for the $LiNbO_3$/Sapphire TF-SAW DUT, measured under small-signal conditions (−15 dBm) before and after being subjected to high-power stress. The high-power stress was applied at a maximum injected power density of 45.93 dBm/mm^2 over a narrow frequency range centered at the resonant frequency f_r (1.789–1.799 GHz).

16 Thermal analysis of a LAW transducer under high RF loads

Based on the electrical modelling method described in **Supplementary Section 15**, power dissipation can be accurately calculated for each frequency, providing an estimate of the optimal frequency range required to heat the transducer effectively. To verify this methodology for power durability testing, thermal images were recorded during frequency sweeps at varying input power levels (input power is defined as the isolator's output power). The actual dissipated power depends on driving frequencies due to differences in reflection, dissipation, and transmission characteristics at each frequency, as shown in **Supplementary Fig. S23a**. The simulated power dissipation profile aligns closely with post-calculated experimental data. From the power dissipation curves, it can be observed that most RF power is absorbed by the LAW transducer at f_a. Following the analysis of frequency characteristics, IR radiation is recorded and calibrated using the emissivity calibration method detailed in the main text. **Supplementary Fig. S23b** indicates that the temperature rises scales with the calculated dissipated power. Despite the compensated TCF remaining low, the maximum temperature rise shifts to lower frequencies, which can be attributed to the non-zero TCF as temperature increases. Temperature rise measurements under varying input power loads further highlight the criticality of the tested frequency range: as input power increases, both temperature and dissipated power rise, driving f_a downward, and these behaviors again amplify the dissipated power until device failure occurs[17,18].

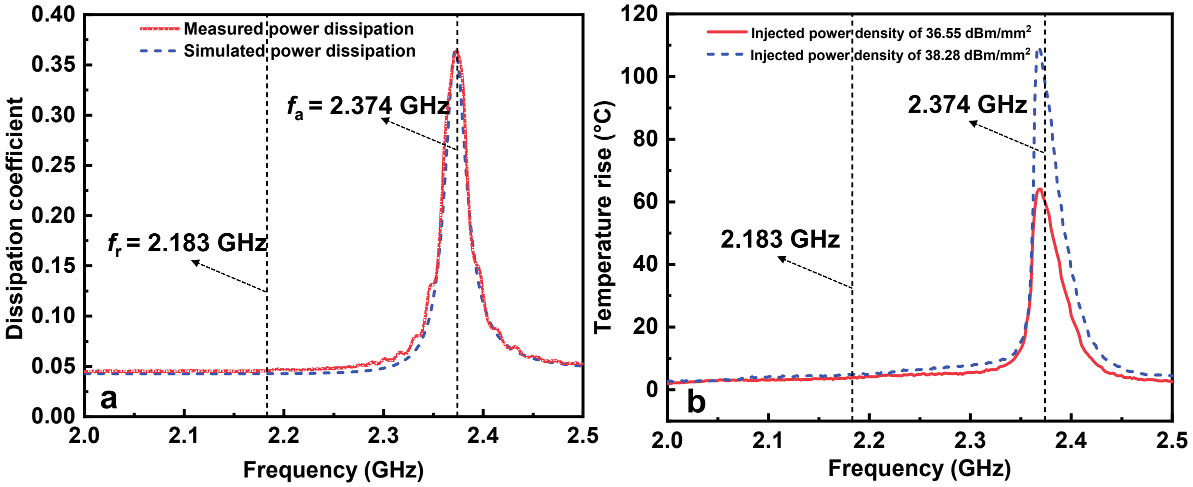

Supplementary Fig. S23 | Power and thermal analysis of a LAW transducer under high RF load. **a,** Post-calculated and simulated power dissipation profiles in the LAW transducer. **b,** Temperature curves of the LAW transducer during frequency sweeps at varying input power levels.

17 Performance comparisons of TF-SAW transducers before and after power tests

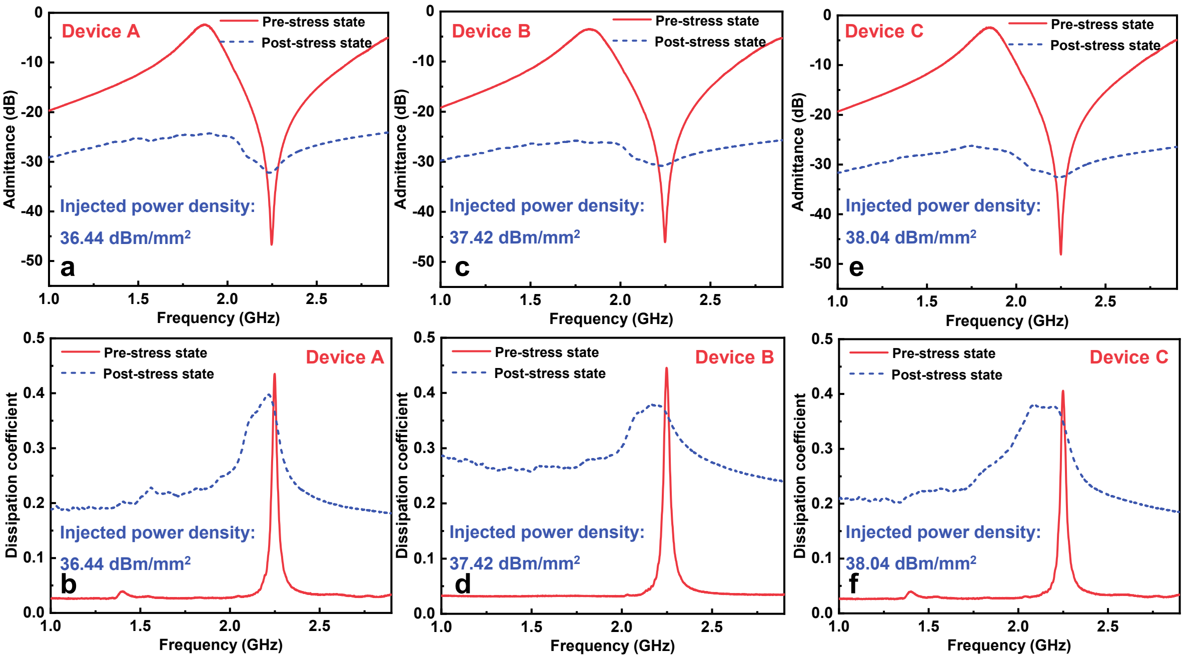

Supplementary Fig. S24 | Performance comparison of TF-SAW transducers before and after high-power loads under different temperatures. a, Admittance curves and **b,** power dissipation profiles of Device A before and after the high-power load test under −85 °C. **c,** Admittance curves and **d,** power dissipation profiles of Device B before and after the high-power load test under −45 °C. **e,** Admittance curves and **f,** power dissipation profiles of Device C before and after the high-power load test under −5 °C.

18 Performance comparisons of LAW transducers before and after power tests

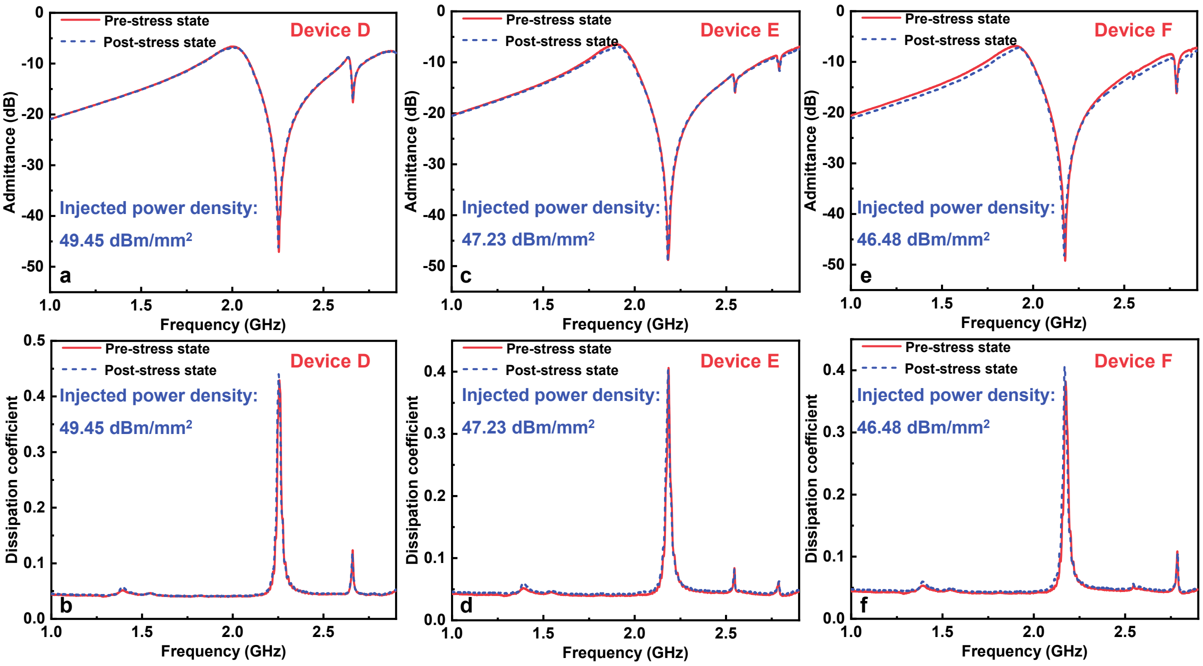

Supplementary Fig. S25 | Performance comparison of LAW transducers before and after high-power loads under different temperatures. a, Admittance curves and **b,** power dissipation profiles of Device D before and after the high-power load test under −85 °C. **c,** Admittance curves and **d,** power dissipation profiles of Device E before and after the high-power load test under −5 °C. **e,** Admittance curves and **f,** power dissipation profiles of Device F before and after the high-power load test under 80 °C.

19 Acoustomigration investigation on broken TF-SAW transducers (State B)

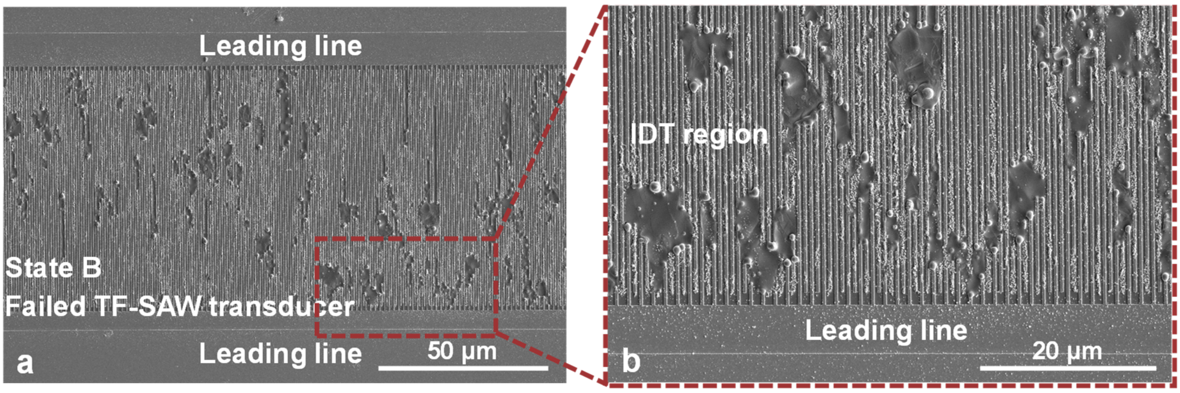

Supplementary Fig. S26 | Post-failure analysis of a TF-SAW transducer (State B). a, Low-magnification top-view SEM image showing random and widespread damage across the entire transduction region. **b,** High-magnification image of a selected area, revealing irregular electrode damage, including localized melting and metal balling, accompanied by numerous irreversible cracks propagating into the underlying $LiNbO_3$ substrate.

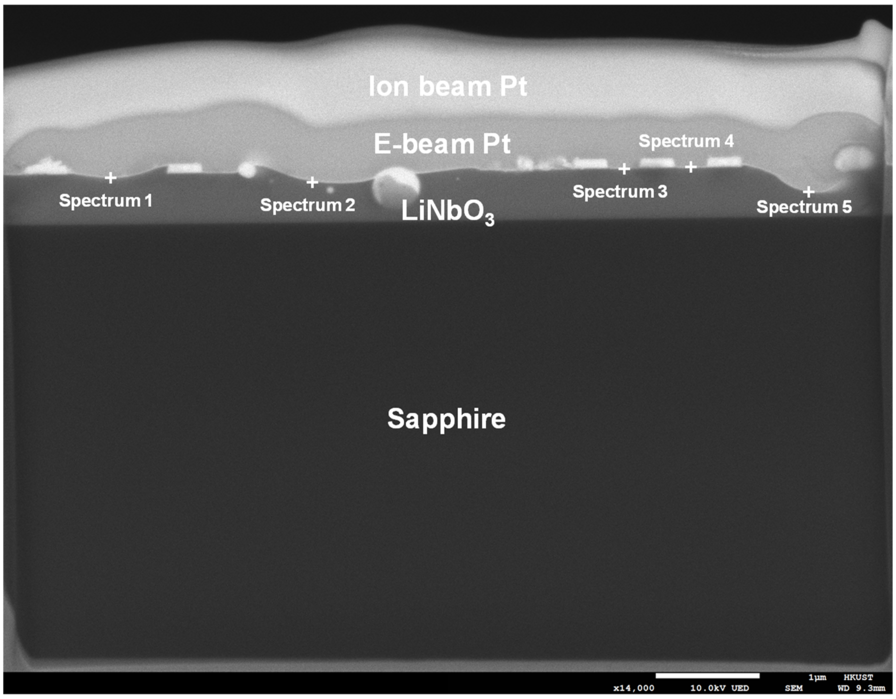

Supplementary Fig. S27 | Acoustomigration investigation on a broken TF-SAW transducer (State B). A cross-sectional scanning electron microscope (SEM) image of a broken TF-SAW transducer following high-power testing. The $LiNbO_3$ layer exhibits significant deformation, with a distinct continuous bright line observed at its surface. An energy-dispersive X-ray spectroscopy (EDS) analysis was performed at five labeled points along this feature, located at the Pt/$LiNbO_3$ interface, as marked in **Supplementary Fig. S11**. At the marked locations, gold is identified as the second most abundant element at these sites, even though it should not be present under small signal working conditions. This anomalous presence strongly indicates that acoustic-induced migration under high-power conditions

has caused gold to accumulate in these regions.

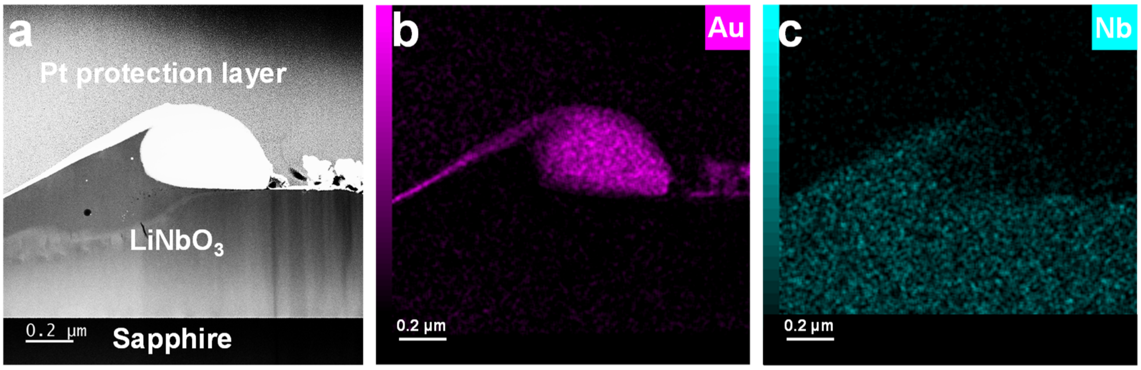

Supplementary Fig. S28 | EDS mapping of broken TF-SAW transducers (State B). a, HAADF imaging STEM image illustrates the zoomed-up view of a failed TF-SAW transducer. Corresponding elemental mapping results for **b,** Au and **c,** Nb in **(a)**. The EDS mapping results confirm the severely deformed $LiNbO_3$ layer and IDTs under high-power loads.

Supplementary Table S2. Elemental analysis at the five labelled sites of the failed TF-SAW transducer

	**Spectrum 1**	**Spectrum 2**	**Spectrum 3**	**Spectrum 4**	**Spectrum 5**
**Pt (M series)**	27.4 %	27.8 %	24.5 %	26.4 %	19.0 %
**Au (M series)**	22.0 %	20.4 %	21.9 %	17.8 %	23.6 %
**Nb (L series)**	11.2 %	10.3 %	14.1 %	15.2 %	19.8 %
**O (K series)**	10.0 %	11.5 %	12.2 %	13.4 %	18.7 %
**C (K series)**	15.6 %	14.5 %	12.7 %	12.8 %	11.6 %
**Cu (K series)**	7.3 %	7.7 %	7.6 %	6.6 %	6.2 %
**Al (K series)**	5.3 %	6.5 %	5.7 %	6.3 %	0 %
**Ni (K series)**	0.8 %	0.8 %	0.6 %	0.8 %	0.6 %
**Cr (K series)**	0 %	0 %	0 %	0.4 %	0.5 %
**Ga (K series)**	0.4 %	0.5 %	0.7 %	0.3 %	0 %

20 EDS mapping of a failed LAW transducer (State D)

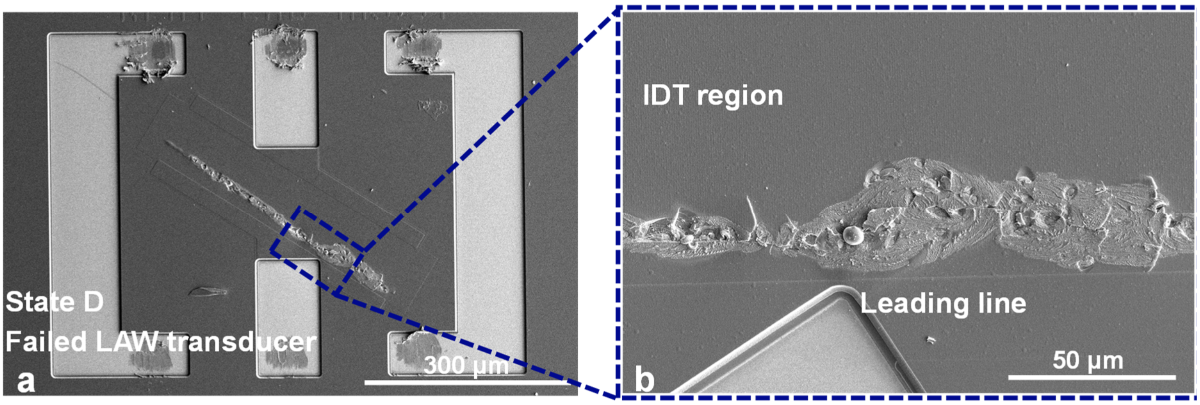

Supplementary Fig. S29 | Post-failure analysis of a LAW transducer (State D). a, A zoomed-out view demonstrating that structural damage is highly localized for LAW DUT at State D, in contrast to the widespread failure observed in conventional SAW devices. **b,** A magnified view of the failure region near the signal output terminal, showing a well-defined, near-parallel crack along the busline direction. The localized nature of the crack indicates that stress concentration, rather than global acoustic overload, governs the ultimate failure under high-power stress.

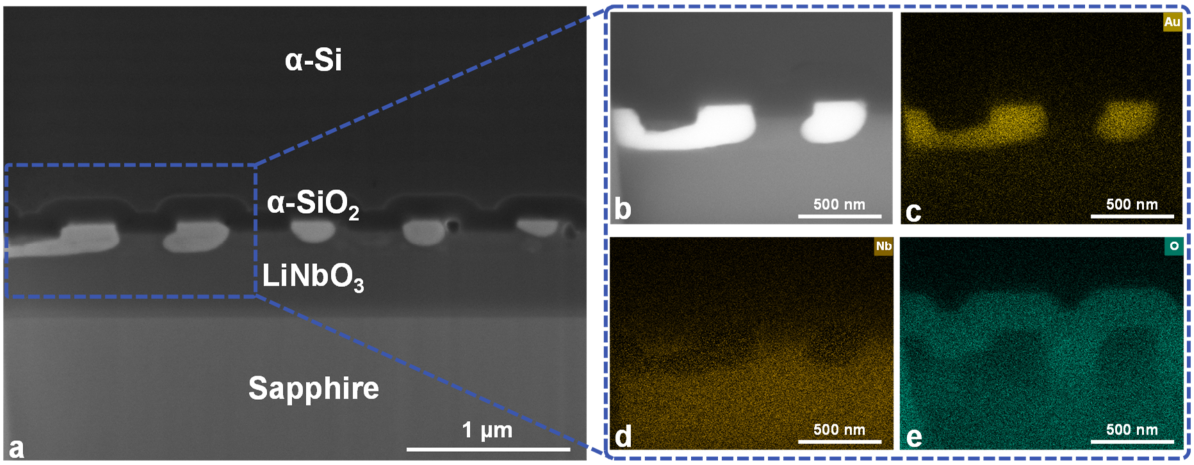

Supplementary Fig. S30 | EDS mapping of broken LAW transducers (State D). a, Zoomed-out and **b,** zoomed-in cross-sectional scanning electron microscope (SEM) image of a broken LAW transducer. Corresponding elemental mapping results for **c,** Au, **d,** Nb, and **e,** O in **(b)**. No acoustomigration behavior can be observed for the LAW transducer after ultra-high power loads, as illustrated in **(a)**. The IDTs shown in **(a)** exhibit severe morphological deformation. Furthermore, localized Nb accumulation is observed above the interface of a pair of interconnected IDTs, suggesting stress-induced material redistribution during mechanical deformation. Although minor delamination occurs between the α-SiO_2 insulating layer and IDTs, the top cladding layers retain conformal coverage across most of the IDT array, highlighting their structural integrity under high-power operational conditions

21 Influence of residual stress of the silicon cladding layer on device performance

Residual stress control is of paramount importance in thick silicon cladding layer deposition, as large residual stress can cause irreversible device damage and modify the material properties of the adjacent layer[19]. Surface profiler KLA Tencor P-7 was utilized to measure the surface profile of a 4-inch silicon wafer before and after α-Si deposition. Average residual stress in thick α-Si can be calculated from the measured profilometry data.

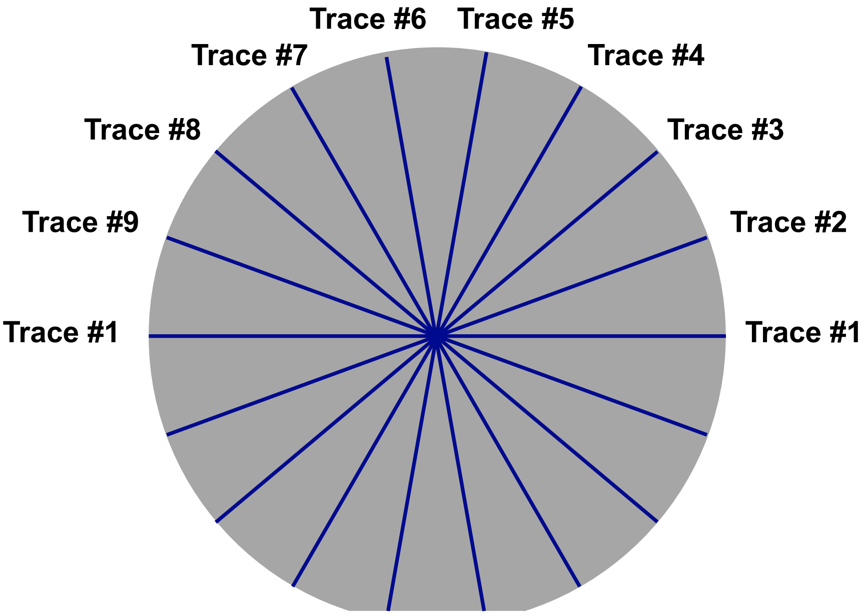

Supplementary Fig. S31. | **Methodology for residual stress extraction via wafer curvature measurement.** Schematic of the radius of curvature (ROC) measurement for a 4-inch wafer before and after α-Si deposition. Residual stress was extracted using the complete Stoney equation applied to nine uniformly distributed scans on a 4-inch wafer.

Profiles of 8-cm length and 5-μm sweeping resolution were obtained by rotating the 4-inch silicon (100) wafer and passing through the center, as shown in **Supplementary Fig. S31**. Subsequently, measured data was fitted by a fifth-order polynomial and utilized to calculate the radius ($R_{(x)}$) of curvature for each data point given by

$$R_{(x)} = \frac{[1 + (dy/dx)^2]^{3/2}}{d^2y/dx^2}, \tag{S13}$$

where y is the height of difference trace and x refers to the position. The residual stress ($\sigma_{(x)}$) for each data point can be calculated by the Stoney equation[20]:

$$\sigma_{(x)} = \frac{1}{6R_{(x)}} \frac{E}{1-\nu} \frac{t_s^2}{t_f}, \tag{S14}$$

where E refers to the Young's modulus of the silicon (100) substrate, ν the Poisson's ratio for the silicon (100) substrate, t_f and t_s is the thickness of α-Si thick film and the silicon (100) substrate. Finally, the average residual stress was determined by averaging the stress derived through the surface profiles.

Supplementary Fig. S32a presents the measured average radius of curvature (ROC) and its associated error bar for each of the nine traces, represented as a scatter plot with a fitted curve. The ROC is the primary experimental observable. Subsequently, the mean residual stress for each trace was calculated from its average ROC using Stoney's equation. **Supplementary Fig. S32b** shows the calculated average stress and its error bar for each trace, also as a scatter plot with a fit. Upon recalculating the weighted average after excluding two clear outliers — Trace #2 and Trace #8 (anomalously high ROC values, potentially from measurement near the wafer edge) — we obtain a refined average residual stress of 50.2 ± 6.9 MPa (with a corresponding average ROC of 337 ± 51 m).

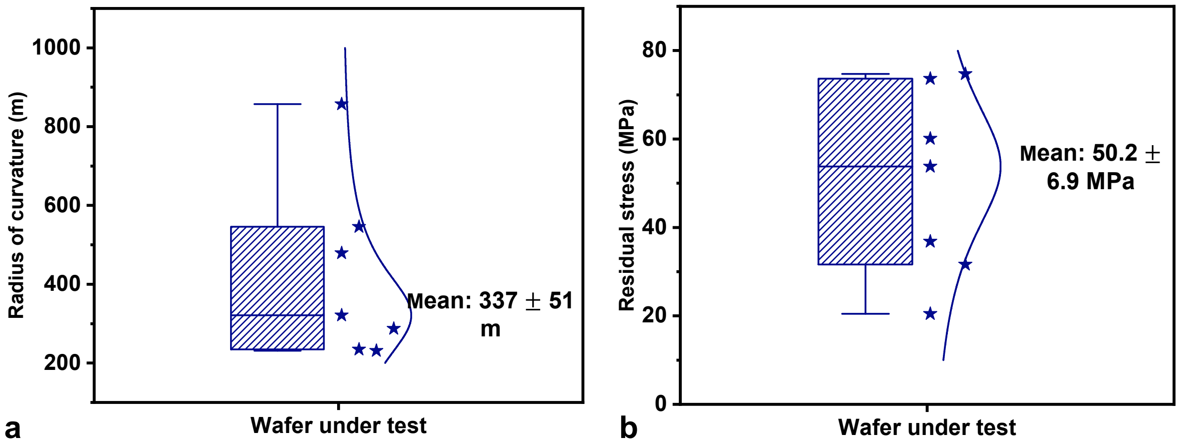

Supplementary Fig. S32. | Statistical analysis of multi-trace curvature measurements. Statistical distribution of the **a,** ROC and **b,** calculated residual stress for Traces #1 through #9, measured across the wafer. Data are presented as mean ± standard deviation, with average values of 337 ± 51 m (ROC) and 50.2 ± 6.9 MPa (low tensile residual stress).

Supplementary Fig. S33 shows measured admittance responses and Bode Q curves on LAW transducers with varying residual stresses in the silicon cladding layer. Notably, the LAW

transducer with high residual stress in the silicon cladding layer exhibits a small k_t^2 of 9.55%, AR of 40 dB, and Bode-Q_{max} of 237. In contrast, the LAW transducer with near-zero residual stress in the silicon cladding layer significantly improves device performance, yielding a larger k_t^2 of 15.34%, AR of 55 dB, and Bode-Q_{max} of 410.

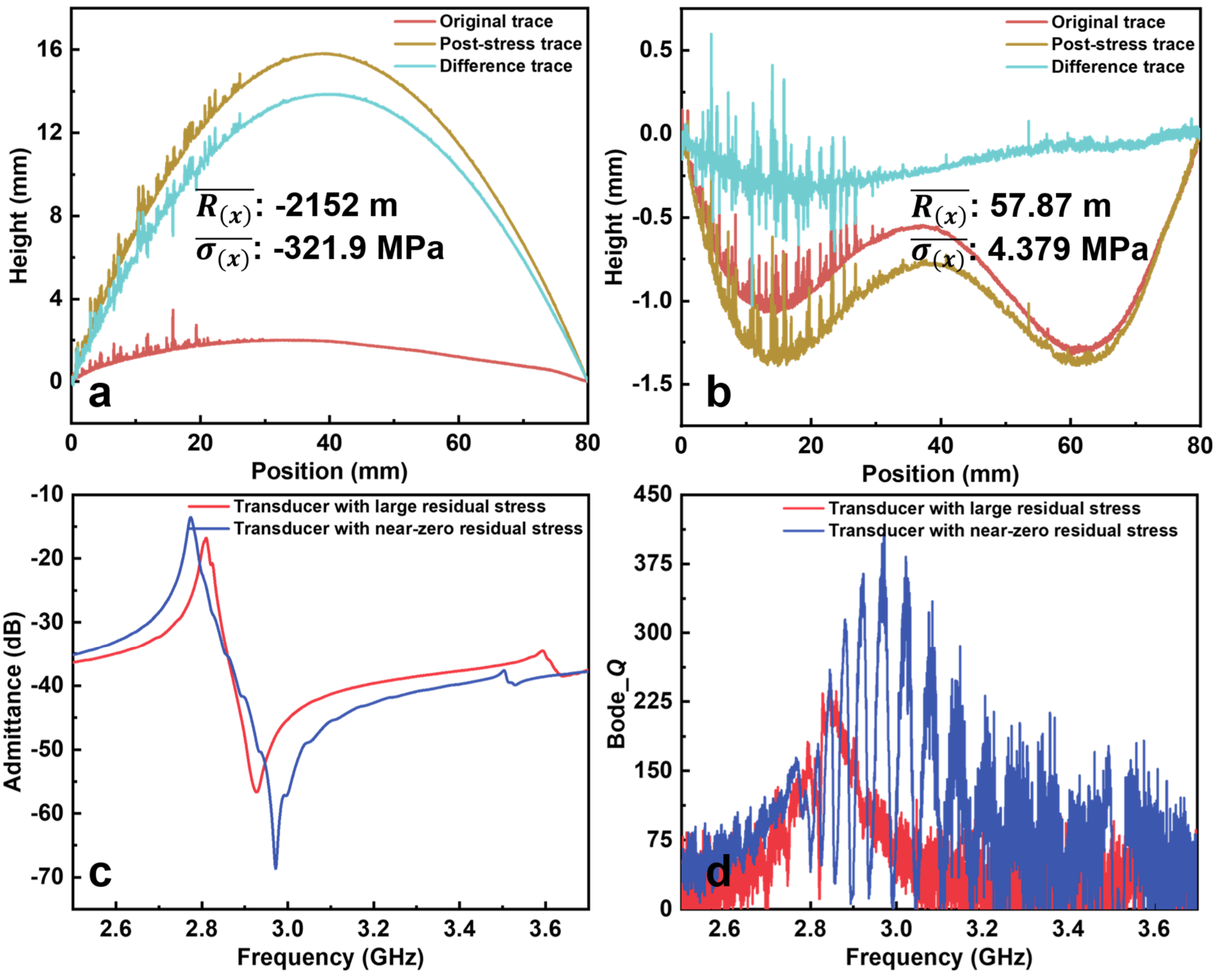

Supplementary Fig. S33 | Influence of residual stress in silicon cladding layer on device performance. Surface profiles of the α-Si thick film under **a,** high residual stress and **b,** near-zero residual stress. The red, yellow, and cyan curves represent the pre-deposition profile, post-deposition profile, and differential profile (used to calculate residual stress in the silicon cladding layer), respectively. **c,** Admittance curves and **d,** Bode Q curves comparing LAW transducers with varying residual stresses in the silicon cladding layer.

22 Calculation method of reflection coefficients for high-power tests

To validate the reflected coefficients extraction methodology, we compared the de-embedded S_{21} (obtained using the power test setup in **Supplementary Fig. S10**) with S_{21} measured directly in the VNA-only test loop (**Supplementary Fig. S34a**). The VNA output power in the power test setup was specifically set to match the load power delivered to the DUTs with that of the VNA-only test loop with calibrated reference plane. The close agreement between the de-embedded S_{21} (via port-extension calibration) and the directly measured S_{21} confirms the accurate de-embedding method. Building on mBVD fitting results under small-signal conditions, we further modified the mBVD model to fit the de-embedded S_{21} under increasing load power by decreasing f_r and Q, as indicated in **Supplementary Fig. S34b.** In summary, reflected and injected power coefficients derived via this mBVD fitting method perfectly match those calculated from direct measurements, validating the de-embedding methodology (**Supplementary Figs. S34c-S34d**).

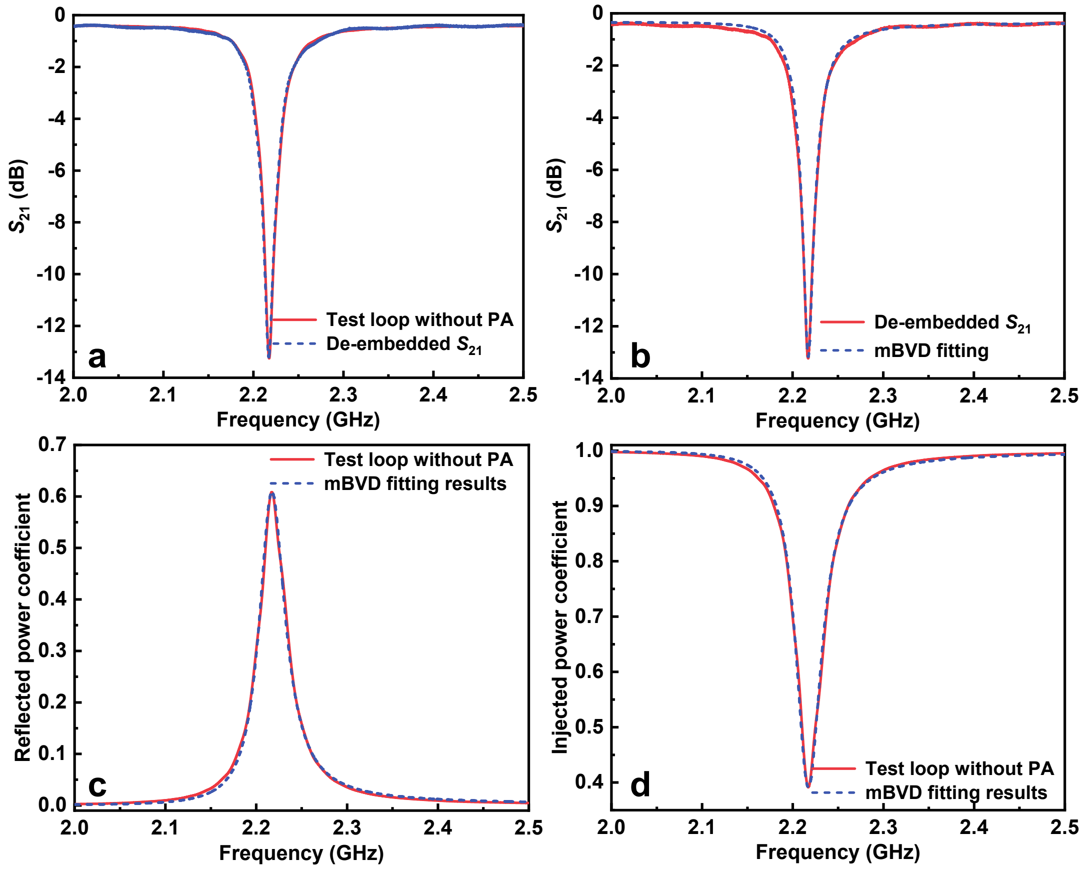

Supplementary Fig. S34 | Extraction method of reflection coefficients for high-power tests. **a,**

Comparison between directly measured S_{21} and de-embedded S_{21} of a LAW transducer under a load power of 10 dBm. **b,** mBVD fitted and de-embedded S_{21} of the LAW transducer. **c,** Reflection coefficient characteristics calculated from directly measured results and mBVD fitting results. **d,** Injected power profiles obtained from directly measured results and mBVD fitting results.

References

1. Wang, Y., Hashimoto, K., Omori, T. & Yamaguchi, M. Change in piezoelectric boundary acoustic wave characteristics with overlay and metal grating materials. *IEEE Trans. Ultrason. Ferroelectr. Freq. Control* **57**, 16–22 (2010).
2. Hashimoto, K. -y., Watanabe, Y., Akahane, M. & Yamaguchi, M. Analysis of acoustic properties of multi-layered structures by means of effective acoustic impedance matrix. In *Proc.* 1990 *IEEE Symposium on Ultrasonics* (IUS) 937–942 (IEEE, 1990).
3. Qian, F., Ho, T. F. & Yang, Y. Twist piezoelectric coupling properties to suppress spurious modes for lithium niobate thin-film acoustic devices. In *Proc. 2023 IEEE/MTT-S International Microwave Symposium (IMS)* 907–910 (IEEE, 2023).
4. Hashimoto, K. *et al.* Revisiting piston mode design for radio frequency surface acoustic wave resonators. In *Proc. 2022 IEEE MTT-S International Conference on Microwave Acoustics and Mechanics (IC-MAM)* 60–63 (IEEE, 2022).
5. Xu, H. *et al.* SAW filters on $LiNbO_3$/SiC heterostructure for 5G n77 and n78 band applications. *IEEE Trans. Ultrason. Ferroelectr. Freq. Control* **70**, 1157–1169 (2023).
6. Qian, F., Zheng, J., Xu, J. & Yang, Y. Heterogeneous interface-enhanced thin-film SAW devices using lithium niobate on Si. *IEEE Microw. Wirel. Technol. Lett.* **35**, 123–126 (2025).
7. Shen, J. *et al.* Suppressed transverse mode generation in TF-SAW resonators based on $LiTaO_3$/Sapphire. *IEEE Electron Device Lett.* **45**, 2241–2244 (2024).
8. Shen, J. *et al.* A low-loss wideband SAW filter with low drift using multilayered structure. *IEEE Electron Device Lett.* **43**, 1371–1374 (2022).
9. Su, R. *et al.* Wideband and low-loss surface acoustic wave filter based on 15° YX-$LiNbO_3$/SiO_2/Si structure. *IEEE Electron Device Lett.* **42**, 438–441 (2021).
10. Xu, H. *et al.* Large-range spurious mode elimination for wideband SAW filters on $LiNbO_3$/SiO_2/Si platform by $LiNbO_3$ cut angle modulation. *IEEE Trans. Ultrason. Ferroelectr. Freq. Control* **69**, 3117–3125 (2022).
11. Liu, P. *et al.* A spurious-free SAW resonator with near-zero TCF using $LiNbO_3$/SiO_2/quartz. *IEEE Electron Device Lett.* **44**, 1796–1799 (2023).
12. Xiao, B. *et al.* Anisotropy-matched LN/quartz heterostructure with inherent spurious mitigation for wideband SAW devices. *IEEE Trans. Microw. Theory Tech.* **73**, 10080–10094 (2025).
13. Zhang, S. *et al.* Surface acoustic wave devices using lithium niobate on silicon carbide. *IEEE Trans. Microw. Theory Tech.* **68**, 3653–3666 (2020).
14. Wen, Z. *et al.* A high power-handling laterally-excited bulk acoustic resonator with scattering vias in double-layer electrodes over +35 dBm. In *Proc. 2024 IEEE Ultrasonics, Ferroelectrics, and Frequency Control Joint Symposium (UFFC-JS)* 1–4 (2024). doi:10.1109/UFFC-JS60046.2024.10793761.
15. Sui, D. *et al.* Miniaturized A1 mode acoustic resonators and filters using inverted T-

shaped electrodes. *IEEE Trans. Microw. Theory Tech.* **73**, 10071–10079 (2025).
16. Fang, X. *et al.* Hybrid integration of dual-mode SAW resonators for high-power and wideband high-frequency filters. *IEEE Trans. Microw. Theory Tech.* **73**, 8490–8499 (2025).
17. Gonzalez-Rodriguez, M. *et al.* Method to measure reflection coefficient under CW high-power signals in SAW resonators. In *Proc. 2021 IEEE International Ultrasonics Symposium (IUS)* 1–4 (IEEE, 2021).
18. van der Wel, P. J., Wunnicke, O., de Bruijn, F. & Strijbos, R. C. Thermal behaviour and reliability of solidly mounted bulk acoustic wave duplexers under high power RF loads. In *Proc. 2009 IEEE International Reliability Physics Symposium (IRPS)* 557–561 (IEEE, 2009).
19. Pan, S., Memon, M. M., Wan, J., Wang, T. & Zhang, W. The influence of pressure on the TCF of AlN-based SAW pressure sensor. *IEEE Sens. J.* **22**, 3097–3104 (2022).
20. Stoney, G. G. & Parsons, C. A. The tension of metallic films deposited by electrolysis. *Proc. R. Soc. Lond. Ser. Contain. Pap. Math. Phys. Character* **82**, 172–175 (1997).
