# Supplementary material for: Suppressing acoustomigration and temperature rise for high-power robust acoustics
Source: Nat Commun. 2026 Apr 22;17:5543. doi: 10.1038/s41467-026-72102-7 (PMC13287746; doi:10.1038/s41467-026-72102-7)
Supplement: Supplementary file 2 — Transparent Peer Review file [file 41467_2026_72102_MOESM2_ESM.pdf]

nature portfolio

Peer Review File

Suppressing Acoustomigration and Temperature Rise for High-Power Robust Acoustics

Corresponding Author: Professor Yansong Yang

Parts of this Peer Review File have been redacted as indicated to remove third-party material.

This file contains all reviewer reports in order by version, followed by all author rebuttals in order by version.

Version 0:

Reviewer comments:

Reviewer #1

(Remarks to the Author)
This manuscript presents a highly impactful and timely study that tackles a long-standing challenge in state-of-the-art piezoelectric acoustic wave devices. While such acoustic wave devices have been intensively investigated for decades, leading to mature systems with well-understood performance in operational frequency, electromechanical coupling, and quality factors, they have been fundamentally constrained by power capacity limitations for a long time. These limitations, arising from stress-induced migration, diffusion, and inadequate thermal management, are becoming increasingly severe with the growing demands of data-intensive workloads and chip miniaturization.
The authors' proposed layered acoustic wave architecture represents a significant conceptual and practical leap forward. The demonstrated results in high-power acoustic wave transduction are not only novel but are supported by comprehensive and high-quality experimental data. The presented performance is exceptional and convincingly benchmarks against the state-of-the-art. Beyond the impressive power capacity enhancement, the manuscript's broader value lies in its methodological contribution. The developed design framework for high-density integration coupled with efficient acoustic energy confinement has the potential to resonate deeply across a wide scientific community, including those working on 3D integration and microscale thermal management for power-demanding systems. It is technically robust and holds considerable promise for both academia and industry. I thoroughly enjoyed reading it and believe it has the potential to attract wide interest and inspire further research in the field.
The manuscript is well-written, and the scientific impact is expected to be high. In my view, it deserves publication in Nature Communications. I only have a few minor points for the authors' consideration:
1. While the theoretical framework in Fig. 2h proposes a strategy for spurious mode suppression, experimental admittance curves in Fig. 4c show spurious responses. Although they do not appear to degrade the target mode's performance, the authors should discuss the nature of these spurious modes and clarify whether they are a fundamental limitation or can be further suppressed in future designs.
2. Given that aluminium is the conventional electrode material due to its cost and acoustic properties, the authors should briefly discuss the rationale for selecting gold over commonly used aluminium electrodes, highlighting the specific benefits in the designing process.
3. To allow for a direct performance comparison, admittance curves for conventional SAW transducers with an identical layout should be included in the Supplementary Information.
4. The key claim that the substrate is not the power-handling bottleneck would be strengthened by supporting evidence from experiments or existing literature.
References and Citations:
a. The formula for kt2 should be properly referenced. The authors should cite its original derivation in the work by Naik et al. (IEEE Trans. Ultrason., Ferroelect., Freq. Contr., 1998, doi: 10.1109/58.646930) where it was first established.
b. To better contextualize the power handling analysis within the current state of the art, the authors could cite several recent high-power BAW and SAW studies. Relevant works include Wen et al. (2024 IEEE UFFC-JS) on power-handling resonators, Fang et al. (IEEE TMTT, 2025) on high-power filters, and Sui et al. (IEEE TMTT, 2025) on miniaturized acoustic resonators. This would strengthen the discussion by acknowledging parallel advancements in the field.
Some possible typos and format issues:
a. Section 2.2, Fifth Paragraph: "efficient electromechanical coupling coefficient" should be "effective electromechanical coupling coefficient".

b. Figure 4 Caption: For terminological accuracy, “Bode Q” and “Bode Qmax” should be written as “Bode-Q” and “Bode-Qmax,” respectively.
c. Figure 5c: The font size of the y-axis labels appears inconsistent with the x-axis labels and should be unified.
d. Methods Section (Electrical Characterization): The terms “Y-parameters” and “S-parameters” should be typeset in italics as “Y-parameters” and “S-parameters”.
e. Supplementary Note #3: The manuscript should specify the method used to determine the “quality factor (Qp)”—whether it is derived from the Bode-Q or the 3-dB bandwidth method—to avoid ambiguity.

Reviewer #2

(Remarks to the Author)
This manuscript studies improvements in power handling, temperature compensation, and Q factor in lithium niobate (LN) on sapphire surface acoustic wave (SAW) resonators via the inclusion of a thick, PECVD Si layer. Overall, the paper is well written and the authors present a highly detailed set of experiments. Notably, the threshold power density is improved by ~14 fold via inclusion of a thick Si layer above the SAW resonator when compared to the thin film LN-on-sapphire SAW resonator without a thick Si top layer, referred to as TF-SAW. The temperature coefficient of frequency (TCF) is also lower and less dispersive than prior LN on substrate SAW resonators from the literature. This is achieved using both theory and finite element modeling to tailor the material stack and thicknesses of each material comprising the SAW.

In the past decade the SAW resonator landscape has been transformed by Murata’s incredibly high-performance SAW (IHP-SAW) resonator technology and its many derivatives. The key concept is to bond a thin, high coupling LN or other piezo layer on a high velocity substrate such as Si, sapphire, or SiC where the velocity mismatch between the piezo layer and the substrate serves to confine the acoustic wave primarily in the piezo layer and prevents bulk radiation of acoustic waves into the substrate. This configuration has resulted in a massive increase in the piezoelectric coupling (K2) and Q for SAW resonators at a given frequency and when high thermal conductivity substrates are used an increase in the power handling. Typically, in these IHP-SAW like approaches the surface of the SAW directly interfaces with air and provides a free boundary condition. The authors present a natural extension of the IHP-SAW approach, by including a high acoustic velocity Si layer above the piezo layer, thereby forming a buried SAW device with high velocity materials both below and above the SAW resonator region. Similar concepts have been previously explored for solidly mounted bulk acoustic wave resonators (see numerated comment below). By including a thick Si layer over the SAW the velocity and Q are increased without degrading the coupling. More importantly, the thermal heat conduction away from the resonator is improved, leading to an increase in the resonator power handling.

While the paper is well written and the experiments are highly detailed, the paper would benefit significantly by comparing the power handling to other works and resonator technologies, not just the benefits when compared to the TF-SAW reported in this paper. The authors also test the resonator power handling near the anti-resonance frequency (fa), and the justification for this in the paper is well supported when testing resonators. However, when testing acoustic filters, which don’t have the same impedance matching challenges observed when testing resonators, the failures often occur near the resonant frequency (fr) of the resonators, as this is where the currents are maximized and typically electro-migration of the metals is the dominant high power failure mechanism. The authors admit as much in the SI, that their approach tests for acousto-migration (not electro-migration) near fa where the currents are suppressed. The results would be much more impactful at the technological level if simple filters were constructed that are matched to 50 ohms for power testing. Filters with as few as 3 resonators would allow a full characterization of the power handling benefits. I enumerate these and other comments for the authors below.

1. The paper would benefit significantly by comparing the power handling to other works and resonator technologies, not just the benefits when compared to the TF-SAW reported in this paper. In particular, how does the power density compare to state-of-the-art membrane and solidly mounted BAW filters? Those typically have much higher power density than SAW resonators.

2. The authors in several places use the phrases “record-high threshold”, “record FOM”, “record-breaking threshold”, and “record power densities” in their paper referring to the results of the LAW resonators. Such statements imply these are the highest performing devices compared to all published works. However, as the reviewer studies the paper, these results are only compared to the TF-SAW also reported in this work and not to all published GHz resonators. The authors should clarify this.

3. In section 2 and Figure 2 the authors portray this work as the first to propose a layer above the resonator (fixed-fixed) to tailor the acoustic energy confinement and improve the thermal dissipation. This technique has been thoroughly explored with some notable contributions made by Texas Instruments in their commercial bulk acoustic wave resonator oscillator research. While the references for this “fixed-fixed” type resonator are many, some examples can be found in the following references,

a. J. Segovia-Fernandez and E. T. . -T. Yen, "Resonant Confiners for Acoustic Loss Mitigation in Bulk Acoustic Wave Resonators," 2023 IEEE 36th International Conference on Micro Electro Mechanical Systems (MEMS), Munich, Germany, 2023, pp. 165-168, doi: 10.1109/MEMS49605.2023.10052545.

b. K. M. Lakin, "Bulk acoustic wave coupled resonator filters," Proceedings of the 2002 IEEE International Frequency Control Symposium and PDA Exhibition (Cat. No.02CH37234), New Orleans, LA, USA, 2002, pp. 8-14, doi:

10.1109/FREQ.2002.1075848.

c. B. Bahr, R. Marathe and D. Weinstein, "Phononic crystals for acoustic confinement in CMOS-MEMS resonators," 2014 IEEE International Frequency Control Symposium (FCS), Taipei, Taiwan, 2014, pp. 1-4, doi: 10.1109/FCS.2014.6859980.

4. The authors test and compare resonator power handling near the anti-resonance frequency (fa). The justification for this is well supported in the paper as this maximizes the resonator absorbed power and temperature rise when testing low impedance resonators in a 50-ohm network. However, when testing acoustic filters, which by design are well-matched to 50-ohms, the failures often occur near the resonant frequency (fr) of the acoustic resonators (as opposed to the anti-resonant frequency) as this is where the currents are maximized and typically electro-migration of the metals is the dominant high power failure mechanism. The authors admit as much in the SI, that their approach tests for acousto-migration (not electro-migration) near fa where the peak electrical currents are suppressed. The results would be much more impactful at the technological level if simple filters were constructed that are matched to 50 ohms for the power testing. Filters with as few as 3 resonators would allow a full characterization of the power handling benefits across frequency. As currently written, the paper reports a 14x increase in power density for resonators near fa and it is entirely unclear what the benefits are of this at the filter level where high power failures occur near fr.

5. While in Fig. 4a the LAW transducer has no observable spurs directly at fa, there are still a large number of spurs between fr and fa that are clearly observable in both the admittance plot in 4a and the Bode Q in 4b. This will cause high passband ripple in any eventual high order filter implementation and clearly causes large oscillations in the Bode-Q. The authors should discuss the implications of these spurs and how they might be eliminated in the SI. The authors should not claim, as is done in 4a, that the response is spurious free. The spurs appear to simply have been shifted away from fa allowing for a higher Q at fa.

6. The authors state that the optimized residual stress for the α-Si is 4.379 MPa. This is a very low value and the authors should place some error bars around this measurement using radius of curvature (ROC). It is also highly unlikely the film stress can be measured with 1 kPa resolution using wafer curvature measurements as the significant digits imply in the listed value.

7. There appears to be an extra legend entry for TF-SAW transducer in Fig. 4a.

Reviewer #3

(Remarks to the Author)
The paper appears to report a modified TC-SAW, with the top SiO2 layer replaced by aSi. The benefit here is that the energy is better confined at the interface. The paper seems to have been written with significant effort in its presentation. However, the authors should provide a fairer comparison between this LSAW technology and the well-established TC-SAW (SiO2 on top of LN/LT) and IHP-SAW (LN/LT-SiO2-carrier) technologies. Despite the authors replacing a thick top SiO2 layer with Si, the reviewer is not convinced that the platform performs better than the conventional technology. A few reasons below:
1) The resonators show really low Q below 500 in the so-called LSAW, while it is well known that the IHP-SAW has Q over 5000, and the TC-SAW has Q over 2000. The new platform is simply not comparable to the existing technologies in commercial products. The 2.5 GHz or so operating frequency for this work is also a range that has been well studied by different technologies. If the authors build filters using the technology, the results might be only moderate compared to existing commercial products.
2) The power handling should not be performed at the resonator level, but at the filter level. In resonators, most signals are reflected and are also affected by the device's impedance. A better comparison method would be to build a filter and compare the power-handling or H2/H3 harmonics of the new technology with those of existing technologies. Currently, the authors seem to be building IHP-SAW/TC-SAW testbeds that are worse than the state of the art and then comparing the LSAW with them. This might not be the best practice.
3) Some of the state-of-the-art comparison tables might miss out important data points. For instance, the current -13 ppm/K TCF is not as good as commercial TC-SAW.
4) The definition of power density here is confusing. The surface area of resonators is not typically the key factor in power handling. Please demonstrate filters and report on the filter area and device performance.
There are still minor technical issues in the work that require further revision. However, those might be less of a concern than the power density testing in the manuscript right now. The reviewer does not believe the paper should be published before a filter demonstration is built and tested at high power levels.

Reviewer #4

(Remarks to the Author)
This manuscript presents a highly valuable acoustic wave transducer design that employs double-sided boundary engineering across mechanical, thermal, and electrical domains. Its most significant contribution is successfully challenging the long-standing paradigm that a top air boundary is irreplaceable for acoustic energy confinement. The work establishes a robust methodology for high-power acoustic wave applications, demonstrating performance superior to other state-of-the-art piezo-on-insulator platforms. The proposed device topology shows clear potential for improved temperature compensation and, owing to the remarkable wave confinement, future high-density integration with 3-D thermal management.
The analysis of failed devices in the supplementary materials is comprehensive and clearly demonstrates the suppression of

acoustomigration. The demonstrated breakdown mechanism in the proposed LAW transducer is also first reported for acoustic transducers, providing new insight into how to improve power density.
For these reasons, I am pleased to recommend publication in Nature Communications after the following minor revisions are addressed:
(1) The spurious responses in acoustic wave transducers would be unacceptable for practical applications, such as interactions between quantum components, where a clean frequency response is highly important for system fidelity and coherence times. The authors should comment on this limitation within their platform and suggest potential pathways to achieve a spurious-free response.
(2) Another important requirement in many applications is the need to accurately set an acoustic wave device's frequency to a predetermined value. Given that the operating frequency increases by ~25% compared to the SAW transducer in Fig. 4a, please clarify the relationship between the operating frequency and the top cladding layer thickness.
(3) To supplement the cross-sectional analysis, top-view images of the failed devices at State C and D should be provided to offer further insight into their mechanical conditions.
(4) To ensure clarity in the EDS analysis, the specific line series used for the elemental data in Table S2 should be clearly indicated.
(5) Terminology (e.g., "Bode-Q") should be standardized for consistency throughout the main text and supplementary information
(6) Please check the equation for the Eyring's model in the Methods section.

Version 1:

Reviewer comments:

Reviewer #1

(Remarks to the Author)
The authors have addressed all of my comments satisfactorily. This is excellent work.
The detailed methodology presented in the Supplementary Information for directly characterizing acoustic transducers under high-power excitation is both clear and highly valuable to the field of piezoelectric acoustics. To the best of my knowledge, no prior work has systematically introduced a framework for direct high-power characterization of acoustic transducers. This study significantly reshapes the community's understanding of acoustic transducer behavior under extreme power input and represents the first work to focus on the fundamental phenomena and physical principles governing transducers in the ultra-high-power regime.

Reviewer #2

(Remarks to the Author)
This manuscript studies improvements in power handling, temperature compensation, and Q factor in lithium niobate (LN) on sapphire surface acoustic wave (SAW) resonators via the inclusion of a thick, PECVD Si layer. The authors have done very thorough and thoughtful work addressing the reviewer comments and as a result have improved the quality and clarity of the paper. There are still a few items remaining that need addressing by the authors. Most important is point #1, which highlights that the authors have only measured the increase in power density for the LAW at a specific impedance that sets the power handling limits of their device at fa. The impact of the paper would be significantly enhanced if the authors would characterize the power handling/power density across a range of static capacitance values that set the maximum power density frequency to different values between fr and fa. As the stress distribution of the acoustic wave, and thus the acousto-migration, surely changes between fr and fa. By measuring the most stressing power density case at only 1 frequency and acoustic stress profile, it is unclear if the LAW improves the power density across the entire bandwidth of the resonator which is vital for all of the cited applications.

1. The authors have made a thorough effort responding to the prior comments about the power handling studies. I appreciate the detailed discussion but some more work is needed here. I am fully convinced that using the large capacitance device with C0 of 1202.6 fF, that the transducer fails near fa in a 50 ohm system, as this is when the largest IDT voltage difference and acoustic stress are imparted to the resonator. I also agree that the power handling in this region is limited by acousto-migration, as the authors have shown highly convincing evidence of this. I also agree that the power handling of a filter is set by the power density of the constituent resonators in the filter and that arraying resonators, etc., can be used to tailor the power handling by altering the power distribution among resonators. But filter topology cannot be used to circumvent the power density of its constituent resonators, and therefore can still be used as a useful tool to study the max. power density of the resonators. The device with C0=1202 fF fails near fa in large part (maybe even entirely) because that is where the resonator impedance results in a large voltage across the IDTs and a large vibratory stress. At other frequencies the resonator is highly mismatched and the test simply cannot couple sufficient acoustic power into the resonator (see Fig. R25c). The authors readily acknowledge as much in Figure R27 and the surrounding discussion. Fig. R27 also shows that the peak stress field distribution changes with frequency based on the impedance matching. It's also normalized and I highly suspect the peak stress field is larger near fr when compared to fa. The authors should show the raw Von Mises stress in their next response letter. Undoubtedly, the stress field distribution in the LAW resonator changes as a function of frequency, and will be different near fr than it is near fa. This is true of all high coupling SAW resonators. My concern with the current work is that the power handling or power density of the LAW device is measured only near fa, and it is claimed the limit at

this frequency is indicative of the power handling or power density across the entire resonator band. I still contend that when well matched, that acoustic resonators tend to fail near fr as this is where the current density and power density are highest!!!! The authors directly show in R27 that when C0 is lower (i.e. the resonator is smaller) the peak power dissipated in a 50 ohm system will move to lower frequency (closer to fr). This will cause the power density to increase. I don't think the authors have adequately tested the failure near fr because their impedance is too low and they cannot couple energy into it at this frequency. There are numerous ways the authors can characterize if their approach improves the power handling/power density across the frequency band of the resonator, rather than just at fa (the data presented by the authors at fa is highly convincing). The authors clearly show in fig. R27 that resonators of multiple sizes (lower C0) could be constructed to move the frequency where the resonator is well matched, allowing the power density to be compared across frequency. It would greatly benefit the impact of the paper for the authors to measure the power density limits of resonators with lower C0 values to obtain the power density enhancements closer to fr.

2. Just above Figure 1, the authors list 4 fundamental physical phenomena that prevent power scaling in SAW, but fail to mention electromigration. Since this is a very common failure mode that has traditionally limited RF SAW devices, I think it should be added here.

3. The fonts in fig. 2h are extremely small and should be increased

4. In the revised manuscript, the authors state that the Au electrode is stiffer than alternative metals such as Cu, Pt, and Ag. However, Au has the lowest Young's modulus of these metals and it is considerably lower than Pt and Cu. The enhanced K2 performance is likely due to the increased mass density. The authors should not state that Au is stiffer.

5. The Bode-Q max values reported in Fig. 4b are highly influenced by the transverse spurious modes. The authors should construct an mBVD model that matches the device admittance and phase and then show a Bode-Q for the equivalent circuit model, as this is more likely to show the true Bode-Q of the resonant mode targeted by the authors. Undoubtedly the Bode-Q is higher for the LAW compared to the other two SAW transducers, but those narrow peak values claimed by the authors are high only due to the spurs.

6. The presentation of Fig. 4h must be improved. It is completely unreadable in its current form.

Reviewer #3

(Remarks to the Author)
The reviewer noticed the authors' significant effort during revision. The paper could be published; however, it would be more convincing if a filter were demonstrated and tested for power-handling or benchmarking purposes. The reviewer is not fully convinced, but thinks the paper is an interesting prototype for the research community and should be shared.

Reviewer #4

(Remarks to the Author)
Thank authors for the detailed responses. While industry efforts typically focus on developing new circuit topologies to enhance high-power handling, this work proposes a bottom-up approach by improving the power-handling capability of the fundamental building block—acoustic transducers. This perspective is both technically sound and highly relevant for practical applications.

Version 2:

Reviewer comments:

Reviewer #2

(Remarks to the Author)
I'd like to thank the authors for carefully considering my prior comments and performing the additional experiments on the smaller C0 devices. In my view, these additional experiments have definitively proven the key claims of the paper. The work is indeed a huge leap forward in both the understanding of power handling limits in IHP-type SAW devices and in their ultimate power handling performance using the LAW structure reported by the authors. The paper in its current form is both of high scientific quality and of high importance to the field of acoustic resonators and filters.

Open Access This Peer Review File is licensed under a Creative Commons Attribution 4.0 International License, which permits use, sharing, adaptation, distribution and reproduction in any medium or format, as long as you give appropriate credit to the original author(s) and the source, provide a link to the Creative Commons license, and indicate if changes were made.
In cases where reviewers are anonymous, credit should be given to 'Anonymous Referee' and the source.
The images or other third party material in this Peer Review File are included in the article's Creative Commons license, unless indicated otherwise in a credit line to the material. If material is not included in the article's Creative Commons license and your intended use is not permitted by statutory regulation or exceeds the permitted use, you will need to obtain permission directly from the copyright holder.
To view a copy of this license, visit https://creativecommons.org/licenses/by/4.0/

Peer Review File

Suppressing Acoustomigration and Temperature Rise for High-power Robust Acoustics

Response Letter: Suppressing Acoustomigration and Temperature Rise for High-power Robust Acoustics

Fangsheng Qian, Shuhan Chen, Wei Wei, Jiashuai Xu, Kai Yang, Junyan Zheng, Zijun Ren, Xingyu Liu, and Yansong Yang*

Summary of response

Dear Reviewers,

We sincerely thank all four reviewers for their time and invaluable feedback. Their insightful comments and constructive suggestions have been thoroughly addressed in the revised manuscript and Supplementary Information, significantly enhancing the quality and clarity of our work. We appreciate the reviewers' positive assessment of the work's novelty, experimental rigor, and potential impact, as exemplified by the following comments:

"The demonstrated results in high-power acoustic wave transduction are not only novel but are supported by comprehensive and high-quality experimental data; The manuscript is well-written, and the scientific impact is expected to be high. In my view, it deserves publication in Nature Communications." (Reviewer #1)

"Overall, the paper is well written and the authors present a highly detailed set of experiments; The authors also test the resonator power handling near the anti-resonance frequency (f_a), and the justification for this in the paper is well supported when testing resonators." (Reviewer #2)

"Its most significant contribution is successfully challenging the long-standing paradigm that a top air boundary is irreplaceable for acoustic energy confinement; The demonstrated breakdown mechanism in the proposed LAW transducer is also first reported for acoustic transducers, providing new insight into how to improve power density; For these reasons, I am pleased to recommend publication in Nature Communications after the following minor revisions are addressed" (Reviewer #4)

Before providing point-by-point responses, we wish to underscore the core novelty and significance of our work in this executive summary:

Building on the proposed method for directly characterizing single transducers, this work starts from fundamental principles and reveals the failure mechanism of acoustic transducers under high power density. Moving from failure mechanisms to solutions, this work introduces a first-of-its-kind, double-sided boundary engineering platform that fundamentally addresses the high-power handling challenge in acoustic transducers. The key innovation is not a simple material substitution, but a synergistic electro-thermo-mechanical co-design: By leveraging a quasi-infinite α-Si medium above the baseline structure, we achieved a ~13-fold improvement in threshold power density. This layer actively redistributes mechanical stress within the resonator cavity and transitions heat dissipation from diffusion-dominated to propagation-dominated transport, as experimentally validated by infrared thermography. This strategy moves beyond prior substrate-focused thermal management in SAW technology, demonstrating for the first

time how simultaneous top-boundary stress modulation and thermal management can unlock performance limits. The platform also achieves a lower and less dispersive temperature coefficient of frequency (TCF) than prior $LiNbO_3$-based transducers.

Our research is driven by the fundamental power-handling limits of the transducer as an individual component, a critical metric for both traditional RF elements and emerging applications in acousto-optics, acoustofluidics, and precise field manipulation. While filter-level performance is ultimately important for specific applications, it is a system-level metric confounded by design-specific power division and layout, making it unsuitable for benchmarking the inherent performance of core transducer technology across platforms. Therefore, our standardized component-level evaluation provides the most essential and unbiased comparison of technological advancement.

In direct response to the editor's and reviewers' requests, we have made the following notable improvements to the manuscript:

1. The motivation for measuring the transducer directly: To investigate the fundamental failure mechanisms, a direct characterization method for the transducer is proposed. If necessary, the single transducer can be tested at any specified frequency. Measuring a filter composed of multiple transducers cannot assess its power tolerance from the perspective of individual components.
2. Theoretical analysis and suppression of bulk wave modes via novel mechanical bandgap engineering.
3. Expanded measurement and simulation data to highlight the novelty of mechanical stress and thermal boundary engineering for performance gains.
4. Enhanced discussion and data on parasitic plate mode suppression and temperature compensation.
5. Well-controlled filter-level experiments to substantiate our rationale for transducer-level power testing.
6. Broadened experimental comparison to include multiple state-of-the-art platforms and commercial products under identical test conditions, improving the robustness of our testing framework.

We thank the editors and reviewers for their invaluable input, which has guided a comprehensive strengthening of the manuscript. All concerns and suggestions have been addressed in the revised text and in the point-by-point responses below. A marked-up version is provided for ease of review. We believe these revisions have clarified the impact of our work, which we hope will benefit the broad readership of *Nature Communications* and may help inspire future advances in high-power acoustics.

REVIEWER COMMENTS

****

Reviewer #1 (Remarks to the Author):

****

This manuscript presents a highly impactful and timely study that tackles a long-standing challenge in state-of-the-art piezoelectric acoustic wave devices. While such acoustic wave devices have been intensively investigated for decades, leading to mature systems with well-understood performance in operational frequency, electromechanical coupling, and quality factors, they have been fundamentally constrained by power capacity limitations for a long time. These limitations, arising from stress-induced migration, diffusion, and inadequate thermal management, are becoming increasingly severe with the growing demands of data-intensive workloads and chip miniaturization.

The authors' proposed layered acoustic wave architecture represents a significant conceptual and practical leap forward. The demonstrated results in high-power acoustic wave transduction are not only novel but are supported by comprehensive and high-quality experimental data. The presented performance is exceptional and convincingly benchmarks against the state-of-the-art. Beyond the impressive power capacity enhancement, the manuscript's broader value lies in its methodological contribution. The developed design framework for high-density integration coupled with efficient acoustic energy confinement has the potential to resonate deeply across a wide scientific community, including those working on 3D integration and microscale thermal management for power-demanding systems. It is technically robust and holds considerable promise for both academia and industry. I thoroughly enjoyed reading it and believe it has the potential to attract wide interest and inspire further research in the field.

The manuscript is well-written, and the scientific impact is expected to be high. In my view, it deserves publication in *Nature Communications.* I only have a few minor points for the authors' consideration:

AUTHOR RESPONSE:

We are particularly encouraged by the extremely positive feedback regarding the quality and impact of our manuscript.

1) While the theoretical framework in Fig. 2h proposes a strategy for spurious mode suppression, experimental admittance curves in Fig. 4c show spurious responses. Although they do not appear to degrade the target mode's performance, the authors should discuss the nature of these spurious

modes and clarify whether they are a fundamental limitation or can be further suppressed in future designs.

AUTHOR RESPONSE:

We agree completely that suppressing spurious responses is an additional and important consideration to further consolidate this work and thanks to this comment, we have now included a comprehensive analysis, which we summarize below and have now included in detail in the revised Supplementary Information, Section 6.

The spurious modes in Fig. 4c are not a fundamental limitation of the LAW concept. They originate from two distinct, well-understood sources: (i) transversal waveguide modes in the aperture (lateral) direction, and (ii) higher-order bulk waves scattered at vertical acoustic boundaries. These are categorically different from the parasitic plate modes (e.g., A0, S0, and SH1 mode in the baseline TF-SAW or SiO_2-overcoated TF-SAW devices) that our LAW architecture was designed to suppress, as demonstrated in Figs. 2f-2g and 4a in the main text.

Critically, both types of spurious responses are addressable through established and novel design strategies. For transversal modes, proven techniques on thin-film piezoelectric platforms, including piston mode designs [1], tilted electrodes [2, 3], and slowness curve modulation [4], can effectively flatten the lateral velocity profile to suppress them.

As for higher-order bulk waves, we have developed a suppression strategy based on mechanical bandgap engineering intrinsic to the layered architecture. More specifically, the LAW platform can be conceptualized as a vertical stack of different acoustic waveguides, as shown in Fig. R1a. Given dispersion relationship for each mode, the targeted SH0 mode is well-

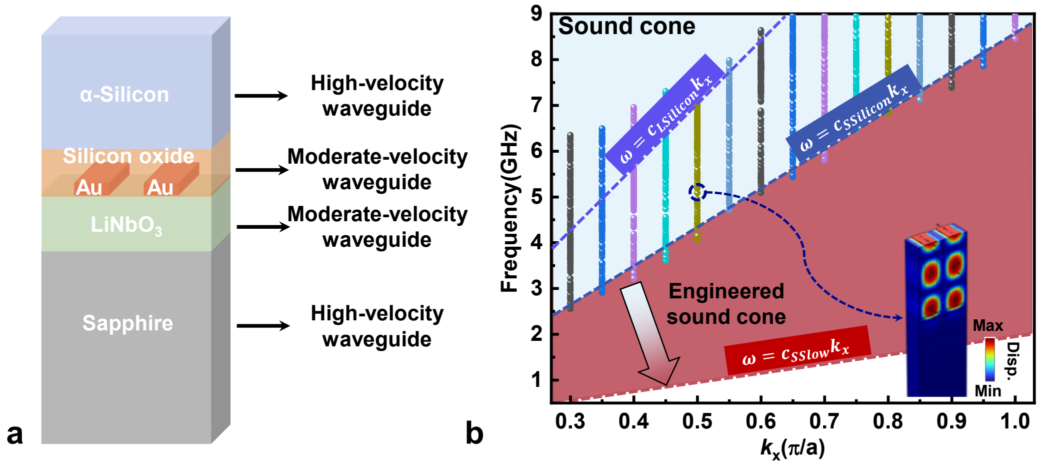

Figure. R1. **a**, Schematic of the initial vertically stacked LAW transducer and its associated acoustic waveguides. **b**, Dispersion relation of an optimized LAW transducer design for suppressing bulk vibrational modes. The light-blue region denotes the initial sound cone for free-propagating bulk modes. The red-extended sound cone represents an engineered waveguide region with sufficiently low acoustic velocity, which redirects bulk modes away from the sandwiched resonator cavity.

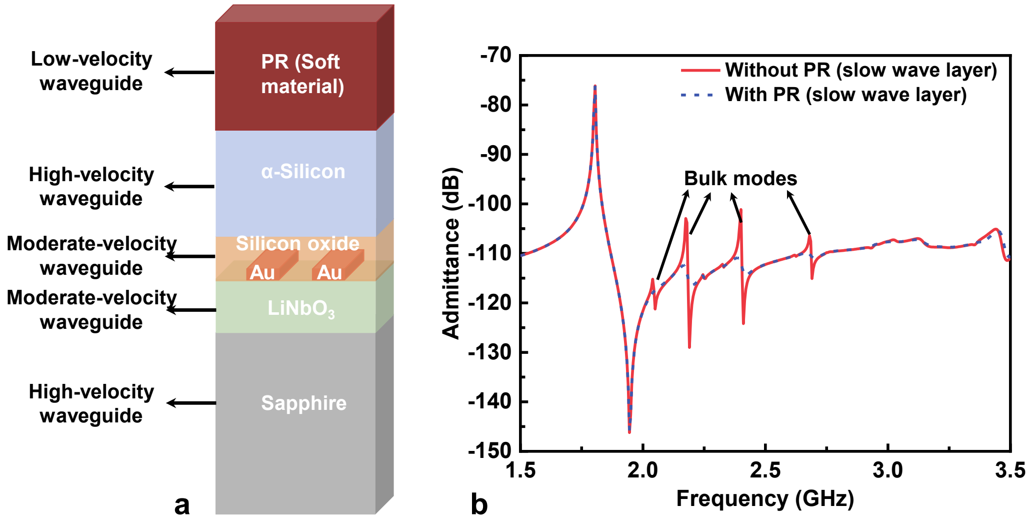

Figure. R2. **a**, Schematic of the optimized LAW transducer design for bulk mode suppression, showcasing the associated acoustic waveguides. **b**, Admittance curves for the LAW transducer design with and without a slow-wave photoresist (PR) waveguide.

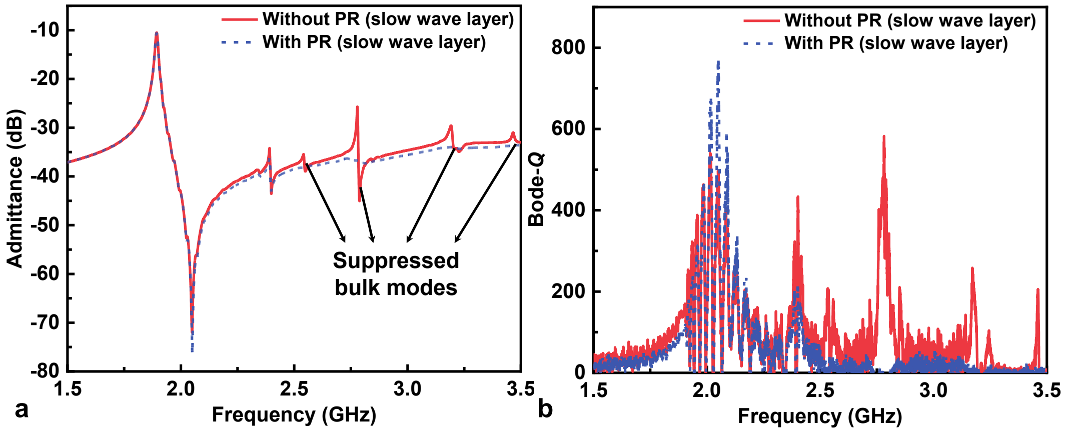

Figure. R3. **a**, Measured admittance curves and **b**, extracted Bode-Q characteristics from one-port measurement, comparing designs with and without the slow-wave PR waveguide. The suppression of bulk modes through engineered mechanical boundaries increased the Bode-Q_{max} of the targeted mode from 541 to 776.

confined within the $LiNbO_3$ cavity, while other higher-order bulk waves propagate towards the upper SiO_2 and α-Si layers. To suppress those unwanted spurious waves, we engineer the system's sound cone in $\vec{k}$-space to broaden the available phase-velocity range for parasitic modes, as illustrated in Fig. R1b.

In this engineered sound cone, initial bulk modes vibrating in the upper SiO_2 and α-Si waveguides propagate into a slower wave layer, which sits on the top of the LAW structure (Fig. R2a). At the same time, the targeted mode is confined individually beneath the SiO_2 and α-Si waveguides. Here, photoresist (PR) is selected as the uppermost slow wave medium. Finite-element simulations confirm the principle: adding a PR waveguide significantly suppresses the bulk-wave spurs without degrading the targeted mode's effective electromechanical coupling (k_t^2) and Q (Fig. R2b).

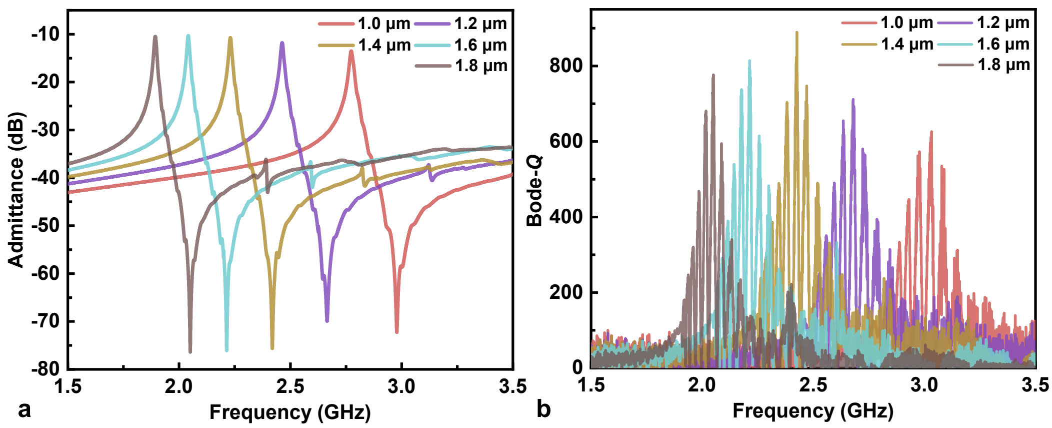

Figure. R4. Wavelength dependence of the **(a)**, measured admittance curves and **(b)**, extracted Bode-Q characteristics for engineered LAW transducers with the slow-wave PR waveguide.

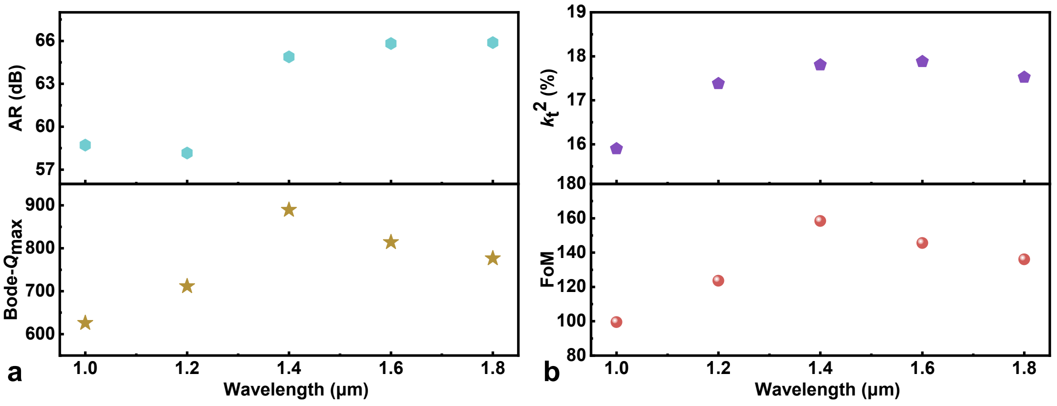

Figure. R5. Key metrics extracted across wavelengths for the engineered LAW transducer with the slow-wave PR waveguide, including **a**, admittance ratio (AR) and Bode-Q_{max}, as well as **b**, k_t^2 and figure of merit (FoM).

We experimentally validated this concept by coating a 5.3-μm-thick AZ® 4620 PR layer on the LAW platform (with probing pads exposed for electrical contact). The measured admittance shows effective suppression of the bulk modes (Fig. R3). Crucially, this approach improves the device's key performance: the maximum Bode-Q of the target mode increased from 541 to 776, while spurious responses were reduced to negligible levels (Fig. R3b). Wavelength-dependent measurements further confirm the robustness of this mechanical boundary condition design, showing consistent improvements in admittance ratio (AR) and Bode-Q (Fig. R4). A full summary of performance metrics, including AR, Bode-Q_{max}, k_t^2 and the figure of merit (FoM = Bode-$Q \times k_t^2$), across wavelengths further confirms the significant advance and potential of mechanical boundary engineering (Fig. R5).

Importantly, this spurious-mode suppression strategy does not compromise the fundamental power-handling advantage of the LAW architecture. The maximum von Mises stress at the critical metal/$LiNbO_3$ interface remains unchanged with the addition of the PR waveguide, indicating a negligible impact on power durability. To maintain the logical flow of the main manuscript, the detailed discussion of this bulk-wave suppression strategy is provided

in the revised Supplementary Information, Section 6. This work demonstrates that the observed spurious responses are not a fundamental limitation but are addressable through further engineering of the acoustic boundaries, underscoring the advanced design flexibility of the LAW platform.

[1] Hashimoto, K. et al. "Revisiting piston mode design for radio frequency surface acoustic wave resonators." In Proc. *2022 IEEE MTT-S International Conference on Microwave Acoustics and Mechanics* (IC-MAM) 60–63 (IEEE, 2022).
[2] Xu, H. et al. "SAW filters on $LiNbO_3$/SiC heterostructure for 5G n77 and n78 band spplications." *IEEE Trans. Ultrason. Ferroelectr. Freq. Control* 70, 1157–1169 (2023).
[3] Qian, F., Zheng, J., Xu, J. & Yang, Y. "Heterogeneous interface-Enhanced thin-film SAW devices using Lithium Niobate on Si." *IEEE Microw. Wirel. Technol. Lett.* 35, 123–126 (2025).
[4] Shen, J. et al. Suppressed transverse mode generation in TF-SAW resonators based on $LiTaO_3$/Sapphire. *IEEE Electron Device Lett.* 45, 2241–2244 (2024).

ACTION TAKEN:

We have now included detailed discussions and added new experimental data on the spurious modes' suppression in the revised Supplementary Information, Section 6.

2. Given that aluminum is the conventional electrode material due to its cost and acoustic properties, the authors should briefly discuss the rationale for selecting gold over commonly used aluminium electrodes, highlighting the specific benefits in the designing process.

AUTHOR RESPONSE:

The authors agree that aluminum (Al) represents a common choice for interdigital electrodes in acoustic wave devices, owing to its low cost, high electrical conductivity, and suitable mass-loading properties. However, for the specific layered architecture presented in this work, the design requirements necessitate a different material consideration.

A pivotal feature of our design is the inclusion of a subsequent SiO_2 layer, which is critical for achieving stress relief and a substantial k_t^2 via a negative $\Delta(V)$, as detailed in the boundary analysis within the Supplementary Information, Section 1. For this reason, the as-deposited electrode material should experience a thermal oxidation process, which will inevitably oxidize the electrode material and increase the ohmic loss. As such, gold (Au) was considered as the electrode material benefiting from its chemical inertness. Beyond stability, the choice of stiffer and denser electrode material like gold provides additional benefits for the transducer's k_t^2 performance. The interdigital transducer (IDT) structure inherently shifts the mechanical stress field from the piezoelectric layer towards the electrode layer. A stiffer electrode like Au couples

more effectively with this stress field and the applied electric field, leading to a higher achievable k_t^2. To attain a comparable electromechanical coupling with Al, a significantly greater electrode thickness would be required due to its different mass-loading characteristics. Such a thick Al layer would introduce considerable challenges in the subsequent lithographic lift-off fabrication process. Consequently, gold not only fulfills the thermal stability requirement but also enables a high-performance, fabrication-friendly device structure with an optimized electrode profile.

In summary, while Al is a standard material for conventional designs, the specific thermal processing and electromechanical objectives of our LAW architecture make Au the functionally superior and necessary choice for this work.

ACTION TAKEN:

We have added a brief discussion in the revised manuscript outlining the rationale for selecting gold electrodes: *"Beyond stability, the choice of stiffer and denser electrode material like Au provides additional benefits for the transducer's* k_t^2 *performance. The IDT structure inherently shifts the mechanical stress field from the piezoelectric layer towards the electrode layer. A stiffer electrode like Au couples more effectively with this stress field and the applied electric field, leading to a higher achievable* k_t^2*."*

3. To allow for a direct performance comparison, admittance curves for conventional SAW transducers with an identical layout should be included in the Supplementary Information.

AUTHOR RESPONSE:

We thank the reviewer for the constructive suggestion to include a direct performance comparison. As requested, we have now provided the admittance curves and extracted Bode-*Q* characteristics of TF-SAW transducers in the revised Supplementary Information, Section 7. These transducers, fabricated from the identical mask layout, are the very same structure onto which the SiO_2-overcoated and subsequent α-Si layer were deposited to form the SiO_2-overcoated and LAW transducers, respectively. Presenting this data fulfills the request for a conventional SAW reference and, more importantly, provides the definitive benchmark that allows readers to directly attribute the performance enhancements demonstrated in the main text solely to the subsequent architectural modifications

ACTION TAKEN:

We added the comparison data about the TF-SAW transducers' performance in the revised Supplementary Information, Section 7.

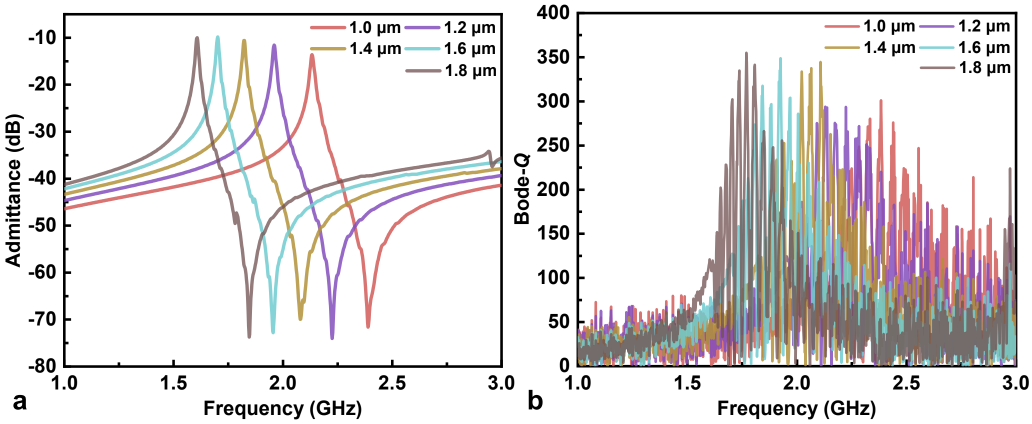

Figure. R6. Wavelength dependence of the **a**, measured admittance curves and **b**, extracted Bode-*Q* characteristics for the TF-SAW transducer before depositing the upper layers.

4. The key claim that the substrate is not the power-handling bottleneck would be strengthened by supporting evidence from experiments or existing literature.

AUTHOR RESPONSE:

We greatly appreciate the reviewer's insightful comment regarding the need to support the claim that the substrate is not the primary bottleneck for power handling. In response to the reviewer's comments, we conducted a dedicated comparative study to quantitatively evaluate the impact of substrate thermal management on ultimate power-handling limits. We selected sapphire and silicon carbide (SiC) as the supporting substrates and fabricated TF-SAW transducers with identical layouts on two substrates for their significantly different thermal conductivities, as summarized in Table R1.

Table R1. Material properties overview between $LiNbO_3$, Sapphire, and SiC

Materials	Thermal conductivity (W/m/K)	Acoustic impedance (kg/m²/s)	Fundamental shear velocity (m/s)
$LiNbO_3$	4.2	1.73e+7	3674 (±4%)
Sapphire	32.5	2.4e+7	6045
SiC	370	2.29e+7	7126

Following the power test framework established in the main text, we began with small-signal *S*-parameters to calculate the RF power reflection, transmission, and dissipation at each

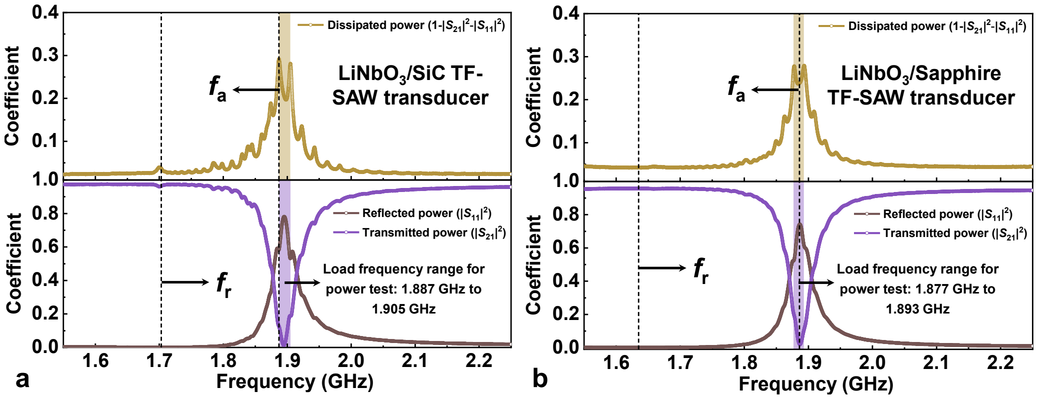

Figure. R7. **a**, Dissipated, reflected, and transmitted power coefficients as a function of driving frequency for a 2-port thin-film $LiNbO_3$/SiC TF-SAW transducer operating at a load power of $P_{incident}$ = -15 dBm. **b**, Dissipated, reflected, and transmitted power coefficients as a function of driving frequencies for a 2-port thin-film $LiNbO_3$/Sapphire TF-SAW transducer operating at a load power of $P_{incident}$ = -15 dBm. Both transducers share an identical wavelength design to ensure equitable high-power comparison under similar loading frequencies. The resonant frequency (f_r) and anti-resonant frequency (f_a) are marked by the dashed lines in each plot. The loading frequency ranges for the two transducers under high power tests are also indicated.

frequency, providing an estimation of which frequency will become hottest and represent the weakest failing point (Fig. R7). We acknowledge potential port impedance mismatch at the single-transducer level and therefore define the injected power as the actual power delivered to the device (incident minus reflected power); the reflection coefficients for both devices in the test band were identical, ensuring a fair comparison.

The results reveal a critical insight. Fig. R8 summarizes the measured steady-state temperature rise versus injected power density. As expected, the DUT on high-thermal-conductivity SiC exhibited far superior heat spreading, with an operating temperature approximately 16 °C lower than its sapphire-based counterpart at comparable power levels. However, despite this substantial thermal advantage, both devices failed at very similar injected power densities. The difference in their failure thresholds, identified from the inflection points in the ΔT-vs-injected power scatter plots, is only 0.78 dB.

Post-failure admittance measurements confirmed irreversible degradation in both cases, as detailed in Fig. R9. To explain why improved heat dissipation did not translate to a proportionally higher power-handling limit, we performed finite-element analysis of the von Mises stress distribution, as seen in Fig. R10. The simulation reveals that, while the stress peaks at the IDT/$LiNbO_3$ interface in both structures, the stress magnitude within the $LiNbO_3$ cavity is actually higher for the device on the stiffer SiC substrate at the identical injected power (Fig. R11). This is because SiC's higher acoustic impedance reflects more energy back into the resonator cavity, intensifying the mechanical stress that drives acoustomigration.

Therefore, this controlled experiment provides direct evidence that while substrate engineering is effective for thermal management, the ultimate power-handling bottleneck is

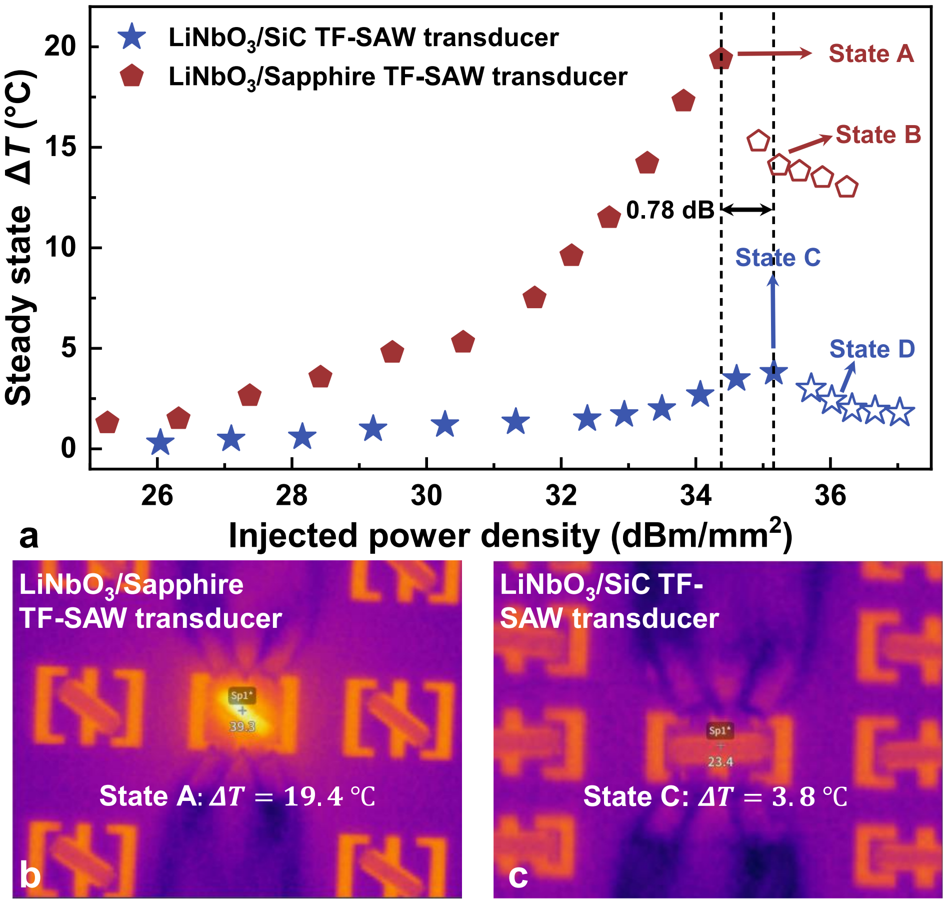

Figure. R8. **a**, Steady-state temperature rise (ΔT) as a function of injected power density, measured by thermal imaging for devices on $LiNbO_3$/SiC and $LiNbO_3$/Sapphire platforms. **(b, c)**, Representative thermal images of the $LiNbO_3$/Sapphire DUT **(b)** and $LiNbO_3$/SiC DUT **(c)** under specific testing conditions (power level and driving frequency). All measurements were conducted at an ambient temperature of ~20 °C.

dictated by mechanical stress concentration at the IDT/$LiNbO_3$ interface. A substrate change alone cannot alleviate this root cause and may even exacerbate it. This finding strongly supports our claim: a more effective strategy is to engineer the top-side boundary conditions of the resonator cavity. Our proposed LAW architecture directly implements this principle by redesigning the upper acoustic boundary to redistribute and mitigate the peak mechanical stress, thereby achieving a fundamental improvement in power handling that transcends the limitations of substrate optimization alone.

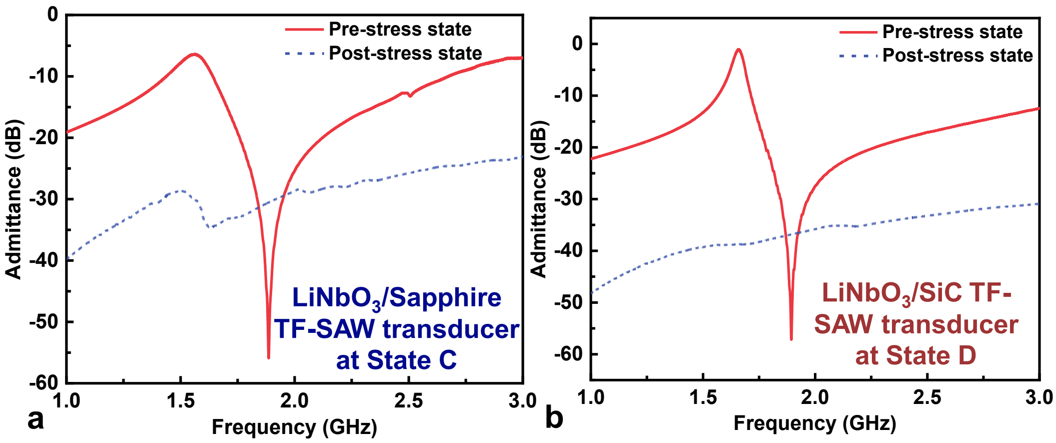

Figure. R9. **a**, A comparison of admittance curves for the $LiNbO_3$/Sapphire TF-SAW transducer between the pre-power test state and post-power test state. **b**, A comparison of admittance curves for the $LiNbO_3$/ SiC TF-SAW transducer between the pre-power test state and post-power test state. All measurements were performed at a load power of P_{incident} = -15 dBm.

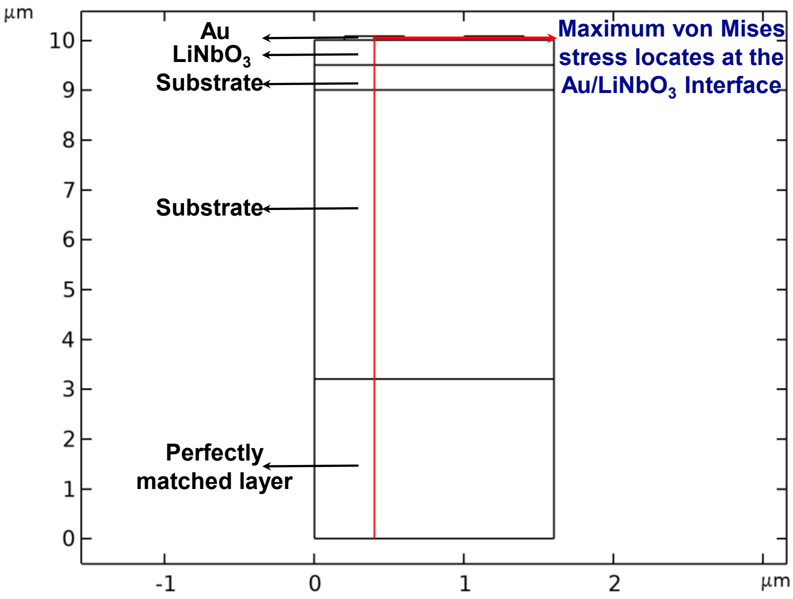

Figure. R10. Method for extracting the von Mises stress along the depth direction, spanning from the top surface of the interdigital layer down to the substrate.

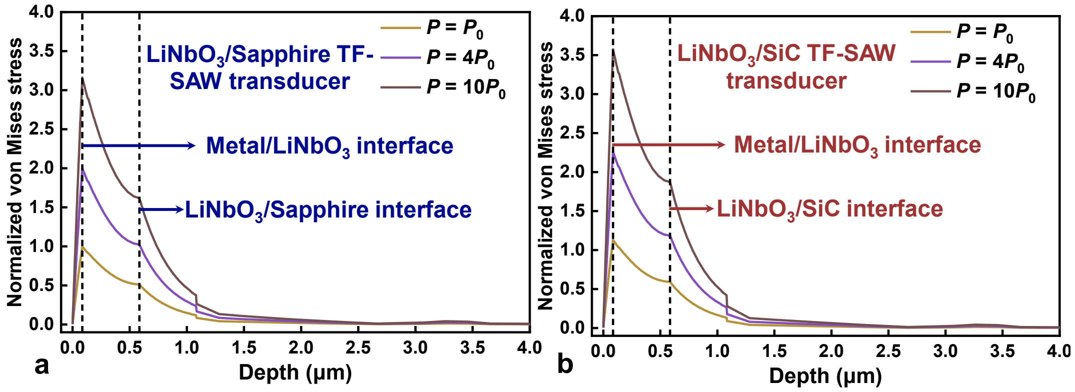

Figure. R11. Depth profiles of the extracted von Mises stress for the **(a)** $LiNbO_3$/Sapphire and **(b)** the $LiNbO_3$/SiC TF-SAW devices. The metal/$LiNbO_3$ and $LiNbO_3$/substrate interfaces are marked by dashed lines. All normalized depth profiles of the von Mises stress in **(a)** and **(b)** are presented relative to the stress distribution obtained in the $LiNbO_3$/sapphire device under an input power of P_0.

References and Citations:

a. The formula for k_t^2 should be properly referenced. The authors should cite its original derivation in the work by Naik et al. (IEEE Trans. Ultrason., Ferroelect., Freq. Contr., 1998, doi: 10.1109/58.646930) where it was first established.

AUTHOR RESPONSE:

We thank the reviewer for pointing this out and for the very careful reading to improve our manuscript. And we have updated the citation in the revised manuscript accordingly.

b. To better contextualize the power handling analysis within the current state of the art, the authors could cite several recent high-power BAW and SAW studies. Relevant works include Wen et al. (2024 IEEE UFFC-JS) on power-handling resonators, Fang et al. (IEEE TMTT, 2025) on high-power filters, and Sui et al. (IEEE TMTT, 2025) on miniaturized acoustic resonators. This would strengthen the discussion by acknowledging parallel advancements in the field.

AUTHOR RESPONSE:

We thank the reviewer for this thoughtful suggestion and have added a brief introduction to contextualize the power handling analysis.

ACTION TAKEN:

We have updated the citations in the revised Supplementary Information, Section 14, as suggested. Please kindly check them.

Some possible typos and format issues:

a. Section 2.2, Fifth Paragraph: “efficient electromechanical coupling coefficient” should be “effective electromechanical coupling coefficient”.

b. Figure 4 Caption: For terminological accuracy, “Bode Q” and “Bode Qmax” should be written as “Bode-Q” and “Bode-Qmax,” respectively.

c. Figure 5c: The font size of the y-axis labels appears inconsistent with the x-axis labels and should be unified.

d. Methods Section (Electrical Characterization): The terms “Y-parameters” and “S-parameters” should be typeset in italics as “*Y*-parameters” and “*S*-parameters”.

e. Supplementary Note #3: The manuscript should specify the method used to determine the “quality factor (Q_p)”—whether it is derived from the Bode-*Q* or the 3-dB bandwidth method—to avoid ambiguity.

AUTHOR RESPONSE:

We thank the reviewer for this keen observation. We acknowledge these typos and format issues. We thank you for bringing this to our attention. The manuscript has been revised and carefully checked accordingly.

ACTION TAKEN:

Section 2.2: *“Additional key performance metrics, including quality factor (Bode-Q_{max}), k_t^2, and figure of merit (Bode-Q_{max} × k_t^2), are also summarized in Figs. 4d-4e.”*

Figure 4 Caption: *“Measured **a**, admittance and **b**, corresponding Bode-Q curves of transducers in conventional TF-SAW, SiO_2-overcoated TF-SAW, and LAW configurations. **c**, Admittance curves of LAW transducers as a function of wavelength. Extracted metrics include **d**, admittance ratio (AR) and Bode-Q_{max} in the passband, …”*

Figure 5c: The font size is now unified.

Methods Section (Electrical Characterization): *“The admittance responses (Y-parameters) were subsequently calculated from the measured S-parameters without applying any extra de-embedding step.”*

Supplementary Note #3: *“As the temperature decreases from 25 °C to 5 °C, the 3-dB quality factor (Q_p) at parallel frequency (f_p) increases sharply from 98 to 314, marking a turnover point in dominant loss mechanisms.”*

****

Reviewer #2 (Remarks to the Author):

****

This manuscript studies improvements in power handling, temperature compensation, and Q factor in lithium niobate (LN) on sapphire surface acoustic wave (SAW) resonators via the inclusion of a thick, PECVD Si layer. Overall, the paper is well written and the authors present a highly detailed set of experiments. Notably, the threshold power density is improved by ~14 fold via inclusion of a thick Si layer above the SAW resonator when compared to the thin film LN-on-sapphire SAW resonator without a thick Si top layer, referred to as TF-SAW. The temperature coefficient of frequency (TCF) is also lower and less dispersive than prior LN on substrate SAW resonators from the literature. This is achieved using both theory and finite element modeling to tailor the material stack and thicknesses of each material comprising the SAW.

In the past decade the SAW resonator landscape has been transformed by Murata's incredibly high-performance SAW (IHP-SAW) resonator technology and its many derivatives. The key concept is to bond a thin, high coupling LN or other piezo layer on a high velocity substrate such as Si, sapphire, or SiC where the velocity mismatch between the piezo layer and the substrate serves to confine the acoustic wave primarily in the piezo layer and prevents bulk radiation of acoustic waves into the substrate. This configuration has resulted in a massive increase in the piezoelectric coupling (K^2) and Q for SAW resonators at a given frequency and when high thermal conductivity substrates are used an increase in the power handling. Typically, in these IHP-SAW like approaches the surface of the SAW directly interfaces with air and provides a free boundary condition. The authors present a natural extension of the IHP-SAW approach, by including a high acoustic velocity Si layer above the piezo layer, thereby forming a buried SAW device with high velocity materials both below and above the SAW resonator region. Similar concepts have been previously explored for solidly mounted bulk acoustic wave resonators (see numerated comment below). By including a thick Si layer over the SAW the velocity and Q are increased without degrading the coupling. More importantly, the thermal heat conduction away from the resonator is improved, leading to an increase in the resonator power handling.

While the paper is well written and the experiments are highly detailed, the paper would benefit significantly by comparing the power handling to other works and resonator technologies, not just the benefits when compared to the TF-SAW reported in this paper. The authors also test the resonator power handling near the anti-resonance frequency (f_a), and the justification for this in the paper is well supported when testing resonators. However, when testing acoustic filters, which don't have the same impedance matching challenges observed when testing resonators, the failures often occur

near the resonant frequency (f_r) of the resonators, as this is where the currents are maximized and typically electro-migration of the metals is the dominant high power failure mechanism. The authors admit as much in the SI, that their approach tests for acousto-migration (not electro-migration) near fa where the currents are suppressed. The results would be much more impactful at the technological level if simple filters were constructed that are matched to 50 ohms for power testing. Filters with as few as 3 resonators would allow a full characterization of the power handling benefits. I enumerate these and other comments for the authors below.

AUTHOR RESPONSE:

We thank the referee for the careful reading and insightful comments on our manuscript.

We have introduced a new design paradigm that co-optimizes materials and boundaries in multi-physics domains, not merely for confinement by a simple substitution of materials, but for holistic performance transformation. Indeed, it is a co-design achievement that strategically optimizes the mechanical boundary conditions to redistribute mechanical stress within the resonator cavity and implement a thickened top boundary layer whose thermal conductivity approaches that of the bulk material for effective thermal management (experimentally validated by our infrared thermography).

Regarding the accurate assessment of power handling capability, to investigate the fundamental failure mechanisms, a direct characterization method for the transducer is proposed. If necessary, the single transducer can be tested at any specified frequency. Measuring a filter composed of multiple transducers cannot assess its power tolerance from the perspective of individual components. In the filtering application, the failure frequency point varies across topologies. We think that testing at the filter level is neither the only nor the best practice for evaluating the fundamental architectural advancement presented in this work. Below, we explain our rationale, grounded in the study's motivation and supported by new controlled experiments. We also did filter-level measurements to support our argument. We note that comparing transducer power handling across different filter topologies is inherently unfair, and it is also impractical for academic studies to replicate proprietary, product-level topologies used in industry for fair comparison.

We fully agree with the reviewer's technical point that electromigration at f_r is another critical failure mode for acoustic wave devices. As suggested, we have followed the suggestion to extend our transducer-level assessment at f_r to comprehensively investigate the interplay between acoustomigration and electromigration.

We provide more detailed physical explanations in the following responses to further support that this work represents a significant conceptual and practical advance beyond incremental modifications to existing technology.

1. The paper would benefit significantly by comparing the power handling to other works and resonator technologies, not just the benefits when compared to the TF-SAW reported in this paper. In particular, how does the power density compare to state-of-the-art membrane and solidly mounted BAW filters? Those typically have much higher power density than SAW resonators.

AUTHOR RESPONSE:

We thank the reviewer for the suggestion to compare our work with other technologies, including bulk acoustic wave (BAW) devices. We agree that situating our contribution within the broader landscape is important.

A fundamental prerequisite for a meaningful comparison is a benchmarking method that isolates architectural innovation. Power handling measured at the filter level, however, reflects a system-level property influenced by numerous design-specific factors, such as cascading resonators, power-routing layout, matching networks, and test conditions. These variables can obscure the intrinsic improvement attributable solely to the core resonator technology. For instance, Dong et al., have experimentally shown that a squared array-based topology enhances total power handling by 8.8-dB at the filter level under the same effective resonator area and technology, simply by distributing power across multiple resonators without improving the intrinsic power density thresholds of individual devices [1].

Complementing this, we also performed a well-controlled, high-power evaluation using filters built from identical constituent IHP-SAW resonators in an identical physical layout, varying only the electrical connection sequence and the presence of encapsulation. Fig. R12a presents the microscope image of the unpackaged filter layout, featuring specifically designed input/output terminals and three integrated capacitors. Fig. R12b shows the optical image of the same filter after packaging, mounted on an evaluation board with SMA connectors. To clarify our measurement: ***forward connection*** denotes the configuration where incident power first arrives at the filter's input terminal; ***reverse connection*** for the opposite case; only the

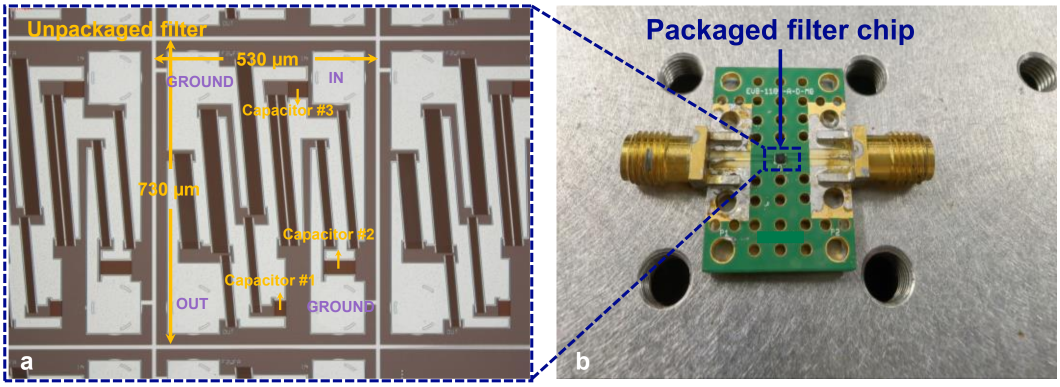

Figure. R12. **a**, Microscope image of a filter before packaging, indicating the input, output, and ground terminals. **b**, Optical image of the packaged filter (after the packaging process of the filter shown in **(a)**) mounted on an evaluation board with SMA connections.

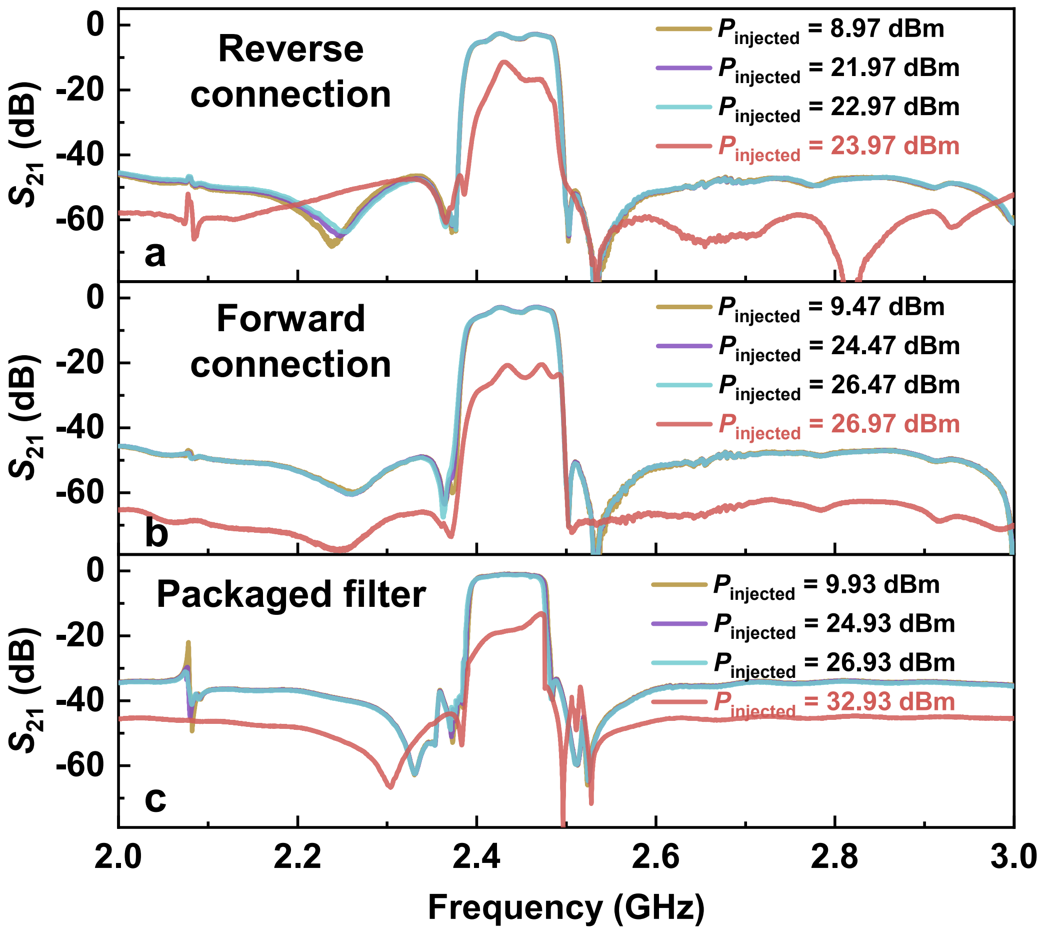

Figure. R13. Comparison of S_{21} responses measured under high-power test conditions across different electrical connection configurations of IHP-SAW filters: **(a)** reverse-connected and unpackaged, **(b)** forward-connected and unpackaged, and **(c)** forward-connected and packaged.

forward configuration was tested for the packaged filter. Fig. R13 summarizes the S_{21} responses under increasing injected power for the unpackaged filter (forward/reverse connections) and the packaged filter. First, while all were stable up to 22.97 dBm, their failure points diverged sharply: 23.97 dBm (reverse, unpackaged), 26.97 dBm (forward, unpackaged), and 32.93 dBm (packaged). Fig. R14, plotting the minimum insertion loss versus injected power, clearly shows these distinct bottlenecks, with a maximum difference of 9.42 dB — even though all filters use identical resonators.

We further investigated via high-power continuous-wave tests on the forward-connected, unpackaged filter at different loading frequencies (Fig. R15). First, thermal images of the forward-connected filter indicate elevated dissipation at passband edges (2.3942 GHz, 2.4842 GHz) compared to the center (2.4515 GHz), with the spatial temperature profile, and thus the internal power distribution, shifting markedly with frequency. This directly visualizes the design-specific and frequency-dependent power flow within the composite acoustic-electromagnetic system. Subsequently, post-test microscopic inspection (Fig. R16) revealed that the locations and failure modes of the damaged resonators varied for each test frequency.

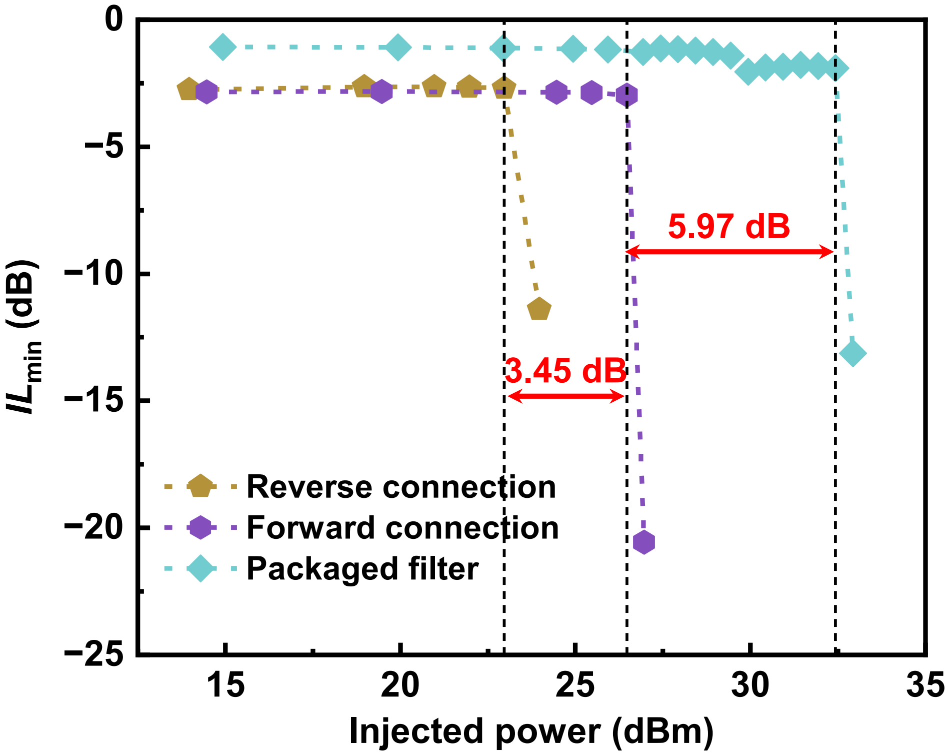

Figure. R14. Measured minimum value of insertion loss (*IL*) under high-power test conditions across different electrical connection configurations of IHP-SAW filters.

Taken together, this combination of thermal, electrical, and mechanical evidence solidifies our conclusion: ***the point of failure in a filter is not predetermined by the resonator alone but is a dynamic outcome of system-level power routing.*** Therefore, assessing power handling at the filter level yields a metric that is intrinsically confounded by design-specific power division and layout. It follows that such a system-level result is misleading and cannot serve as a direct benchmark for the inherent performance of the constituent resonator technology across different acoustic wave platforms. These experimental results highlight that filter-to-filter comparisons are often more reflective of layout and system design than of the resonator architecture itself. As such, to isolate and evaluate performance advancements from architectural innovations, we employ transducer-level benchmarking using an active-area-normalized power density, providing a more direct and accurate assessment of acoustic boundary engineering (as wide-spread in the literature).

Regarding the comparison with BAW technology, we acknowledge that bulk acoustic wave devices generally feature better power durability than surface acoustic wave resonators, benefiting from lower electrode resistance and favorable stress profiles in the thickness-extensional (TE) mode. However, this TE mode is a double-edged sword: while beneficial for power handling, it confines acoustic energy vertically, which inherently limits its use in applications that rely on propagating acoustic waves—such as traveling-wave-based sensing, microfluidics, or on-chip signal processing where in-plane coupling is essential. Moreover, BAW fabrication typically involves multi-layer processing, making it less compatible with

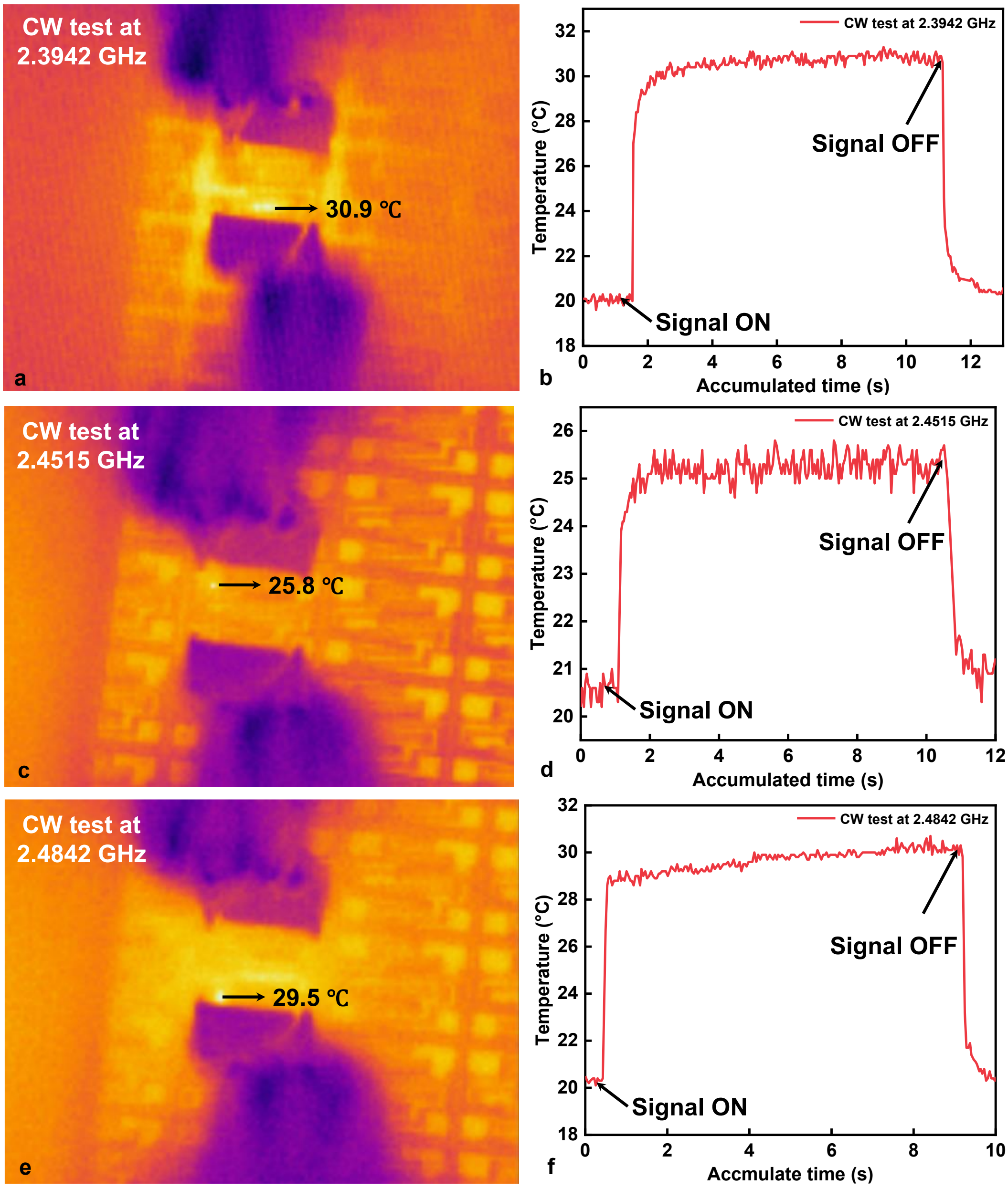

Figure. R15. Thermal characterization of the unpackaged, forward-connected IHP-SAW filters under high-power continuous wave (CW) tests at different loading frequencies. Each pair of panels shows the extracted thermal image (left) and the corresponding temperature versus accumulated time plot (right): (**a, b**) 2.3942 GHz (left edge of the passband), (**c, d**) 2.4515 GHz (center of the passband), and (**e, f**) 2.4842 GHz (right edge of the passband).

straightforward planar integration compared to the lithographically simpler SAW platform [2]. In contrast, SAW devices excel in on-chip integrability, lower fabrication cost, and natural coupling with other physical fields (e.g., optical, mechanical, and fluidic systems), as widely demonstrated in emerging hybrid systems [3-5]. Thus, rather than seeking to outperform BAW in all high-power scenarios, our work aims to set out a route to fundamentally advance the power-handling limits of SAW-based platforms where surface-wave propagation, planar integrability, and multi-domain coupling are defining advantages.

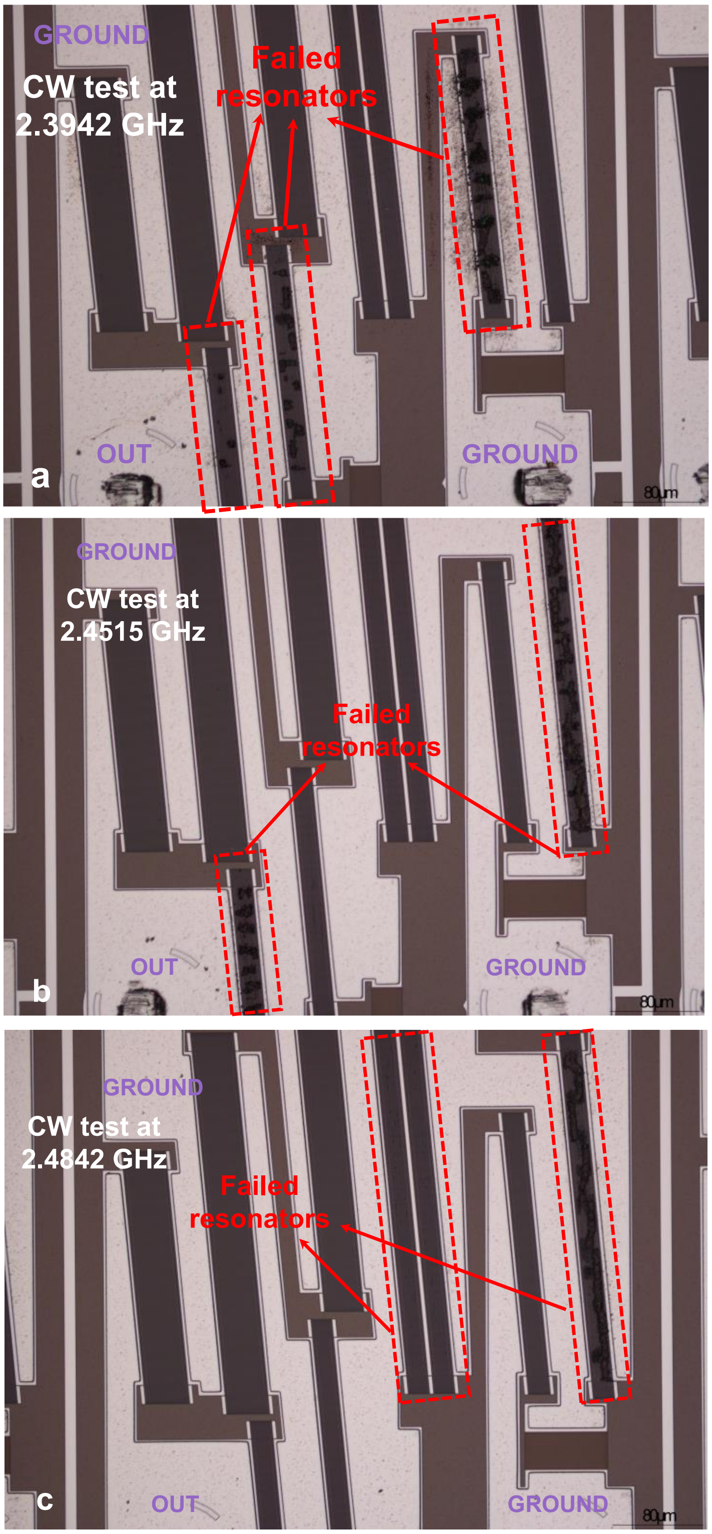

Figure. R16. Post-characterization optical microscope images of the unpackaged IHP-SAW filters (forward-connected), corresponding to the filters after being subjected to the high-power CW tests at various frequencies detailed in Figure R15.

To directly address the reviewer's comment, we have expanded our experimental comparison to include multiple state-of-the-art platforms under identical testing conditions,

including X-$LiNbO_3$/SiC TF-SAW, X-$LiNbO_3$/Sapphire TF-SAW, SiO_2/bulk 128°Y-$LiNbO_3$ TC-SAW, and our α-Si/SiO_2/X-$LiNbO_3$/Sapphire LAW architectures. The detailed experiments are as follows:

We conducted a dedicated comparative study to quantitatively evaluate the impact of ***substrate thermal management*** on ultimate power-handling limits. We selected sapphire and SiC as the supporting substrates and fabricated TF-SAW transducers with identical layouts on two substrates for their significantly different thermal conductivities, as summarized in Table R2.

Table R2. Material properties overview between $LiNbO_3$, Sapphire, and SiC

**Materials**	**Thermal conductivity (W/m/K)**	**Acoustic impedance ($kg/m^2/s$)**	**Fundamental shear velocity (m/s)**
$LiNbO_3$	4.2	1.73e+7	3674 (±4%)
Sapphire	32.5	2.4e+7	6045
SiC	370	2.29e+7	7126

Following the power test framework established in the main text, we began with small-signal *S*-parameters to calculate the RF power reflection, transmission, and dissipation at each frequency, providing an estimation of which frequency will become hottest and represent the weakest failing point (Fig. R17). We acknowledge potential port impedance mismatch at the single-transducer level and therefore define the injected power as the actual power delivered to the device (incident minus reflected power); the reflection coefficients for both devices in the

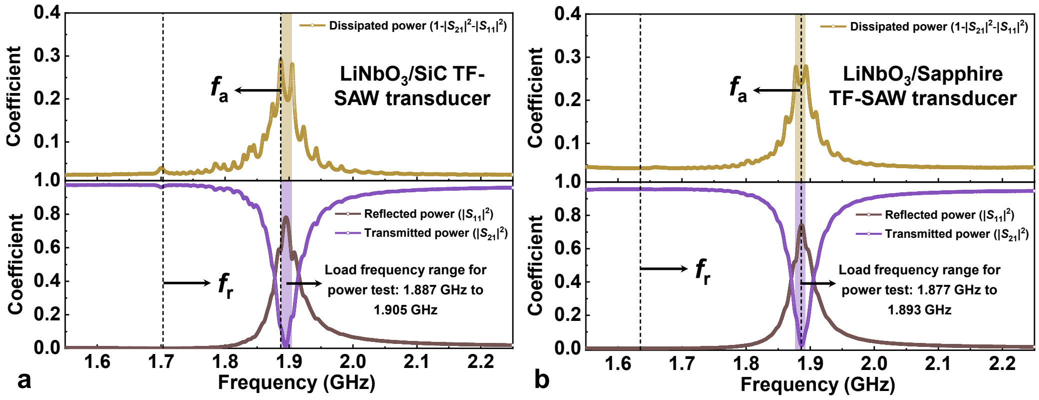

Figure. R17. **a**, Dissipated, reflected, and transmitted power coefficients as a function of driving frequency for a 2-port thin-film $LiNbO_3$/SiC TF-SAW transducer operating at a load power of $P_{incident}$ = -15 dBm. **b**, Dissipated, reflected, and transmitted power coefficients as a function of driving frequencies for a 2-port thin-film $LiNbO_3$/Sapphire TF-SAW transducer operating at a load power of $P_{incident}$ = -15 dBm. Both transducers share an identical wavelength design to ensure equitable high-power comparison under similar loading frequencies. The resonant frequency (f_r) and anti-resonant frequency (f_a) are marked by the dashed lines in each plot. The loading frequency ranges for the two transducers under high power tests are also indicated.

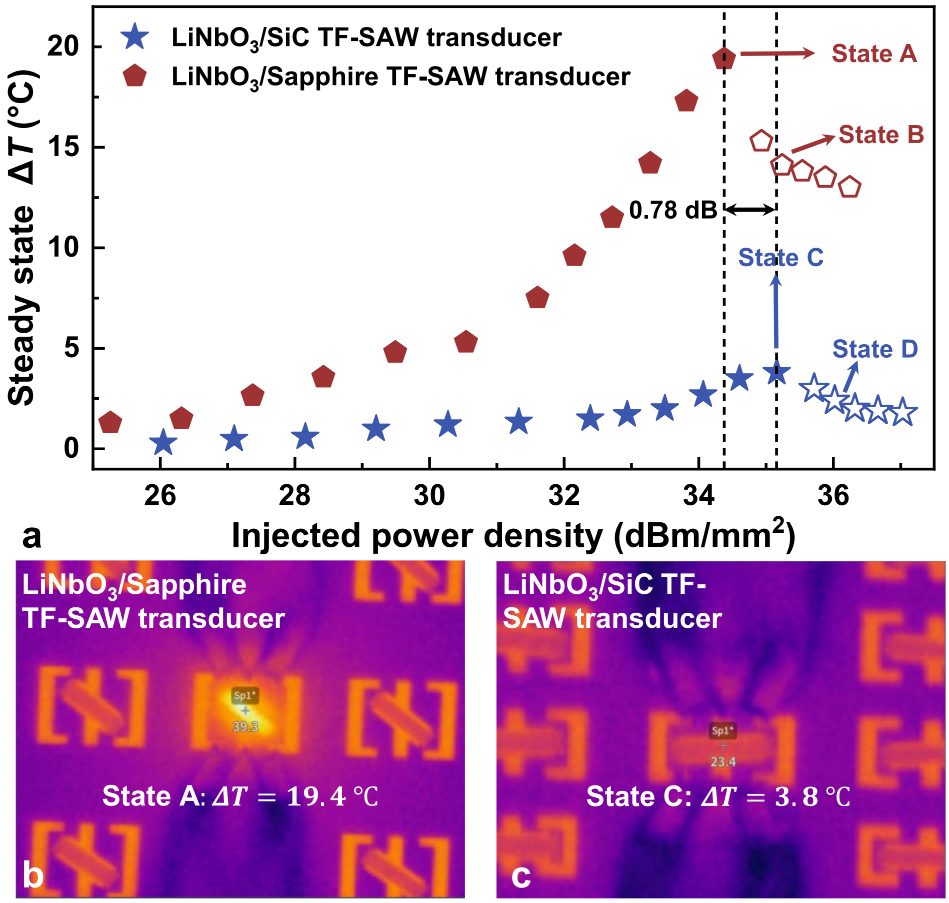

Figure. R18. **a**, Steady-state temperature rise (ΔT) as a function of injected power density, measured by thermal imaging for devices on $LiNbO_3$/SiC and $LiNbO_3$/Sapphire platforms. **(b, c)**, Representative thermal images of the $LiNbO_3$/Sapphire DUT **(b)** and $LiNbO_3$/SiC DUT **(c)** under specific testing conditions (power level and driving frequency). All measurements were conducted at an ambient temperature of ~20 °C.

test band were identical, ensuring a fair comparison.

The results reveal a critical insight. Fig. R18 summarizes the measured steady-state temperature rise versus injected power density. As expected, the DUT on high-thermal-conductivity SiC exhibited far superior heat spreading, with an operating temperature approximately 16 °C lower than its sapphire-based counterpart at comparable power levels. However, despite this substantial thermal advantage, both devices failed at very similar injected power densities. The difference in their failure thresholds, identified from the inflection points in the ΔT-vs-injected power scatter plots, is only 0.78 dB. Post-failure admittance measurements confirmed irreversible degradation in both cases, as detailed in Fig. R19.

In addition, we performed a direct power durability comparison with an industry-standard commercial TC-SAW device (from a top-tier TC-SAW company) using our established protocol. This comparison serves to evaluate the impact of top boundary engineering and thereby highlights the advances achieved by our mechanical stress engineering innovations. Strictly speaking, a power test aiming to probe the intrinsic failure point of an acoustic device

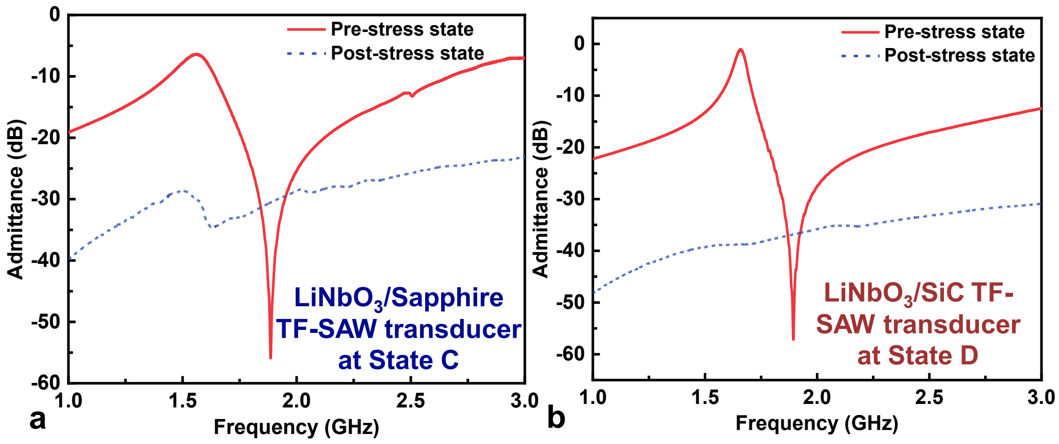

Figure. R19. **a**, A comparison of admittance curves for the $LiNbO_3$/Sapphire TF-SAW transducer between the pre-power test state and post-power test state. **b**, A comparison of admittance curves for the $LiNbO_3$/ SiC TF-SAW transducer between the pre-power test state and post-power test state. All measurements were performed at a load power of P_{incident} = -15 dBm.

should target the frequency of its peak power dissipation. Nevertheless, to ensure a fair comparison across platforms, we deliberately selected a frequency range with identical power dissipation for testing the TC-SAW device, as shown in Fig. R20. Key performance characteristics of the TC-SAW DUT are also included. Consistent with our previous methodology, the injected power is defined as the power delivered to the DUT (incident power minus reflected power). The power density is calculated using this value over the entire rectangular active area, encompassing the IDTs, reflectors, and bus lines.

Fig. R20b plots the steady-state temperature rise versus injected power density. At an injected power density of 32.82 dBm/mm^2, the TC-SAW device temperature rose to 144.4 °C. This contrasts directly with our TF-SAW (12.8 °C at 33.22 dBm/mm^2) and LAW (4.6 °C at 32.94 dBm/mm^2) platforms, highlighting its poor thermal management rooted in bulk $LiNbO_3$ and unoptimized thermal boundaries. Due to the upper detection limit (200 °C) of our IR camera, temperature measurements entered a "dead zone" for injected power densities between 33.87 and 37.76 dBm/mm^2. We therefore paused the test at 35.92 dBm/mm^2 (State A) — already above the TF-SAW transducer's threshold—to measure admittance characteristics (Fig. R21a). This revealed permanent frequency shifts ($\Delta f_r \approx$ 2.2 MHz, $\Delta f_a \approx$ 3.88 MHz). This shift points to a change in the acoustic boundary conditions, as purely electrical degradation would not cause such a frequency drift. The device ultimately failed at 37.76 dBm/mm^2 (State B), marked by a sudden temperature collapse from >200 °C to 26 °C and confirmed by degraded admittance (Fig. R21b). We thus define 37.76 dBm/mm^2 as the TC-SAW's power density threshold under this testing condition. Although the IDT in the TC-SAW architecture is likewise covered by a 1.9 µm-thick temperature-compensating SiO_2 top layer, it nevertheless fails at a power density of 37.76 dBm/mm^2. This represents a significant 7.85 dB difference

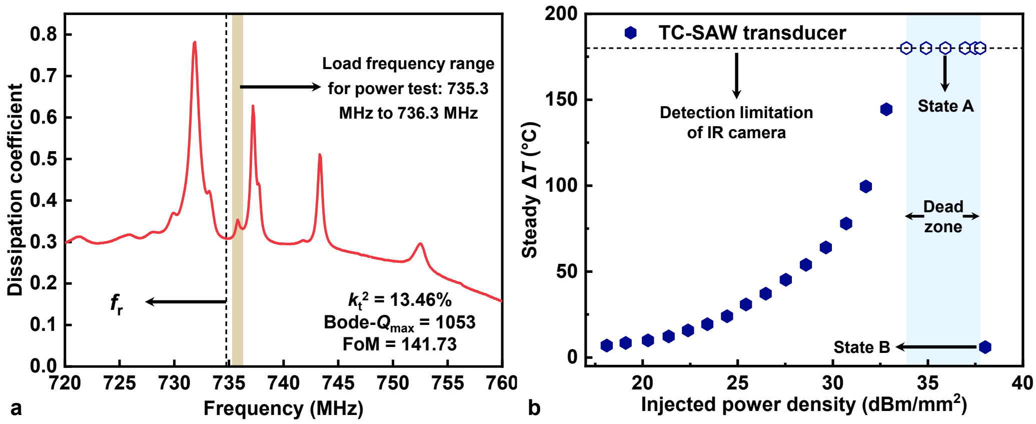

Figure. R20. **a**, Calculated power dissipation as a function of loading frequencies. The frequency range for the high-power test is indicated by the highlighted region. **b**, experimentally measured steady-state temperature rise of the TC-SAW DUT as a function of injected power density. The fitted first-order TCF at f_r and f_a are also indicated. Temperature data between 33.87 dBm/mm^2 to 37.76 dBm/mm^2 exceed the IR camera's upper detection limit, marking a measurement dead zone.

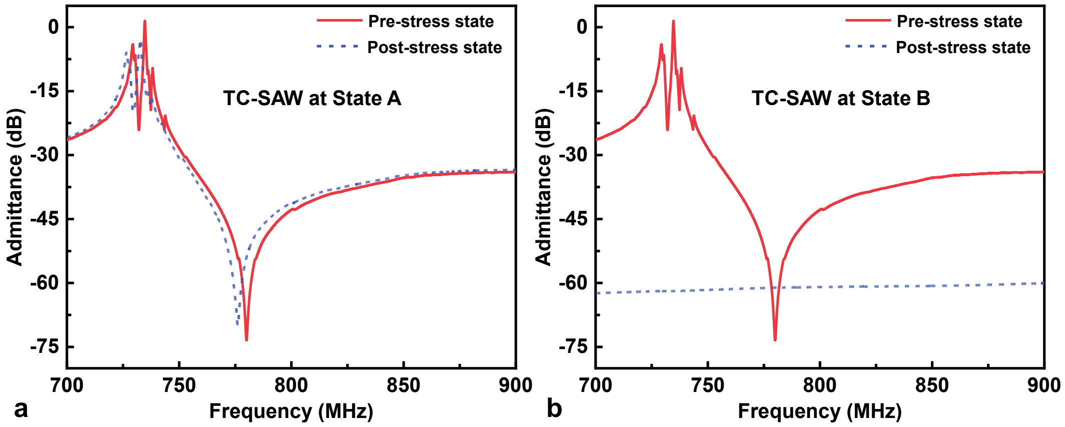

Figure. R21. [In our setup] **a**, Admittance comparison of the TC-SAW DUT before the power test and after subjection to an injected power density of 35.92 dBm/mm^2 (State A). **b**, Admittance comparison before the power test and after the final failure at State B (37.76 dBm/mm^2).

compared to the 45.61 dBm/mm^2 threshold achieved by our proposed LAW platform.

To fundamentally understand the power-handling limits across the tested acoustic platforms, we performed a finite-element analysis of the von Mises stress distribution. For each platform, the mechanical stress profile along the depth (from the top surface to the bottom boundary) was evaluated at the frequency corresponding to its weakest failure point, with all geometrical features held identical in the simulations. In Fig. R22, all normalized depth profiles of von Mises stress are presented relative to the maximum stress value obtained in the $LiNbO_3$/sapphire TF-SAW transducer at a reference input power, P_0. The simulations reveal a key insight: while the stress universally peaks at the IDT/$LiNbO_3$ interface, its magnitude within the $LiNbO_3$ cavity differs significantly among the structures. Since the mechanical stress is proportional to the square root of input power (as validated in Fig. R22), these differences

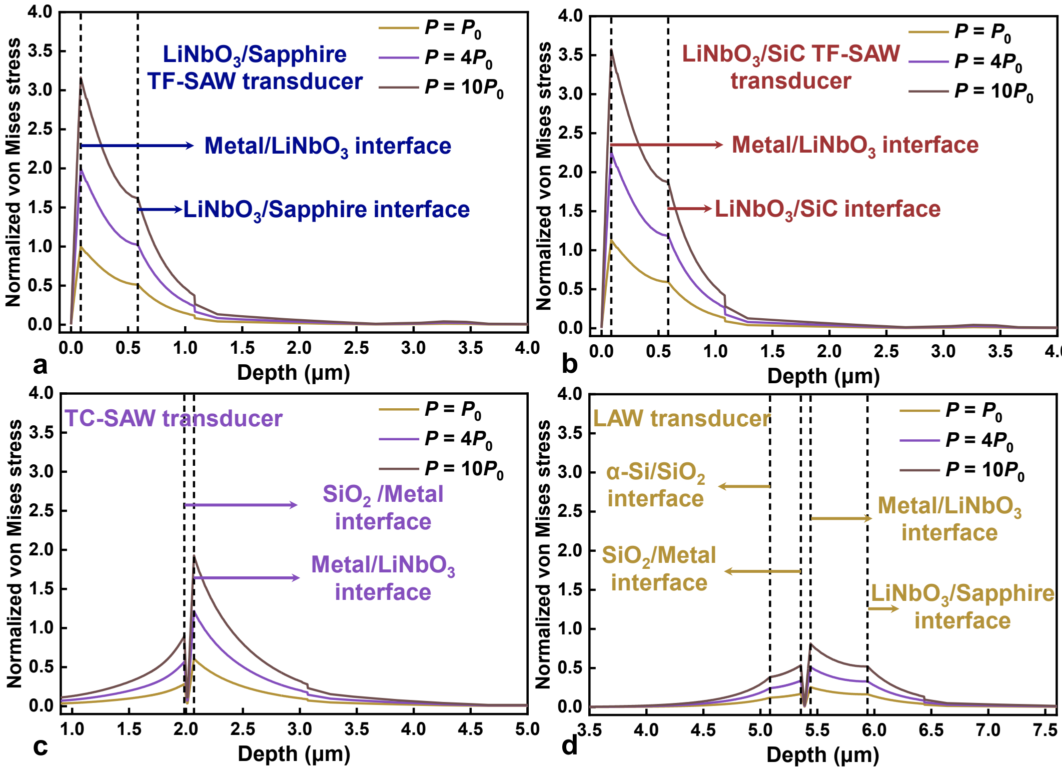

Figure. R22. Depth profiles of the extracted von Mises stress for **(a)** baseline $LiNbO_3$/Sapphire TF-SAW DUT, **(b)** $LiNbO_3$/SiC TF-SAW DUT, **(c)** SiO_2/$LiNbO_3$ TC-SAW DUT, and **(d)** our proposed α-Si/SiO_2/$LiNbO_3$/Sapphire LAW DUTs. All mechanical stress profiles are normalized to the maximum stress in the baseline TF-SAW (a) at reference power, P_0. Key interfaces are marked by dashed lines.

directly correlate with power-handling capability. Notably, the device on the stiffer SiC substrate exhibits higher mechanical stress within the cavity at the same injected power. This is because SiC's higher acoustic impedance reflects more energy back into the resonator cavity, intensifying the mechanical stress that drives acoustomigration. For the TC-SAW transducer, despite having a 1.9 μm-thick SiO_2 top layer, the mechanical stress is reduced to approximately 3/5 of that in the baseline $LiNbO_3$/sapphire TF-SAW transducer at the reference input power, P_0. Most importantly, our proposed LAW platform achieves the minimum stress distribution along the depth direction, with a value of only 1/4 of the baseline stress at P_0. Additionally, the mechanical stress within the resonator cavity is more uniformly distributed compared to the other three structures. Notably, even under an input power ten times higher ($10P_0$), the maximum stress in the LAW platform (~0.8×) remains lower than that in the baseline $LiNbO_3$/sapphire TF-SAW transducer at the reference power P_0.

In summary, under identical test conditions using injected power density (incident minus reflected), our LAW architecture exhibits a 6.09-fold (7.85 dB) improvement over the commercial TC-SAW (with 1.9 μm top SiO_2 layer) , an 11.09-fold (10.45 dB) improvement over $LiNbO_3$/SiC TF-SAW, and a 12.73-fold (11.23 dB) improvement over $LiNbO_3$/sapphire TF-SAW transducers. Combining experimental data with this simulation analysis confirms that

our LAW architecture represents more than a simple substitution of materials. It is a co-design achievement that strategically optimizes the mechanical boundary conditions to maximize energy confinement, redistribute stress within the resonator, and implement a thickened top boundary layer whose thermal conductivity approaches that of bulk material for effective thermal management. This holistic approach enables a significant power-handling enhancement within the SAW technology family, pushing its performance closer to the realm of more specialized platforms while fully preserving its inherent application advantages.

[1] Wang, W. et al. "Ultra-high power handling capability bulk acoustic wave filters using square array topology.*" J. Micromechanics Microengineering* **35**, 085009 (2025).

[2] Bolgar, A. N. et al. "Quantum regime of a wwo-dimensional phonon cavity." *Phys. Rev. Lett.* **120**, 223603 (2018).

[3] Lin, Q. et al. Optical multi-beam steering and communication using integrated acousto-optics arrays. *Nat. Commun.* **16**, 4501 (2025).

[4] Devendran, C., Collins, D. J., Ai, Y. & Neild, A. Huygens-Fresnel acoustic interference and the development of robust time-averaged patterns from traveling surface acoustic waves. *Phys. Rev. Lett.* **118**, 154501 (2017).

[5] Hackett, L. et al. Towards single-chip radiofrequency signal processing via acoustoelectric electron–phonon interactions. *Nat. Commun.* **12**, 2769 (2021).

ACTION TAKEN:

We added new experimental data on the power tests at the filter level (revised Supplementary Information, Section 8) and provided detailed discussions about the rationale for the power tests at the transducer level in the revised manuscript.

2. The authors in several places use the phrases "record-high threshold", "record FOM", "record-breaking threshold", and "record power densities" in their paper referring to the results of the LAW resonators. Such statements imply these are the highest performing devices compared to all published works. However, as the reviewer studies the paper, these results are only compared to the TF-SAW also reported in this work and not to all published GHz resonators. The authors should clarify this.

AUTHOR RESPONSE:

We sincerely thank the reviewer for highlighting this important issue regarding the language used to describe our results. We agree that phrases such as "record-high" and "record-breaking" were imprecise and could be misinterpreted as a claim against all published technologies, rather than a specific comparison within the context of our study. We apologize

for this lack of clarity. In direct response to this comment, we have carefully revised the manuscript to eliminate all hyperbolic or absolute comparative language. Specifically, we have replaced terms like “record-high threshold”, “record FOM,” and “record power densities” with more accurate and objective descriptions.

All relevant sections, including the abstract, results, and conclusion, have been updated accordingly. We are grateful for the reviewer’s constructive criticism, which has helped us present our work with greater precision. The revised manuscript now provides a clearer and more appropriate assessment of the performance advancements enabled by our layered acoustic waveguide architecture.

ACTION TAKEN:

All claims of “record-high” or “record-breaking” performance have been removed. The results are now presented using objective and qualified language throughout the manuscript to accurately reflect the specific comparisons made within the study.

3. In section 2 and Figure 2 the authors portray this work as the first to propose a layer above the resonator (fixed-fixed) to tailor the acoustic energy confinement and improve the thermal dissipation. This technique has been thoroughly explored with some notable contributions made by Texas Instruments in their commercial bulk acoustic wave resonator oscillator research. While the references for this “fixed-fixed” type resonator are many, some examples can be found in the following references,

a. J. Segovia-Fernandez and E. T. . -T. Yen, "Resonant Confiners for Acoustic Loss Mitigation in Bulk Acoustic Wave Resonators," 2023 IEEE 36th International Conference on Micro Electro Mechanical Systems (MEMS), Munich, Germany, 2023, pp. 165-168, doi: 10.1109/MEMS49605.2023.10052545.

b. K. M. Lakin, "Bulk acoustic wave coupled resonator filters," Proceedings of the 2002 IEEE International Frequency Control Symposium and PDA Exhibition (Cat. No.02CH37234), New Orleans, LA, USA, 2002, pp. 8-14, doi: 10.1109/FREQ.2002.1075848.

c. B. Bahr, R. Marathe and D. Weinstein, "Phononic crystals for acoustic confinement in CMOS-MEMS resonators," 2014 IEEE International Frequency Control Symposium (FCS), Taipei, Taiwan, 2014, pp. 1-4, doi: 10.1109/FCS.2014.6859980.

AUTHOR RESPONSE:

We thank the reviewer for these insightful comments and for providing relevant references regarding “fixed-fixed” boundary conditions in acoustic devices. We agree that the use of additional layers above a resonator cavity has been demonstrated for different purposes, as

exemplified by the cited works. We acknowledge these excellent contributions. Below, we address the specific contributions of these references and clarify the distinct architectural innovation of our present work on layered acoustic wave transducers.

First, the referenced works primarily explore the "fixed-fixed" boundary conditions for applications different from our motivation of simultaneous acoustic confinement and high-power thermal management. Specifically, Lakin's work describes coupling multiple piezoelectric layers to enhance k_t^2 only, while Segovia-Fernandez et al. specifically employ a "resonant confiner" to mitigate lateral acoustic loss and improve the quality factor in a TE-based BAW resonator. Besides, Bahr et al., investigated phonon crystals for acoustic confinement in CMOS-MEMS resonators, with a focus on integrability. Such Bragg reflector-based or multilayered-phonon crystal designs require extremely precise thickness control for each layer to function, significantly increasing the fabrication cost. Critically, they introduce multiple thermal interfaces, which are known to create significant thermal boundary resistances. As established in the literature, such vertical interfaces can severely impede heat flow in micro-scale devices, causing the effective cross-plane thermal conductivity to fall far below the intrinsic value of the bulk materials [1, 2]. This creates a fundamental trade-off between acoustic confinement and thermal dissipation in these multi-layer approaches.

In contrast, our work introduces a first-of-its-kind, co-designed architecture for IDT-based transducers that tackles the less-explored challenge of high-power handling head-on. The broader impact of our work lies in addressing a fundamental bottleneck shared by several emerging interdisciplinary platforms that rely on high-performance IDT-based acoustic transducers. For instance, in the multi-beam acousto-optic beam steering (mAOBS) system reported by Lin et al. [3], several independent IDT-based TF-SAW transducers are driven with multi-frequency microwave signals to generate numerous, independently steerable optical beams for MIMO free-space communication. The authors clearly identify that the scalability in beam count and data rate is directly limited by the power tolerance and thermal reliability of the constituent TF-SAW transducers. Enhancing IDT power handling is therefore essential to directly increase the beam count, improve modulation bandwidth, and ensure robust operation. Similarly, in advanced acoustic manipulation techniques, the field generated by a single propagating IDT is used to create complex interference patterns for particle control [4]. The spatial range and patterning force of such time-averaged fields are directly governed by the acoustic intensity, which is ultimately limited by the transducer's power tolerance. Thus, improving IDT power handling directly strengthens and extends the capabilities of these manipulation techniques. Furthermore, for integrated acoustoelectric RF signal processors, increasing the RF power dynamic range and ensuring stable operation under bias are identified as critical challenges [5]. Our work directly targets this shared limitation at the core transducer level. By fundamentally improving IDT power-handling capability, we aim to extend the

practical performance ceiling and scalability of these pioneering systems, from high-throughput photonics to powerful lab-on-a-chip platforms. This aligns with the scope of *Nature Communications* in publishing significant advances that are of interest to researchers across multiple disciplines. And the novelty is not merely adding a layer, but in its synergistic electro-thermo-mechanical function:

- The over-layer is designed not just for confinement, but to fundamentally redistribute the mechanical stress field and suppress the primary failure mechanism under high power. This distinction is quantitatively demonstrated by the depth-resolved von Mises stress profiles in Fig. R23, where all data are normalized to the peak stress in the baseline $LiNbO_3$/sapphire TF-SAW transducer at a reference input power, P_0 (Fig. R23). While the commercial TC-SAW device, mounted a 1.9 μm-thick SiO_2 top layer, reduces the von Mises stress to approximately 3/5 of the baseline, our proposed LAW platform achieves a drastically lower and more uniform stress distribution, with a value of only 1/4 of the baseline stress at P_0. The advantage of our architecture becomes even more pronounced under a higher power density. Notably, at an input power ten times higher ($10P_0$), the maximum stress in our LAW platform remains lower (~0.8×) than the stress in the baseline $LiNbO_3$/sapphire TF-SAW transducer at its reference power, P_0. In contrast, the TC-SAW platform already exceeds the baseline stress level (~1.22×) at only $4P_0$. This analysis proves that the LAW architecture is not a trivial material deposition. Instead, it is a targeted design that enhances power capacity by redistributing and mitigating mechanical stress at the fundamental level, thereby directly inhibiting the driving force for acoustomigration. This represents a paradigm of functional co-designing process that moves beyond simple confinement to achieve holistic performance gains.
- Contrary to complex multi-layer stacks, we employ a single, significantly thicker over-

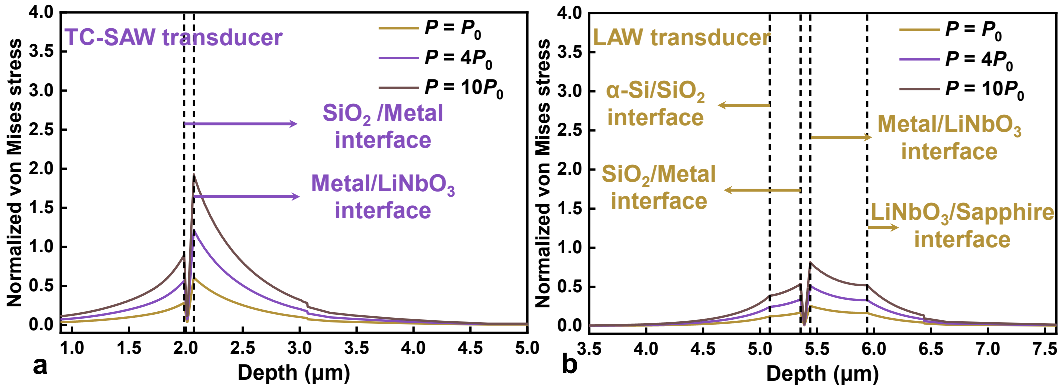

Figure. R23. Depth profiles of the extracted von Mises stress for **(a)** $SiO_2/LiNbO_3$ TC-SAW and **(b)** our proposed α-Si/SiO_2/$LiNbO_3$/Sapphire LAW DUTs. All mechanical stress profiles are normalized to the maximum stress in the baseline TF-SAW in Fig. R22 at reference power, P_0. Key interfaces are marked by dashed lines.

layer. While its intrinsic thermal conductivity is moderate, its increased thickness and direct placement create an efficient vertical heat-spreading path. This design explicitly minimizes the interfacial thermal resistance penalty associated with numerous thin layers.

- The efficacy of this integrated thermal management strategy is not merely simulated but experimentally confirmed. We have provided infrared camera imaging that visually demonstrates the improved heat dissipation from the active region of our device under high-power, a direct validation absent in the cited BAW literature.

Our proposed architecture is a foundational platform, not a final optimum. We explicitly state that the over-layer material can be further optimized. For instance, a single-crystal silicon substrate could be bonded atop the flattened SiO_2-overcoated TF-SAW device, maximizing the acoustic confinement while further enhancing the cross-plane thermal conductivity near the theoretical limit of silicon. This potential for seamless material substitution for superior thermal performance underscores the flexibility and novelty of our co-design concept.

In conclusion, while the "fixed-fixed" boundary conditions were reported for purposes like Q-enhancement or coupling boosting, our work is the first to demonstrate a co-designed fixed-fixed IDT-based acoustic wave architecture that synergistically engineer the von Mises stress distribution and integrated thermal management, validated by direct experiment. We have revised the manuscript in Introduction and Results to more precisely highlight this innovation in light of prior art. We thank the reviewer for prompting this valuable clarification.

[1] Dou, Z., Lei, C., Wu, K. & Yu, G. "The development of thermal interface materials." *Nat. Electron*. **8**, 1146–1155 (2025).

[2] Huang, Z. et al. "First demonstration of three-dimensional thermal conductivity distribution measurements of interconnect stacks down to 3nm process nodes." in *Proc. 2025 IEEE International Electron Devices Meeting (IEDM)*. 23.2.1-23.2.4 (2025).

[3] Lin, Q. et al. Optical multi-beam steering and communication using integrated acousto-optics arrays. *Nat. Commun.* **16**, 4501 (2025).

[4] Devendran, C., Collins, D. J., Ai, Y. & Neild, A. Huygens-Fresnel acoustic interference and the development of robust time-averaged patterns from traveling surface acoustic waves. *Phys. Rev. Lett.* **118**, 154501 (2017).

[5] Hackett, L. et al. Towards single-chip radiofrequency signal processing via acoustoelectric electron–phonon interactions. *Nat. Commun.* **12**, 2769 (2021).

ACTION TAKEN:

The Introduction and Results have been revised to more precisely position our innovation

relative to prior work on fixed-fixed boundaries. We have clarified that the novelty lies not in adding a top layer per se, but in its **synergistic electro-thermo-mechanical** revolution. This includes a new discussion of the unique mechanical stress redistribution and integrated thermal management, supported by added FEA simulation in Figs. 6c and 6d.

4. The authors test and compare resonator power handling near the anti-resonance frequency (fa). The justification for this is well supported in the paper as this maximizes the resonator absorbed power and temperature rise when testing low impedance resonators in a 50-ohm network. However, when testing acoustic filters, which by design are well-matched to 50-ohms, the failures often occur near the resonant frequency (fr) of the acoustic resonators (as opposed to the anti-resonant frequency) as this is where the currents are maximized and typically electro-migration of the metals is the dominant high power failure mechanism. The authors admit as much in the SI, that their approach tests for acousto-migration (not electro-migration) near fa where the peak electrical currents are suppressed. The results would be much more impactful at the technological level if simple filters were constructed that are matched to 50 ohms for the power testing. Filters with as few as 3 resonators would allow a full characterization of the power handling benefits across frequency. As currently written, the paper reports a 14x increase in power density for resonators near fa and it is entirely unclear what the benefits are of this at the filter level where high power failures occur near fr.

AUTHOR RESPONSE:

We thank the reviewer for this very insightful concern regarding failure mechanisms and the suggestion for practical validation. We appreciate the opportunity to clarify our methodology and to present additional data that addresses the core of this concern.

Measuring a filter composed of multiple transducers cannot assess its power tolerance from the perspective of individual components. In the filtering application, the failure frequency point varies across topologies. We think that testing at the filter level is neither the only nor the best practice for benchmarking the fundamental architectural improvement of the transducer itself, for two key reasons:

- First, the motivation and impact of our work are fundamentally at the transducer level. Our research is driven by the fundamental challenge of overcoming the power-handling limitation intrinsic to interdigital transducers (IDTs) as individual components. This advancement is critical not only for traditional RF elements but also for emerging transformative platforms, such as integrated acousto-optics and precise acoustic field manipulation, where performance is dictated by individual transducers, not composite filters, as detailed in our previous response to Comment #1.
- Second, a filter's power handling is a system-level metric, not a pure component metric.

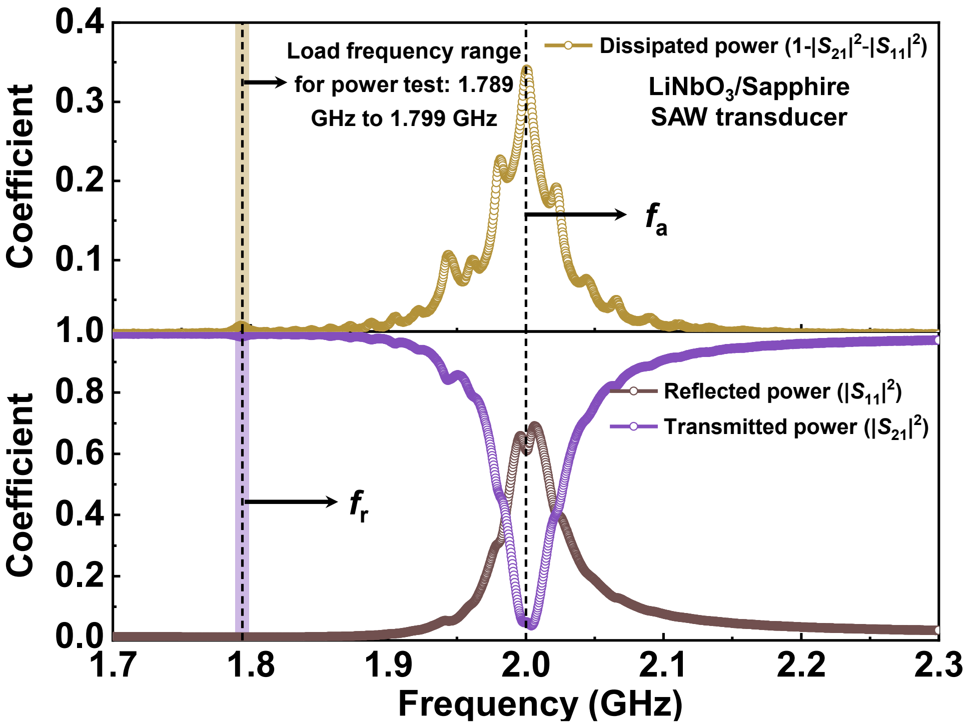

Figure. R24. Dissipated, reflected, and transmitted power coefficients as a function of driving frequencies for a 2-port thin-film $LiNbO_3$/Sapphire TF-SAW transducer under a -15 dBm incident load. Frequency range for power test: 1.789–1.799 GHz.

> It is confounded by design-specific variables including topology, power routing, electromagnetic matching networks, and test conditions. These factors can obscure the intrinsic improvement of the core resonator technology, making filter-to-filter comparisons an unreliable benchmark for an accurate, device-level assessment of technological advances across different acoustic wave platforms. For instance, our own controlled experiments (detailed in previous response to Comment #1) show that identical filter layouts can exhibit a 9.42 dB variation in failure threshold based solely on connection configuration and packaging, underscoring that system-level results are an unreliable benchmark for isolating device-level architectural advances. Experimental details and discussion are provided in our previous response to Comment #1. Comparing power handling across different filter topologies is unfair, and it is also impossible for authors to use the same topology as the products in the market.

Nevertheless, we fully agree with the reviewer's technical point that electromigration at f_r is another critical failure mode for acoustic wave devices. Therefore, we have followed the suggestion to extend our transducer-level assessment at f_r to comprehensively investigate this mechanism. We first characterized the RF power budget at f_r for a TF-SAW transducer, as presented in Fig. R24. The tested bandwidth was 10 MHz centered at f_r, identical to the range used in the main text. Within this band, the calculated power reflection coefficient is 0.00017 and the transmission coefficient is 0.9834, indicating nearly negligible power rejection. The associated power dissipation at f_r, though marginally higher relative to adjacent bands, is orders

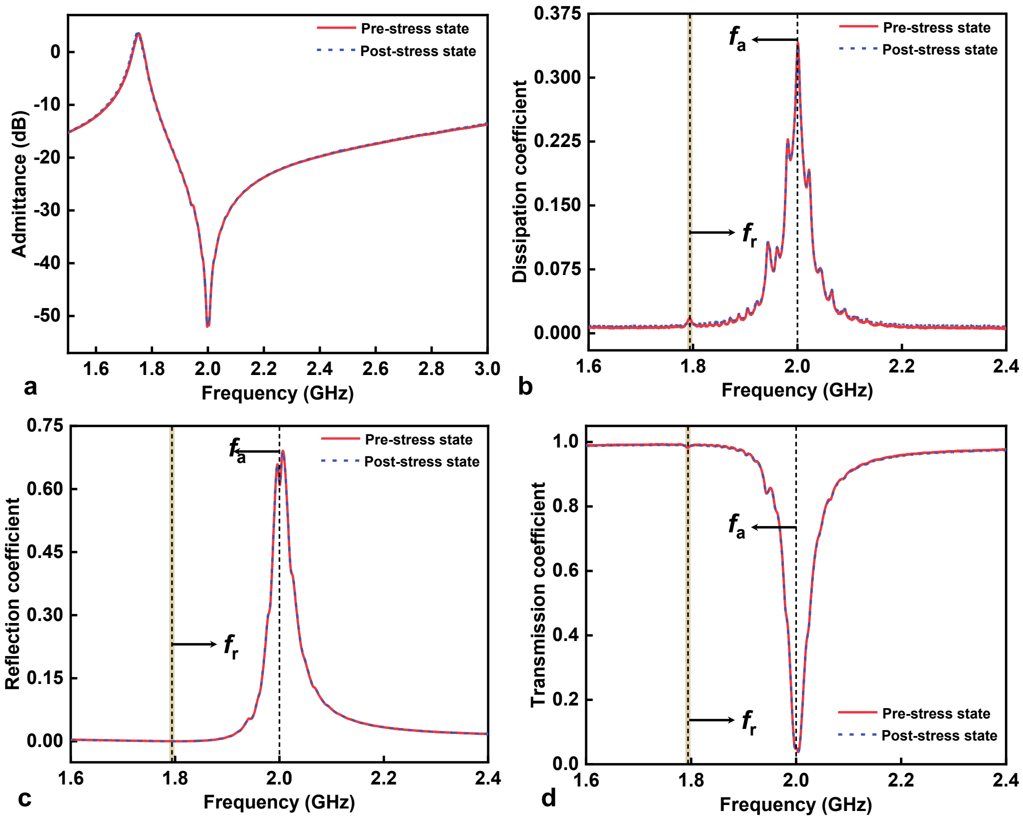

Figure. R25. **a**, Admittance curves, **b**, dissipation, **c**, reflection, and **d**, transmission coefficients for the $LiNbO_3$/Sapphire TF-SAW DUT, compared before and after high-power testing at a maximum injected power density of 45.93 dBm/mm^2. The measurements were performed under a -15 dBm incident load.

of magnitude lower than that near the anti-resonant frequency (f_a).

Subsequently, the device was subjected to a high-power stress test following the established test procedure in the main text, with the injected power density increased from 25.93 dBm/mm^2 to 45.93 dBm/mm^2. No appreciable steady-state temperature rise was captured, even at power levels surpassing the failure point of the LAW transducer in Fig. 5c in the main text. Post-stress characterization was performed by remeasuring *S*-parameters at a low power (-15 dBm) after a necessary off-wafer calibration. Post-stress low-power *S*-parameter measurements confirmed no degradation in device performance (Fig. R25). This combination of evidence—minimal dissipation at f_r, absence of significant temperature-rise under extreme power, and robustness in post-stress performance—strongly indicates that for our transducers, the predominant failure mechanism under high acoustic power is acoustomigration (driven by mechanical stress) rather than electromigration.

In principle, electromigration, which predominantly occurs at f_r, is another critical failure mode for acoustic wave devices. Therefore, to investigate the competing failure mechanisms in acoustic wave devices—specifically, electromigration (driven by current density) versus acoustomigration (driven by mechanical stress)—we performed finite element analysis (FEA) on a TF-SAW transducer unit cell to extract the frequency-dependent von Mises stress profile

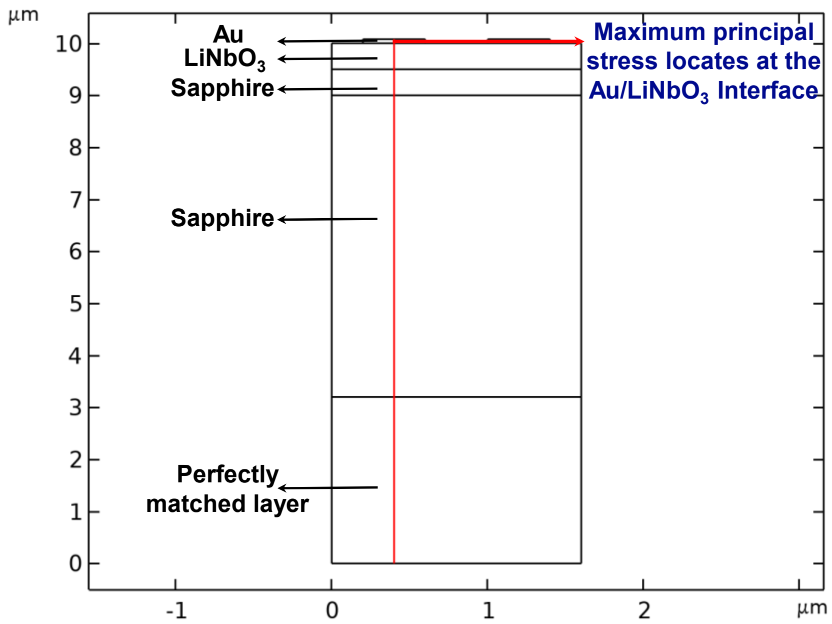

Figure. R26. Method for extracting the von Mises stress along the depth direction, spanning from the top surface of the interdigital layer down to the sapphire substrate.

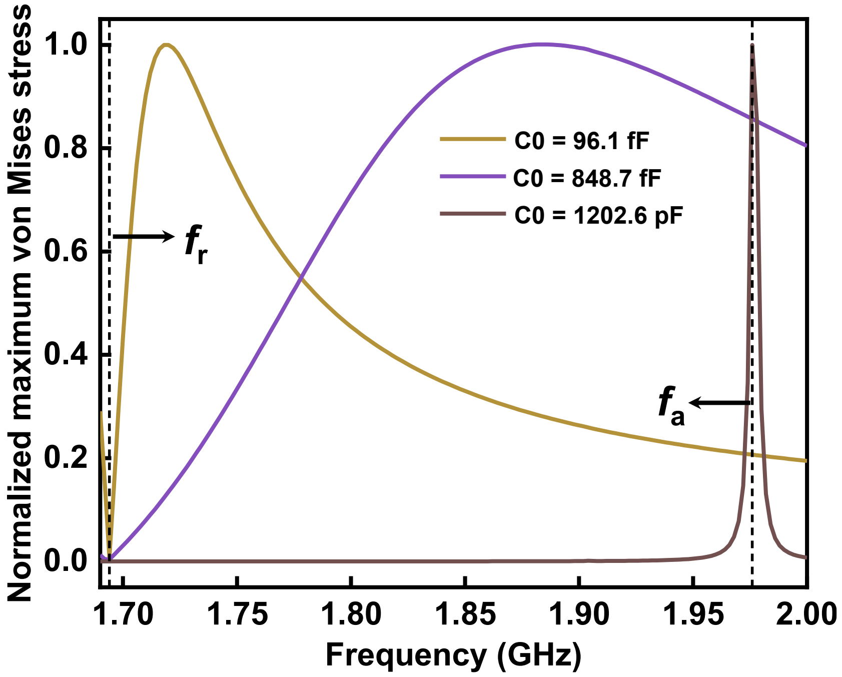

Figure. R27. Simulated von Mises stress profiles versus frequency for the $LiNbO_3$/sapphire SAW device with varied static capacitances. The maximum stress is consistently located at the metal/$LiNbO_3$ interface. For each device, all profiles are normalized to the maximum von Mises stress in the depth direction extracted from the frequency sweep simulation.

and identify the weakest failing point in the frequency domain, as indicated in Fig. R26. A perfectly matched layer (PML) was applied at the bottom boundary to absorb leaked acoustic energy and eliminate spurious reflections. The simulated stress profile was obtained by sweeping the frequency and recording the maximum von Mises stress along the depth (from the IDT top surface into the substrate), which consistently peaked at the critical Au/$LiNbO_3$ interface.

As shown in Fig. R27 (with f_r and f_a indicated by dashed lines, as they are capacitance-independent), the normalized stress peak systematically shifts from the vicinity of f_r toward f_a as the static capacitance increases from 96.1 fF to 1202.6 pF. This shift is rooted in the transducer's impedance: At f_r, the transducer behaves as a near-short circuit. This maximizes current density (promoting electromigration) but results in a minimal potential difference between IDT fingers, leading to negligible mechanical strain and thus minimal von Mises stress (thereby minimizing acoustomigration). Conversely, as the load frequency increases towards f_a, the high impedance leads to a large potential difference, maximizing mechanical stress and acoustomigration risk. Therefore, the dominant failure mechanism and its corresponding "weakest failing point" in the frequency domain are not fixed; they shift between f_r and f_a, depending on the device's electrical design (i.e., its static capacitance), as showcased in Fig. R27. For our transducers for power tests, the static capacitance was specifically designed to complement the spatial resolution of our infrared camera, which consequently shifted the failure point at the f_a. In a standard filter design, the static capacitance is typically tuned for 50-Ω impedance matching, which positions the peak power dissipation (and thus the most likely failure point) somewhere between f_r and f_a. This controlled alignment further validates that the observed failures are primarily driven by acoustomigration at the designed stress maximum, rather than by electromigration.

ACTION TAKEN:

In response, we have undertaken the following revisions: (1) clarified in the revised main text why filter-level power handling is a system-level metric unsuitable for benchmarking the intrinsic performance advancement from architecture innovations; (2) added the detailed filter-level comparison and analysis to the revised Supplementary Information, Section 11; (3) comprehensively expanded our transducer-level analysis by performing high-power tests at the f_r in the revised Supplementary Information, Section 12; (4) conducted finite-element simulations to investigate the competing electromigration and acoustomigration failure mechanisms, identifying the frequency- and capacitance-dependent failure point (revised Supplementary Information, Section 15).

5. While in Fig. 4a the LAW transducer has no observable spurs directly at fa, there are still a large number of spurs between fr and fa that are clearly observable in both the admittance plot in 4a and the Bode-Q in 4b. This will cause high passband ripple in any eventual high order filter implementation and clearly causes large oscillations in the Bode-Q. The authors should discuss the implications of these spurs and how they might be eliminated in the SI. The authors should not claim, as is done in 4a, that the response is spurious free. The spurs appear to simply have been shifted away from fa allowing for a higher Q at fa.

AUTHOR RESPONSE:

We thank the reviewer for the insightful observation regarding the spurious modes present between f_r and f_a in Figs. 4a and 4b. We agree that such features can contribute to passband ripples in filter implementations and that our initial description could be more precise. We have revised the manuscript accordingly to clarify that our claim of spurious suppression specifically refers to the elimination of key parasitic plate modes (e.g., A0, S0, and SH1 mode) inherent to the baseline TF-SAW and SiO_2-overcoated TF-SAW structures, rather than denoting a completely spur-free response across the entire spectrum. Below, we address the nature of the spurs in Fig. 4a and their implications.

The advantage of our LAW structure is not merely a frequency shift of the spurious mode near f_a, but in the fundamental suppression of specific plate modes at the coupling level (Fig. R28). As demonstrated by the eigenmode analysis in Figs. 2f and 2g, the design of the material stack and acoustic boundary conditions nullifies the electromechanical coupling to modes like the strong A0 mode, which remains prominent in the other two platforms. Similarly, higher-order modes like SH1 mode (in SiO_2-overcoated) and S0 mode (in TF-SAW) are also suppressed. These results can be directly attributed to the tailored sound cone and stress field, providing more freedom in acoustic boundary designs. Additionally, it should be emphasized that all three structures in Fig. 4a share identical IDT designs; the observable suppression in spurious modes is thus a direct consequence of the LAW's acoustic boundary engineering.

The spurious modes noted by the reviewer between f_r and f_a are of a different, well-understood nature. They are primarily transversal modes arising from waveguiding effects in the aperture direction, as can be confirmed via FEM simulation. Proven techniques exist and can be directly applied to our LAW architecture, including: apodized or tilted electrodes to destructively interfere with transversal harmonics [1, 2], slowness curve modulation via substrate engineering, piston-mode designs to modulate the velocity profile [1, 3, 4]. Therefore, these spurs represent an addressable design consideration rather than a fundamental limitation of the LAW concept.

In response to this comment, we have taken two concrete steps. First, we have revised the caption of Fig. 4a to precisely state that the LAW design eliminates the specified parasitic plate modes, avoiding the overgeneralized term "spurious-free." Second, we have added a discussion in the Supplementary Information, Section 6, addressing the origin of the high-order transversal spurs and outlining established methods (e.g., piston-mode design) for their suppression in future implementations.

We thank the reviewer for this valuable comment, which has helped us present our findings with higher quality. The revised manuscript now more clearly distinguishes between the fundamental plate-mode suppression achieved by our architecture and the manageable

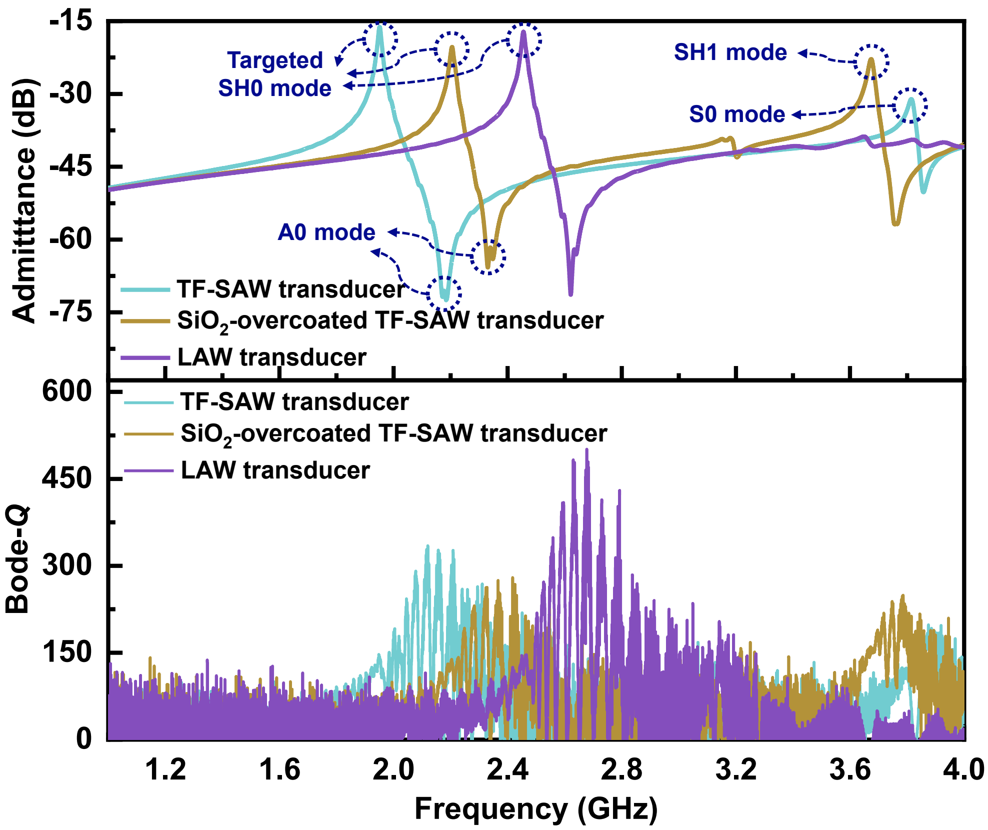

Figure. R28. Measured wideband admittance and extracted Bode-*Q* characteristics of the TF-SAW, SiO_2-overcoated TF-SAW, and LAW transducers. All three transducers originate from an identically fabricated baseline TF-SAW transducer; the SiO_2-overcoated and LAW transducers are fabricated and tested by subsequently overcoating the same device with SiO_2 and α-Si, respectively, enabling a direct evaluation of the architectural modifications.

engineering of transversal modes.

[1] Hashimoto, K. et al. "Revisiting piston mode design for radio frequency surface acoustic wave resonators." In Proc. *2022 IEEE MTT-S International Conference on Microwave Acoustics and Mechanics* (IC-MAM) 60–63 (IEEE, 2022).

[2] Xu, H. et al. "SAW filters on $LiNbO_3$/SiC heterostructure for 5G n77 and n78 band spplications." *IEEE Trans. Ultrason. Ferroelectr. Freq. Control* 70, 1157–1169 (2023).

[3] Qian, F., Zheng, J., Xu, J. & Yang, Y. "Heterogeneous interface-Enhanced thin-film SAW devices using Lithium Niobate on Si." *IEEE Microw. Wirel. Technol. Lett.* 35, 123–126 (2025).

[4] Shen, J. et al. Suppressed transverse mode generation in TF-SAW resonators based on $LiTaO_3$/Sapphire. *IEEE Electron Device Lett.* 45, 2241–2244 (2024).

ACTION TAKEN:

We have revised Figs. 4a and 4b to present the wide-spectrum admittance response and clarified that the LAW design specifically suppresses key parasitic plate modes. A further discussion on the nature and mitigation of the observed transversal spurs and bulk waves has been added in

the revised Supplementary Information, Section 6.

6. The authors state that the optimized residual stress for the α-Si is 4.379 MPa. This is a very low value and the authors should place some error bars around this measurement using radius of curvature (ROC). It is also highly unlikely the film stress can be measured with 1 kPa resolution using wafer curvature measurements as the significant digits imply in the listed value.

AUTHOR RESPONSE:

We thank the reviewer for the insightful comment regarding the precision of the residual stress value reported for the α-Si film. We appreciate the opportunity to clarify our measurement methodology, error analysis, and to provide updated and averaged data from a more comprehensive analysis.

The residual stress was determined using the well-established wafer curvature technique based on Stoney's equation, as discussed in the Supplementary Information. To ensure accuracy and account for wafer-scale uniformity, we performed profile measurements on a 4-inch wafer both before and after α-Si deposition. As schematically illustrated in Fig. R29, nine equally spaced traces were profiled across the wafer under test. The change in height along each trace was used to calculate the radius of curvature (ROC) along the trace. Fig. R30a presents the measured average ROC and its associated error bar for each of the nine traces, represented as a scatter plot with a fitted curve. The ROC is the primary experimental observable. Subsequently, the mean residual stress for each trace was calculated from its average ROC using Stoney's equation. Fig. R30b shows the calculated average stress and its error bar for each trace, also as

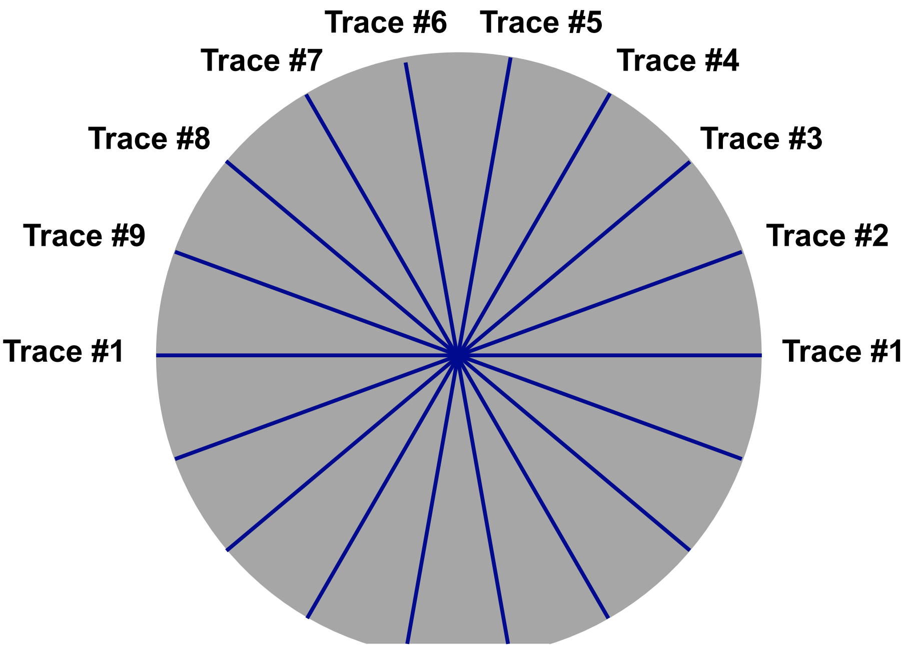

Figure. R29. Schematic of the radius of curvature (ROC) measurement for pre- and post- α-Si deposition. Residual stress was extracted using the complete Stoney equation applied to nine uniformly distributed scans on a 4-inch wafer.

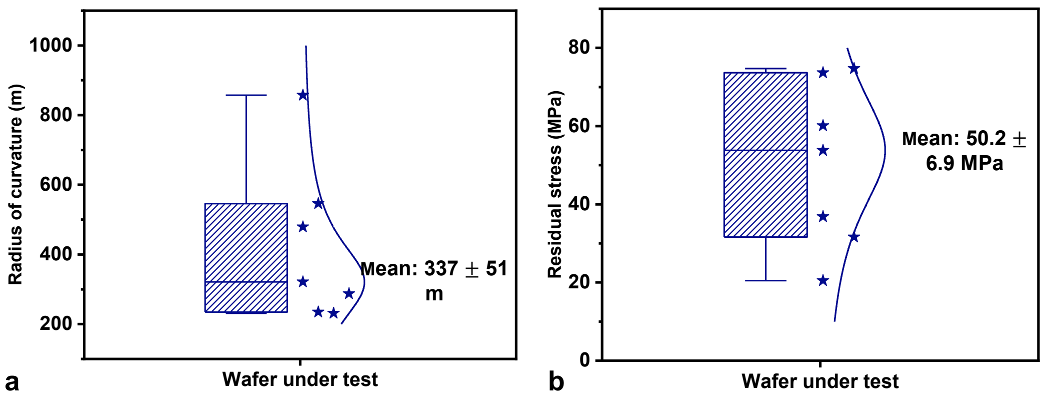

Figure. R30. Statistical distribution of the ROC (a) and residual stress (b) for Traces #1 through #9. Data are presented as mean ± standard deviation, with average values of 337 ± 51 m (ROC) and 50.2 ± 6.9 MPa (residual stress).

Table R3. Summary of measured ROC and residual stress values across Traces #1–#9.

Trace No	Radius of curvature (m)	Residual stress (MPa)	Trace No	Radius of curvature (m)	Residual stress (MPa)
#1	478.9	36.84	#6	287.4	60.11
#2	7477	2.31	#7	545.8	31.65
#3	321	53.81	#8	-3053	-5.658
#4	234.6	73.63	#9	857	20.47
#5	231.1	74.74			

a scatter plot with a fit. Crucially, the error bar for residual stress is not measured directly but is derived through standard error propagation analysis from the uncertainty in the ROC measurement, incorporating the uncertainties in film thickness and substrate parameters. Therefore, the resolution of 1 kPa is not a claim about the direct measurement instrument but is a consequence of this calculation and error propagation process. The four significant figures in the initially reported value (4.379 MPa) originated from the numerical output of this precise calculation based on the average experimental inputs.

Following the reviewer's suggestion, we have summarized the measured and derived data from all nine traces, as shown in Table R3. Upon recalculating the weighted average after excluding two clear outliers — Trace #2 and Trace #8 (anomalously high ROC values, potentially from measurement near the wafer edge) — we obtain a refined average residual stress of 50.2 ± 6.9 MPa (with a corresponding average ROC of 337 ± 51 m). We have updated this value throughout the revised manuscript. This comprehensive averaging yields a more reliable and representative result. We emphasize that updated residual stress remains at a very low level for a deposited amorphous silicon film of this thickness.

In summary, the high numerical precision of the residual stress value stems from a

calculation based on multi-trace curvature measurements and error propagation. We have now provided full error bars (Figs. R30a, R30b) and a complete dataset table, and have corrected the manuscript to report the finalized, averaged value of 50.2 ± 6.9 MPa. We thank the reviewer for prompting this important clarification and more rigorous data presentation.

ACTION TAKEN:

The residual stress value for the α-Si thick film has been updated based on a comprehensive multi-trace curvature analysis. Full error bars, the complete dataset, and a detailed discussion of the measurement methodology have been provided in the revised Supplementary Information, Section 21.

7. There appears to be an extra legend entry for TF-SAW transducer in Fig. 4a.

AUTHOR RESPONSE:

We thank the reviewer for their careful observation regarding the legend in Fig. 4a. The entry in question resulted from a line break in the lengthy label "SiO_2-overcoated TF-SAW transducer," which may have created the visual impression of a separate legend item. We have revised the figure to present this label more clearly, eliminating any potential for misinterpretation. We appreciate the reviewer's attention to detail, which has helped improve the clarity of our presentation.

ACTION TAKEN:

Fig. 4a has been revised.

****

Reviewer #3 (Remarks to the Author):

****

The paper appears to report a modified TC-SAW, with the top SiO_2 layer replaced by aSi. The benefit here is that the energy is better confined at the interface. The paper seems to have been written with significant effort in its presentation. However, the authors should provide a fairer comparison between this LSAW technology and the well-established TC-SAW (SiO_2 on top of LN/LT) and IHP-SAW (LN/LT-SiO_2-carrier) technologies. Despite the authors replacing a thick top SiO_2 layer with Si, the reviewer is not convinced that the platform performs better than the conventional technology. A few reasons below:

AUTHOR RESPONSE:

Thank the reviewer for the critical concern. We would like to point out that this is a foundational study, and the primary motivation is to reveal and address the limitations of power-handling in acoustic wave transducers, not only for RF filter products.

If the comparison with filter products in the market is necessary, our work differs fundamentally from temperature-compensated SAW (TC-SAW) devices in the very physical mechanisms governing resonant frequency, electromechanical coupling, and power handling:

In TC-SAW, the resonant frequency varies with the thickness of SiO_2 due to the insufficient energy confinement of SiO_2. In contrast, α-Si in our proposed LAW exhibits a critical thickness (~0.6 μm) beyond which the resonant frequency saturates and becomes invariant (Fig. R31). This is because the acoustic energy is effectively confined at the IDT/$LiNbO_3$ interface, with the strain field decaying rapidly into the α-Si layer, making it act

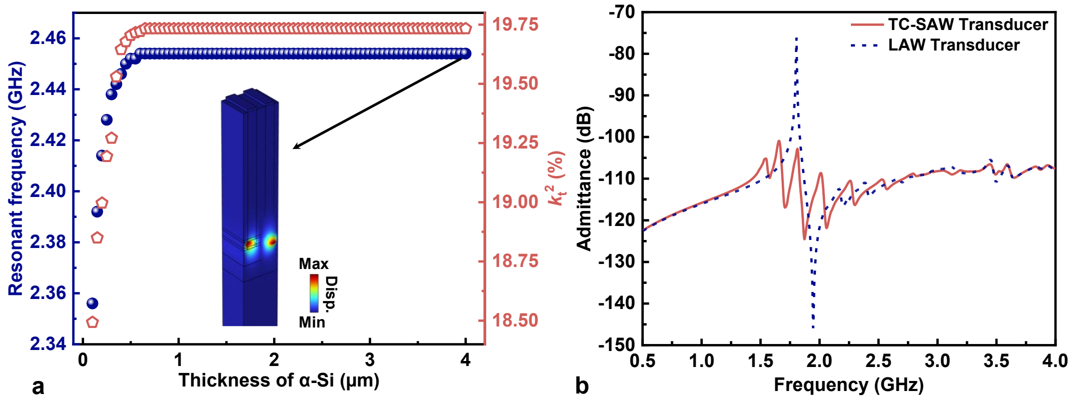

Figure. R31. **(a)** Resonant frequency and k_t^2 dependence on the α-Si top-cladding thickness and corresponding mode profile at a thickness of 4 μm. **(b)** Admittance responses comparing the traditional TC-SAW (with a 4 μm SiO_2 layer) and the proposed LAW transducer (with a 4 μm α-Si layer).

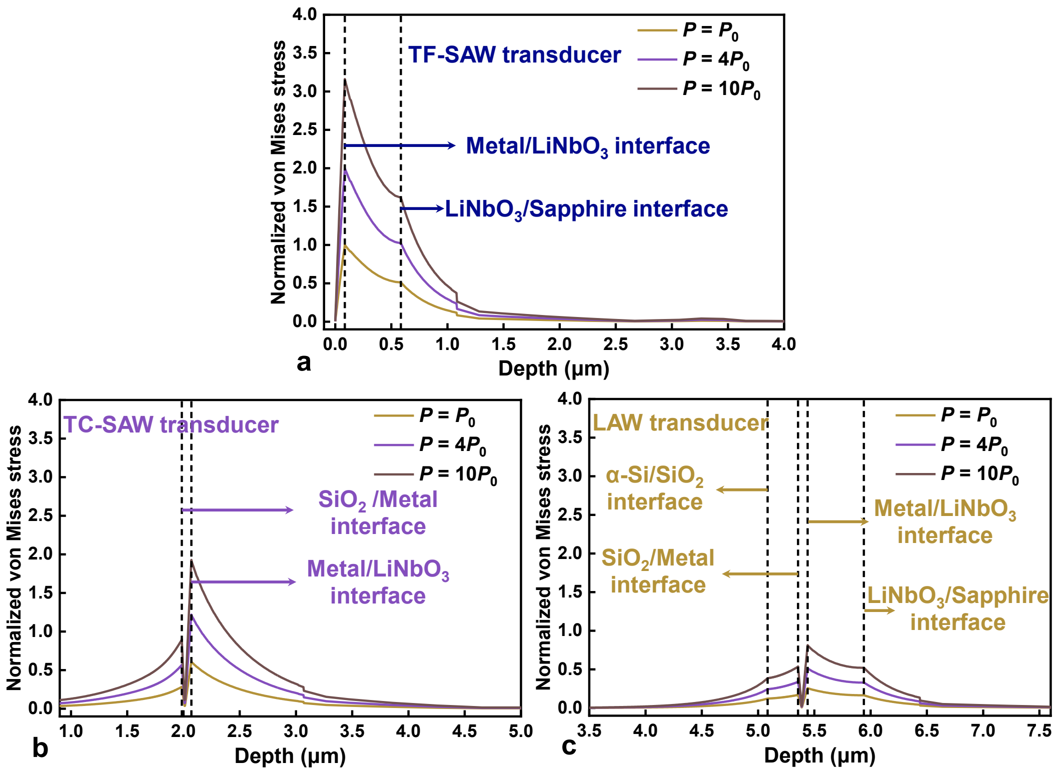

Figure. R32. Depth profiles of the extracted von Mises stress for **(a)** baseline $LiNbO_3$/Sapphire TF-SAW, **(b)** SiO_2/$LiNbO_3$ TC-SAW, and **(c)** our proposed α-Si/SiO_2/$LiNbO_3$/Sapphire LAW DUTs. All mechanical stress profiles are normalized to the maximum stress in the baseline $LiNbO_3$/Sapphire TF-SAW DUT **(a)** at reference power, P_0. Key interfaces are marked by dashed lines.

as a semi-infinite medium for the guided mode. Similarly, temperature compensation in TC-SAW comes at a significant cost, typically sacrificing k_t^2 to be less than 7% and introducing more bulk spurious modes (Fig. R31b). Conversely, our LAW platform enhances k_t^2 to over 18% while simultaneously providing temperature compensation, as shown in Fig. R31b. This highlights a fundamental advantage in temperature compensation.

Stress engineering for power handling: we performed a finite-element analysis of the von Mises stress distribution to fundamentally understand the power-handling limits across acoustic platforms from the SAW technology family, including the baseline $LiNbO_3$/Sapphire TF-SAW, SiO_2/$LiNbO_3$ TC-SAW, and our α-Si/SiO_2/$LiNbO_3$/Sapphire LAW transducers. For each platform, the mechanical stress profile along the depth (from the top surface to the bottom boundary) was evaluated at the frequency corresponding to its weakest failure point, with all geometrical features held identical in the simulations. In Fig. R32, all normalized depth profiles of von Mises stress are presented relative to the maximum stress value obtained in the baseline $LiNbO_3$/Sapphire TF-SAW transducer at a reference input power, P_0. Since the mechanical stress is proportional to the square root of input power (as validated in Fig. R32), these differences directly correlate with power-handling capability. Notably, the device on the SiO_2/$LiNbO_3$ TC-SAW transducer exhibits higher mechanical stress within the cavity at the

same injected power compared to that of our LAW platform. While a commercial TC-SAW device, mounted a 1.9 μm-thick SiO_2 top layer, reduces the von Mises stress to approximately 3/5 of the baseline, our proposed LAW platform achieves a drastically lower and more uniform stress distribution, with a value of only 1/4 of the baseline stress at P_0. The advantage of our architecture becomes even more pronounced under a higher power density. Notably, at an input power ten times higher ($10P_0$), the maximum stress in our LAW platform remains lower (~0.8×) than the stress in the baseline $LiNbO_3$/sapphire TF-SAW transducer at its reference power P_0. In contrast, the TC-SAW platform already exceeds the baseline stress level (~1.22×) at only $4P_0$. This analysis proves that the LAW architecture is not a simple replacement of top SiO_2 layer. Instead, it is a targeted design that enhances power capacity by redistributing and mitigating mechanical stress at the fundamental level, thereby directly inhibiting the driving force for acoustomigration.

Therefore, characterizing our work as a "modified TC-SAW" overlooks its foundational contributions. We have introduced a new design paradigm that co-optimizes materials and boundaries in multi-physics domains not merely for confinement, but for holistic performance transformation. We provide more detailed physical explanations in the following responses to further support that this work represents a significant conceptual and practical advance beyond incremental modifications to existing technology. This represents a paradigm of functional co-designing process that moves beyond simple confinement to achieve holistic performance gains.

1) The resonators show really low Q below 500 in the so-called LSAW, while it is well known that the IHP-SAW has Q over 5000, and the TC-SAW has Q over 2000. The new platform is simply not comparable to the existing technologies in commercial products. The 2.5 GHz or so operating frequency for this work is also a range that has been well studied by different technologies. If the authors build filters using the technology, the results might be only moderate compared to existing commercial products.

AUTHOR RESPONSE:

We thank the reviewer for raising this critical point regarding the quality factor (Q) and the technology's position relative to mature commercial platforms like IHP-SAW and TC-SAW. We fully acknowledge that the Q of our current proof-of-concept devices is below the exceptionally high levels achieved by these highly optimized products after years of dedicated development. Below, we provide context for this difference and clarify the distinct focus, mechanistic insight, and promise of our foundational study.

First, the primary motivation of this work is to introduce and validate a new architectural paradigm for overcoming the power-handling limitation, a critical yet comparatively less

[Figure Redacted]

Figure. R33. **a-f**, Evolution of the thin-film bulk acoustic resonator (FBAR) Q-circle starting from 1992 to 2007 [1].

explored bottleneck for high-power microacoustic applications rather than Q optimization. Our core contribution lies in demonstrating that engineered control of the internal von Mises stress distribution and integrated thermal management can fundamentally raise the power-density ceiling. This addresses a key need for next-generation, high-power-density applications, rather than aiming for direct, comprehensive performance parity with commercial filters at this initial proof-of-concept stage.

Second, it must be noted that achieving high Q is a recognized, complex challenge. As established in the literature [1], the final Q is a metric affected by first-order design choices and numerous second-order effects, including but not limited to: acoustic reflections and scattering on strips and IDTs, charge distribution on IDTs, mechanical and topographical loading at edges, refraction and diffraction, substrate anisotropy, and spurious modes. Fully optimizing these requires extensive, iterative development, as evidenced by the 15-year evolution of thin-film bulk acoustic wave resonator (FBAR) technology to achieve $Q > 5000$, as seen in Fig. R33 [1].

Our current proof-of-concept devices prioritize demonstrating the new power-handling principle. However, they already show a clear pathway for Q enhancement. For instance, by integrating a slow-wave medium to modify the mechanical boundary, we experimentally increased the Q from 344 (baseline TF-SAW transducer) to 889 (engineered LAW transducer), as detailed in the revised Supplementary Information, Section 6. This more than 2.5-fold improvement, achieved without introducing new loss mechanisms, strongly suggests that dedicated optimization targeting the known loss mechanisms can push Q beyond 2000 in future

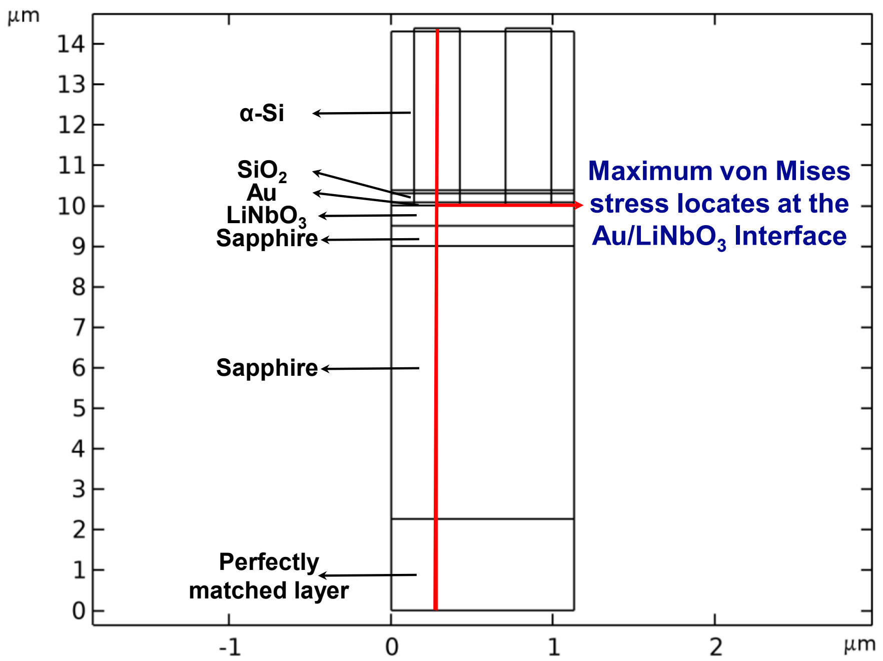

Figure. R34. Method for extracting the von Mises stress along the depth direction, spanning from the top surface of the LAW transducer down to the substrate.

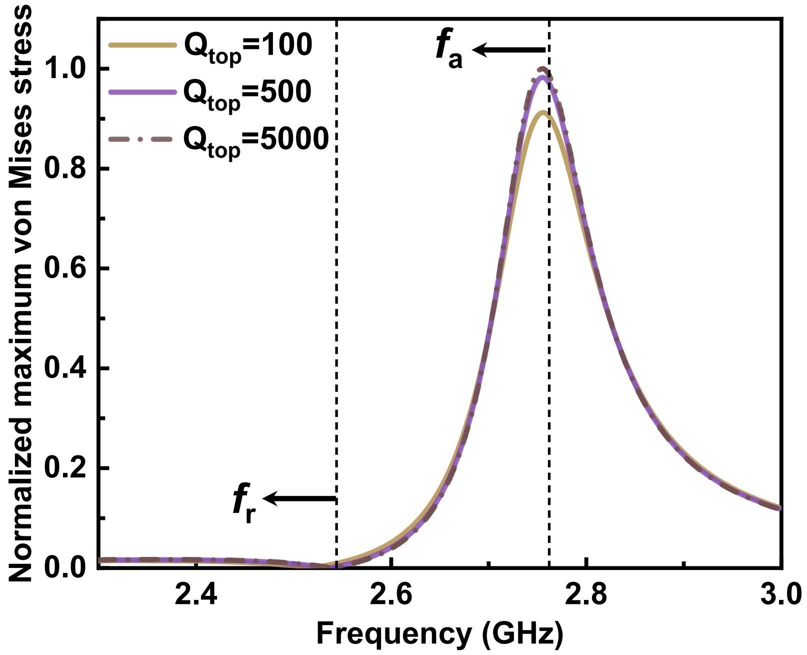

Figure. R35. Simulated von Mises stress profiles as a function of frequency for the LAW transducer, showing the effect of varying the quality factors in the top layers. Stresses are normalized to the maximum in-depth von Mises stress simulated for the case with a top-layer quality factor of 5000.

iterations. Further established strategies, such as bonding a single-crystal silicon wafer, offer more viable routes for further Q enhancement.

Last, a key question is the relationship between Q and the power-handling capability that is the central focus of our work. As pointed out by Hashimoto [1], the power handling capability of acoustic devices shows a strongly positive correlation with the internal mechanical stress

field. To investigate this, we performed a finite element analysis to examine the correlation between the Q (modelled via the material loss in the stress-manipulation layer) and the resulting von Mises stress profile—the primary driver of acoustomigration failure (Fig. R34). The results, presented in Fig. R35, reveal a critical insight: the normalized von Mises stress in the passband remains remarkably stable even as Q is varied over two orders of magnitude (from 100 to 5000). This indicates that the fundamental power-handling enhancement achieved through our stress-engineering architecture is not intrinsically limited by the current Q value. While the absolute insertion loss of a filter would be affected, the core advancement in power resilience remains valid and impactful.

We therefore position this study as a foundational step that addresses a key unmet need in power handling and provides a new design variable (stress engineering) for the community. We agree that direct performance parity with mature commercial filters requires further work. Consequently, we have been careful to limit our claims in the manuscript, explicitly stating the need for future optimization of Q and temperature stability. The value of this work lies in demonstrating a viable new approach to solve a persistent problem, opening a co-design space where power handling and Q can be simultaneously improved for next-generation, high-power acoustic devices.

[1] RF bulk acoustic wave filters for communications. (Artech House, Norwood, Mass.; London, 2009).

2) The power handling should not be performed at the resonator level, but at the filter level. In resonators, most signals are reflected and are also affected by the device's impedance. A better comparison method would be to build a filter and compare the power-handling or H2/H3 harmonics of the new technology with those of existing technologies. Currently, the authors seem to be building IHP-SAW/TC-SAW testbeds that are worse than the state of the art and then comparing the LSAW with them. This might not be the best practice.

AUTHOR RESPONSE:

We thank the reviewer for pointing out this concern. With respect, we would like to emphasize that the focus of this work is on acoustic wave transducers, rather than RF filters, and we intentionally avoid restricting their applications to RF filtering so as not to overlook broader cross-disciplinary relevance and other researchers' contributions, as discussed in the Introduction. Regarding the accurate assessment of power handling capability, we respectfully disagree with the suggestion that testing at the filter level is the only or best practice for evaluating the fundamental architectural advancement presented in this work. Below, we explain our rationale, grounded in the study's motivation and supported by new controlled

experiments.

First, we are motivated by the fundamental challenge of overcoming the power-handling limitation inherent to IDTs at the individual component level, which is critical for advancing next-generation acoustic-wave platforms. While piezoelectric acoustic waves have long been used in RF filters, the individual IDT-based transducer has evolved into a transformative platform for emerging fields such as integrated acousto-optics and precise acoustic field manipulation. For instance, in the multi-beam acousto-optic beam steering (mAOBS) system reported by Lin et al. [1], several independent IDT-based TF-SAW transducers are driven with multi-frequency microwave signals to generate numerous, independently steerable optical beams for MIMO free-space communication. The authors clearly identify that the scalability in beam count and data rate is directly limited by the power tolerance and thermal reliability of the constituent TF-SAW transducers. Enhancing IDT power handling is therefore essential to directly increase the beam count, improve modulation bandwidth, and ensure robust operation. Similarly, in advanced acoustic manipulation techniques, the field generated by a single propagating IDT is used to create complex interference patterns for particle control [2]. The spatial range and patterning force of such time-averaged fields are directly governed by the acoustic intensity, which is ultimately limited by the transducer's power tolerance. Thus, improving IDT power handling directly strengthens and extends the capabilities of these manipulation techniques. Furthermore, for integrated acoustoelectric RF signal processors, increasing the RF power dynamic range and ensuring stable operation under bias are identified as critical challenges [3]. Our work directly targets this shared limitation at the core transducer level. By fundamentally improving IDT power-handling capability, we aim to extend the practical performance ceiling and scalability of these pioneering systems, from high-throughput photonics to powerful lab-on-a-chip platforms. This aligns with the scope of *Nature Communications* in publishing significant advances that are of interest to researchers across multiple disciplines.

Second, while the authors recognize that a comprehensive comparison could help contextualize our work, a fundamental prerequisite for a meaningful comparison is a benchmarking method that isolates architectural innovation. Power handling measured at the filter level, however, reflects a system-level property influenced by numerous design-specific factors, such as cascading resonators, power-routing layout, matching networks, and test conditions. These variables can obscure the intrinsic improvement attributable solely to the core resonator technology. Consequently, comparing literature values obtained under different conditions can be misleading. This variability holds true even when comparing filters composed of nominally identical resonators. For instance, Dong et al., have experimentally shown that a squared array-based topology enhances total power handling by 8.8-dB at the filter level under the same effective resonator area and technology, simply by distributing power across multiple

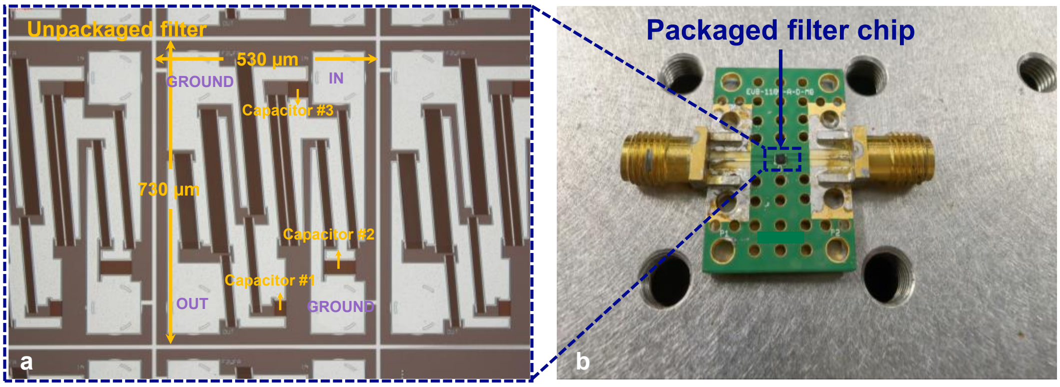

Figure. R36. **a**, Microscope image of a filter before packaging, indicating the input, output, and ground terminals. **b**, Optical image of the packaged filter (after the packaging process of the filter shown in **(a)**) mounted on an evaluation board with SMA connections.

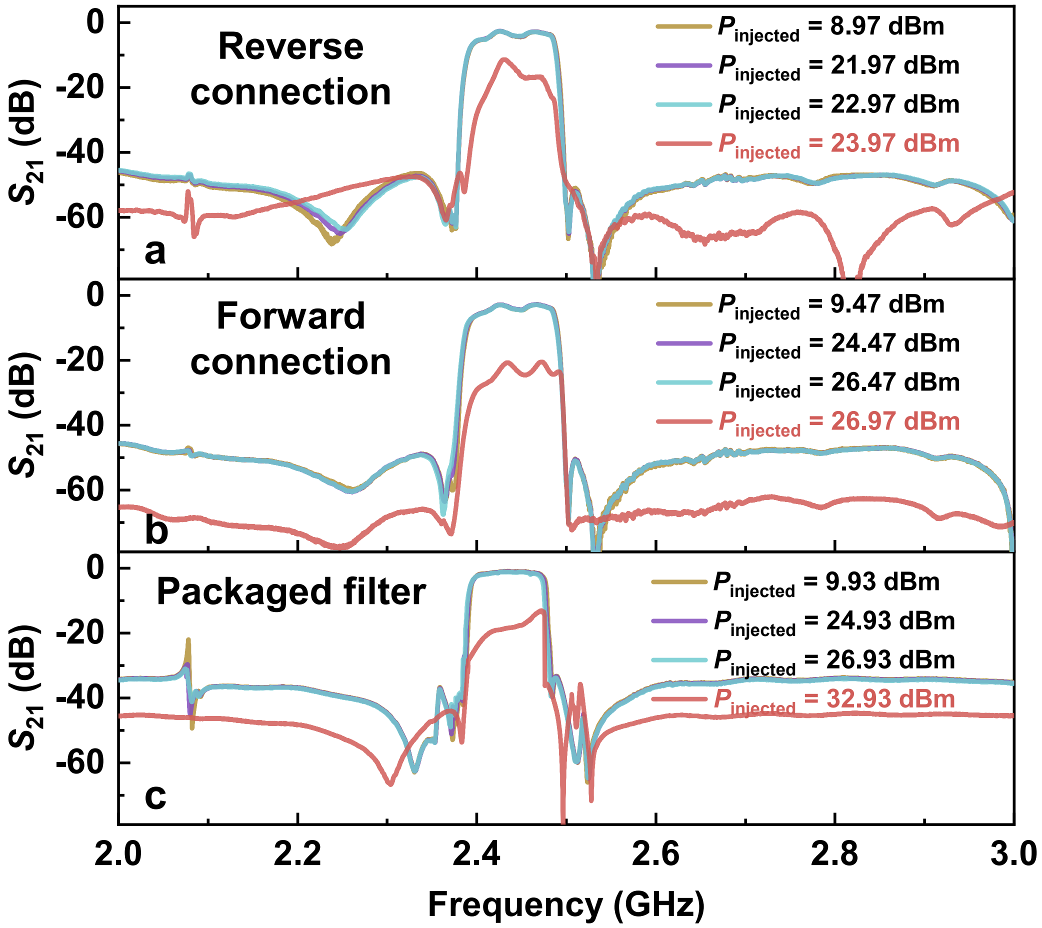

Figure. R37. Comparison of S_{21} responses measured under high-power test conditions across different electrical connection configurations of IHP-SAW filters: **(a)** reverse-connected and unpackaged, **(b)** forward-connected and unpackaged, and **(c)** forward-connected and packaged.

resonators without improving the intrinsic power density thresholds of individual devices. [4].

As a direct step to better substantiate our claims, we performed a well-controlled, high-power evaluation using filters built from identical constituent resonators in an identical physical layout, varying only the electrical connection sequence and the presence of encapsulation. Fig. R36a presents the microscope image of unpackaged filter layout, featuring specifically

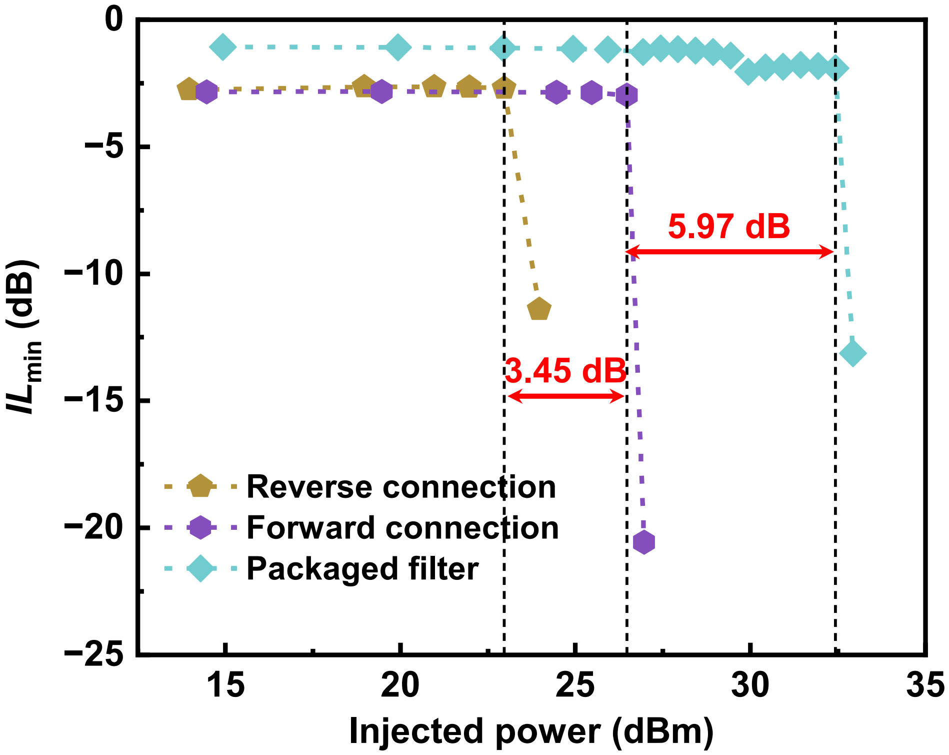

Figure. R38. Measured minimum value of insertion loss (*IL*) under high-power test conditions across different electrical connection configurations of IHP-SAW filters.

designed input/output terminals and three integrated capacitors. Fig. R36b shows the optical image of the same filter after packaging, mounted on an evaluation board with SMA connectors. To clarify our measurement: ***forward connection*** denotes the configuration where incident power first arrives at the filter's input terminal; ***reverse connection*** for the opposite case; only the forward configuration was tested for the packaged filter. Fig. R37 summarizes the S_{21} responses under increasing injected power for the unpackaged filter (forward/reverse connections) and the packaged filter. First, while all were stable up to 22.97 dBm, their failure points diverged sharply: 23.97 dBm (reverse, unpackaged), 26.97 dBm (forward, unpackaged), and 32.93 dBm (packaged). Fig. R38, plotting the minimum insertion loss versus injected power, clearly shows these distinct bottlenecks, with a maximum difference of 9.42 dB — even though all filters use identical resonators.

We further investigated via high-power continuous-wave tests on the forward-connected, unpackaged filter at different loading frequencies (Fig. R39). First, thermal images of the forward-connected filter indicate elevated dissipation at passband edges (2.3942 GHz, 2.4842 GHz) compared to the center (2.4515 GHz), with the spatial temperature profile — and thus the internal power distribution — shifting markedly with frequency. This directly visualizes the design-specific and frequency-dependent power flow within the composite acoustic-electromagnetic system. Subsequently, post-test microscopic inspection (Fig. R40) revealed that the locations and failure modes of the damaged resonators varied for each test frequency. Taken together, this combination of thermal, electrical, and mechanical evidence solidifies our

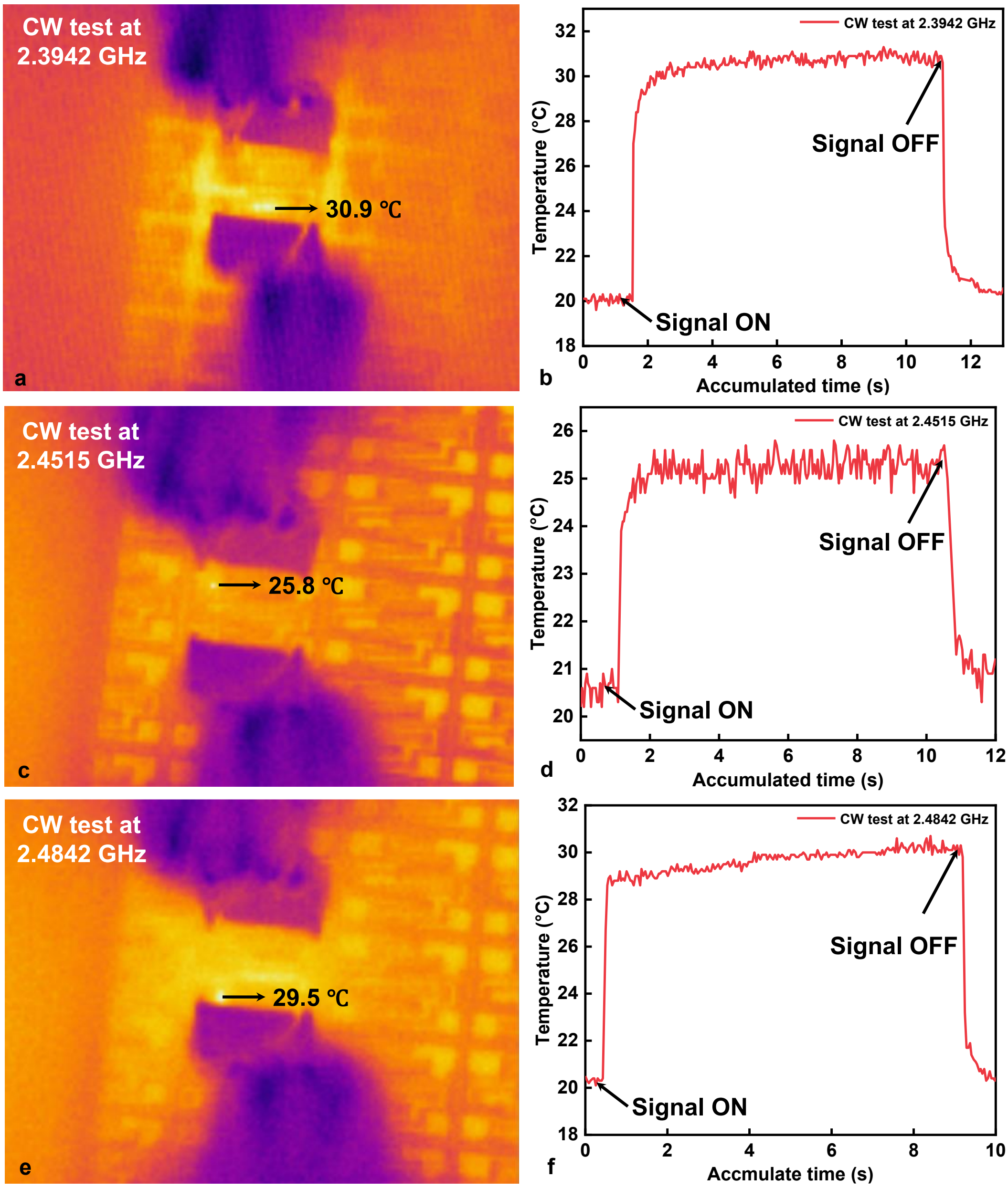

Figure. R39. Thermal characterization of the unpackaged, forward-connected IHP-SAW filters under high-power continuous wave (CW) tests at different loading frequencies. Each pair of panels shows the extracted thermal image (left) and the corresponding temperature versus accumulated time plot (right): (**a, b**) 2.3942 GHz (left edge of the passband), (**c, d**) 2.4515 GHz (center of the passband), and (**e, f**) 2.4842 GHz (right edge of the passband).

conclusion: ***the point of failure in a filter is not predetermined by the resonator alone but is a dynamic outcome of system-level power routing.*** Therefore, assessing power handling at the filter level yields a metric that is intrinsically confounded by design-specific power division and layout. It follows that such a system-level result is misleading and cannot serve as a direct benchmark for the inherent performance of the constituent resonator technology across different acoustic wave platforms.

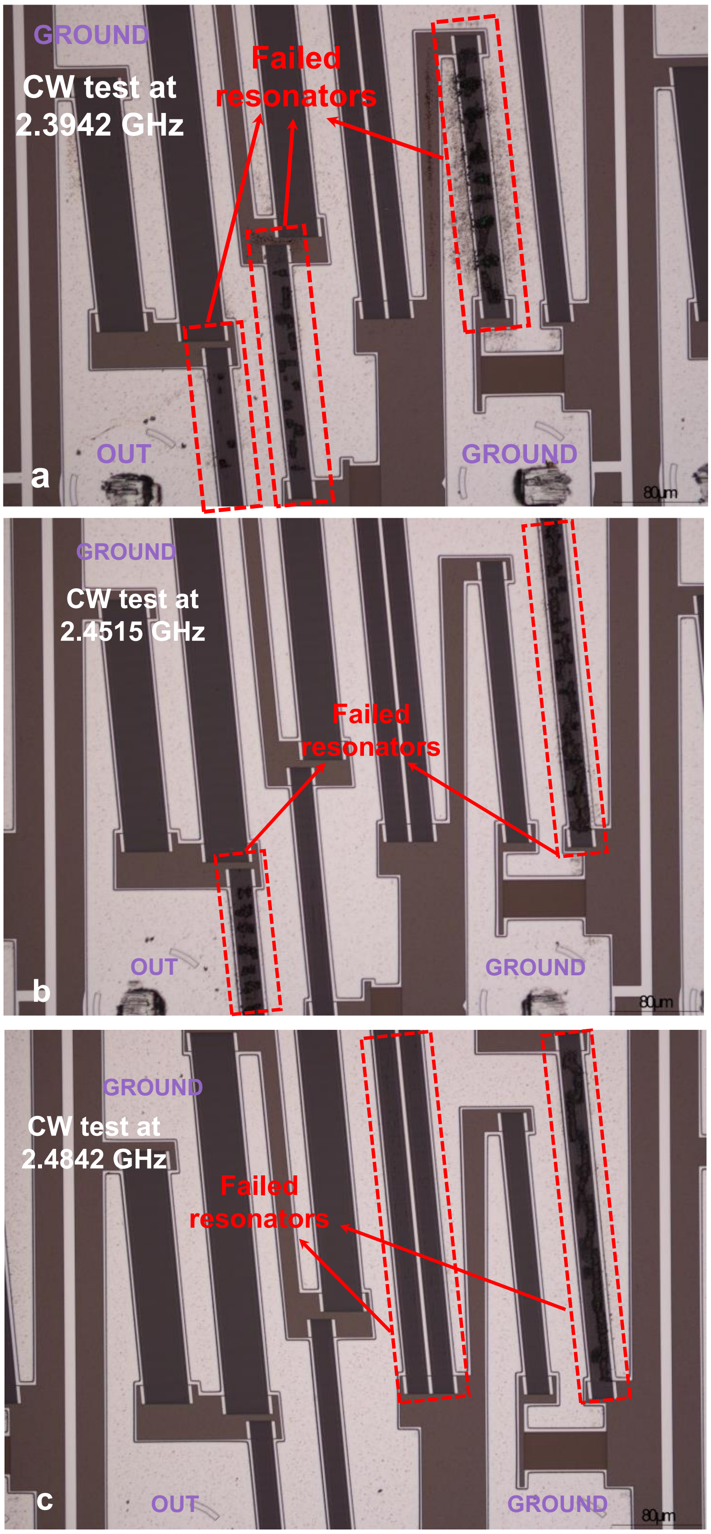

Figure. R40. Post-characterization optical microscope images of the unpackaged IHP-SAW filters (forward-connected), corresponding to the filters after being subjected to the high-power CW tests at various frequencies detailed in Figure R14.

To directly address the reviewer's comment, we have expanded our experimental comparison to include multiple state-of-the-art platforms under identical testing conditions, including X-$LiNbO_3$/SiC TF-SAW, X-$LiNbO_3$/Sapphire TF-SAW, SiO_2/bulk 128°Y-$LiNbO_3$

TC-SAW, and our α-Si/SiO_2/X-$LiNbO_3$/Sapphire LAW architectures. The detailed experiments are as follows:

We firstly conducted a dedicated comparative study to quantitatively evaluate the impact of ***substrate thermal management*** on ultimate power-handling limits. We selected sapphire and SiC as the supporting substrates and fabricated TF-SAW transducers with identical layouts on two substrates for their significantly different thermal conductivities, as summarized in Table R4.

Table R4. Material properties overview between $LiNbO_3$, Sapphire, and SiC

**Materials**	**Thermal conductivity (W/m/K)**	**Acoustic impedance (kg/m²/s)**	**Fundamental shear velocity (m/s)**
$LiNbO_3$	4.2	1.73e+7	3674 (±4%)
Sapphire	32.5	2.4e+7	6045
SiC	370	2.29e+7	7126

Following the power test framework established in the main text, we began with small-signal *S*-parameters to calculate the RF power reflection, transmission, and dissipation at each frequency, providing an estimation of which frequency will become hottest and represent the weakest failing point (Fig. R41). We acknowledge potential port impedance mismatch at the single-transducer level and therefore define the injected power as the actual power delivered to the device (incident minus reflected power); the reflection coefficients for both devices in the test band were identical, ensuring a fair comparison.

The results reveal a critical insight. Fig. R42 summarizes the measured steady-state temperature rise versus injected power density. As expected, the DUT on high-thermal-conductivity SiC exhibited far superior heat spreading, with an operating temperature approximately 16 °C lower than its sapphire-based counterpart at comparable power levels. However, despite this substantial thermal advantage, both devices failed at very similar injected power densities. The difference in their failure thresholds, identified from the inflection points in the ΔT-vs-injected power scatter plots, is only 0.78 dB. Post-failure admittance measurements confirmed irreversible degradation in both cases, as detailed in Fig. R43.

To fundamentally understand the power-handling limits across the tested acoustic platforms, we performed a finite-element analysis of the von Mises stress distribution. For each platform, the mechanical stress profile along the depth (from the top surface to the bottom boundary) was evaluated at the frequency corresponding to its weakest failure point, with all geometrical features held identical in the simulations. In Fig. R44, all normalized depth profiles of von Mises stress are presented relative to the maximum stress value obtained in the

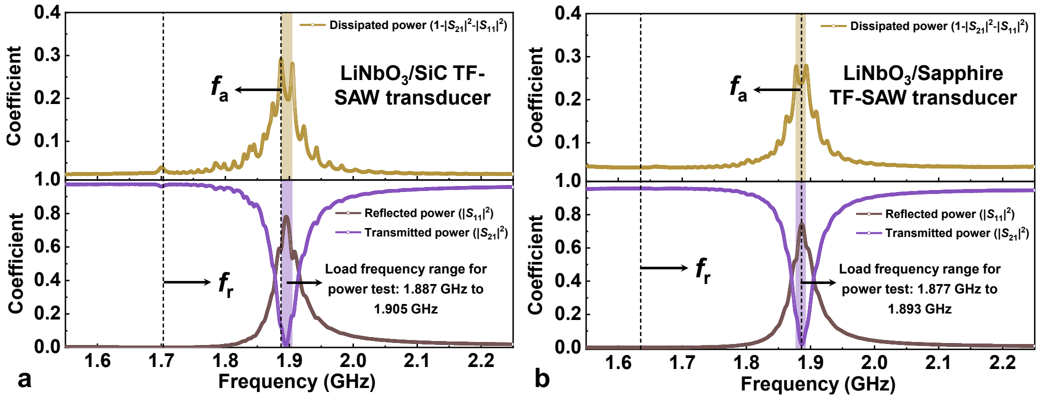

Figure. R41. **a**, Dissipated, reflected, and transmitted power coefficients as a function of driving frequency for a 2-port thin-film $LiNbO_3$/SiC TF-SAW transducer operating at a load power of $P_{incident}$ = -15 dBm. **b**, Dissipated, reflected, and transmitted power coefficients as a function of driving frequencies for a 2-port thin-film $LiNbO_3$/Sapphire TF-SAW transducer operating at a load power of $P_{incident}$ = -15 dBm. Both transducers share an identical wavelength design to ensure equitable high-power comparison under similar loading frequencies. The resonant frequency (f_r) and anti-resonant frequency (f_a) are marked by the dashed lines in each plot. The loading frequency ranges for the two transducers under high power tests are also indicated.

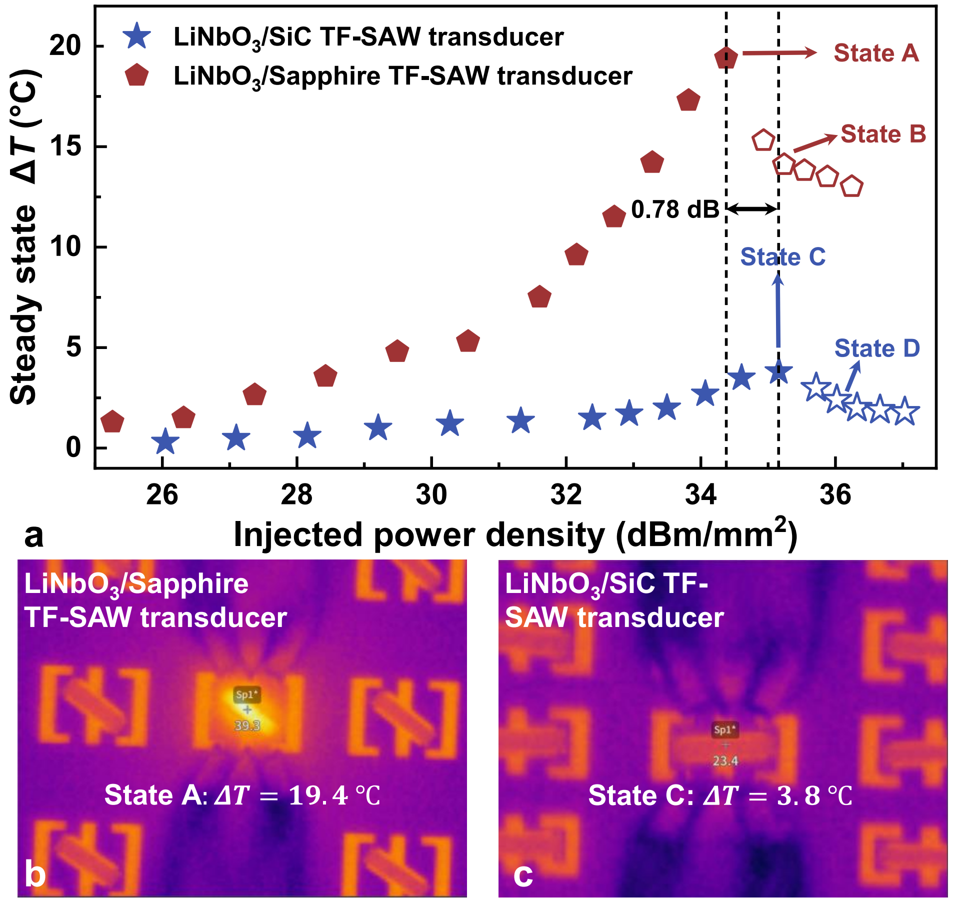

Figure. R42. **a**, Steady-state temperature rise (ΔT) as a function of injected power density, measured by thermal imaging for devices on $LiNbO_3$/SiC and $LiNbO_3$/Sapphire platforms. **(b, c)**, Representative thermal images of the $LiNbO_3$/Sapphire DUT **(b)** and $LiNbO_3$/SiC DUT **(c)** under specific testing conditions (power level and driving frequency). All measurements were conducted at an ambient temperature of ~20 °C.

$LiNbO_3$/sapphire TF-SAW transducer at a reference input power, P_0. The simulations reveal a

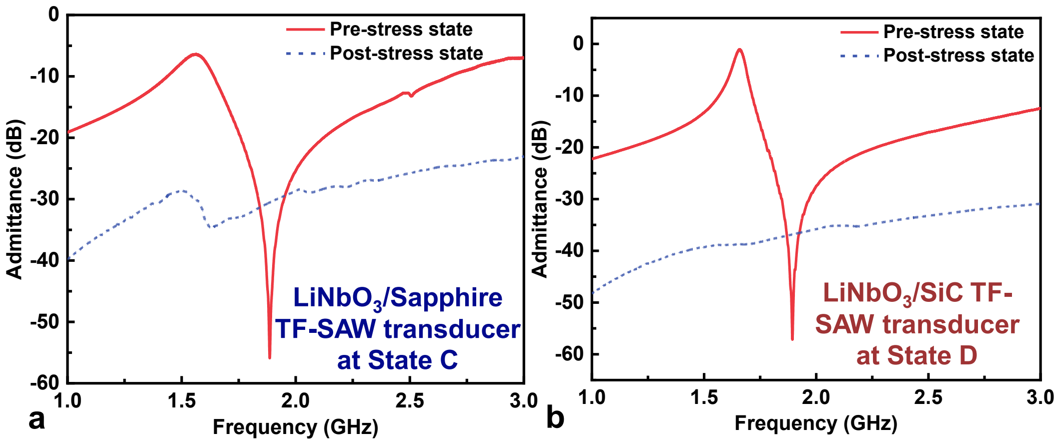

Figure. R43. **a**, A comparison of admittance curves for the $LiNbO_3$/Sapphire TF-SAW transducer between the pre-power test state and post-power test state. **b**, A comparison of admittance curves for the $LiNbO_3$/ SiC TF-SAW transducer between the pre-power test state and post-power test state. All measurements were performed at a load power of P_{incident} = -15 dBm.

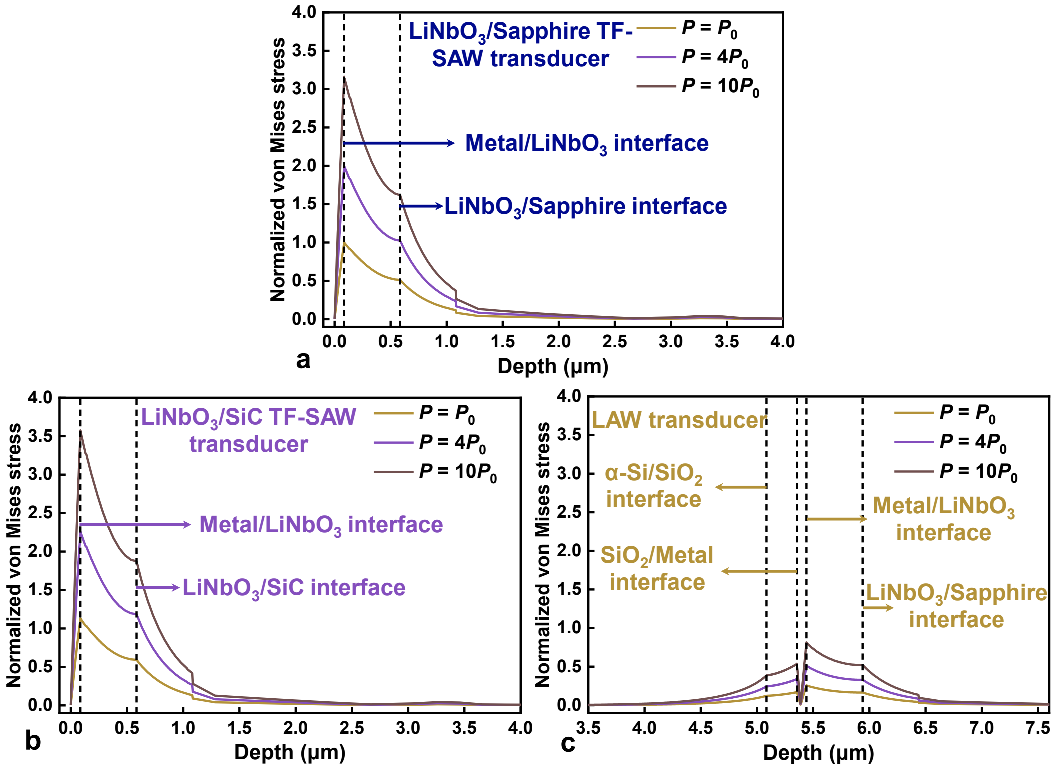

Figure. R44. Depth profiles of the extracted von Mises stress for **(a)** baseline $LiNbO_3$/Sapphire TF-SAW DUT, **(b)** $LiNbO_3$/SiC TF-SAW DUT, and **(c)** our proposed α-Si/SiO_2/$LiNbO_3$/Sapphire LAW DUTs. All mechanical stress profiles are normalized to the maximum stress in the baseline $LiNbO_3$/Sapphire TF-SAW DUT **(a)** at reference power, P_0. Key interfaces are marked by dashed lines.

key insight: while the stress universally peaks at the IDT/$LiNbO_3$ interface, its magnitude within the $LiNbO_3$ cavity differs significantly among the structures. Since the mechanical stress is proportional to the square root of input power (as validated in Fig. R44), these differences directly correlate with power-handling capability. Notably, the device on the stiffer SiC

substrate exhibits higher mechanical stress (~1.13×) within the cavity at the same injected power. This is because SiC's higher acoustic impedance reflects more energy back into the resonator cavity, intensifying the mechanical stress that drives acoustomigration. In contrast, our proposed LAW platform achieves the minimum stress distribution along the depth direction, with a value of only 1/4 of the baseline stress at P_0. Additionally, the mechanical stress within the resonator cavity is more uniformly distributed compared to the other two TF-SAW structures. Notably, even under an input power ten times higher ($10P_0$), the maximum stress in the LAW platform (~0.8×) remains lower than that in the baseline $LiNbO_3$/Sapphire TF-SAW transducer at the reference power P_0.

In summary, under identical test conditions using injected power density (incident minus reflected), our LAW architecture exhibits an 11.09-fold (10.45 dB) improvement over $LiNbO_3$/SiC TF-SAW and a 12.73-fold (11.23 dB) improvement over $LiNbO_3$/sapphire TF-SAW transducers. This controlled experiment provides direct evidence that while substrate engineering is effective for thermal management, the ultimate power-handling bottleneck is dictated by mechanical stress concentration at the IDT/$LiNbO_3$ active interface. A substrate change alone cannot alleviate this root cause and may even exacerbate it. This finding strongly supports our claim: a more effective strategy is to engineer the top-side boundary conditions of the resonator. Our proposed LAW architecture directly implements this principle by redesigning the upper acoustic boundary to redistribute and mitigate the peak mechanical stress, thereby achieving a fundamental improvement in power handling that transcends the limitations of substrate optimization alone. This comparative analysis has been added to the Supplementary Information to strengthen our argument.

In addition, we also performed a direct power durability comparison with an industry-standard commercial TC-SAW device (from a top-tier TC-SAW company) using our established protocol, which is comprehensively presented in the following response to Comments #3. This comparison serves to evaluate the impact of top boundary engineering strategy, not merely a trivial material deposition or SiO_2 replacement, and thereby highlights the advances achieved by our mechanical stress and thermal boundary engineering innovations.

Last, while the authors acknowledge the reviewer's concern regarding signal reflection and impedance at the transducer-level, it is crucial to note that, within our selected frequency range, the power rejection due to port impedance mismatch is identical for both the TF-SAW and LAW transducer architectures tested. This equivalency is ensured by our delicate mechanical boundary design, which eliminates any comparative bias from this factor. Therefore, to isolate and fairly compare the core technological advancement, we respectfully disagree with the reviewer's comment, and have to insist that our component-level (transducer-level) comparison provides the most essential and unbiased metric. We believe these advances could benefit the broad readership of *Nature Communications* and may help inspire future

developments in high-power acoustics. We again appreciate the reviewer's feedback, which helped us improve the manuscript substantially.

[1] Lin, Q. et al. Optical multi-beam steering and communication using integrated acousto-optics arrays. *Nat. Commun.* **16**, 4501 (2025).
[2] Devendran, C., Collins, D. J., Ai, Y. & Neild, A. Huygens-Fresnel acoustic interference and the development of robust time-averaged patterns from traveling surface acoustic waves. *Phys. Rev. Lett.* **118**, 154501 (2017).
[3] Hackett, L. et al. Towards single-chip radiofrequency signal processing via acoustoelectric electron–phonon interactions. *Nat. Commun.* **12**, 2769 (2021).
[4] Wang, W. et al. "Ultra-high power handling capability bulk acoustic wave filters using square array topology." *J. Micromechanics Microengineering* **35**, 085009 (2025).

ACTION TAKEN:

We added new experimental data on the power tests at the filter level (revised Supplementary Information, Section 11) and provided detailed discussions about the rationale for the power tests at the transducer level in the revised manuscript.

3) Some of the state-of-the-art comparison tables might miss out important data points. For instance, the current -13 ppm/K TCF is not as good as commercial TC-SAW.

AUTHOR RESPONSE:

We thank the reviewer for the critical assessment regarding the temperature stability and for providing a more detailed comparison with mature commercial platforms like temperature-compensated SAW (TC-SAW). We acknowledge that the absolute TCF value of our proof-of-concept device requires further optimization. However, we respectfully suggest that evaluating the merit of a temperature compensation architecture based solely on a single TCF figure of merit is incomplete, as a fair comparison must account for fundamental acoustic dispersion relations and other performance trade-offs.

First, in principle, the formalism of TCF of the SAW resonators is defined as the derivative of frequency f to temperature T, which can be written as:

$$TCF = \frac{1}{f}\frac{\partial f}{\partial T} = \frac{1}{V_p}\frac{\partial V_p}{\partial T} - \frac{1}{\lambda}\frac{\partial \lambda}{\partial T} \quad \text{(R1)}$$

Where V_p refers to the phase velocity of acoustic wave and λ is the wavelength. The first term on the right of Equation #R1 is the temperature coefficient of velocity (TCV), which is inherently dispersive (i.e., dependent on λ). Consequently, comparing TCF values reported at different wavelengths does not fairly assess the intrinsic temperature stability offered by an

[Figure Redacted]

Figure. R45. Measured f_a shift of resonators based on (a) $LiNbO_3$/60°Y90°X-Quartz and (b) $LiNbO_3$/69°Y90°X-Quartz as a function of temperatures for different wavelengths. (d) Fitted TCF_a and (d) TCF_r of resonators on different acoustic wave platforms, including $LiNbO_3$/69°Y90°X-Quartz, $LiNbO_3$/60°Y90°X-Quartz, $LiNbO_3$/AT-Quartz, and $LiNbO_3$/YZ-Quartz with varying wavelengths. All data are from [1].

architectural approach. A fair comparison must, therefore, be conducted on a common baseline: at the same operating wavelength (λ). This principle is well-established in previous works published on high-impact journals. As a representative example, Pan et al., systematically demonstrated how TCF varies with λ across different platforms, including $LiNbO_3$/69°Y90°X-Quartz, $LiNbO_3$/60°Y90°X-Quartz, $LiNbO_3$/AT-Quartz, and $LiNbO_3$/YZ-Quartz (see Fig. R45). Accordingly, our state-of-the-art analysis in the main text and supplementary information exclusively for acoustic wave devices operating at the same wavelength λ. This provides an equitable benchmark for evaluating the temperature stability of different technological approaches, including our own.

Second, the temperature compensation effect in TC-SAW originates from the positive temperature coefficient of elastic stiffness (TCE) of SiO_2, which can compensate for the

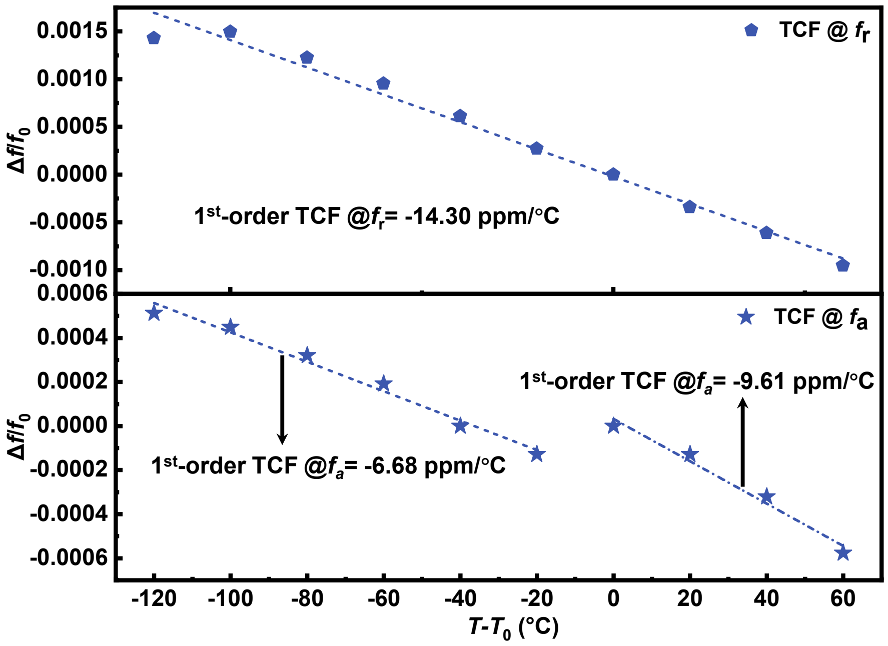

Figure. R46. Extracted relative frequency drifts for f_r and f_a under temperature variation. The fitted first-order TCF at f_r and f_a are also indicated.

negatively large TCE of $LiNbO_3$. Consequently, with deposited SiO_2 getting thicker, the TCF becomes smaller with significantly sacrificing k_t^2 (an important metric for development of high-performance acoustic devices). As such, the performance ceiling of a filter is determined by the figure of merit (FoM, FoM = Bode-Q × k_t^2) of its resonators, which is the primary determinant of achievable filter quality.

In response to the reviewer's suggestion, we have conducted a comparative analysis with a commercial TC-SAW resonator, including thermal stability, admittance, and power durability measurements. The device under test (λ = 4.4 μm) comprises a standard 128°Y-cut $LiNbO_3$ substrate with a ~1.9 μm SiO_2 temperature compensation layer and 100 nm-thick IDTs. The measured frequency drift versus temperature (Fig. R46) reveals a distinctive, piecewise-linear discontinuity in the f_a shift between 0°C and 20°C. Accordingly, the TCF at f_a was separately fitted for the ranges -100°C to 0°C and 0°C to 80°C, yielding values of -6.68 ppm/°C and -9.61 ppm/°C, respectively. The TCF at f_r is -14.3 ppm/°C. In comparison, our LAW transducer exhibits TCF values of -21.8 ppm/°C (f_r) and -13 ppm/°C (f_a). This comparability is notable given that our device operates at a significantly smaller wavelength, which theory predicts should lead to a substantially larger TCF (as illustrated in Fig. R45). Additionally, our platform demonstrates a significantly higher k_t^2: 17.7% versus 13.46% for the TC-SAW, representing a 32% enhancement that is promising for achieving wider bandwidth.

A direct power durability comparison was also carried out on the commercial TC-SAW device using our established protocol. Strictly speaking, a power test aiming to probe the intrinsic failure point of an acoustic device should target the frequency of its peak power

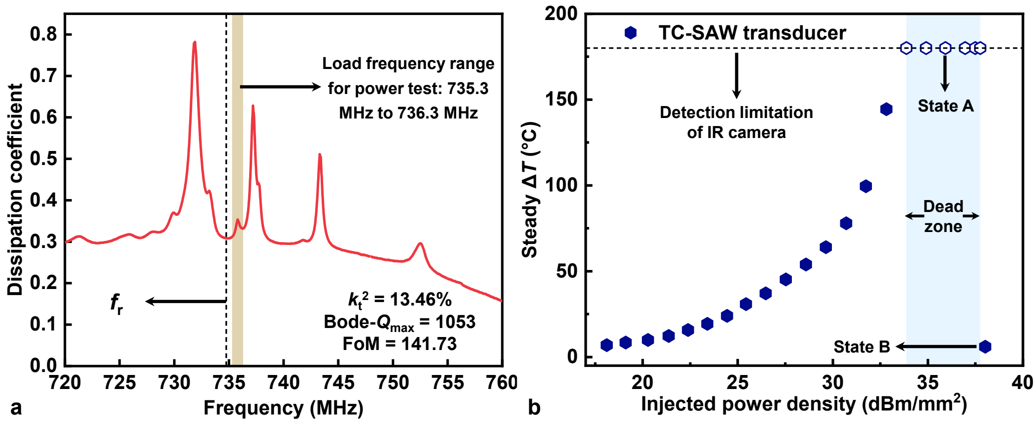

Figure. R47. **a**, Calculated power dissipation as a function of loading frequencies. The frequency range for the high-power test is indicated by the highlighted region. **b**, experimentally measured steady-state temperature rise of the TC-SAW DUT as a function of injected power density. The fitted first-order TCF at f_r and f_a are also indicated. Temperature data between 33.87 dBm/mm^2 to 37.76 dBm/mm^2 exceed the IR camera's upper detection limit, marking a measurement dead zone.

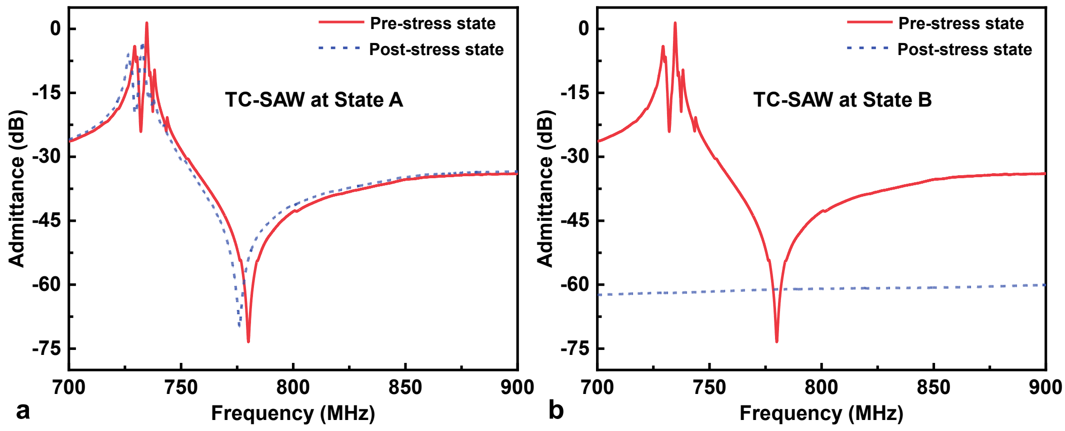

Figure. R48. [In our setup] **a**, Admittance comparison of the TC-SAW DUT before the power test and after subjection to an injected power density of 35.92 dBm/mm^2 (State A). **b**, Admittance comparison before the power test and after the final failure at State B (37.76 dBm/mm^2).

dissipation. Nevertheless, to ensure a fair comparison across platforms, we deliberately selected a frequency range with identical power dissipation for testing the TC-SAW device, as shown in Fig. R47. Key performance characteristics of the TC-SAW DUT are also included. Consistent with our methodology, the injected power is defined as the power delivered to the DUT (incident power minus reflected power). The power density is calculated using this value over the entire rectangular active area, encompassing the IDTs, reflectors, and bus lines.

Fig. R47b plots the steady-state temperature rise versus injected power density. At an injected power density of 32.82 dBm/mm^2, the TC-SAW device temperature rose to 144.4 °C. This contrasts directly with our TF-SAW (12.8 °C at 33.22 dBm/mm^2) and LAW (4.6 °C at 32.94 dBm/mm^2) platforms, highlighting its poor thermal management rooted in bulk $LiNbO_3$ and unoptimized thermal boundaries. Due to the upper detection limit (200 °C) of our IR camera,

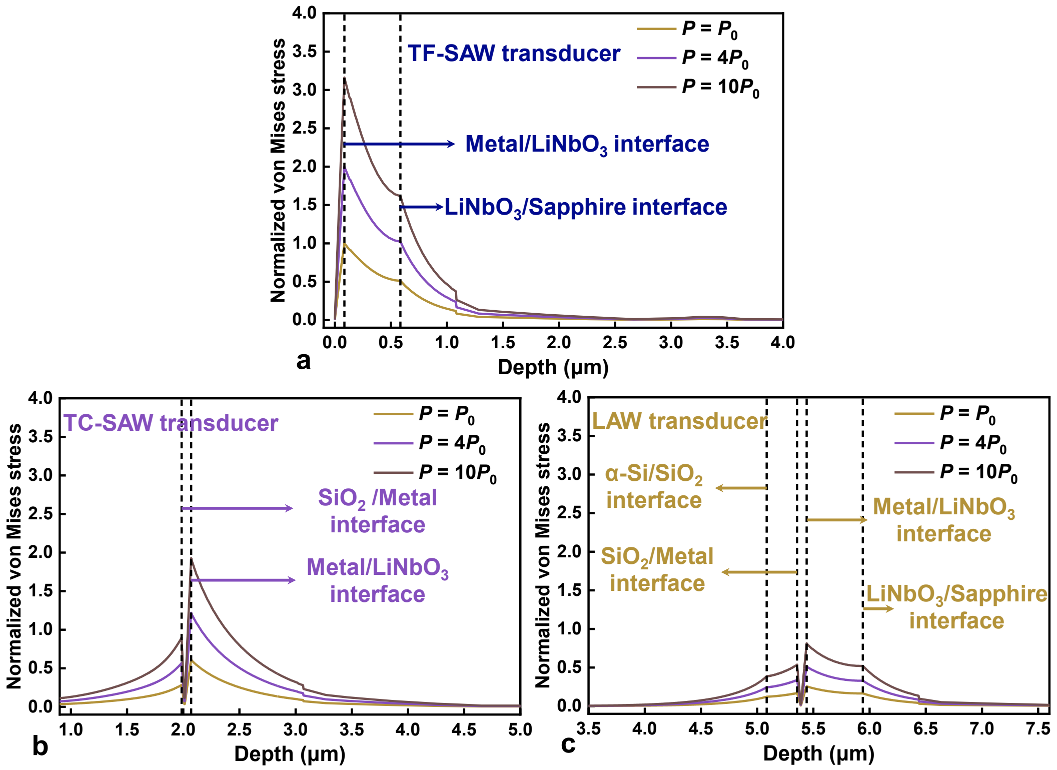

Figure. R49. Depth profiles of the extracted von Mises stress for **(a)** baseline $LiNbO_3$/Sapphire TF-SAW, **(b)** $SiO_2/LiNbO_3$ TC-SAW, and **(c)** our proposed α-Si/$SiO_2/LiNbO_3$/Sapphire LAW DUTs. All mechanical stress profiles are normalized to the maximum stress in the baseline $LiNbO_3$/Sapphire TF-SAW DUT **(a)** at reference power, P_0. Key interfaces are marked by dashed lines.

temperature measurements entered a "dead zone" for power densities between 33.87 and 37.76 dBm/mm^2. We therefore paused the test at 35.92 dBm/mm^2 (State A) — already above the TF-SAW transducer's threshold—to measure admittance characteristics (Fig. R48a). This revealed permanent frequency shifts ($\Delta f_r \approx$ 2.2 MHz, $\Delta f_a \approx$ 3.88 MHz). This shift points to a change in the acoustic boundary conditions, as purely electrical degradation would not cause such a frequency drift. The device ultimately failed at 37.76 dBm/mm^2 (State B), marked by a sudden temperature collapse from >200 °C to 26 °C and confirmed by degraded admittance (Fig. R48b). We thus define 37.76 dBm/mm^2 as the TC-SAW's power density threshold under these testing conditions. Although the IDT in the TC-SAW architecture is likewise covered by a 1.9 μm-thick temperature-compensating SiO_2 top layer, its corresponding power density threshold (37.76 dBm/mm^2) represents a significant 7.85 dB difference compared to the 45.61 dBm/mm^2 threshold achieved by our proposed LAW platform.

To fundamentally understand the power-handling limits across the tested acoustic platforms, we performed a finite-element analysis of the von Mises stress distribution. For each platform, the mechanical stress profile along the depth (from the top surface to the bottom boundary) was evaluated at the frequency corresponding to its weakest failure point, with all geometrical features held identical in the simulations. In Fig. R49, all normalized depth profiles

of von Mises stress are presented relative to the maximum stress value obtained in the baseline $LiNbO_3$/Sapphire TF-SAW transducer at a reference input power, P_0. Since the mechanical stress is proportional to the square root of input power (as validated in Fig. R49), these differences directly correlate with power-handling capability. Notably, the device on the $SiO_2/LiNbO_3$ TC-SAW transducer exhibits higher mechanical stress within the cavity at the same injected power compared to that of our LAW platform. While the commercial TC-SAW device, mounted a 1.9 µm-thick SiO_2 top layer, reduces the von Mises stress to approximately 3/5 of the baseline, our proposed LAW platform achieves a drastically lower and more uniform stress distribution, with a value of only 1/4 of the baseline stress at P_0. The advantage of our architecture becomes even more pronounced under a higher power density. Notably, at an input power ten times higher ($10P_0$), the maximum stress in our LAW platform remains lower (~0.8×) than the stress in the baseline $LiNbO_3$/sapphire TF-SAW transducer at its reference power P_0. In contrast, the TC-SAW platform already exceeds the baseline stress level (~1.22×) at only $4P_0$. This analysis proves that the LAW architecture is not a simple replacement of top SiO_2 layer. Instead, it is a targeted design that enhances power capacity by redistributing and mitigating mechanical stress at the fundamental level, thereby directly inhibiting the driving force for acoustomigration. This represents a paradigm of functional co-designing process that moves beyond simple confinement to achieve holistic performance gains.

[1] Xiao, B. et al. "Anisotropy-matched LN/Quartz heterostructure with inherent spurious mitigation for wideband SAW devices." *IEEE Trans. Microw. Theory Tech.* **73**, 10080–10094 (2025).

ACTION TAKEN:

We have strengthened the comparative analysis by adding TCF data from additional state-of-the-art acoustic platforms to Fig. 4h and Supplementary Fig. S11. This provides a broader and more equitable benchmark for evaluating temperature stability performance.

4)The surface area of resonators is not typically the key factor in power handling. Please demonstrate filters and report on the filter area and device performance.

AUTHOR RESPONSE:

We thank the reviewer for raising this critical point regarding the failure mechanisms (key factors in power handling). The failure mechanisms are related to the internal von Mises stress distribution and thermal management. We showed detailed characteristics of failure phenomena in Supplementary Information.

If the "surface area" in the reviewer's comment refers to the definition of injected power

density. We fully agree that the choice of reference area for power density calculation requires careful justification, and we appreciate the opportunity to clarify our methodological reasoning.

Our study aims to set out a route for overcoming the intrinsic power handling bottlenecks of IDT-based microacoustic transducers by engineering the internal von Mises stress distribution and improved thermal management. A central challenge in such work is to isolate improvements stemming purely from device architecture from those influenced by system-level design choices. Performing power tests at the filter level introduces significant non-architectural variables—such as layout-dependent power flow distribution and potential current/voltage division effects—that can obscure the fundamental contribution of the resonator design itself. For instance, as detailed in our response to Comment #2, merely changing the electrical connection configuration of a filter altered its power handling by 3.45 dB, highlighting a pronounced sensitivity to layout rather than to resonator architecture. Similarly, Dong et al., have experimentally demonstrated that a squared array-based topology enhance total power handling by 8.8-dB at the filter level under the same effective resonator area and technology, simply by distributing power across multiple resonators without improving the intrinsic power density thresholds of any individual device [1].

Thus, to cleanly benchmark architectural innovation in boundary-condition engineering, we adopt the active transduction area of the resonator, including the IDT, reflectors, and busbars, as the normalization reference for power density. This approach is well established in the literature and enables a consistent, device-level comparison across acoustic platforms [2-5].

Last, we acknowledge the reviewer's related concern regarding possible port impedance mismatch at the single-transducer level. To address this, we explicitly define the injected power as the delivered power (incident power minus reflected power), thereby rigorously accounting for mismatch effects. That being said, the potential issue of impedance mismatch is controlled and does not introduce bias into our comparative analysis.

Filter-level performance is not the focus of this work and is largely determined by circuit topology (as discussed in our response to Comment #2, where modern hybrid filter technologies employ integrated inductors and capacitors to enhance power handling), and we believe that foundational advances must first be established at the transducer level, where the fundamental physical limits of power handling are defined. Moreover, comparing transducer power handling across different filter topologies is inherently unfair, and it is also impractical for academic studies to replicate proprietary, product-level topologies used in industry for meaningful comparison. To reiterate with utmost respect, filter topology is not the focus of this article.

We thank the reviewer again for this valuable comment, which has helped us to clarify the rationale behind our chosen metric and to strengthen the presentation of our power-handling analysis.

[1] Wang, W. et al. "Ultra-high power handling capability bulk acoustic wave filters using square array topology." *J. Micromechanics Microengineering* **35**, 085009 (2025).
[2] Wen, Z. et al. "A High Power-handling Laterally-excited Bulk Acoustic Resonator with Scattering Vias in Double-layer Electrodes over +35 dBm." In *Proc. 2024 IEEE Ultrasonics, Ferroelectrics, and Frequency Control Joint Symposium (UFFC-JS)* 1–4 (2024).
[3] Sui, D. et al. Miniaturized A1 mode acoustic resonators and filters using inverted T-shaped electrodes. *IEEE Trans. Microw. Theory Tech.* **73**, 10071–10079 (2025).
[4] Ivira, B. et al. "Self-heating study of bulk acoustic wave resonators under high RF power." *IEEE Trans. Ultrason. Ferroelectr. Freq. Control.* **55**, 139–147 (2008).
[5] Hassine, N. B. et al. "Self heating under RF power in BAW SMR and its predictive 1D thermal model." In *Proc. 2009 IEEE International Frequency Control Symposium Joint with the 22nd European Frequency and Time forum* 237–240 (IEEE, 2009).

There are still minor technical issues in the work that require further revision. However, those might be less of a concern than the power density testing in the manuscript right now. The reviewer does not believe the paper should be published before a filter demonstration is built and tested at high power levels.

AUTHOR RESPONSE:

We thank the reviewer for the valuable suggestions. Although this work is not focused on RF filters, we have, out of respect, performed power analyses on different filter types to demonstrate that direct evaluation of transducers' power handling is appropriate.

We also aim for this work to provide a methodology for directly characterizing the power handling capability of transducers, without the need to construct complex circuit topologies such as ladder filters. We note that comparing transducer power handling across different filter topologies is inherently unfair, and it is also impractical for academic studies to replicate proprietary, product-level topologies used in industry for fair comparison. While industry efforts typically focus on optimizing circuit topology to enhance power handling, our work instead emphasizes a more fundamental, transducer-level investigation. These two approaches are complementary and, taken together, can ultimately improve the power handling capability of practical filters, an outcome that may align with the reviewer's interests.

****

Reviewer #4 (Remarks to the Author):

****

This manuscript presents a highly valuable acoustic wave transducer design that employs double-sided boundary engineering across mechanical, thermal, and electrical domains. Its most significant contribution is successfully challenging the long-standing paradigm that a top air boundary is irreplaceable for acoustic energy confinement. The work establishes a robust methodology for high-power acoustic wave applications, demonstrating performance superior to other state-of-the-art piezo-on-insulator platforms. The proposed device topology shows clear potential for improved temperature compensation and, owing to the remarkable wave confinement, future high-density integration with 3-D thermal management.

The analysis of failed devices in the supplementary materials is comprehensive and clearly demonstrates the suppression of acoustomigration. The demonstrated breakdown mechanism in the proposed LAW transducer is also first reported for acoustic transducers, providing new insight into how to improve power density.

For these reasons, I am pleased to recommend publication in *Nature Communications* after the following minor revisions are addressed:

AUTHOR RESPONSE:

We thank Reviewer #4 for the very positive evaluation of our work.

(1) The spurious responses in acoustic wave transducers would be unacceptable for practical applications, such as interactions between quantum components, where a clean frequency response is highly important for system fidelity and coherence times. The authors should comment on this limitation within their platform and suggest potential pathways to achieve a spurious-free response.

AUTHOR RESPONSE:

We agree completely that suppressing spurious responses is an additional and important consideration to further consolidate this work and thanks to this comment, we have now included a comprehensive analysis, which we summarize below and have now included in detail in the revised Supplementary Information, Section 6.

The spurious modes in Fig. 4c are not a fundamental limitation of the LAW concept. They originate from two distinct, well-understood sources: (i) transversal waveguide modes in the aperture (lateral) direction, and (ii) higher-order bulk waves scattered at vertical acoustic boundaries. These are categorically different from the parasitic plate modes (e.g., A0, S0, and

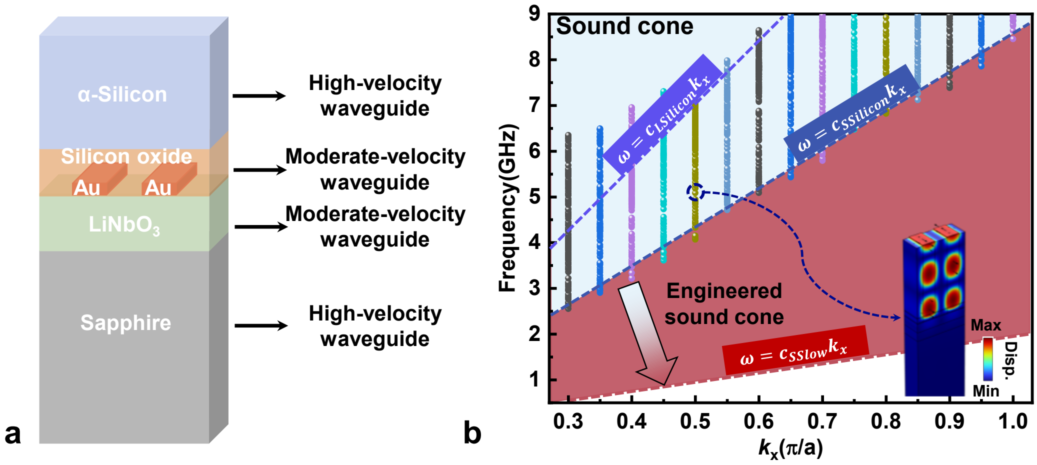

Figure. R50 **a**, Schematic of the initial vertically stacked LAW transducer and its associated acoustic waveguides. **b**, Dispersion relation of an optimized LAW transducer design for suppressing bulk vibrational modes. The light-blue region denotes the initial sound cone for free-propagating bulk modes. The red-extended sound cone represents an engineered waveguide region with sufficiently low acoustic velocity, which redirects bulk modes away from the sandwiched resonator cavity.

SH1 mode in the baseline TF-SAW or SiO_2-overcoated TF-SAW devices) that our LAW architecture was designed to suppress, as demonstrated in Figs. 2f-2g and 4a in the main text.

Critically, both types of spurious responses are addressable through established and novel design strategies. For transversal modes, proven techniques on thin-film piezoelectric platforms, including piston mode designs [1], tilted electrodes [2, 3], and slowness curve modulation [4], can effectively flatten the lateral velocity profile to suppress them.

As for higher-order bulk waves, we have developed a suppression strategy based on mechanical bandgap engineering intrinsic to the layered architecture. More specifically, the LAW platform can be conceptualized as a vertical stack of different acoustic waveguides, as shown in Fig. R50a. Given dispersion relationship for each mode, the targeted SH0 mode is well-confined within the $LiNbO_3$ cavity, while other higher-order bulk waves propagate towards the upper SiO_2 and α-Si layers. To suppress those unwanted spurious waves, we engineer the system's sound cone in $\vec{k}$-space to broaden the available phase-velocity range for parasitic modes, as illustrated in Fig. R50b.

In this engineered sound cone, initial bulk modes vibrating in the upper SiO_2 and α-Si waveguides propagate into a slower wave layer, which sits on the top of the LAW structure (Fig. R51a). At the same time, the targeted mode is confined individually beneath the SiO_2 and α-Si waveguides. Here, photoresist (PR) is selected as the uppermost slow wave medium. Finite-element simulations confirm the principle: adding a PR waveguide significantly suppresses the bulk-wave spurs without degrading the targeted mode's effective electromechanical coupling (k_t^2) and Q (Fig. R51b).

We experimentally validated this concept by coating a 5.3-µm-thick AZ® 4620 PR layer

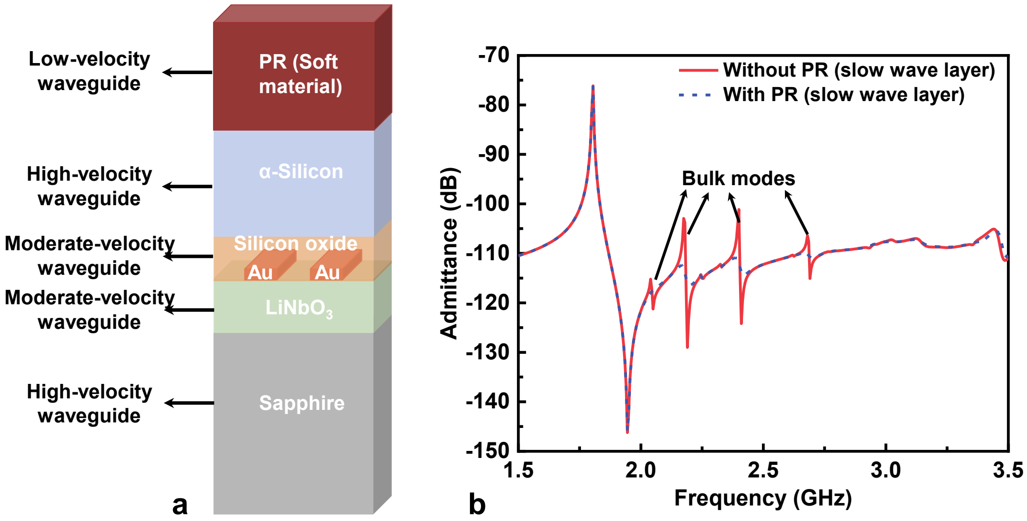

Figure. R51. **a**, Schematic of the optimized LAW transducer design for bulk mode suppression, showcasing the associated acoustic waveguides. **b**, Admittance curves for the LAW transducer design with and without a slow-wave photoresist (PR) waveguide.

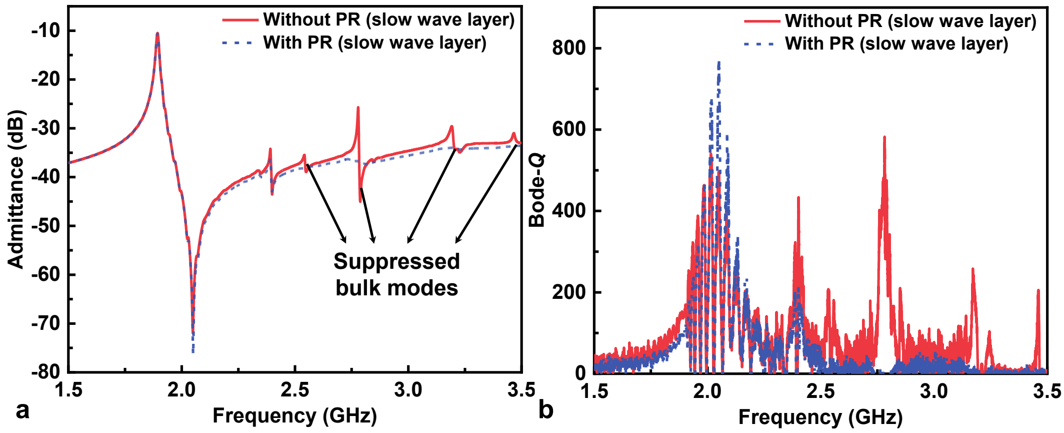

Figure. R52. **a**, Measured admittance curves and **b**, extracted Bode-Q characteristics from one-port measurement, comparing designs with and without the slow-wave PR waveguide. The suppression of bulk modes through engineered mechanical boundaries increased the Bode-Q_{max} of the targeted mode from 541 to 776.

on the LAW platform (with probing pads exposed for electrical contact). The measured admittance shows effective suppression of the bulk modes (Fig. R52). Crucially, this approach improves the device's key performance: the maximum Bode-Q of the target mode increased from 541 to 776, while spurious responses were reduced to negligible levels (Fig. R3b). Wavelength-dependent measurements further confirm the robustness of this mechanical boundary condition design, showing consistent improvements in admittance ratio (AR) and Bode-Q (Fig. R53). A full summary of performance metrics, including AR, Bode-Q_{max}, k_t^2 and the figure of merit (FoM = Bode-$Q \times k_t^2$), across wavelengths further confirms the significant advance and potential of mechanical boundary engineering (Fig. R54).

Importantly, this spurious-mode suppression strategy does not compromise the fundamental power-handling advantage of the LAW architecture. The maximum von Mises

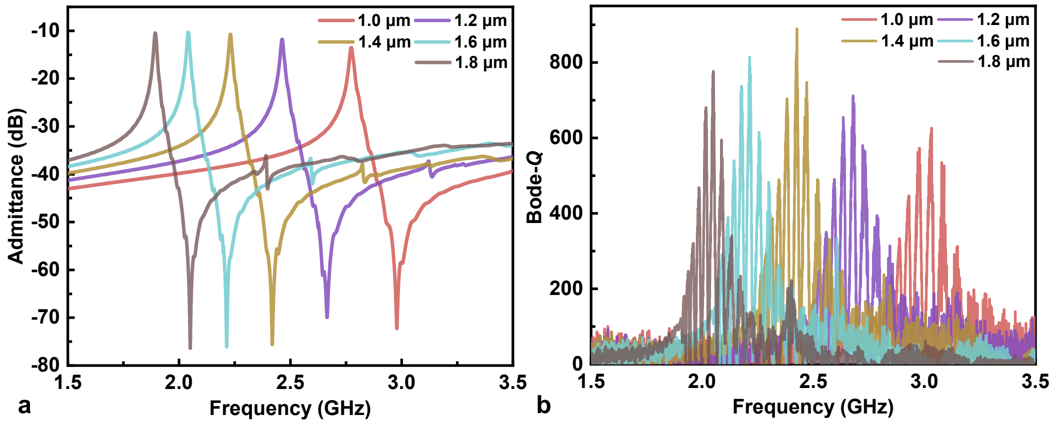

Figure. R53. Wavelength dependence of the **(a)**, measured admittance curves and **(b)**, extracted Bode-*Q* characteristics for engineered LAW transducers with the slow-wave PR waveguide.

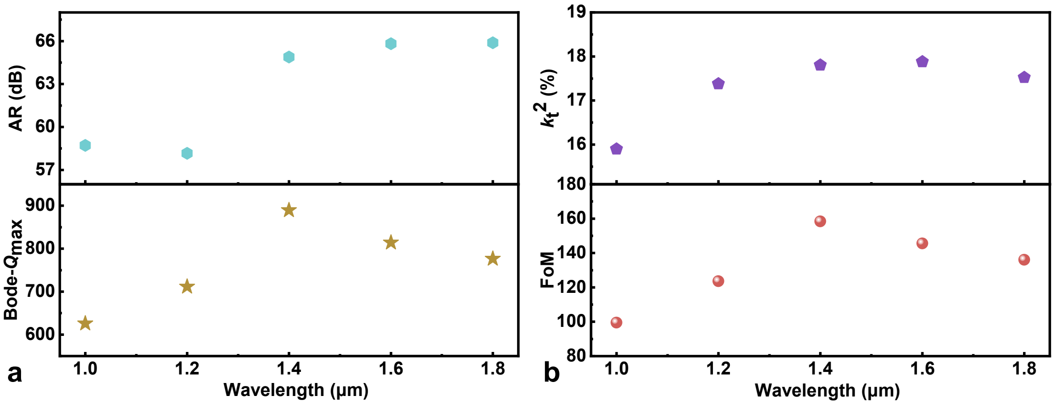

Figure. R54. Key metrics extracted across wavelengths for the engineered LAW transducer with the slow-wave PR waveguide, including **a**, admittance ratio (AR) and Bode-Q_{max}, as well as **b**, k_t^2 and figure of merit (FoM).

stress at the critical metal/$LiNbO_3$ interface remains unchanged with the addition of the PR waveguide, indicating a negligible impact on power durability. To maintain the logical flow of the main manuscript, the detailed discussion of this bulk-wave suppression strategy is provided in the revised Supplementary Information Section 6. This work demonstrates that the observed spurious responses are not a fundamental limitation but are addressable through further engineering of the acoustic boundaries, underscoring the advanced design flexibility of the LAW platform.

[1] Hashimoto, K. et al. "Revisiting piston mode design for radio frequency surface acoustic wave resonators." In Proc. *2022 IEEE MTT-S International Conference on Microwave Acoustics and Mechanics* (IC-MAM) 60–63 (IEEE, 2022).

[2] Xu, H. et al. "SAW filters on $LiNbO_3$/SiC heterostructure for 5G n77 and n78 band spplications." *IEEE Trans. Ultrason. Ferroelectr. Freq. Control* 70, 1157–1169 (2023).

[3] Qian, F., Zheng, J., Xu, J. & Yang, Y. "Heterogeneous interface-Enhanced thin-film SAW devices using Lithium Niobate on Si." *IEEE Microw. Wirel. Technol. Lett.* 35, 123–126 (2025).

[4] Shen, J. et al. Suppressed transverse mode generation in TF-SAW resonators based on $LiTaO_3$/Sapphire. *IEEE Electron Device Lett.* 45, 2241–2244 (2024).

ACTION TAKEN:

We have now included detailed discussions and added new experimental data on the spurious modes' suppression in the revised Supplementary Information, Section 6.

(2) Another important requirement in many applications is the need to accurately set an acoustic wave device's frequency to a predetermined value. Given that the operating frequency increases by ~25% compared to the SAW transducer in Fig. 4a, please clarify the relationship between the operating frequency and the top cladding layer thickness.

AUTHOR RESPONSE:

We thank the reviewer for raising this critical point regarding frequency control and trimming, which is indeed essential for practical applications. In direct response, we have systematically investigated the relationship between the operating frequency of our LAW platform and the thickness of the top α-Si stress-manipulation layer.

As shown in Fig. R55, the resonant frequency of the fundamental layered SH0 mode increases with the α-Si thickness, as the stress-manipulation layer progressively modifies the effective Young's modulus and confines more energy within the resonator cavity. Notably, this frequency shift saturates when the α-Si thickness exceeds approximately 0.6 μm. Beyond this point, further increases in thickness yield negligible changes in the resonant frequency. This saturation behavior is elucidated by the simulated mode profile (inset of the figure for a 4-μm-thick layer), which demonstrates that the acoustic energy is effectively confined at the IDT/$LiNbO_3$ interface, with the strain field decaying rapidly into the stress-manipulation layer. Therefore, beyond the critical thickness, the stress-manipulation layer acts as a semi-infinite medium from the perspective of the guided mode, making the frequency independent of further thickness increases. This well-defined saturation characteristic provides a reliable and practical route for setting the operating frequency to a pre-determined target in a manufacturing environment. For the frequency trimming process, it can be divided into two steps: the baseline SAW device (without the α-Si stress-manipulation layer) is first fabricated and measured. Its frequency can be finely adjusted to a desired target slightly below the final intended frequency using established trim techniques [1]. The stress-manipulation layer is then deposited to a thickness greater than the critical saturation threshold. This step induces a predictable, one-time upward frequency shift to the saturated value based on the fabrication data library, which is solely determined by the material stack's properties and is immune to small variations in the final α-Si thickness.

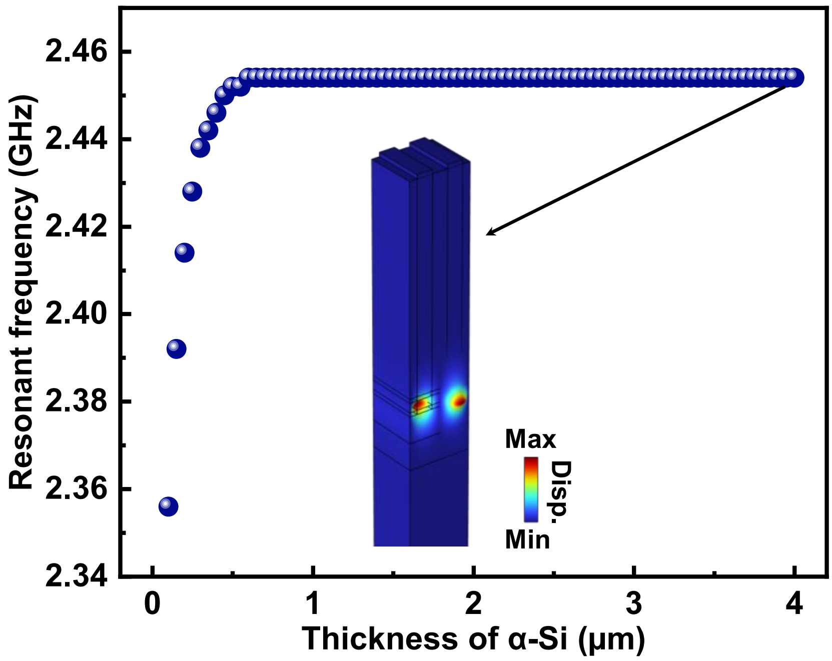

Figure. R55. Resonant frequency dependence on the α-Si top-cladding thickness and corresponding mode profile at a thickness of 4 μm.

We thank the reviewer for this insightful question, which has allowed us to strengthen the discussion on the practical application of our technology.

[1] Aliev, V. S. & Avramov, I. D. "Precision frequency trimming of SAW and STW resonators using Xe^{+} heavy ion bombardment." *IEEE Trans. Ultrason. Ferroelectr. Freq. Control.* **41**, 694–698 (1994).

ACTION TAKEN:

The suggested clarification on the frequency versus top-layer thickness relationship, including the velocity saturation effect, has been provided in the revised Supplementary Information, Section 4.

(3) To supplement the cross-sectional analysis, top-view images of the failed devices at State C and D should be provided to offer further insight into their mechanical conditions.

AUTHOR RESPONSE:

We thank the reviewer for the valuable suggestion to provide top-view images of the failed devices, which can absolutely complement the cross-sectional analysis. As requested, we have added a detailed post-failure SEM characterization.

Comparative top-view images of DUTs at their failed states (State B for TF-SAW, State D for LAW) are presented in Fig. R56, providing clear and distinct insights into their mechanical failure modes. For TF-SAW transducer at State B (Fig. R56a and R56b), the device

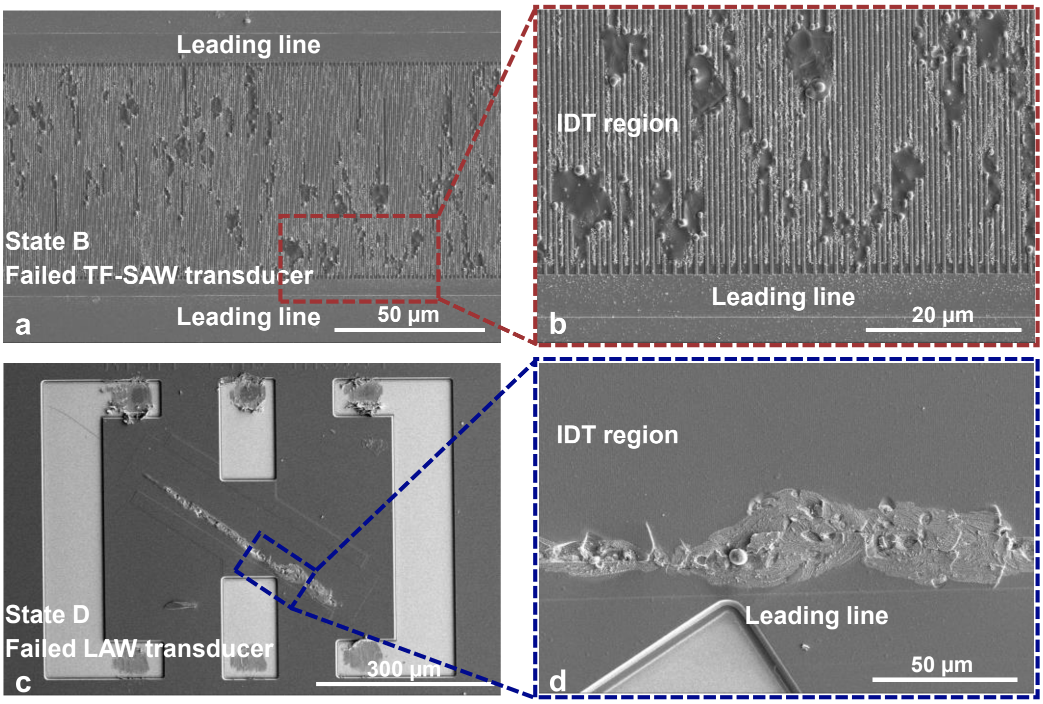

Figure. R56. **a**, A zoomed-out view showing random and widespread damage across the entire transduction region for TF-SAW DUT at State B. **b**, A magnified view revealing irregular electrode damage (including localized melting and metal balling) accompanied by numerous irreversible cracks propagating into the underlying $LiNbO_3$ substrate. **c**, A zoomed-out view demonstrating that structural damage is highly localized for LAW DUT at State D. **d**, A magnified view of the failure region near the signal output terminal, showing a well-defined, near-parallel crack along the busline direction.

failure is catastrophic and spatially random across the entire transduction area. The IDT fingers show widespread, irregular damage, including localized melting and balling of the metal electrodes. Additionally, this is accompanied by numerous irreversible cracks propagating randomly into the underlying $LiNbO_3$ substrate. This pattern indicates a global breakdown of the acoustic structure under excessive stress, with no clear epicenter, leading to complete device failure. In contrast, the LAW device exhibits a highly localized and ordered failure (Fig. R56c and R56d). The damage is concentrated in a specific region near the signal output terminal, manifesting as a well-defined, nearly parallel crack along the busline direction. Importantly, the vast majority of the IDT region remains structurally intact. This suggests that the failure was initiated by a localized stress concentration at that geometric feature, rather than a global overload of the acoustic resonator cavity. This observed contrast strongly indicates that the LAW architecture holds the potential for redistributing and mitigating the globally destructive mechanical stress that leads to random failure in conventional TF-SAW platforms.

We have added these figures and a corresponding discussion to the Supplementary Information. We thank the reviewer for prompting this insightful analysis, which has strengthened the evidence for our conclusions regarding failure modes on different platforms and pointed toward a potential path for future performance enhancement.

ACTION TAKEN:

Post-failure top-view SEM images for both failed DUTs have been added to the revised Supplementary Information, Sections 19 and 20.

(4) To ensure clarity in the EDS analysis, the specific line series used for the elemental data in Table S2 should be clearly indicated.

AUTHOR RESPONSE:

We thank the reviewer for this suggestion to enhance clarity. The specific X-ray emission lines (series) used for the quantitative EDS analysis in the revised Table S2 have now been clearly indicated, as shown below in Table R5.

Table R5. Elemental analysis at the five labelled sites of the failed TF-SAW transducer

	**Spectrum 1**	**Spectrum 2**	**Spectrum 3**	**Spectrum 4**	**Spectrum 5**
**Pt (M series)**	27.4 %	27.8 %	24.5 %	26.4 %	19.0 %
**Au (M series)**	22.0 %	20.4 %	21.9 %	17.8 %	23.6 %
**Nb (L series)**	11.2 %	10.3 %	14.1 %	15.2 %	19.8 %
**O (K series)**	10.0 %	11.5 %	12.2 %	13.4 %	18.7 %
**C (K series)**	15.6 %	14.5 %	12.7 %	12.8 %	11.6 %
**Cu (K series)**	7.3 %	7.7 %	7.6 %	6.6 %	6.2 %
**Al (K series)**	5.3 %	6.5 %	5.7 %	6.3 %	0 %
**Ni (K series)**	0.8 %	0.8 %	0.6 %	0.8 %	0.6 %
**Cr (K series)**	0 %	0 %	0 %	0.4 %	0.5 %
**Ga (K series)**	0.4 %	0.5 %	0.7 %	0.3 %	0 %

ACTION TAKEN:

In the revised Supplementary Table S2, the elemental composition at each analyzed site is now presented alongside the corresponding emission line series (e.g., Pt M-series, O K-series, Nb L-series), as demonstrated in the provided Table R5.

(5) Terminology (e.g., "Bode-*Q*") should be standardized for consistency throughout the main text and supplementary information.

AUTHOR RESPONSE:

We thank the reviewer for this keen observation. We acknowledge these format issues and thank you for bringing this to our attention. The manuscript has been revised and carefully checked accordingly.

ACTION TAKEN:

The terminology in the revised manuscript and Supplementary Information has been thoroughly checked and unified.

(6) Please check the equation for the Eyring's model in the Methods section.

AUTHOR RESPONSE:

We gratefully acknowledge the reviewer's careful reading. The equation has now been corrected and the manuscript thoroughly checked.

ACTION TAKEN:

The definition of the parameter T in the Eyring's equation within the Methods section has been corrected.

Peer Review File

Suppressing Acoustomigration and Temperature Rise for High-power Robust Acoustics

Response Letter: Suppressing Acoustomigration and Temperature Rise for High-power Robust Acoustics

Fangsheng Qian, Shuhan Chen, Wei Wei, Jiashuai Xu, Kai Yang, Junyan Zheng, Zijun Ren, Xingyu Liu, and Yansong Yang*

Summary of response

Dear Reviewers,

We sincerely thank all four reviewers for their continued engagement, insightful comments, and constructive suggestions throughout this revision process. Their careful reading and thoughtful comments have guided us in substantially strengthening the manuscript. We are gratified that the reviewers recognize the significance of our work, as reflected in their comments:

"The authors have addressed all of my comments satisfactorily. This is excellent work. The detailed methodology presented in the Supplementary Information for directly characterizing acoustic transducers under high-power excitation is both clear and highly valuable to the field of piezoelectric acoustics. To the best of my knowledge, no prior work has systematically introduced a framework for direct high-power characterization of acoustic transducers. This study significantly reshapes the community's understanding of acoustic transducer behavior under extreme power input and represents the first work to focus on the fundamental phenomena and physical principles governing transducers in the ultra-high-power regime." (Reviewer #1)

"The authors have done very thorough and thoughtful work addressing the reviewer comments and as a result have improved the quality and clarity of the paper." (Reviewer #2)

"The reviewer noticed the authors' significant effort during revision. The paper could be published; ... but thinks the paper is an interesting prototype for the research community and should be shared" (Reviewer #3)

"This work proposes a bottom-up approach by improving the power-handling capability of the fundamental building block—acoustic transducers. This perspective is both technically sound and highly relevant for practical applications" (Reviewer #4)

In direct response to the reviewers' latest comments, we have made the following key improvements to the manuscript and Supplementary Information:

1. **Raw von Mises stress data:** We have provided non-normalized raw stress profiles (Fig. R2), demonstrating that for small-C0 devices, stress near f_r indeed exceeds that near f_a, consistent with the reviewer's expectation, while for large-C0 devices, the maximum stress shifts toward f_a.
2. **Comprehensive power handling validation across the entire resonator band:** Following Reviewer #2's suggestion, we performed high-power characterizations on

TF-SAW and LAW devices with different C0 values (≈520 fF and ≈5.5 pF) at multiple frequencies spanning the resonator band. These measurements confirm that the LAW architecture achieves approximately an order-of-magnitude improvement across the entire operating band, rather than merely at a single frequency point (Fig. R3).

3. **Improved figure clarity:** Following Reviewer #2's suggestions, we have enlarged fonts in Fig. 2h and simplified Fig. 4h by moving detailed literature descriptions to the footnote, enhancing the readability of this manuscript.
4. **Expanded discussion of failure mechanisms:** Following Reviewer #2's suggestion, we have added electromigration as the fourth fundamental phenomenon limiting power handling in TF-SAW devices, alongside acoustomigration, thermoelastic instability, and mechanical stress accumulation.

We believe these revisions directly address the core concerns raised during the review process and have resulted in a more rigorous and complete manuscript. The additional experiments and analyses not only respond to the specific points raised but also strengthen the overall conclusions of our work. We are grateful to the reviewers for their help in refining this contribution and hope that the revised manuscript now meets the high standards of *Nature Communications*.

REVIEWER COMMENTS

****

Reviewer #1 (Remarks to the Author):

****

The authors have addressed all of my comments satisfactorily. This is excellent work.
The detailed methodology presented in the Supplementary Information for directly characterizing acoustic transducers under high-power excitation is both clear and highly valuable to the field of piezoelectric acoustics. To the best of my knowledge, no prior work has systematically introduced a framework for direct high-power characterization of acoustic transducers. This study significantly reshapes the community's understanding of acoustic transducer behavior under extreme power input and represents the first work to focus on the fundamental phenomena and physical principles governing transducers in the ultra-high-power regime.

AUTHOR RESPONSE:

We thank the reviewer for their thoughtful and encouraging feedback. We are delighted to hear that the reviewer finds our responses satisfactory and that they recognize the value of our contribution to the field. We are pleased that the reviewer appreciates the clarity and utility of the detailed methodology presented in the Supplementary Information, and we share their hope that this work will help reshape the community's understanding of acoustic transducer behavior in the ultra-high-power regime. We are sincerely grateful to the reviewer for their time, expertise, and constructive engagement throughout the review process, which have played an important role in strengthening this work.

****

Reviewer #2 (Remarks to the Author):

****

This manuscript studies improvements in power handling, temperature compensation, and *Q* factor in lithium niobate (LN) on sapphire surface acoustic wave (SAW) resonators via the inclusion of a thick, PECVD Si layer. The authors have done very thorough and thoughtful work addressing the reviewer comments and as a result have improved the quality and clarity of the paper. There are still a few items remaining that need addressing by the authors. Most important is point #1, which highlights that the authors have only measured the increase in power density for the LAW at a specific impedance that sets the power handling limits of their device at f_a. The impact of the paper would be significantly enhanced if the authors would characterize the power handling/power density across a range of static capacitance values that set the maximum power density frequency to different values between f_r and f_a. As the stress distribution of the acoustic wave, and thus the acousto-migration, surely changes between f_r and f_a. By measuring the most stressing power density case at only 1 frequency and acoustic stress profile, it is unclear if the LAW improves the power density across the entire bandwidth of the resonator which is vital for all of the cited applications.

AUTHOR RESPONSE:

We sincerely thank the reviewer for their thoughtful and constructive summary, and we appreciate the recognition of our efforts to improve the quality and clarity of the manuscript. We fully agree that demonstrating power handling enhancement across the entire resonance bandwidth, rather than at a single frequency, is essential for the impact of this work.

In the following detailed responses, we address this important point directly by presenting comprehensive high-power characterizations performed on TF-SAW and LAW devices with varying C0 values across multiple frequencies between f_r and f_a. As shown in the accompanying figures, these measurements confirm that the proposed LAW architecture achieves approximately an order-of-magnitude improvement in power density across the entire operating band, rather than merely at a single frequency point.

1. The authors have made a thorough effort responding to the prior comments about the power handling studies. I appreciate the detailed discussion but some more work is needed here. I am fully convinced that using the large capacitance device with C0 of 1202.6 pF, that the transducer fails near f_a in a 50 ohm system, as this is when the largest IDT voltage difference and acoustic stress are imparted to the resonator. I also agree that the power handling in this region is limited by acousto-migration, as the authors have shown highly convincing evidence of this. I also agree that the power

handling of a filter is set by the power density of the constituent resonators in the filter and that arraying resonators, etc., can be used to tailor the power handling by altering the power distribution among resonators. But filter topology cannot be used to circumvent the power density of its constituent resonators, and therefore can still be used as a useful tool to study the max. power density of the resonators. The device with C0=1202 pF fails near fa in large part (maybe even entirely) because that is where the resonator impedance results in a large voltage across the IDTs and a large vibratory stress. At other frequencies the resonator is highly mismatched and the test simply cannot couple sufficient acoustic power into the resonator (see Fig. R25c). The authors readily acknowledge as much in Figure R27 and the surrounding discussion. Fig. R27 also shows that the peak stress field distribution changes with frequency based on the impedance matching. It's also normalized and I highly suspect the peak stress field is larger near f_r when compared to f_a. The authors should show the raw Von Mises stress in their next response letter. Undoubtedly, the stress field distribution in the LAW resonator changes as a function of frequency, and will be different near f_r than it is near f_a. This is true of all high coupling SAW resonators. My concern with the current work is that the power handling or power density of the LAW device is measured only near f_a, and it is claimed the limit at this frequency is indicative of the power handling or power density across the entire resonator band. I still contend that when well matched, that acoustic resonators tend to fail near f_r as this is where the current density and power density are highest!!!! The authors directly show in R27 that when C0 is lower (i.e. the resonator is smaller) the peak power dissipated in a 50 ohm system will move to lower frequency (closer to f_r). This will cause the power density to increase. I don't think the authors have adequately tested the failure near f_r because their impedance is too low and they cannot couple energy into it at this frequency. There are numerous ways the authors can characterize if their approach improves the power handling/power density across the frequency band of the resonator, rather than just at f_a (the data presented by the authors at f_a is highly convincing). The authors clearly show in Fig. R27 that resonators of multiple sizes (lower C0) could be constructed to move the frequency where the resonator is well matched, allowing the power density to be compared across frequency. It would greatly benefit the impact of the paper for the authors to measure the power density limits of resonators with lower C0 values to obtain the power density enhancements closer to f_r.

AUTHOR RESPONSE:

We sincerely thank the reviewer for the careful reading and insightful comments. We appreciate the opportunity to clarify several points regarding our power handling measurements and failure mechanism analysis.

We apologize for any lack of clarity in our previous response regarding the failure mechanism between electromigration and acoustomigration. Following the reviewer's suggestion, we conducted the high-power stress tests near the resonant frequency f_r, specifically over the range of 1.789–1.799 GHz centered at f_r. As shown in Fig. R1b–d, within this test

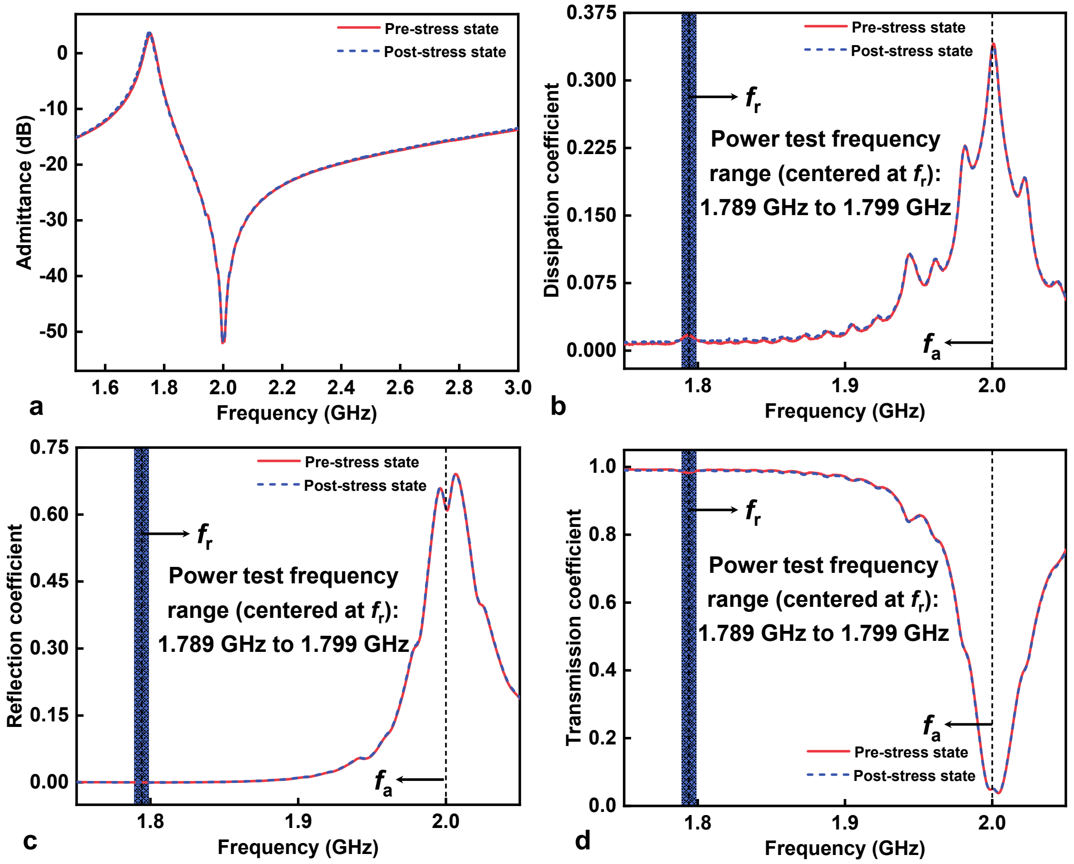

Figure. R1. **a**, Admittance curves, **b**, dissipation, **c**, reflection, and **d**, transmission coefficients for the $LiNbO_3$/Sapphire TF-SAW DUT, measured under small-signal conditions (-15 dBm) before and after being subjected to high-power stress. The high-power stress was applied at a maximum injected power density of 45.93 dBm/mm^2 over a narrow frequency range centered at the resonant frequency f_r (1.789–1.799 GHz).

frequency range (centered at f_r), the reflection coefficient is near zero (Fig. R1c), indicating excellent impedance matching, while the dissipation coefficient is minimal and the transmission coefficient reaches 0.9834, demonstrating that over 98% of the incident power is delivered into the device. Therefore, in this transducer, we can achieve good matching and sufficient power coupling near f_r.

The apparent robustness near f_r for our large-C0 DUT (C0 = 1083 pF) is not due to poor matching, but rather to the current path distribution dictated by the mBVD model. At f_r, the static branch impedance is only ~0.14 Ω, while the motional branch impedance is ~0.6 Ω. Consequently, most of the RF current flows through the static branch, effectively bypassing the motional branch where acoustomigration would occur. In contrast, for a small-C0 DUT (e.g., 96.1 fF), the static branch impedance is ~900 Ω, forcing current through the motional branch and enabling acoustomigration-induced failure. This observation reinforces our central claim: when significant acoustic energy is present in the motional branch, enabling acoustomigration-induced failure, the device becomes vulnerable. The fact that the large-C0 device handles over-45 dBm/mm^2 injected power density near f_r, despite excellent power coupling, demonstrates

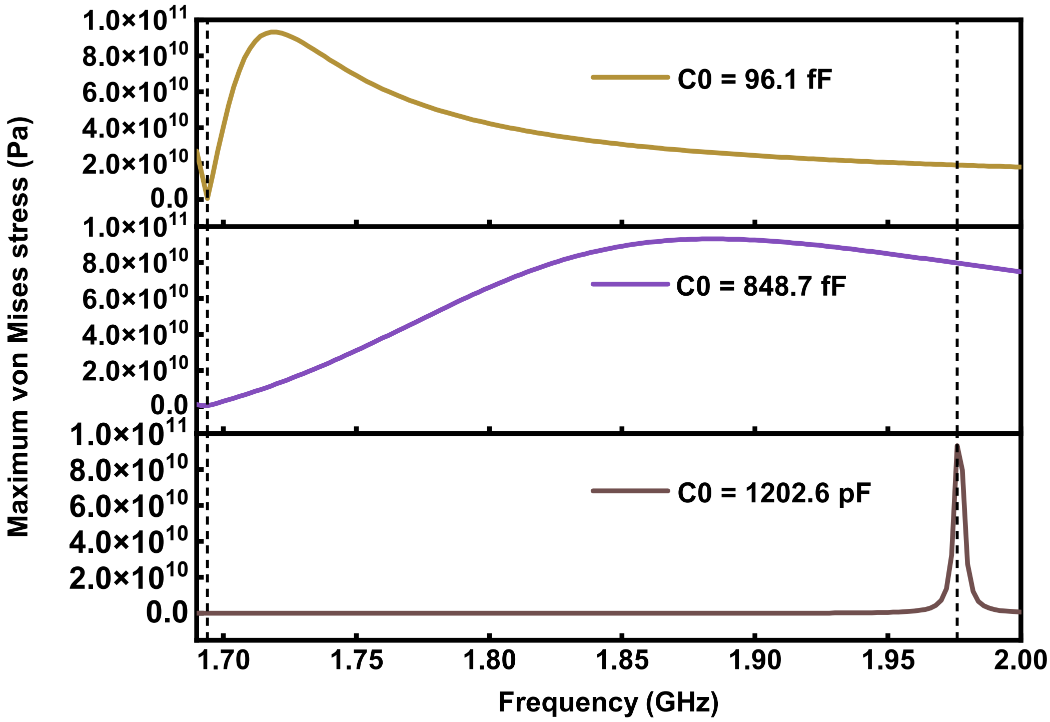

Figure. R2. Simulated von Mises stress profiles versus frequency for the $LiNbO_3$/sapphire SAW device with varied static capacitances. The maximum stress is consistently located at the metal/$LiNbO_3$ interface. All profiles are presented in absolute values (MPa).

that acoustomigration, not Joule heating, is the dominant failure mechanism in TF-SAW transducers.

Following the reviewer's suggestion, we have now provided the non-normalized raw von Mises stress data in Fig. R2. In our previous response, the stress profiles were normalized to the maximum value within the simulated frequency range for each device individually. Even in the non-normalized data presented in Fig. R2, the same trend is evident: for devices with small C0, the stress near f_r is indeed higher than that near f_a, consistent with the reviewer's expectation. However, as C0 becomes exceptionally large, the frequency corresponding to the maximum stress shifts toward f_a, while the stress near f_r becomes significantly smaller compared to that near f_a. This observation further supports our mBVD-based explanation for the shift in the failure-prone frequency region with increasing C0.

In direct response to the reviewer's valuable suggestion regarding measurements on devices with varying C0, we have now performed comprehensive high-power characterizations on TF-SAW and LAW devices with different C0 values across the resonator band. Fig. R3a presents a comparison of the injected power density thresholds for TF-SAW and LAW DUTs with C0 $\approx$520 fF, measured at three selected frequencies: f_r, the frequency of maximum dissipation (f_{max}), and f_a. The exact driving frequencies and their corresponding dissipation coefficients are indicated in the figures.

For the TF-SAW devices, the threshold injected power density at f_{max} is 28.53 dBm/mm^2, representing the most failure-prone point. At f_r, the threshold is slightly higher at

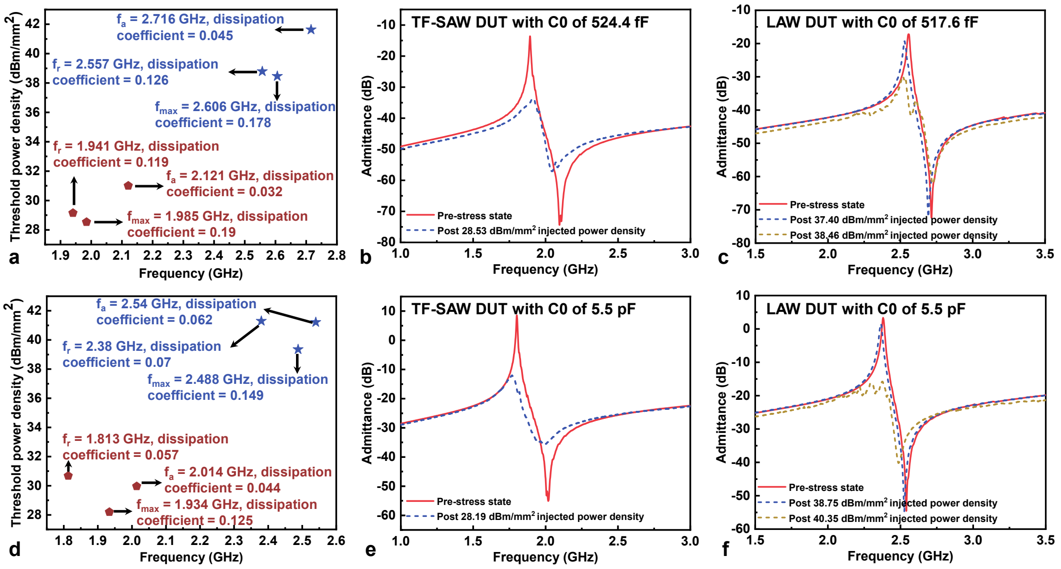

Figure. R3. **a**, Comparison of injected power density thresholds for TF-SAW and LAW DUTs with C0 ≈ 520 fF, measured at three selected frequencies across the resonator band. The driving frequencies and corresponding dissipation coefficients are indicated. **b-c**, Admittance responses before (pre-stress) and after (post-stress) high-power exposure for a TF-SAW DUT with C0 = 524.4fF **(b)** and a LAW DUT with C0 = 517.6 fF **(c)**. **d**, Same as a but for DUTs with C0 ≈ 5.5 pF. **e-f**, Corresponding pre- and post-stress admittance comparisons for a TF-SAW with C0 = 5.5 pF **(e)** and a LAW DUT with C0 = 5.5 pF **(f)**. The post-stress responses were obtained by small-signal frequency sweeps after the devices were subjected to continuous-wave high-power stress at the frequency corresponding to maximum dissipative absorption.

29.15 dBm/mm^2, while at f_a it increases to 31 dBm/mm^2 due to the lower dissipation (Fig. R3a). These results for the small-C0 device show consistency with the reviewer's expectation that failure susceptibility is highest near the frequencies where acoustic energy is most strongly coupled. For the LAW device, the thresholds at f_r, f_{max}, and f_a are 38.76, 38.46, and 41.63 dBm/mm^2, respectively. Although the improvement varies slightly across frequencies, the overall enhancement factor of approximately 11-fold for the LAW compared to the TF-SAW remains consistent with our earlier conclusions. To further illustrate the difference in power handling robustness between the two architectures, Figs. R3b and R3c show the admittance responses of the TF-SAW and LAW devices before and after high-power stress at their respective maximum-dissipation frequencies. After exposure to 28.53 dBm/mm^2, the TF-SAW device exhibits irreversible damage: spurious modes appear, the Q-factor drops significantly, the electromechanical coupling coefficient (k_t^2) decreases, and the static capacitance C0 is also reduced (Fig. R3b). In contrast, the LAW device stressed at 37.4 dBm/mm^2 shows only a minor frequency shift, while both k_t^2 and Q remain nearly unchanged (Fig. R3c). Only when the power density is increased to 38.46 dBm/mm^2 does the LAW device begin to show degradation similar to that of the TF-SAW, yet its performance (e.g., k_t^2) is still superior to the damaged TF-SAW DUT.

We extended the same measurement for devices with C0 ≈ 5.5 pF. The frequency offset

between f_r and f_{max} increases with C0: for C0 ≈ 520 fF, the offsets are 44 MHz for TF-SAW and 49 MHz for LAW; for C0 ≈ 5.5 pF, they become 121 MHz for TF-SAW and 108 MHz for LAW (Fig. R3d). At the maximum-dissipation frequency, the LAW again demonstrates a marked improvement: the critical power density for the TF-SAW is 28.19 dBm/mm^2, while the LAW withstands 38.75 dBm/mm^2 before showing any notable change, an enhancement factor of 11.38-fold. Post-stress admittance comparisons confirm that at 28.19 dBm/mm^2 the TF-SAW is irreversibly damaged, with its admittance ratio drops to 23.8 dB and spurious modes appearing, whereas the LAW stressed at 38.75 dBm/mm^2 exhibits only a slight frequency shift with k_t^2 and Q essentially preserved. Only after exposure to 40.35 dBm/mm^2 does the LAW show similar degradation, with an admittance ratio of 25.4 dB and emerging spurious responses.

These additional measurements, conducted across different C0 values and at multiple frequencies within the resonator band, consistently demonstrate that structural innovation significantly enhances the power handling capability by approximately an order of magnitude, and that this improvement is not confined to a single frequency point. Furthermore, these transducer-level characterizations also indicate the necessity of such measurements for elucidating the competitive or synergistic interplay between acoustomigration and electromigration, which is essential for guiding future strategies to enhance power handling capability. The data fully support our original conclusion and previous responses that the proposed design effectively mitigates acoustomigration-driven failure across the entire operating band.

2. Just above Figure 1, the authors list 4 fundamental physical phenomena that prevent power scaling in SAW, but fail to mention electromigration. Since this is a very common failure mode that has traditionally limited RF SAW devices, I think it should be added here.

AUTHOR RESPONSE:

We thank the reviewer for this important and constructive suggestion. The reviewer is absolutely correct that electromigration is a well-established and historically significant failure mechanism in SAW devices, particularly under high current density conditions in the IDT electrodes. We acknowledge that our original summary of four fundamental phenomena inadvertently omitted this key factor.

Electromigration refers to the mass transport of metal atoms due to momentum transfer from conducting electrons under high DC or RF current densities, leading to void formation and ultimately open-circuit failure in the electrode fingers. While our work focuses primarily on acoustomigration, which is driven by mechanical stress associated with the acoustic wave itself, electromigration remains an important consideration, especially in narrow electrode

geometries and under high-power operation where current densities can become significant.

Following the reviewer's suggestion, we have now revised the introductory paragraph to include electromigration as the fourth fundamental phenomenon, while renumbering the subsequent points accordingly. We appreciate the reviewer's attention to detail, which has helped enhance the completeness and accuracy of our manuscript.

ACTION TAKEN:

The original text has now been corrected to: "*Fundamentally, four physical phenomena prevent power-scaling into large-signal regimes: 1) acoustomigration, driven by mechanical stress and self-heating effects, degrades electromechanical coupling by transporting metallic clusters; 2) thermoelastic instability, manifesting as frequency drift from temperature-dependent acoustic velocity and thermal expansion mismatch; 3) mechanical stress accumulation, inducing thin-film cracking or delamination; and 4) electromigration, where high current densities in the IDT electrodes induce atomic diffusion via electron momentum transfer, leading to void formation and eventual open-circuit failure. Additionally, arc discharging, another parasitic effect arising from pyroelectric charging, can be significantly mitigated by material engineering*."

3. The fonts in Fig. 2h are extremely small and should be increased.

AUTHOR RESPONSE:

We thank the reviewer for this helpful observation. Following their suggestion, we have increased the font size in Fig. 2h to ensure all labels are clearly legible. The revised figure now presents the data with improved readability while maintaining consistency with the overall formatting of the manuscript. We appreciate the reviewer's attention to detail, which has helped enhance the clarity of our figures.

ACTION TAKEN:

We have now updated Fig. 2h in the revised manuscript.

4. In the revised manuscript, the authors state that the Au electrode is stiffer than alternative metals such as Cu, Pt, and Ag. However, Au has the lowest Young's modulus of these metals, and it is considerably lower than Pt and Cu. The enhanced K^2 performance is likely due to the increased mass density. The authors should not state that Au is stiffer.

AUTHOR RESPONSE:

We thank the reviewer for this important correction. The reviewer is absolutely right: Au

actually has a lower Young's modulus (approximately 70 GPa) compared to Cu (120 GPa) and Pt (168 GPa), and therefore cannot be described as "stiffer". The enhanced electromechanical coupling performance observed with Au electrodes is primarily attributed to its higher mass density, which provides more effective confinement of acoustic energy within the piezoelectric layer. We have revised the manuscript accordingly.

ACTION TAKEN:

The original text has now been corrected to: "*Beyond stability, the choice of dense electrode material such as Au provides additional benefits for the transducer's* k_t^2 *performance. The IDT structure inherently shifts the mechanical stress field from the piezoelectric layer toward the electrode layer. A high-density electrode like Au enhances mass loading and couples more effectively with this stress field and the applied electric field, leading to a higher achievable* k_t^2*.*"

5. The Bode-Q_{max} values reported in Fig. 4b are highly influenced by the transverse spurious modes. The authors should construct an mBVD model that matches the device admittance and phase and then show a Bode-Q for the equivalent circuit model, as this is more likely to show the true Bode-Q of the resonant mode targeted by the authors. Undoubtedly the Bode-Q is higher for the LAW compared to the other two SAW transducers, but those narrow peak values claimed by the authors are high only due to the spurs.

AUTHOR RESPONSE:

We thank the reviewer for this constructive suggestion. Following the reviewer's recommendation, we have performed mBVD model fitting to accurately fit the admittance and phase of the main mode for each transducer, and then computed the corresponding Bode-Q curves based on the fitted equivalent circuit parameters. The updated results are now presented in the revised Fig. R4a (admittance) and Fig. R4b (Bode-Q). The fitted Bode-Q_{max} values are 305 for the conventional TF-SAW, 234 for the SiO_2-overcoated TF-SAW, and 445 for the LAW transducer. While these absolute values differ from those obtained directly from the measured data due to the influence of transverse spurious modes, the trend remains clear: the LAW architecture exhibits a significant improvement in Q_{max}, approximately 46% higher than that of

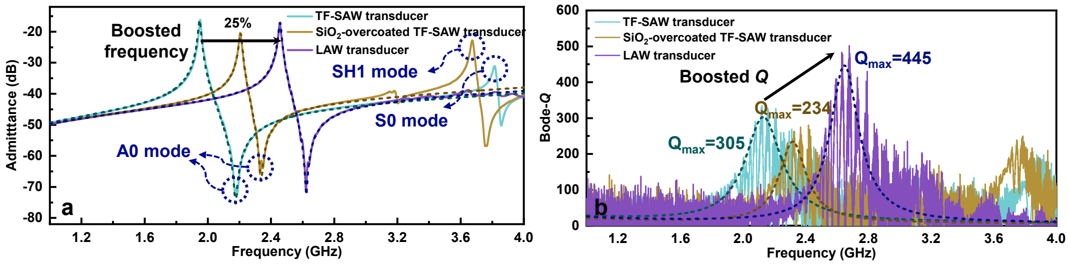

Figure. R4. Measured (solid lines) and fitted (short dash lines) **a**, admittance and **b**, corresponding Bode-*Q* curves for transducers in conventional TF-SAW, SiO_2-overcoated TFSAW, and LAW configurations. The fitted curves are obtained from mBVD model extraction of the main mode, effectively removing the influence of transverse spurious modes.

the conventional TF-SAW and nearly double that of the SiO_2-overcoated TF-SAW architecture.

ACTION TAKEN:

We have now updated Fig. 4a and 4b in the revised manuscript.

6. The presentation of Fig. 4h must be improved. It is completely unreadable in its current form.

AUTHOR RESPONSE:

We thank the reviewer for pointing out the readability issue with Fig. 4h. In our previous response, we attempted to provide a comprehensive comparison of TCF values across various device architectures from the literature, which resulted in an overcrowded figure with excessive text labels. Following the reviewer's suggestion, we have now simplified the figure (Fig. R5) by moving the detailed descriptions of each referenced work to the footnote. The revised Fig. 4h presents only essential labels directly on the plot, making the comparison clear and immediately accessible. The corresponding TCF_r comparison figure in the revised Supplementary Information has also been updated accordingly. We appreciate the reviewer's constructive input, which has significantly improved the clarity and professionalism of this figure.

ACTION TAKEN:

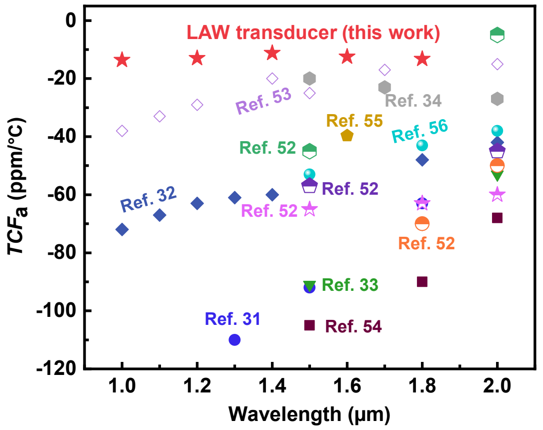

Figure. R5. Scatter plot comparison of TCF_a for the LAW transducers in this work and for advanced TF-SAW transducers reported in the literature. Data points are compiled from the following references: $LiNbO_3/SiO_2$/Quartz [31]; $LiNbO_3$/SiC [32]; $LiNbO_3/SiO_2$/Si [33]; $SiO_2/LiNbO_3$ [34]; $LiNbO_3$/69°Y90°X-Quartz, $LiNbO_3$/60°Y90°X-Quartz, $LiNbO_3$/AT-Quartz and $LiNbO_3$/YZ-Quartz [52]; $LiNbO_3/SiO_2$/SiC [53, 54]; $LiNbO_3$/SiC [55]; and $LiNbO_3/SiO_2$/p-Si/Si [56].

****
Reviewer #3 (Remarks to the Author):
****

The reviewer noticed the authors' significant effort during revision. The paper could be published; however, it would be more convincing if a filter were demonstrated and tested for power-handling or benchmarking purposes. The reviewer is not fully convinced, but thinks the paper is an interesting prototype for the research community and should be shared:

AUTHOR RESPONSE:

We sincerely thank the reviewer for acknowledging the significant effort we have made during the revision and for recognizing our work as an interesting and valuable contribution to the research community. We greatly appreciate the reviewer's professional insights and constructive suggestions regarding filter-level demonstration.

In response to this comment, we performed extensive validations at the transducer level across multiple conditions, following the suggestions of Reviewer #2. As shown in the Fig. R6, we characterized devices with different C0 values (≈ 520 fF and ≈ 5.5 pF) at multiple frequencies across the resonator band, specifically at the resonant frequency f_r, the frequency of maximum dissipation f_{max}, and the anti-resonant frequency f_a. The exact driving frequencies and their corresponding dissipation coefficients are indicated in the figure.

For the TF-SAW devices, the threshold injected power density at f_{max} is 28.53 dBm/mm^2, representing the most failure-prone point. At f_r, the threshold is slightly higher at 29.15 dBm/mm^2, while at f_a it increases to 31 dBm/mm^2 due to the lower dissipation (Fig. R6a). These results for the small-C0 device show consistency with the reviewer's expectation that failure susceptibility is highest near the frequencies where acoustic energy is most strongly coupled. For the LAW device, the thresholds at f_r, f_{max}, and f_a are 38.76, 38.46, and 41.63 dBm/mm^2, respectively. Although the improvement varies slightly across frequencies, the overall enhancement factor of approximately 11-fold for the LAW compared to the TF-SAW remains consistent with our earlier conclusions. To further illustrate the difference in power handling robustness between the two architectures, Figs. R6b and R6c show the admittance responses of the TF-SAW and LAW devices before and after high-power stress at their respective maximum-dissipation frequencies. After exposure to 28.53 dBm/mm^2, the TF-SAW device exhibits irreversible damage: spurious modes appear, the Q-factor drops significantly, the electromechanical coupling coefficient (k_t^2) decreases, and the static capacitance C0 is also reduced (Fig. R6b). In contrast, the LAW device stressed at 37.4 dBm/mm^2 shows only a minor frequency shift, while both k_t^2 and Q remain nearly unchanged (Fig. R6c). Only when the power density is increased to 38.46 dBm/mm^2 does the

LAW device begin to show degradation similar to that of the SAW, yet its performance (e.g., k_t^2) is still superior to the damaged TF-SAW DUT.

We extended the same measurement for devices with C0 ≈ 5.5 pF. The frequency offset between f_r and f_{max} increases with C0: for C0 ≈ 520 fF, the offsets are 44 MHz for TF-SAW and 49 MHz for LAW; for C0 ≈ 5.5 pF, they become 121 MHz for TF-SAW and 108 MHz for LAW. At the maximum-dissipation frequency, the LAW again demonstrates a marked improvement: the critical power density for the TF-SAW is 28.19 dBm/mm^2, while the LAW withstands 38.75 dBm/mm^{2}before showing any notable change, an enhancement factor of 11.38-fold. Post-stress admittance comparisons confirm that at 28.19 dBm/mm^2 the TF-SAW is irreversibly damaged, with its admittance ratio drops to 23.8 dB and spurious modes appearing, whereas the LAW stressed at 38.75 dBm/mm^2 exhibits only a slight frequency shift with k_t^2 and Q essentially preserved. Only after exposure to 40.35 dBm/mm^2 does the LAW show similar degradation, with an admittance ratio of 25.4 dB and emerging spurious responses. These measurements consistently demonstrate that the proposed LAW architecture achieves approximately an order of magnitude improvement in power handling capability across the entire operating band, not merely at a single frequency point. We believe this comprehensive transducer-level validation provides strong and convincing evidence for the effectiveness of our approach.

Regarding the reviewer's suggestion to demonstrate a complete filter, we fully agree that filter-level validation represents an important and logical next step. We are planning to pursue this direction in collaboration with industrial partners. Future work will involve designing and testing ladder-type filters, with detailed investigations into power flow distribution within the filter network, characterization including infrared thermal imaging, in-depth post-failure and mechanism analysis. These studies will provide valuable insights into how the transducer-level improvements translate to filter-level performance.

However, we respectfully submit that including a complete filter demonstration in the current manuscript would fundamentally shift the core narrative and make the paper overly lengthy. The present work has already dedicated substantial effort to rigorous transducer-level analysis, including comprehensive power handling tests across multiple C0 values and frequencies, detailed mBVD modeling, von Mises stress simulations, and post-failure characterizations, to establish a clear and physically grounded understanding of the underlying mechanisms. Adding a full filter demonstration would not only disrupt the logical flow but also potentially reduce the clarity and impact of the paper for its broad readership, and the scope of the readers would become narrower.

We hope the reviewer understands our rationale and agrees that the current manuscript, with its thorough transducer-level validation, already provides a compelling and impactful contribution to the field. We are committed to pursuing the suggested filter-level studies as a

natural extension of this work in the near future.

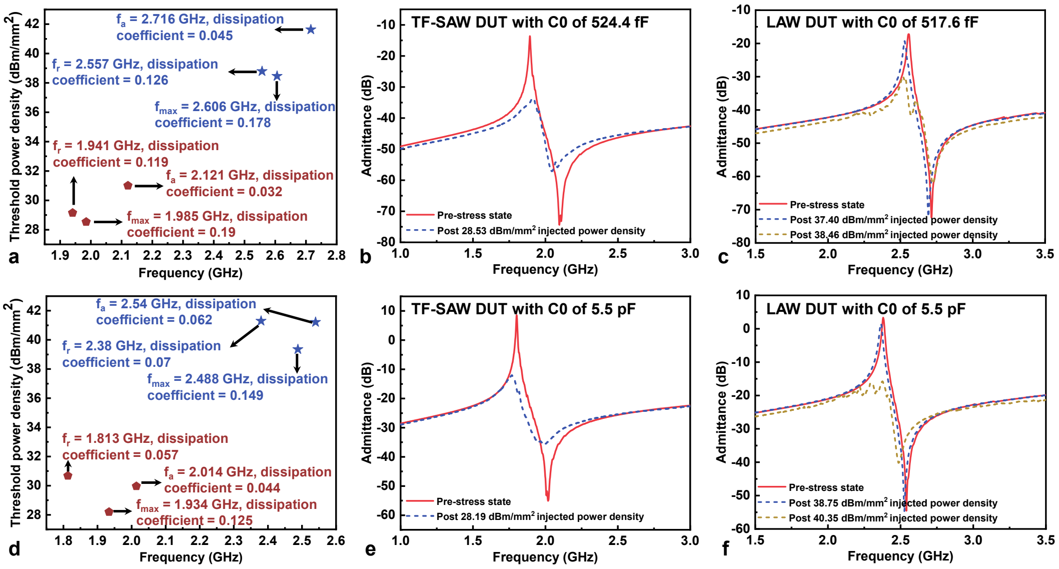

Figure. R6. **a**, Comparison of injected power density thresholds for TF-SAW and LAW DUTs with C0 ≈ 520 fF, measured at three selected frequencies across the resonator band. The driving frequencies and corresponding dissipation coefficients are indicated. **b-c**, Admittance responses before (pre-stress) and after (post-stress) high-power exposure for a TF-SAW DUT with C0 = 524.4fF **(b)** and a LAW DUT with C0 = 517.6 fF **(c)**. **d**, Same as a but for DUTs with C0 ≈ 5.5 pF. **e-f**, Corresponding pre- and post-stress admittance comparisons for a TF-SAW with C0 = 5.5 pF (b) and a LAW DUT with C0 = 5.5 pF. The post-stress responses were obtained by small-signal frequency sweeps after the devices were subjected to continuous-wave high-power stress at the frequency corresponding to maximum dissipative absorption.

****

Reviewer #4 (Remarks to the Author):

****

Thank authors for the detailed responses. While industry efforts typically focus on developing new circuit topologies to enhance high-power handling, this work proposes a bottom-up approach by improving the power-handling capability of the fundamental building block—acoustic transducers. This perspective is both technically sound and highly relevant for practical applications.

AUTHOR RESPONSE:

We thank the reviewer for this encouraging and insightful feedback. We are gratified that the reviewer appreciates our bottom-up approach, focusing on improving the power-handling capability of the fundamental transducer building block rather than relying solely on circuit-level topologies. We thank the reviewer again for their time and positive assessment of our work.

Peer Review File

Suppressing Acoustomigration and Temperature Rise for High-Power Robust Acoustics

REVIEWER COMMENTS

****

Reviewer #2 (Remarks to the Author):

****

I'd like to thank the authors for carefully considering my prior comments and performing the additional experiments on the smaller C_0 devices. In my view, these additional experiments have definitively proven the key claims of the paper. The work is indeed a huge leap forward in both the understanding of power handling limits in IHP-type SAW devices and in their ultimate power handling performance using the LAW structure reported by the authors. The paper in its current form is both of high scientific quality and of high importance to the field of acoustic resonators and filters.

AUTHOR RESPONSE:

We sincerely thank Reviewer #2 for the positive feedback and for recognizing the significance of our work. We are glad that the additional experiments were considered valuable and that the revised manuscript now meets the standards required for publication. We greatly appreciate the reviewer's time and thoughtful engagement throughout the review process.
